# Morphological diversity in true and false crabs reveals the plesiomorphy of the megalopa phase

**DOI:** 10.1038/s41598-024-58780-7

**Published:** 2024-04-15

**Authors:** Florian Braig, Carolin Haug, Joachim T. Haug

**Affiliations:** 1 Faculty of Biology, LMU Munich, Biocenter, Großhaderner Str. 2, 82152 Planegg-Martinsried, Germany; 2 GeoBio-Center of the LMU Munich, Richard-Wagner-Str. 10, 80333 Munich, Germany

**Keywords:** Zoology, Developmental biology

## Abstract

Brachyura and Anomala (or Anomura), also referred to as true and false crabs, form the species-rich and globally abundant group of Meiura, an ingroup of Decapoda. The evolutionary success of both groups is sometimes attributed to the process of carcinization (evolving a crab-like body), but might also be connected to the megalopa, a specific transitional larval phase. We investigate these questions, using outline analysis of the shields (carapaces) of more than 1500 meiuran crabs. We compare the morphological diversity of different developmental phases of major ingroups of true and false crabs. We find that morphological diversity of adults is larger in false crabs than in true crabs, indicating that taxonomic diversity and morphological diversity are not necessarily linked. The increasing morphological disparity of adults of true and false crabs with increasing phylogenetic distance furthermore indicates diverging evolution of the shield morphology of adult representatives of Meiura. Larvae of true crabs also show larger diversity than their adult counterparts, highlighting the importance of larvae for biodiversity studies. The megalopa phase of Meiura appears to be plesiomorphic, as it overlaps between true and false crabs and shows little diversity. Causes may be common evolutionary constraints on a developmental phase specialized for transitioning.

## Introduction

True and false crabs, Brachyura and Anomala (or Anomura), are the two evolutionary successful major ingroups of Meiura. With about 10,000 globally abundant formally described species, this diverse group conquered marine, limnic and terrestrial habitats^1–3^. True and false crabs form globally important fisheries^4,5^ and are often important actors in ecosystems^6,7^. To scientists they are of special interest, among others, for their shared phenomenon of carcinization, i.e., evolving a crab-like body shape (laterally widened body with the pleon tucked underneath)^8,9^. Herein, false crabs express fewer groups with truly crab-like bodies and a smaller total number of species compared to true crabs^10,11^. The fact that the crab body shape evolved several times within Meiura suggests a potential evolutionary advantage, although this topic is still under discussion^12,13^.

During the early life phases though, neither true nor false crabs assume their crab shape yet. They first undergo a planktic larval phase, the zoea^14^. Larvae in this dispersal phase have an anterior–posterior elongated body, often long spines on the shield (carapace), and use thorax exopods for locomotion^14^. After a metamorphic molt, the zoea develops into the megalopa (also named decapodid in general, or glaucothoe in Anomala)^15^, a phase specialized for the transition from planktic to benthic habitat^14^. The megalopa already looks more like a crab, with a dorso-ventrally flattened body and prominent chelae, but the pleon is not tucked underneath the body yet^14^. The megalopa in this morphology is specific to the group Meiura, although other ingroups of Decapoda express similar phases (e.g., puerulus phase and nisto phase in the group Achelata)^14,16,17^. The megalopa then undergoes another metamorphic molt, into the juvenile crab, which can sometimes still differ morphologically from fully grown adults^14,18^.

Both, carcinization and the megalopa phase, providing an additional metamorphic molt during ontogeny, are mentioned as potential reasons for the evolutionary success of both true and false crabs^1,19^. However, it is difficult to test the influence of these adaptations on the evolutionary success of the group, especially with the current lack of larval material from the fossil record^20,21^. We therefore follow another approach by comparing morphological diversity (i.e., the range of morphologies), as well as morphological disparity (i.e., the difference in morphology) between true and false crabs throughout their ontogeny and phylogeny. We perform a large-scale quantitative analysis, using the shield as a proxy for morphology, as it is the most accessible feature in crabs, is reproducible, and provides ecological as well as phylogenetic information about the organism^22^. We expect to find little diversity and overlapping morphology among larval phases of both groups due to the assumed low interspecific variability of both groups and in larval phases (Hypothesis 1)^15,23,24^. We further expect larger morphological diversity in the adult phase compared to the larval phase due to the ecological diversity of the adults (H2)^23^. Lastly, we expect larger morphological diversity in true than in false crabs due to the difference in species numbers (H3)^25^.

## Results

### The crab morphospace

The principal component analysis (PCA), performed on the results of the elliptic Fourier analysis (EFA) of the shields of true and false crabs resulted in 17 principal components (PCs) explaining over 99% of variation in the data set. The first and second PCs were plotted to visualize the morphospace, as they represent the largest amount of variation (62.3% and 13.2% respectively). For all following quantitative analyses, all 17 PCs were used as input data.

**Figure 1:**
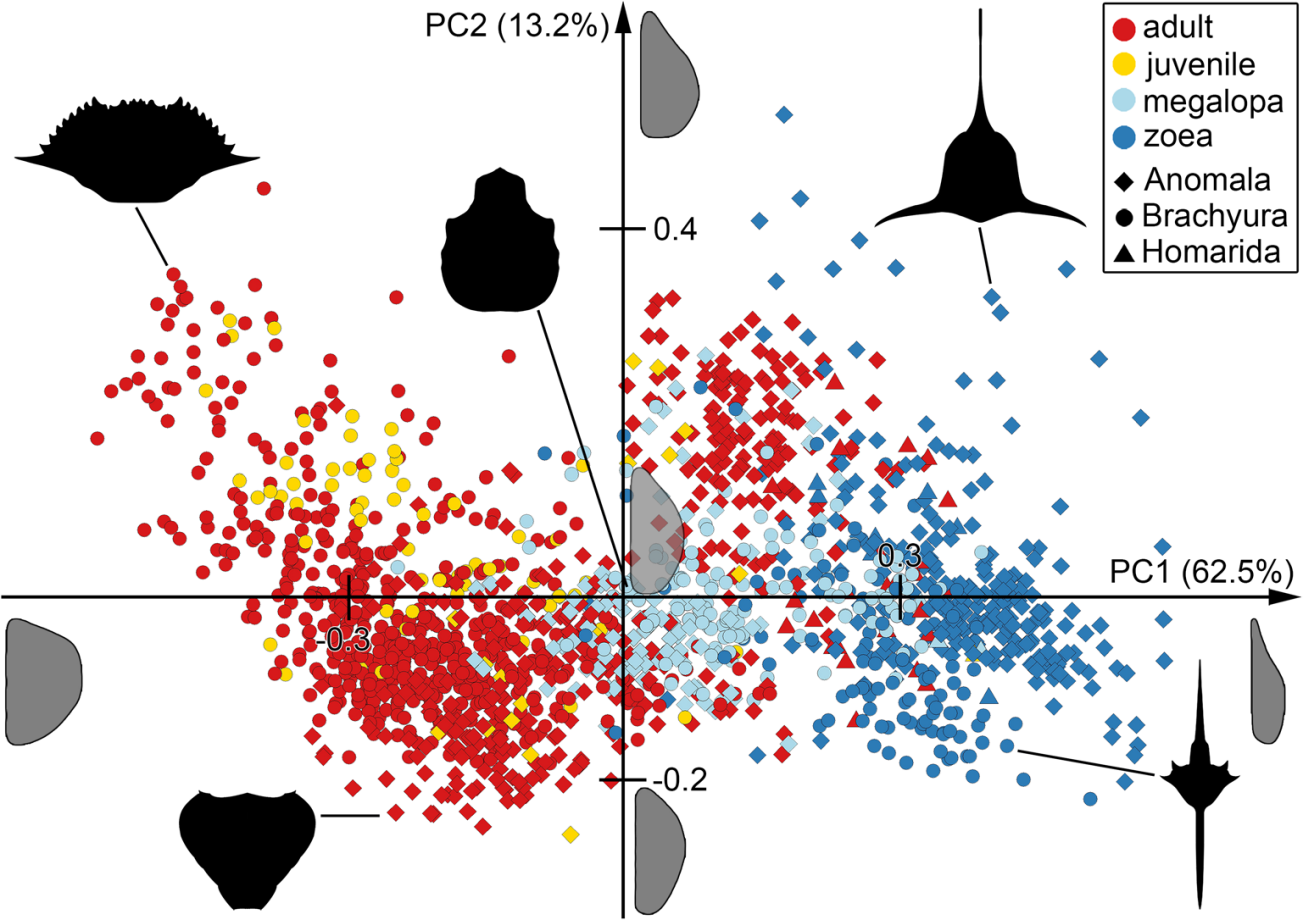
Morphospace of meiuran crabs. Principal components 1 and 2 of principal component analysis on the shield outline plotted against each other. A total of 1567 shields of true and false crabs were reconstructed, including zoea, megalopa, juvenile and adult developmental phases. Gray shapes represent graphical component loadings for the axis of the mean and ± 1 standard deviation per axis. Black shapes represent individual reconstruction drawings from the data set, which have been duplicated, mirrored, and merged to resemble their source and biological shape.

The first PC is dominated by the width of the shield (Fig. 1). Positive values represent slim shields with long rostrums, negative values represent wide shields, especially in the antero-lateral region of the shield, with less pronounced rostrums (Fig. 1). The second PC is dominated by the position of the widest point of the shield (Fig. 1). Positive values represent posteriorly widened shields, negative values represent anteriorly widened shields (Fig. 1). Graphical component loadings for all 17 PCs are depicted in Supplementary Fig. S1.

Within the morphospace, the group of adult true crabs plots in an ellipse from the bottom left off the center of the morphospace towards the top left, representing their wide shields. Adults of false crabs also plot at the bottom left off center of the morphospace, but extend in an ellipse towards the top right, showing variation with shields that are slimmer and anteriorly widening. Juveniles of both groups plot within the respective point clouds of their adult counterparts. Megalopae of both groups are mostly overlapping. They plot in a tight circle around the center of the morphospace, spreading out slightly towards the right. This position indicates a median morphology, with posteriorly widened shields and small rostrums. The zoeae of both groups plot on the right side of the morphospace, indicating their slimmer shields. True crab zoeae mostly plot on the bottom of the right side of the morphospace, indicating prominent posterior spines. False crab zoeae plot from the bottom right towards the top center of the morphospace in a spread-out cloud, indicating larger diversity in terms of spines.

### Patterns of discreteness

**Figure 2:**
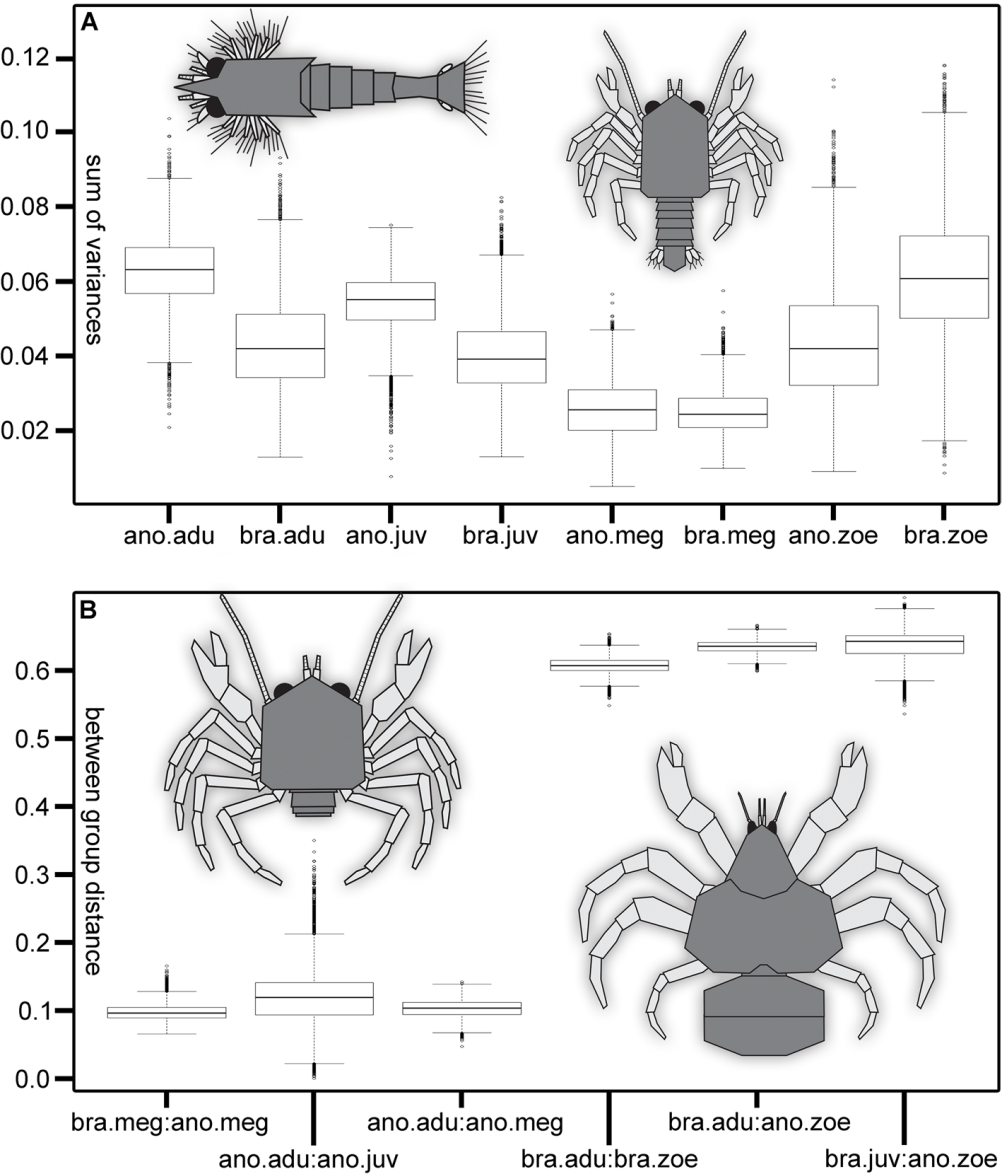
Boxplots of diversity metrics, quantifying the morphospace. Groups are created by pooling all representatives of one developmental phase (zoea, megalopa, juvenile or adult) of one of the two phylogenetic ingroups of Meiura (Anomala or Brachyura) together. (**A**) Boxplot of the bootstrapped values for the sum of variances across all dimensions for the developmental phase of Anomala and Brachyura respectively. Values are corrected for sample size by rarefaction. Reconstruction drawing on the left represents a schematic meiuran zoea larva, on the right a schematic meiuran megalopa larva. (**B**) Boxplot of the Euclidean distances between the group centroids of the developmental phases for Anomala and Brachyura respectively. Depicted are only the three smallest and three largest distances between group centroids. All values are bootstrapped. Reconstruction drawing on the left represents a schematic meiuran adult crab, on the right a schematic robber crab (*Birgus latro*). *adu* Adult, *ano* Anomala, *bra* Brachyura, *juv* Juvenile, *meg* Megalopa, *zoe* Zoea.

We compared the occupied area within the morphospace of the four developmental phases (zoea, megalopa, juvenile, adult) for true and false crabs respectively as a measure for morphological diversity. Using the sum of variances across all PCs as metric, we found that almost all groups occupied significantly different-sized areas within the morphospace (pairwise comparison of groups, bootstrapped and corrected for differences in sample size, Welch’s two-sided t-test, Bonferroni corrected for multiple testing, all *p* values < 0.001). One exception was the comparison of adults of true crabs and zoeae of false crabs, which occupied similarly sized areas within the morphospace (pairwise comparison of groups, bootstrapped and corrected for differences in sample size, Welch’s two-sided t-test, Bonferroni corrected for multiple testing, *p* value > 0.001). We found that adults of false crabs showed the largest morphological diversity (Table 1, Fig. 2A). Zoea larvae of true crabs showed similarly large morphological diversity. The other developmental phases of both true and false crabs showed smaller morphological diversities. Hereby, megalopae of both groups showed the smallest morphological diversities (Table 1, Fig. 2A).

**Table 1:** Numeric values for metrics measuring different aspects of the morphospace.

Group	Dev. phase	Sum var	Avg. disp.
Anomala	Zoea	0.042	2.878
Anomala	Megalopa	0.025	1.428
Anomala	Juvenile	0.055	1.168
Anomala	Adult	0.063	1.063
Brachyura	Zoea	0.061	2.580
Brachyura	Megalopa	0.024	1.626
Brachyura	Juvenile	0.039	1.919
Brachyura	Adult	0.042	1.858

*avg. disp.* Average displacements metric, *dev. phase* Developmental phase, *sum var.* Sum of variances metric.

We also looked at the positions of the same eight groups within the morphospace. Testing the average displacement of individuals from their group centroid, we found that again all groups showed significantly different occupations of the morphospace (pairwise comparison of groups, bootstrapped and corrected for differences in sample size, Welch’s two-sided t-test, Bonferroni corrected for multiple testing, all *p* values < 0.001). Hereby, the Euclidean distance between the centroids of the groups of megalopae of true and false crab was smallest (Fig. 2B). Meanwhile, the distance was largest between adults of true crabs and zoeae of false crabs.

**Figure 3:**
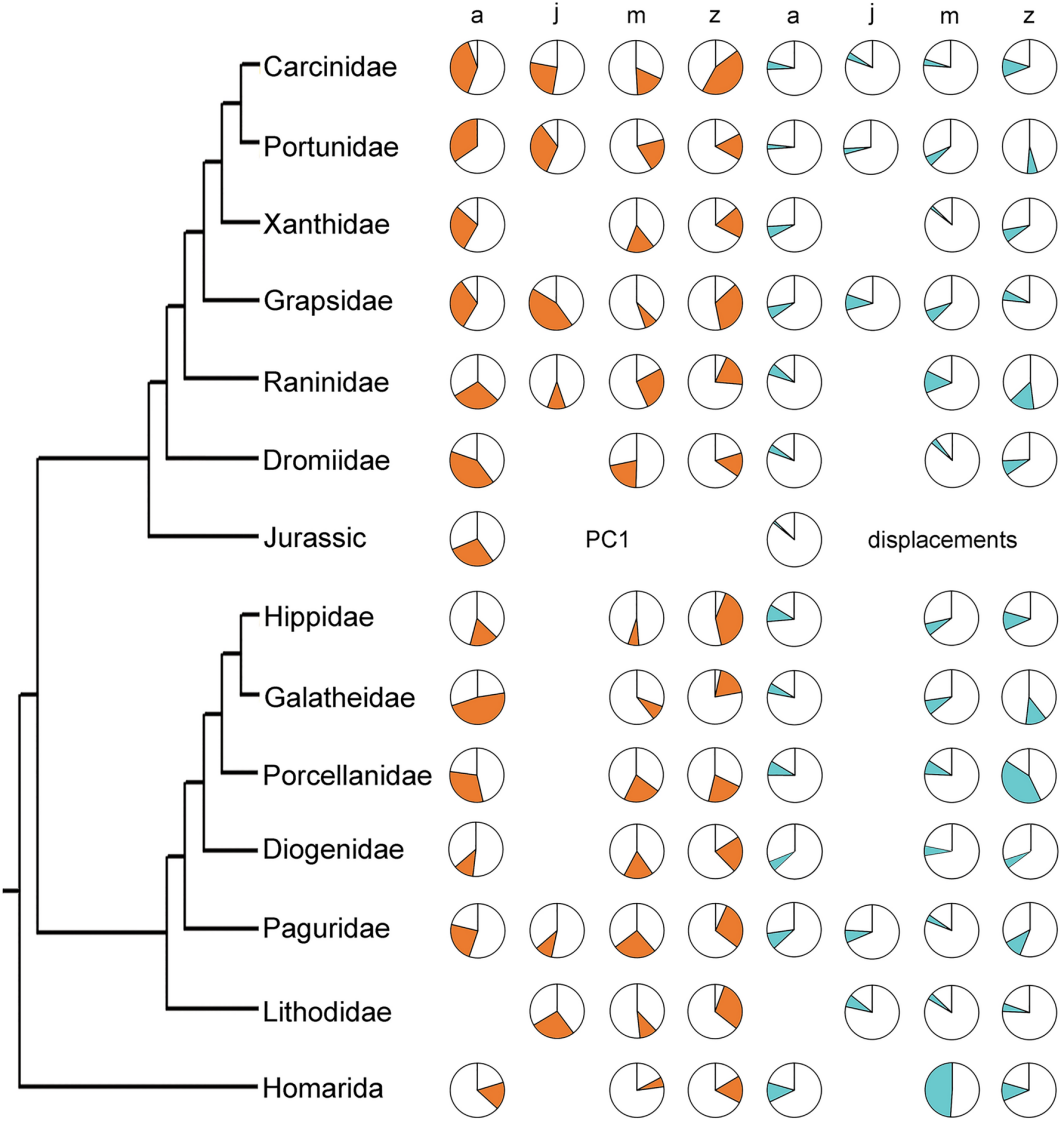
Phylogenetic tree of Meiura and outgroup Homarida after Karasawa et al.^42^, Bracken-Grissom et al.^43^ and Tsang et al.^44^. Orange-filled pie charts represent the proportion out of the total range covered of PC1 for each ingroup respectively. Clockwise from positive to negative values. Cyan-filled pie charts represent 25–75% quantiles of sample size corrected and bootstrapped average displacements, relative to the maximum average displacements for each ingroup respectively. Clockwise from high to small displacement from morphospace center. *a* Adult, *j* Juvenile, *m* Megalopa, *z* Zoea.

Lastly, we quantified the change of morphology during development for the largest ingroups of true and false crabs, respectively (Fig. 3). We calculated the average displacements for every group, additionally comparing occupation ranges for PC1 of the morphospace. Herein, adults of true crabs showed a trend of gradually occupying more negative values for PC1 further up (graphically speaking) in the phylogenetic tree (Fig. 3). The juveniles of true crabs followed this trend. However, adults and juveniles of true crabs did not show increasing distance to the center of the morphospace (average displacements). Zoeae of true crabs showed no visible trend, but a larger range of values both for PC1 and average displacements. Adults of false crabs showed a trend of acquiring slightly more positive values for PC1 going up the phylogenetic tree (again graphically speaking; Fig. 3). They showed decreasing values for the average displacements, marking smaller distances to the center of the morphospace going up the phylogenetic tree. The zoeae of false crabs, like the zoeae of true crabs, did not show a phylogenetic trend. Megalopae of both groups showed small ranges for PC1 and small distances to the center of the morphospace.

## Discussion

### Limitations of the study

The present analysis is a first look into the morphological diversity in Meiura and Decapoda. We plan to build on this data set in the future, including more groups and specimens, to enable the investigation of further topics.

Here, we could not include juveniles, i.e., crab 1 and crab 2 stages, of every ingroup in our analysis due to a lack of material available in the literature. This aspect was generally the largest limitation of our analysis, as collecting enough material for an ingroup, especially of larval stages, proved to be difficult. This lack highlights the importance of publishing morphological data and the important role open databases and collections play for large-scale quantitative analyses and the understanding of biodiversity.

We disregarded asymmetry in the shield for the sake of improved alignment of shapes. Due to the size of our data set and the total covered variation, we expect biological asymmetry to play a minor role in morphological diversity, compared to e.g., the difference between developmental phases. Disregarding asymmetry allowed us to use only one half of the shield, with which a more precise alignment of the shapes could be reached.

We did not possess a robust phylogeny for the comparison of change through the ingroups of true and false crabs (Fig. 3). We rather combined and abbreviated trees after Karasawa et al.^26^, Bracken-Grissom et al.^27^ and Tsang et al.^28^ to construct a dichotomous phylogeny including all relevant ingroups.

Our sample sizes for representatives of true and false crabs are balanced and therefore not proportionate to species numbers of the respective group, i.e. we sampled relatively more false crabs than true crabs. We decided to balance sample sizes instead of sample proportion to reduce artifacts in the statistical analysis. Furthermore, the sample size of over 700 representatives across each group seemed appropriate to us to represent the diversity of each group.

### True versus false crab morphological diversity

When comparing the adult life phase, morphologies of true and false crabs were significantly different. Furthermore, adults of false crabs expressed larger morphological diversity than adults of true crabs (falsifying Hypothesis 3). Some unique forms of false crabs were shapes that were slimmer than in true crabs and were widening posteriorly, not anteriorly (Fig. 1). The difference in diversity between true and false crabs stands in line with some observations from literature^12,13,29^. False crabs represent some of the more charismatic ingroups of Decapoda, with some adult hermit crabs having specialized morphologies adapted to hiding in shells^30^, or the adult robber crab adapted to climb trees^31^. True crabs seemingly lack such extreme forms, which might be one reason for the smaller morphological diversity of their adults. Furthermore, full carcinization seems to have happened three times independently within false crabs^8,13^, creating further morphological disparity, e.g., between hermit crabs and king crabs, which are closely related^32^. Habitat diversity (and consecutive adaptations) could be another driver of morphological diversity in false crabs. But contradicting this explanation is the fact that false crabs have conquered terrestrial habitats more recently and on fewer occasions than true crabs^33,34^. Generally, true and false crabs have conquered similar habitats around the globe, and representatives from marine and terrestrial habitats, but not from freshwater, are present for both groups in our analysis. Carcinization could also be used to argue for the difference in morphological diversity. As the crab body shape has been brought up as an evolutionary advantageous morphology that can adapt to many habitats^1^, false crabs that are not (fully) carcinized, i.e. fulfilling the characters of a crab sensu Scholtz (depressed shield with a lateral margin, wide sternum, ventrally flexed pleon)^12^, may have had to develop a larger range of morphologies to adapt to the same environmental conditions. Simply put, the crab body shape may be a “one fits all” solution, which was not available to not fully-carcinized false crabs. The difference in morphological diversity between true and false crabs also demonstrates that taxonomic diversity and morphological diversity (and therefore ecological diversity)^35^ are not necessarily linked^36–38^. After all, the taxonomic diversity of true crabs exceeds that of false crabs, more than two-fold^10,11^.

Morphological diversity of the zoea phase was likewise not equal between true and false crabs (falsifying H1). Instead, the morphologies were significantly different, with zoeae of true crabs expressing larger morphological diversity than zoeae of false crabs (Table 1, Fig. 2A). One example for this difference are unique forms in zoeae of true crabs such as larvae with a rostrum protruding anteriorly, a posterior spine protruding posteriorly, and an additional pair of lateral spines protruding laterally away from the body (Fig. 1). The morphological disparity between true and false crab zoeae is interesting considering the similar selective pressures of the pelagic environment and generally assumed low interspecific variation within each group^15,23,24^. Again, habitat adaptations may be a reason for increased morphological adaptations (such as adaptations to different water depths). Another potential reason may be the duration of the zoea phase. False crabs usually have four to five zoea stages (although there are indications for longer sequences, e.g. giant larvae)^39^, while true crabs can have up to twelve zoea stages^15^. Going through more zoea stages usually takes more time and therefore equals a longer zoea phase. This longer time period may provide potential for additional adaptations or even require these, especially since it also leads to a larger body size, again requiring new morphological adaptations to deal with buoyancy and predator avoidance^15,40^.

### The megalopa is a plesiomorphic and potentially phylotypic stage

The megalopa phases of both groups showed comparatively small morphological diversity (Fig. 1, Table 1), and both occupied a similar area within the morphospace, indicating morphological similarity with each other (partial support for H1; Fig. 2B, Table 1). The center position they occupy within the morphospace is also close to that of zoea and adult representatives of Homarida and the earliest adult representatives of Brachyura (Fig. 1; partial support for H1). Homarida represents our outgroup and an ancestral morphology, as the representatives of Homarida are lobster-like, which is the supposed ancestral morphology for Meiura^12,41^. Meanwhile, the earliest adult representatives of true crabs are represented by fossils from the Jurassic, including the so far oldest ones^42,43^. Together, these observations suggest, that the megalopa morphology is plesiomorphic for the group Meiura. A possible explanation for this observation may be common selective pressures. The megalopa phase must mediate a transition from the planktic to the benthic lifestyle of the animal^15^. This change is accompanied by a morphological transition from a slim spiny larva to a bulky crab. These two tasks may constrain the morphology of the megalopa, not allowing strong deviation from the ancestral state.

Furthermore, the megalopa phase in Meiura also appears to be less morphologically diverse than the preceding zoea phase and the following adult phase (Table 1). This is generally an indication for a phylotypic stage. The phylotypic stage, meaning a stage in which all representatives of a group show the largest degree of similarity^44^, has been mostly discussed in vertebrates^44,45^. Yet, it also has been discussed for insects^46,47^, and (other) crustaceans^48^. Since mutations are selected against in phylotypic stages, this could provide another explanation for the plesiomorphic appearance^49^. However, the megalopa stage is reached late during the development, while a phylotypic stage has been suggested to occur early during organogenesis^49^. Yet, this may reflect a vertebrate-centered view, as in many crustaceans specific organs become differentiated only later in ontogeny. Investigating the spatial gene expression in megalopae of true and false crabs could therefore provide additional information on the hypothesis of the megalopa as a phylotypic stage as well as the reason for the strong conservation of phylotypic stages^44,49^.

### Patterns of divergence in the evolution of Meiura

Carcinization is also often discussed in the context of convergent evolution^9^. True crabs as well as ingroups of false crabs (Porcellanidae, Lithodidae, Lomisidae) have convergently evolved a crab-like habitus via carcinization^9,13^. In our analysis, we can find partial support for this pattern. Representatives of Porcellanidae plot close to the center of the morphospace, overlapping with some true crabs, those of Lithodidae however plot mostly on the far right of the morphospace (Supplementary Figure S4). Representatives of the more derived ingroups of true crabs also plot further to the top left of the morphospace, diverging from both the center of the morphospace and false crabs. This pattern could potentially be caused by true crab forms showing increasingly wider than long forms in more derived ingroups, especially with long lateral spines, while some representatives of false crabs have long bodies. The present analysis does therefore not provide further support for convergent evolution within the body shape of crabs.

Interestingly, adults of true crabs showed smaller morphological diversity than zoea larvae of true crabs (partially falsifying H2). Meanwhile, adults of false crabs expressed larger morphological diversity than their zoea larvae (partial support for H2; Table 1). This pattern indicates that different ends of the developmental pathway show increases in diversity for true and false crabs. In false crabs, the late phase of (post-embryonic) development shows increased diversity, while in true crabs the early phase of (post-embryonic) development shows increased diversity, forming a pattern of divergence (Fig. 2A). However, this divergent pattern could simply be an artifact of the earlier described diversity differences between true and false crabs. In any case, these results highlight the importance of larvae for biodiversity studies, which are often still overlooked^38,40^.

Focusing on the adults, if we look at the change of morphology throughout the phylogeny of the groups, somewhat reconstructing the evolutionary history, we find another pattern of divergence. With increasing phylogenetic distance, true and false crabs occupy more distant regions in the morphospace (Fig. 3). While morphological disparity between the more closely related ingroups of true and false crabs seems smaller, the disparity increases between the more derived ingroups. This pattern indicates diverging evolution in the shield morphology of Meiura, despite the process of carcinization. Shared habitats between true and false crabs may cause competition that has driven adaptation in opposing directions. Another reason might be that true crab shields are becoming gradually broader over time, while in false crabs they become longer. So far, we can only speculate as to why this pattern arises.

## Conclusion

The morphology of the megalopa in true and false crabs suggests that it is plesiomorphic and potentially phylotypic for the group. Together with the larval diversity found in the zoea phase, these findings highlight the importance of larval information in biological research. The larger morphological diversity in adult false crabs, despite true crabs having a larger species count, highlights the importance of different metrics for measuring biodiversity. Lastly, although carcinization is often brought up as an example of convergent evolution in crabs, the here presented results also demonstrate some patterns of divergence for the group Meiura.

## STAR methods

**Table:** Key resource table

REAGENT or RESOURCE	SOURCE	IDENTIFIER
Deposited data		
Shapes of specimens	This study	10.5281/zenodo.10125461
Software and algorithms		
R software-environment ver. 4.1.2	R Core Team 2021	RRID:SCR_001905; https://cran.r-project.org/
Custom R scripts for analysis and statistical tests	This study	https://github.com/rianbreak/AnomalaVsBrachyura
Adobe Illustrator CS2	Adobe	RRID:SCR_010279; http://www.adobe.com/products/illustrator.html
Inkscape	The Inkscape project	RRID:SCR_014479; https://inkscape.org/en/
Photoshop CS2	Adobe	RRID:SCR_014199; https://www.adobe.com/products/photoshop.html

### Material

Material used for this study originated from published images and reconstruction drawings in literature, open databases, collections, and museums. Some of the material was documented and published by the authors (for documentation methods, see Eiler et al.^50^)^40,51,52^. Our material included extant and fossil species of Meiura. For a detailed overview on data origin, see Supplementary Table S2, for the list of respective literature references, see Supplementary References S3. From these sources the outline of the shield in dorsal view was reconstructed for each of the 1567 specimens (identical to earlier studies)^45,53^. Source material viable for reconstruction had to meet certain criteria, such as: complete specimen; documented in dorsal view without large inclination; good lighting in the case of images. We sorted each specimen into a phylogenetic category (Anomala or false crab; Brachyura or true crab) and an ontogenetic category (zoea, megalopa, juvenile, adult).

### Sampling

When gathering material for this analysis, we did not sample every major ingroup (usually ranked as “superfamilies”) of true and false crabs. Instead, we picked major ingroups based on available material, species numbers, and phylogenetic distance, to create a broad sampling pattern across Brachyura and Anomala. These groups were Carcinidae, Dromiidae, Grapsidae, fossil representatives from the Jurassic, Portunidae, Raninidae, and Xanthoidea for true crabs and Diogenidae, Galatheidae, Hippoidea, Lithodidae, Paguroidea, and Porcellanidae for false crabs. We are aware that there are still some major groups missing from this analysis, like Majoidea or Leucosioidea (both ingroups of Brachyura). However, we refrained from including more specimens to keep the balanced sample sizes between true and false crabs.

We also balanced sample sizes of included specimens for true and false crabs instead of proportionately sampling specimens. We decided for balanced sample sizes to reduce artifacts in the statistical analysis created by sample size correction methods. Also, including over 700 specimens per group over a large phylogenetic distance seemed to represent a large share of variation in both groups.

### Data generation

We used vector graphic software, *Adobe Illustrator CS2* and free and open software *InkScape*, for the reconstruction of shield outlines. Each shield outline was manually traced in one of the two programmes. To prevent variation in the data due to left–right asymmetry, we only reconstructed the left or the right half of the shield and, where necessary, mirrored it to always end up with a right half. Asymmetry in Meiura exists for various reasons^54^. However, we chose to disregard it in favour of improved alignment to improve our analysis. We expect the impact of asymmetry to be minor on the analysis, due to the size of the data set and the overall scale of morphological variation.

### Quantification and statistical analysis

All analyses were performed offline, using custom scripts in the R-statistics environment (ver. 4.1.039)^38^. The shape of the shield was quantified using elliptic Fourier analysis (EFA), which applies the principle of the Fourier transformation to translate the two-dimensional outline into a mathematical object^38,55,56^. The shield shape is decomposed into a harmonic sum of trigonometric functions, weighted with harmonic coefficients^55,56^. These harmonic coefficients are then aligned according to a homologous starting point^55,56^. We aligned and centered the shield shapes according to the center left point of the shape, i.e., halfway along the straight line where the shield was halved. We then scaled the outlines based on centroid distance to reduce any artefacts stemming from scale differences in the source material. We used a calibration function of the *Momocs* package on our whole data set to find an appropriate number of harmonics to describe the shapes. The results yielded 11 harmonics representing 99% of variation in the data, which we opted for. The results were then analyzed with a principal component analysis (PCA), choosing to retain the first 17 PCs, because they amounted to more than 99% of variation in the data set (PC1 = 62.5%; PC2 = 13.2%; PC3 = 10.1%; PC4 = 4.9%; PC5 = 2.5%; PC6 = 1.5%; PC7 = 1.1%; PC8 = 0.9%; PC9 = 0.7%; PC10 = 0.6%; PC11 = 0.4%; PC12 = 0.4%; PC13 = 0.3%; PC14 = 0.2%; PC15 = 0.2%; PC16 = 0.2%; PC17 = 0.1%). We chose the 99% of variation criterion over other methods to choose a cut-off value, because the scree-plot is subjective, and the computational approach was not feasible with our data^57^. The matrix of principal components (PCs) was then used as input data for further statistical analysis and graphical interpretation.

For initial visualization of the morphospace, PC1 and PC2 were plotted against each other in scatterplots, using *ggplot2* (ver. 3.3.542), adding biological shapes manually in *Photoshop CS2* for easier interpretation.

We then calculated different metrics to measure morphological diversity using the package *dispRity* (ver. 1.6.043). For this approach, we first bootstrapped each data set 10,000 times and applied rare-faction-based correction for differences in sample sizes, using the sample size of the smallest group of each comparison. We tested different metrics, following Guillerme and Cooper^58^, and chose appropriate metrics to measure morphospace occupation and position within morphospace.

We first calculated the sum of variances across all PCs for the four developmental phases of true and false crabs respectively, as a measure for morphological diversity. Meaning we grouped all representatives of one developmental phase of one phylogenetic group together, resulting in eight groups. The smallest sample size used for correction was that of juvenile false crabs with 16 specimens. In total, 1567 shield shapes were compared. We then tested the groups for significant differences, using pairwise Welch’s two-sided t-test with Bonferroni correction for multiple testing.

We then calculated the average displacement of individuals from their group centroids in relation to the center of the morphospace, again for the four developmental phases of true and false crabs respectively. This metric measures the position within the morphospace, therefore giving insight into how morphologies differ between groups. The smallest sample size used for correction was again that of juvenile false crabs with 16 specimens. In total, 1567 shield shapes were compared. We again tested the groups for significant differences, using pairwise Welch’s two-sided t-test with Bonferroni correction for multiple testing.

We furthermore used the average displacements metric as well as the occupation of PC1 of the developmental phases of every larger ingroup of true and false crabs included in our analysis as a measure for the change of morphology during development along the phylogenetic tree (included groups are: Carcinidae, Diogenidae, Dromiidae, fossil representatives of Brachyura from the Jurassic, Galatheidae, Grapsidae, Hippidae, Lithodidae, Paguridae, Porcellanidae, Portunidae, Raninidae, and Xanthidae). As we only used representatives of these groups, the sample size in this analysis is smaller (e.g., not all representatives of Hippoidea in our analysis are also representatives of Hippidae). We calculated the smallest and largest value of any specimen for PC1 and defined these two values as the possible range of occupation for PC1. We then calculated the smallest and largest value for every developmental phase for every phylogenetic ingroup respectively. We then plotted this occupied range of PC1 as a part of a pie chart. Similarly, we calculated the average displacements for every developmental phase of every phylogenetic ingroup, this time taking the 25% and 75% quantiles as lower and upper boundary for the occupied range. We then used the 75% quantile of the group with the largest value as maximum value and set the minimum to zero (as the least displacement from the center equals a value of zero), defining the possible range of average displacements. Again, we plotted the realized average displacements of each group as a part in a pie chart.

We also calculated the Euclidean distance between group centroids for all eight established groups to compare how different the morphologies of each group are. For this metric, we used bootstrapped but not sample size corrected data sets. We selected the three largest and the three smallest distances to display within the text. The number three was picked arbitrarily, based on the need to reduce the number of displayed distances in the main text.

### Supplementary Information

Supplementary Information 1.

Supplementary Information 2.

Supplementary Information 3.

Supplementary Information 4.

## Data Availability

The dataset generated and analyzed during the current study is available in the Zenodo repository, DOI: 10.5281/zenodo.10125461. All data reported in this paper will also be shared by the lead contact upon request. Florian Braig (florian.braig@palaeo-evo-devo.info).

## References

[1] Förster R (1985). Evolutionary trends and ecology of Mesozoic decapod crustaceans. Earth Environ. Sci. Trans. R. Soc. Edinb..

[2] Schram FR (1986). Crustacea.

[3] Taylor RS, Schram FR, Savazzi E (1999). Meiura (anomalan and brachyuran crabs). Functional Morphology of the Invertebrate Skeleton.

[4] Rao PV, Thomas MM, Rao GS (1973). The crab fishery resources of India. Proc. Symp. Living Res. Seas India.

[5] Stevens BG, Miller TJ, Lovrich G, Thiel M (2020). Crab fisheries. Fisheries and Aquaculture.

[6] Smith TJ, Boto KG, Frusher SD, Giddins RL (1991). Keystone species and mangrove forest dynamics: The influence of burrowing by crabs on soil nutrient status and forest productivity. Estuar. Coast. Shelf Sci..

[7] Citadin M, Costa TM, Netto SA (2018). Response of estuarine meiofauna communities to shifts in spatial distribution of keystone species: An experimental approach. Estuar. Coast. Shelf Sci..

[8] Borradaile LA (1916). Crustacea. Part II—*Porcellanopagurus*: An instance of Carcinization British Antarctic (“Terra Nova”) expediton, 1910. Nat. Hist. Rep..

[9] Wolfe JM, Luque J, Bracken-Grissom HD (2021). How to become a crab: Phenotypic constraints on a recurring body plan. BioEssays.

[10] De Grave S, Decock W, Dekeyzer S, Davie PJ, Fransen CH, Boyko CB, Poore GCB, Macpherson E, Ahyong ST, Crandall KA, Santos S (2023). Benchmarking global biodiversity of decapod crustaceans (Crustacea: Decapoda). J. Crust. Biol..

[11] WoRMS Editorial Board World Register of Marine Species. https://www.marinespecies.org (2023). 10.14284/170

[12] Scholtz G (2014). Evolution of crabs–history and deconstruction of a prime example of convergence. Contrib. Zool..

[13] Keiler J, Wirkner CS, Richter S (2017). One hundred years of carcinization—The evolution of the crab-like habitus in Anomura (Arthropoda: Crustacea). Biol. J. Linn. Soc..

[14] Martin JW, Olesen J, Høeg JT (2014). Atlas of Crustacean Larvae.

[15] Anger K (2001). The Biology of Decapod Crustacean Larvae.

[16] Rice AL (1981). The megalopa stage in brachyuran crabs. The Podotremata Guinot. J. Nat. Hist..

[17] Møller OS, Ira K, Guerao G, Anger K, Harzsch S, Thiel M (2020). Patterns of larval development in developmental biology and larval ecology. Developmental Biology and Larval Ecology: The Natural History of the Crustacea.

[18] Williamson DI (1969). Names of larvae in the Decapoda and Euphausiacea. Crustaceana.

[19] Haug JT, Anger K, Harzsch S, Thiel M (2020). Metamorphosis in Crustaceans. Developmental Biology and Larval Ecology: The Natural History of the Crustacea.

[20] Maisey JG, de Carvalho MDGP (1995). First records of fossil sergestid decapods and fossil brachyuran crab larvae (Arthropoda, Crustacea), with remarks on some supposed palaemonid fossils, from the Santana Formation (Aptian-Albian, NE Brazil). American Mus. Novit.

[21] Haug JT, Martin JW, Haug C (2015). A 150-million-year-old crab larva and its implications for the early rise of brachyuran crabs. Nat. Commun..

[22] Spiridonov VA (2020). An update of phylogenetic reconstructions, classification and morphological characters of extant Portunoidea Rafinesque, 1815 (Decapoda, Brachyura, Heterotremata), with a discussion of their relevance to fossil material. Geologija.

[23] Høeg JT, Møller OS (2006). When similar beginnings lead to different ends: Constraints and diversity in cirripede larval development. Invert. Reprod. Devel..

[24] Rice AL (1980). Crab zoeal morphology and its bearing on the classification of the Brachyura. Trans. Zool. Soc. London.

[25] Purvis A, Hector A (2000). Getting the measure of biodiversity. Nature.

[26] Karasawa H, Schweitzer CE, Feldmann RM (2011). Phylogenetic analysis and revised classification of podotrematous Brachyura (Decapoda) including extinct and extant families. J. Crust. Biol..

[27] Bracken-Grissom HD, Cannon ME, Cabezas P, Feldmann RM, Schweitzer CE, Ahyong ST, Felder DL, Lemaitre R, Crandall KA (2013). A comprehensive and integrative reconstruction of evolutionary history for Anomura (Crustacea: Decapoda). BMC Evol. Biol..

[28] Tsang LM, Schubart CD, Ahyong ST, Lai JC, Au EY, Chan TY, Ng PKL, Chu KH (2014). Evolutionary history of true crabs (Crustacea: Decapoda: Brachyura) and the origin of freshwater crabs. Mol. Biol. Evol..

[29] Tsang LM, Chan TY, Ahyong ST, Chu KH (2011). Hermit to king, or hermit to all: Multiple transitions to crab-like forms from hermit crab ancestors. Syst. Biol..

[30] Williams JD, McDermott JJ (2004). Hermit crab biocoenoses: A worldwide review of the diversity and natural history of hermit crab associates. J. Exp. Mar. Biol. Ecol..

[31] Horst R (1902). On the habits of the Cocoa-nut Crab or Palm thief. Notes Leyden Mus..

[32] Hall S, Thatje S (2009). Global bottlenecks in the distribution of marine Crustacea: Temperature constraints in the family Lithodidae. J. Biogeogra..

[33] Wolfe JM, Ballou L, Luque J, Watson-Zink VM, Ahyong ST, Barido-Sottani J, Chan T-Y, Chu KH, Crandall KA, Daniels SR, Felder DA, Mancke H, Martin JW, Ng PKL, Ortega-Hernández J, Theil EP, Pentcheff ND, Robles R, Thoma BP, Tsang LM, Wetzer R, Windsor AM, Bracken-Grissom HD (2022). Convergent adaptation of true crabs (Decapoda: Brachyura) to a gradient of terrestrial environments. BioRxiv.

[34] Greenaway P (2003). Terrestrial adaptations in the Anomura (Crustacea: Decapoda). Memo. Mus. Victoria.

[35] Ricklefs RE, Miles DB, Wainwright PC, Reilly SM (1994). Ecological and evolutionary inferences from morphology: An ecological perspective. Ecological Morphology: Integrative Organismal Biology.

[36] Foote M (1993). Discordance and concordance between morphological and taxonomic diversity. Paleobiology.

[37] Triantis KA, Rigal F, Parent CE, Cameron RA, Lenzner B, Parmakelis A, Yeung NW, Alonso MR, Ibáñez M, de Frias Martins A, Cowie RH (2016). Discordance between morphological and taxonomic diversity: Land snails of oceanic archipelagos. J. Biogeogr..

[38] Haug C, Braig F, Haug JT (2023). Quantitative analysis of lacewing larvae over more than 100 million years reveals a complex pattern of loss of morphological diversity. Sci. Rep..

[39] Gundi P, Cecchin C, Fetzer LL, Haug C, Melzer RR, Haug JT (2020). Giant planktic larvae of anomalan crustaceans and their unusual compound eyes. Helgoland Mar. Res..

[40] Braig F, Haug C, Haug JT (2023). Phenotypic variability in the shield morphology of wild- vs. lab-reared eumalacostracan larvae. Nauplius.

[41] Glaessner MF, Glaessner MF (1969). Decapoda. Treatise on Invertebrate Paleontology, Part R, Arthropoda.

[42] Haug JT, Haug C (2014). *Eoprosopon klugi* (Brachyura)—The oldest unequivocal and most “primitive” crab reconsidered. Palaeodiversity.

[43] Scholtz G (2020). *Eocarcinus praecursor* Withers, 1932 (Malacostraca, Decapoda, Meiura) is a stem group brachyuran. Arth. Struc. Dev..

[44] Slack JM, Holland PW, Graham CF (1993). The zootype and the phylotypic stage. Nature.

[45] Richardson MK, Minelli A, Coates M, Hanken J (1998). Phylotypic stage theory. Trends Ecol. Evol..

[46] Sander K (1976). Specification of the basic body pattern in insect embryogenesis. Adv. Insect. Physiol..

[47] Švorcová J (2012). The phylotypic stage as a boundary of modular memory: Non mechanistic perspective. Theory Biosci..

[48] Williams TA (1994). The nauplius larva of crustaceans: Functional diversity and the phylotypic stage. Am. Zool..

[49] Galis F, Hansen TF, Houle D, Pavličev M, Pélabon C (2023). Evolvability of body plans: On phylotypic stages, developmental modularity, and an ancient metazoan constraint. Evolvability: A Unifying Concept in Evolutionary Biology?.

[50] Eiler SM, Haug C, Haug JT (2016). Detailed description of a giant polychelidan eryoneicus-type larva with modern imaging techniques (Eucrustacea, Decapoda, Polychelida). Spixiana.

[51] Rudolf NR, Haug C, Haug JT (2016). Functional morphology of giant mole crab larvae: A possible case of defensive enrollment. Zool. Lett..

[52] Braig F, Posada Zuluaga V, Haug C, Haug JT (2021). Diversity of hippoidean crabs-considering ontogeny, quantifiable morphology, and phenotypic plasticity. Nauplius.

[53] Haug C, Posada Zuluaga V, Zippel A, Braig F, Müller P, Gröhn C, Weiterschan T, Wunderlich J, Haug GT, Haug JT (2022). The morphological diversity of antlion larvae and their closest relatives over 100 million years. Insects.

[54] Spani F, Scalici M (2018). Carapace asymmetries in crabs. Crustaceana.

[55] Bonhomme V, Picq S, Gaucherel C, Claude JM (2014). Outline analysis—Using R. J. Stat. Soft..

[56] Braig F, Haug C, Haug JT (2023). Diversification events of the shield morphology in shore crabs and their relatives through development and time. Paleontologia Electronica.

[57] Joliffe IT, Joliffe IT (2002). Choosing a subset of principal components or variables. Principal Component Analysis. Springer Series in Statistics.

[58] Guillerme, T. & Cooper, N. dispRity manual. figshare. Preprint. 10.6084/m9.figshare.6187337.v1 (2018).

