# Supplementary figures and images for: Morphological diversity in true and false crabs reveals the plesiomorphy of the megalopa phase
Source: Sci Rep. 2024 Apr 15;14:8682. doi: 10.1038/s41598-024-58780-7 (PMC11018780; doi:10.1038/s41598-024-58780-7)

**Supplementary Figure S1:** Graphical component loadings of first 17 PCs of PCA on shield outlines.

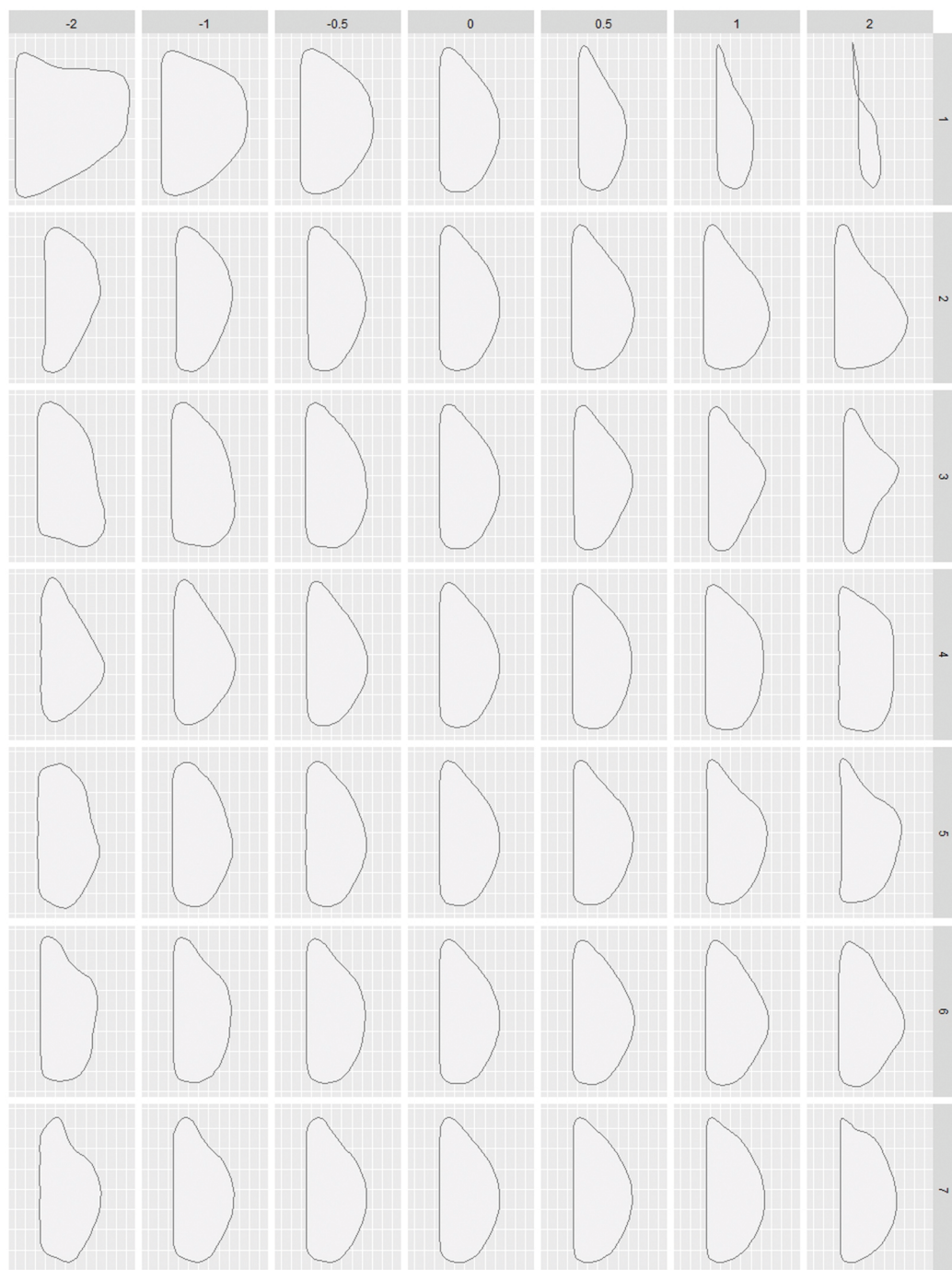

Supplement: Supplementary file 1 — Supplementary Information 1. [file 41598_2024_58780_MOESM1_ESM.pdf]
