# Supplementary material for: Morphological diversity in true and false crabs reveals the plesiomorphy of the megalopa phase
Source: Sci Rep. 2024 Apr 15;14:8682. doi: 10.1038/s41598-024-58780-7 (PMC11018780; doi:10.1038/s41598-024-58780-7)
Supplement: Supplementary file 2 — Supplementary Information 2. [file 41598_2024_58780_MOESM2_ESM.pdf]

Supplementary Table S2: List of material used in the study.

no	general_group	major_out	major_group	species_group	species	developmental_phase	status
CaDi_002	Brachyura	Carcinidae	Carcininae	*Carcinus*	*aestuarii*	adult	extant
CaDi_003	Brachyura	Carcinidae	Parathranitiinae	*Parathranites*	*intermedius*	adult	extant
CaDi_004	Brachyura	Carcinidae	Parathranitiinae	*Parathranites*	*hexagonus*	adult	extant
CaDi_005	Brachyura	Carcinidae	Parathranitiinae	*Parathranites*	*ponens*	adult	extant
CaDi_006	Brachyura	Carcinidae	Parathranitiinae	*Parathranites*	*tuberosus*	adult	extant
CaDi_007	Brachyura	Carcinidae	Pirimelinae	*Pirimela*	*denticulata*	adult	extant
CaDi_008	Brachyura	Carcinidae	Pirimelinae	*Pirimela*	*denticulata*	adult	extant

no	general_group	major_out	major_group	species_group	species	developmental_phase	status
CaDi_009	Brachyura	Carcinidae	Pirimelinae	*Pirimela*	*denticulata*	adult	extant
CaDi_010	Brachyura	Carcinidae	Platyonichinae	*Portumnus*	*latipes*	adult	extant
CaDi_011	Brachyura	Carcinidae	Platyonichinae	*Portumnus*	*latipes*	adult	extant
CaDi_012	Brachyura	Carcinidae	Platyonichinae	*Portumnus*	*latipes*	adult	extant
CaDi_013	Brachyura	Carcinidae	Platyonichinae	*Portumnus*	*latipes*	adult	extant
CaDi_014	Brachyura	Carcinidae	Platyonichinae	*Portumnus*	*lysianassa*	adult	extant
CaDi_015	Brachyura	Carcinidae	Platyonichinae	*Portumnus*	*lysianassa*	adult	extant

no	general_group	major_out	major_group	species_group	species	developmental_phase	status
CaDi_016	Brachyura	Carcinidae	Polybiinae	*Olicarcinus*	*trevisani*	adult	fossil
CaDi_017	Brachyura	Carcinidae	Carcininae	*Carcinus*	*aestuarii*	adult	extant
CaDi_018	Brachyura	Carcinidae	Carcininae	*Carcinus*	*aestuarii*	adult	extant
CaDi_019	Brachyura	Carcinidae	Carcininae	*Carcinus*	*aestuarii*	adult	extant

no	general_group	major_out	major_group	species_group	species	developmental_phase	status
CaDi_020	Brachyura	Carcinidae	Carcininae	*Carcinus*	*aestuarii*	adult	extant
CaDi_021	Brachyura	Carcinidae	Carcininae	*Carcinus*	*aestuarii*	adult	extant
CaDi_022	Brachyura	Carcinidae	Parathranitiinae	*Parathranites*	*orientalis*	adult	extant
CaDi_023	Brachyura	Carcinidae	Parathranitiinae	*Parathranites*	*orientalis*	adult	extant
CaDi_024	Brachyura	Carcinidae	Parathranitiinae	*Parathranites*	*orientalis*	adult	extant
CaDi_025	Brachyura	Carcinidae	Parathranitiinae	*Parathranites*	*orientalis*	adult	extant

no	general_group	major_out	major_group	species_group	species	developmental_phase	status
CaDi_026	Brachyura	Carcinidae	Parathranitiinae	*Parathranites*	*orientalis*	adult	extant
CaDi_027	Brachyura	Carcinidae	Pirimelinae	*Pirimela*	*denticulata*	adult	extant
CaDi_028	Brachyura	Carcinidae	Pirimelinae	*Pirimela*	*denticulata*	adult	extant
CaDi_029	Brachyura	Carcinidae	Pirimelinae	*Sirpus*	*zariquieyi*	adult	extant

no	general_group	major_out	major_group	species_group	species	developmental_phase	status
CaDi_030	Brachyura	Carcinidae	Platyonichinae	*Portumnus*	*latipes*	adult	extant
CaDi_031	Brachyura	Carcinidae	Platyonichinae	*Portumnus*	*latipes*	adult	extant
CaDi_032	Brachyura	Carcinidae	Platyonichinae	*Portumnus*	*latipes*	adult	extant
CaDi_033	Brachyura	Carcinidae	Platyonichinae	*Portumnus*	*latipes*	adult	extant

no	general_group	major_out	major_group	species_group	species	developmental_phase	status
CaDi_034	Brachyura	Carcinidae	Platyonichinae	*Portumnus*	*lysianassa*	adult	extant
CaDi_035	Brachyura	Carcinidae	Platyonichinae	*Portumnus*	*lysianassa*	adult	extant
CaDi_036	Brachyura	Carcinidae	Platyonichinae	*Xaiva*	*biguttata*	adult	extant
CaDi_037	Brachyura	Carcinidae	Carcininae	*Carcinus*	*aestuarii*	megalopa	extant
CaDi_038	Brachyura	Carcinidae	Platyonichinae	*Portumnus*	*latipes*	megalopa	extant
CaDi_039	Brachyura	Carcinidae	Pirimelinae	*Pirimela*	*denticulata*	megalopa	extant
CaDi_040	Brachyura	Carcinidae	Platyonichinae	*Portumnus*	*latipes*	megalopa	extant
CaDi_041	Brachyura	Carcinidae	Platyonichinae	*Portumnus*	*latipes*	juvenile	extant
CaDi_042	Brachyura	Carcinidae	Parathranitiinae	*Parathranites*	*orientalis*	adult	extant
CaDi_043	Brachyura	Carcinidae	Parathranitiinae	*Parathranites*	*orientalis*	adult	extant
CaDi_044	Brachyura	Carcinidae	Parathranitiinae	*Parathranites*	*granosus*	adult	extant

no	general_group	major_out	major_group	species_group	species	developmental_phase	status
CaDi_045	Brachyura	Carcinidae	Parathranitiinae	*Parathranites*	*granosus*	adult	extant
CaDi_046	Brachyura	Carcinidae	Parathranitiinae	*Parathranites*	*tuberosus*	adult	extant
CaDi_047	Brachyura	Carcinidae	Parathranitiinae	*Parathranites*	*tuberosus*	adult	extant
CaDi_048	Brachyura	Carcinidae	Parathranitiinae	*Parathranites*	*tuberogranosus*	adult	extant
CaDi_049	Brachyura	Carcinidae	Parathranitiinae	*Parathranites*	*tuberogranosus*	adult	extant
CaDi_050	Brachyura	Carcinidae	Parathranitiinae	*Parathranites*	*hexagonus*	adult	extant
CaDi_051	Brachyura	Carcinidae	Parathranitiinae	*Parathranites*	*hexagonus*	adult	extant
CaDi_052	Brachyura	Carcinidae	Parathranitiinae	*Parathranites*	*orientalis*	adult	extant
CaDi_053	Brachyura	Carcinidae	Parathranitiinae	*Parathranites*	*orientalis*	adult	extant
CaDi_054	Brachyura	Carcinidae	Platyonichinae	*Portumnus*	*latipes*	megalopa	extant
CaDi_055	Brachyura	Carcinidae	Platyonichinae	*Portumnus*	*latipes*	juvenile	extant
CaDi_056	Brachyura	Carcinidae	Platyonichinae	*Portumnus*	*latipes*	megalopa	extant
CaDi_057	Brachyura	Carcinidae	Pirimelinae	*Pirimela*	*denticulata*	megalopa	extant
CaDi_058	Brachyura	Carcinidae	Platyonichinae	*Portumnus*	*latipes*	juvenile	extant
CaDi_059	Brachyura	Carcinidae	Pirimelinae	*Sirpus*	*ponticus*	adult	extant
CaDi_060	Brachyura	Carcinidae	Pirimelinae	*Sirpus*	*ponticus*	adult	extant
CaDi_061	Brachyura	Carcinidae	Pirimelinae	*Sirpus*	*monodi*	adult	extant
CaDi_062	Brachyura	Carcinidae	Pirimelinae	*Sirpus*	*zariquieyi*	adult	extant
CaDi_063	Brachyura	Carcinidae	Pirimelinae	*Sirpus*	*zariquieyi*	adult	extant
CaDi_064	Brachyura	Carcinidae	Pirimelinae	*Sirpus*	*zariquieyi*	adult	extant
CaDi_065	Brachyura	Carcinidae	Pirimelinae	*Sirpus*	*zariquieyi*	adult	extant

no	general_group	major_out	major_group	species_group	species	developmental_phase	status
CaDi_066	Brachyura	Carcinidae	Pirimelinae	*Sirpus*	*zariquieyi*	adult	extant
CaDi_067	Brachyura	Carcinidae	Platyonichinae	*Portumnus*	*latipes*	adult	extant
CaDi_068	Brachyura	Carcinidae	Platyonichinae	*Portumnus*	*latipes*	adult	extant
CaDi_069	Brachyura	Carcinidae	Platyonichinae	*Portumnus*	*latipes*	adult	extant
CaDi_070	Brachyura	Carcinidae	Platyonichinae	*Xaiva*	*mcleayi*	adult	extant
CaDi_071	Brachyura	Carcinidae	Platyonichinae	*Xaiva*	*biguttata*	adult	extant
CaDi_072	Brachyura	Carcinidae	Platyonichinae	*Xaiva*	*pulchella*	adult	extant
CaDi_073	Brachyura	Carcinidae	Platyonichinae	*Xaiva*	*biguttata*	adult	extant
CaDi_074	Brachyura	Carcinidae	Platyonichinae	*Xaiva*	*biguttata*	adult	extant
CaDi_075	Brachyura	Carcinidae	Platyonichinae	*Xaiva*	*biguttata*	adult	extant
CaDi_076	Brachyura	Carcinidae	Platyonichinae	*Xaiva*	*mcleayi*	juvenile	extant
CaDi_077	Brachyura	Carcinidae	Platyonichinae	*Xaiva*	*mcleayi*	adult	extant
CaDi_078	Brachyura	Carcinidae	Parathranitiinae	*Parathranites*	*orientalis*	adult	extant
CaDi_079	Brachyura	Carcinidae	Platyonichinae	*Xaiva*	*biguttata*	adult	extant
CaDi_080	Brachyura	Carcinidae	Platyonichinae	*Xaiva*	*mcleayi*	adult	extant
CaDi_081	Brachyura	Carcinidae	Platyonichinae	*Portumnus*	*latipes*	adult	extant
CaDi_082	Brachyura	Carcinidae	Carcininae	*Carcinides*	*minor*	adult	fossil
CaDi_083	Brachyura	Carcinidae	Carcininae	*Cicarnus*	*fumiae*	adult	fossil
CaDi_084	Brachyura	Carcinidae	Platyonichinae	*Portumnus*	*tricarinatus*	adult	fossil
CaDi_085	Brachyura	Carcinidae	Carcininae	*Miopipus*	*pygmaeus*	adult	fossil
CaDi_086	Brachyura	Carcinidae	Carcininae	*Miopipus*	*zovensis*	adult	fossil
CaDi_087	Brachyura	Carcinidae	Coelocarcininae	*Coelocarcinus*	*aff. foliatus*	adult	extant
CaDi_088	Brachyura	Carcinidae	Coelocarcininae	*Coelocarcinus*	*foliatus*	adult	extant

no	general_group	major_out	major_group	species_group	species	developmental_phase	status
CaDi_089	Brachyura	Carcinidae	Polybiidae	*Macropipus*	*tuberculatus*	adult	extant
CaDi_090	Brachyura	Carcinidae	Thiidae	*Thia*	*scutellata*	adult	extant
CaDi_091	Brachyura	Carcinidae	Polybiidae	*Necora*	*puber*	adult	extant
CaDi_092	Brachyura	Carcinidae	Polybiidae	*Bathynectes*	*maravigna*	adult	extant
CaDi_093	Brachyura	Carcinidae	Polybiidae	*Bathynectes*	*longispina*	adult	extant
CaDi_094	Brachyura	Carcinidae	Polybiidae	*Liocarcinus*	*maculatus*	adult	extant
CaDi_095	Brachyura	Carcinidae	Polybiidae	*Liocarcinus*	*navigator*	adult	extant
CaDi_096	Brachyura	Carcinidae	Polybiidae	*Polybius*	*henslowii*	adult	extant
CaDi_097	Brachyura	Carcinidae	Polybiidae	*Liocarcinus*	*holsatus*	adult	extant
CaDi_098	Brachyura	Carcinidae	Polybiidae	*Liocarcinus*	*depurator*	adult	extant
CaDi_099	Brachyura	Carcinidae	Polybiidae	*Liocarcinus*	*marmoreus*	adult	extant
CaDi_100	Brachyura	Carcinidae	Polybiidae	*Liocarcinus*	*vernalis*	adult	extant
CaDi_101	Brachyura	Carcinidae	Coelocarcininae	*Coelocarcinus*	*foliatus*	adult	extant
CaDi_102	Brachyura	Carcinidae	Coelocarcininae	*Coelocarcinus*	*marindicus*	adult	extant
CaDi_103	Brachyura	Carcinidae	Polybiidae	*Minohellenus*	*inexpressus*	adult	fossil
CaDi_104	Brachyura	Carcinidae	Polybiidae	*Maeandricampus*	*triangulum*	adult	fossil
CaDi_105	Brachyura	Carcinidae	Polybiidae	*Maeandricampus*	*starri*	adult	fossil
CaDi_106	Brachyura	Carcinidae	Polybiidae	*Bathynectes*	*muelleri*	adult	fossil
CaDi_107	Brachyura	Carcinidae	Polybiidae	*Bathynectes*	*maravigna*	adult	extant

no	general_group	major_out	major_group	species_group	species	developmental_phase	status
CaDi_108	Brachyura	Carcinidae	Polybiidae	*Bathynectes*	*piperitus*	adult	extant
CaDi_109	Brachyura	Carcinidae	Polybiidae	*Liocarcinus*	*corrugatus*	adult	extant
CaDi_110	Brachyura	Carcinidae	Polybiidae	*Liocarcinus*	*corrugatus*	megalopa	extant
CaDi_111	Brachyura	Carcinidae	Polybiidae	*Minohellenus*	*traingulum*	adult	fossil
CaDi_112	Brachyura	Carcinidae	Polybiidae	*Minohellenus*	*macrocheilus*	adult	fossil
CaDi_113	Brachyura	Carcinidae	Polybiidae	*Olicarcinus*	*trevisani*	adult	fossil
CaDi_114	Brachyura	Carcinidae	Polybiidae	*Liocarcinus*	*holsatus*	juvenile	fossil
CaDi_115	Brachyura	Carcinidae	Polybiidae	*Liocarcinus*	*depurator*	juvenile	extant
CaDi_116	Brachyura	Carcinidae	Polybiidae	*Liocarcinus*	*depurator*	juvenile	extant
CaDi_117	Brachyura	Carcinidae	Polybiidae	*Liocarcinus*	*depurator*	megalopa	extant
CaDi_118	Brachyura	Carcinidae	Polybiidae	*Liocarcinus*	*heintzi*	adult	fossil
CaDi_119	Brachyura	Carcinidae	Carcinidae	*Miopipus*	*zavensis*	adult	fossil
CaDi_120	Brachyura	Carcinidae	Polybiidae	*Megokkos*	sp.	adult	fossil
CaDi_121	Brachyura	Carcinidae	Polybiidae	*Megokkos*	*alaskensis*	adult	fossil

no	general_group	major_out	major_group	species_group	species	developmental_phase	status
CaDi_122	Brachyura	Carcinidae	Polybiidae	*Megokkos*	*alaskensis*	adult	fossil
CaDi_123	Brachyura	Carcinidae	Polybiidae	*Bathynectes*	*maravigna*	adult	extant
CaDi_124	Brachyura	Carcinidae	Polybiidae	*Liocarcinus*	*corrugatus*	adult	extant
CaDi_125	Brachyura	Carcinidae	Polybiidae	*Liocarcinus*	*corrugatus*	adult	extant
CaDi_126	Brachyura	Carcinidae	Polybiidae	*Liocarcinus*	*depurator*	adult	extant
CaDi_127	Brachyura	Carcinidae	Polybiidae	*Liocarcinus*	*pusillus*	adult	extant
CaDi_128	Brachyura	Carcinidae	Polybiidae	*Liocarcinus*	*pusillus*	adult	extant

no	general_group	major_out	major_group	species_group	species	developmental_phase	status
CaDi_129	Brachyura	Carcinidae	Polybiidae	*Liocarcinus*	*vernalis*	adult	extant
CaDi_130	Brachyura	Carcinidae	Polybiidae	*Liocarcinus*	*zariquieyi*	adult	extant
CaDi_131	Brachyura	Carcinidae	Polybiidae	*Macropipus*	*australis*	adult	extant
CaDi_132	Brachyura	Carcinidae	Polybiidae	*Macropipus*	*tuberculatus*	adult	extant
CaDi_133	Brachyura	Carcinidae	Polybiidae	*Thia*	*scutellata*	adult	extant
CaDi_134	Brachyura	Carcinidae	Polybiidae	*Thia*	*scutellata*	adult	extant
CaDi_135	Brachyura	Carcinidae	Polybiidae	*Macropipus*	*tuberculatus*	zoea	extant
CaDi_136	Brachyura	Carcinidae	Polybiidae	*Macropipus*	*tuberculatus*	zoea	extant
CaDi_137	Brachyura	Carcinidae	Polybiidae	*Macropipus*	*tuberculatus*	zoea	extant
CaDi_138	Brachyura	Carcinidae	Polybiidae	*Necora*	*puber*	zoea	extant
CaDi_139	Brachyura	Carcinidae	Polybiidae	*Liocarcinus*	*holsatus*	zoea	extant
CaDi_140	Brachyura	Carcinidae	Polybiidae	*Necora*	*puber*	zoea	extant

no	general_group	major_out	major_group	species_group	species	developmental_phase	status
CaDi_141	Brachyura	Carcinidae	Polybiidae	*Liocarcinus*	*holsatus*	zoea	extant
CaDi_142	Brachyura	Carcinidae	Polybiidae	*Necora*	*puber*	zoea	extant
CaDi_143	Brachyura	Carcinidae	Polybiidae	*Liocarcinus*	*holsatus*	zoea	extant
CaDi_144	Brachyura	Carcinidae	Polybiidae	*Necora*	*puber*	zoea	extant
CaDi_145	Brachyura	Carcinidae	Polybiidae	*Liocarcinus*	*holsatus*	zoea	extant
CaDi_146	Brachyura	Carcinidae	Polybiidae	*Necora*	*puber*	zoea	extant
CaDi_147	Brachyura	Carcinidae	Polybiidae	*Liocarcinus*	*holsatus*	zoea	extant
car_001	Brachyura	Carcinidae	Carcininae	*Carcinus*	*maenas*	adult	extant
car_002	Brachyura	Carcinidae	Carcininae	*Carcinus*	*maenas*	adult	extant
car_003	Brachyura	Carcinidae	Carcininae	*Carcinus*	*maenas*	adult	extant
car_004	Brachyura	Carcinidae	Carcininae	*Carcinus*	*maenas*	adult	extant
car_005	Brachyura	Carcinidae	Carcininae	*Carcinus*	*maenas*	adult	extant
car_006	Brachyura	Carcinidae	Carcininae	*Carcinus*	*maenas*	adult	extant
car_007	Brachyura	Carcinidae	Carcininae	*Carcinus*	*maenas*	adult	extant
car_008	Brachyura	Carcinidae	Carcininae	*Carcinus*	*maenas*	adult	extant
car_009	Brachyura	Carcinidae	Carcininae	*Carcinus*	*maenas*	megalopa	extant
car_010	Brachyura	Carcinidae	Carcininae	*Carcinus*	*maenas*	adult	extant
car_011	Brachyura	Carcinidae	Carcininae	*Carcinus*	*maenas*	adult	extant
car_012	Brachyura	Carcinidae	Carcininae	*Carcinus*	*maenas*	adult	extant
car_013	Brachyura	Carcinidae	Carcininae	*Carcinus*	*maenas*	adult	extant
car_014	Brachyura	Carcinidae	Carcininae	*Carcinus*	*maenas*	adult	extant
car_015	Brachyura	Carcinidae	Carcininae	*Carcinus*	*maenas*	adult	extant
car_016	Brachyura	Carcinidae	Carcininae	*Carcinus*	*maenas*	megalopa	extant
car_017	Brachyura	Carcinidae	Carcininae	*Carcinus*	*maenas*	megalopa	extant
car_018	Brachyura	Carcinidae	Carcininae	*Carcinus*	*maenas*	megalopa	extant
car_019	Brachyura	Carcinidae	Carcininae	*Carcinus*	*maenas*	megalopa	extant
car_020	Brachyura	Carcinidae	Carcininae	*Carcinus*	*maenas*	megalopa	extant
car_021	Brachyura	Carcinidae	Carcininae	*Carcinus*	*maenas*	megalopa	extant
car_022	Brachyura	Carcinidae	Carcininae	*Carcinus*	*maenas*	megalopa	extant
car_023	Brachyura	Carcinidae	Carcininae	*Carcinus*	*maenas*	megalopa	extant
car_024	Brachyura	Carcinidae	Carcininae	*Carcinus*	*maenas*	megalopa	extant

no	general_group	major_out	major_group	species_group	species	developmental_phase	status
car_025	Brachyura	Carcinidae	Carcininae	*Carcinus*	*maenas*	megalopa	extant
car_026	Brachyura	Carcinidae	Carcininae	*Carcinus*	*maenas*	megalopa	extant
car_027	Brachyura	Carcinidae	Carcininae	*Carcinus*	*maenas*	megalopa	extant
car_028	Brachyura	Carcinidae	Carcininae	*Carcinus*	*maenas*	megalopa	extant
car_029	Brachyura	Carcinidae	Carcininae	*Carcinus*	*maenas*	juvenile	extant
car_030	Brachyura	Carcinidae	Carcininae	*Carcinus*	*maenas*	juvenile	extant
car_031	Brachyura	Carcinidae	Carcininae	*Carcinus*	*maenas*	juvenile	extant
car_032	Brachyura	Carcinidae	Carcininae	*Carcinus*	*maenas*	megalopa	extant
car_033	Brachyura	Carcinidae	Carcininae	*Carcinus*	*maenas*	megalopa	extant
car_034	Brachyura	Carcinidae	Carcininae	*Carcinus*	*maenas*	megalopa	extant
car_035	Brachyura	Carcinidae	Carcininae	*Carcinus*	*maenas*	megalopa	extant
car_036	Brachyura	Carcinidae	Carcininae	*Carcinus*	*maenas*	megalopa	extant
car_037	Brachyura	Carcinidae	Carcininae	*Carcinus*	*maenas*	juvenile	extant
car_038	Brachyura	Carcinidae	Carcininae	*Carcinus*	*maenas*	juvenile	extant
car_039	Brachyura	Carcinidae	Carcininae	*Carcinus*	*maenas*	juvenile	extant
car_040	Brachyura	Carcinidae	Carcininae	*Carcinus*	*maenas*	juvenile	extant
car_041	Brachyura	Carcinidae	Carcininae	*Carcinus*	*maenas*	juvenile	extant
car_042	Brachyura	Carcinidae	Carcininae	*Carcinus*	*maenas*	juvenile	extant
car_043	Brachyura	Carcinidae	Carcininae	*Carcinus*	*maenas*	juvenile	extant
car_044	Brachyura	Carcinidae	Carcininae	*Carcinus*	*maenas*	juvenile	extant
car_045	Brachyura	Carcinidae	Carcininae	*Carcinus*	*maenas*	juvenile	extant
car_046	Brachyura	Carcinidae	Carcininae	*Carcinus*	*maenas*	juvenile	extant
car_047	Brachyura	Carcinidae	Carcininae	*Carcinus*	*maenas*	adult	extant
car_048	Brachyura	Carcinidae	Carcininae	*Carcinus*	*maenas*	adult	extant

no	general_group	major_out	major_group	species_group	species	developmental_phase	status
car_049	Brachyura	Carcinidae	Carcininae	*Carcinus*	*maenas*	adult	extant
car_050	Brachyura	Carcinidae	Carcininae	*Carcinus*	*maenas*	adult	extant
car_051	Brachyura	Carcinidae	Carcininae	*Carcinus*	*maenas*	adult	extant
car_052	Brachyura	Carcinidae	Carcininae	*Carcinus*	*maenas*	adult	extant
car_053	Brachyura	Carcinidae	Carcininae	*Carcinus*	*maenas*	adult	extant
car_054	Brachyura	Carcinidae	Carcininae	*Carcinus*	*maenas*	adult	extant
car_055	Brachyura	Carcinidae	Carcininae	*Carcinus*	*maenas*	adult	extant
car_056	Brachyura	Carcinidae	Carcininae	*Carcinus*	*maenas*	adult	extant
car_057	Brachyura	Carcinidae	Carcininae	*Carcinus*	*maenas*	adult	extant
Dio_001	Anomala	Paguroidea	Diogenidae	*Clibanarius*	*aequabilis*	zoea	extant
Dio_002	Anomala	Paguroidea	Diogenidae	*Clibanarius*	*aequabilis*	zoea	extant
Dio_003	Anomala	Paguroidea	Diogenidae	*Clibanarius*	*aequabilis*	megalopa	extant
Dio_004	Anomala	Paguroidea	Diogenidae	*Diogenes*	*planimanus*	zoea	extant
Dio_005	Anomala	Paguroidea	Diogenidae	*Diogenes*	*planimanus*	zoea	extant
Dio_006	Anomala	Paguroidea	Diogenidae	*Diogenes*	*planimanus*	zoea	extant
Dio_007	Anomala	Paguroidea	Diogenidae	*Diogenes*	*planimanus*	megalopa	extant

no	general_group	major_out	major_group	species_group	species	developmental_phase	status
Dio_008	Anomala	Paguroidea	Diogenidae	*Diogenes*	*planimanus*	juvenile	extant
Dio_009	Anomala	Paguroidea	Diogenidae	*Diogenes*	*manaarensis*	zoea	extant
Dio_010	Anomala	Paguroidea	Diogenidae	*Diogenes*	*manaarensis*	zoea	extant
Dio_011	Anomala	Paguroidea	Diogenidae	*Diogenes*	*manaarensis*	megalopa	extant
Dio_012	Anomala	Paguroidea	Diogenidae	*Diogenes*	*nitidimanus*	adult	extant
Dio_013	Anomala	Paguroidea	Diogenidae	*Diogenes*	*nitidimanus*	zoea	extant
Dio_014	Anomala	Paguroidea	Diogenidae	*Diogenes*	*nitidimanus*	zoea	extant
Dio_015	Anomala	Paguroidea	Diogenidae	*Diogenes*	*nitidimanus*	zoea	extant
Dio_016	Anomala	Paguroidea	Diogenidae	*Diogenes*	*nitidimanus*	zoea	extant
Dio_017	Anomala	Paguroidea	Diogenidae	*Diogenes*	*nitidimanus*	megalopa	extant
Dio_018	Anomala	Paguroidea	Diogenidae	*Clibanarius*	*antillensis*	megalopa	extant
Dio_019	Anomala	Paguroidea	Diogenidae	*Clibanarius*	*antillensis*	adult	extant
Dio_020	Anomala	Paguroidea	Diogenidae	*Clibanarius*	*antillensis*	adult	extant
Dio_021	Anomala	Paguroidea	Diogenidae	*Clibanarius*	*vittatus*	megalopa	extant
Dio_022	Anomala	Paguroidea	Diogenidae	*Paguristes*	*spinipes*	megalopa	extant
Dio_023	Anomala	Paguroidea	Diogenidae	*Areopaguristes*	*japonicus*	zoea	extant
Dio_024	Anomala	Paguroidea	Diogenidae	*Areopaguristes*	*japonicus*	megalopa	extant
Dio_025	Anomala	Paguroidea	Diogenidae	*Paguristes*	*tortugae*	zoea	extant
Dio_026	Anomala	Paguroidea	Diogenidae	*Paguristes*	*tortugae*	zoea	extant
Dio_027	Anomala	Paguroidea	Diogenidae	*Paguristes*	*tortugae*	zoea	extant
Dio_028	Anomala	Paguroidea	Diogenidae	*Paguristes*	*tortugae*	megalopa	extant
Dio_029	Anomala	Paguroidea	Diogenidae	*Clibanarius*	*antillensis*	zoea	extant

no	general_group	major_out	major_group	species_group	species	developmental_phase	status
Dio_030	Anomala	Paguroidea	Diogenidae	*Clibanarius*	*antillensis*	zoea	extant
Dio_031	Anomala	Paguroidea	Diogenidae	*Clibanarius*	*antillensis*	zoea	extant
Dio_032	Anomala	Paguroidea	Diogenidae	*Clibanarius*	*antillensis*	zoea	extant
Dio_033	Anomala	Paguroidea	Diogenidae	*Clibanarius*	*antillensis*	zoea	extant
Dio_034	Anomala	Paguroidea	Diogenidae	*Clibanarius*	*antillensis*	megalopa	extant
Dio_035	Anomala	Paguroidea	Diogenidae	*Diogenes*	*manaarensis*	adult	extant
Dio_036	Anomala	Paguroidea	Diogenidae	*Diogenes*	*jousseaumei*	adult	extant
Dio_037	Anomala	Paguroidea	Diogenidae	*Diogenes*	*jubatus*	adult	extant
Dio_038	Anomala	Paguroidea	Diogenidae	*Diogenes*	*platyops*	adult	extant
Dio_039	Anomala	Paguroidea	Diogenidae	*Diogenes*	*jubatus*	adult	extant
Dio_040	Anomala	Paguroidea	Diogenidae	*Diogenes*	*persicus*	adult	extant
Dio_041	Anomala	Paguroidea	Diogenidae	*Diogenes*	*persicus*	adult	extant
Dio_042	Anomala	Paguroidea	Diogenidae	*Diogenes*	*mercatoris*	adult	extant
Dio_043	Anomala	Paguroidea	Diogenidae	*Diogenes*	*crosnieri*	adult	extant
Dio_044	Anomala	Paguroidea	Diogenidae	*Diogenes*	*erythromanus*	adult	extant
Dio_045	Anomala	Paguroidea	Diogenidae	*Diogenes*	*arguinensis*	adult	extant
Dio_046	Anomala	Paguroidea	Diogenidae	*Clibanarius*	*vittatus*	adult	extant
Dio_047	Anomala	Paguroidea	Diogenidae	*Clibanarius*	*symmetricus*	adult	extant
Dio_048	Anomala	Paguroidea	Diogenidae	*Clibanarius*	*hirsutimanus*	adult	extant
Dio_049	Anomala	Paguroidea	Diogenidae	*Clibanarius*	*sachalinicus*	adult	extant
Dio_050	Anomala	Paguroidea	Diogenidae	*Clibanarius*	*harisi*	adult	extant
Dio_051	Anomala	Paguroidea	Diogenidae	*Clibanarius*	*serenei*	adult	extant
Dio_052	Anomala	Paguroidea	Diogenidae	*Clibanarius*	*snelliusi*	adult	extant
Dio_053	Anomala	Paguroidea	Diogenidae	*Clibanarius*	*striolatus*	adult	extant
Dio_054	Anomala	Paguroidea	Diogenidae	*Clibanarius*	*virescens*	adult	extant
Dio_055	Anomala	Paguroidea	Diogenidae	*Areopaguristes*	*japonicus*	adult	extant
Dio_056	Anomala	Paguroidea	Diogenidae	*Areopaguristes*	*nigroapiculus*	adult	extant

no	general_group	major_out	major_group	species_group	species	developmental_phase	status
Dio_057	Anomala	Paguroidea	Diogenidae	*Paguristes*	*digitalis*	adult	extant
Dio_058	Anomala	Paguroidea	Diogenidae	*Paguristes*	*ortmanni*	adult	extant
Dio_059	Anomala	Paguroidea	Diogenidae	*Paguristes*	*tortugae*	adult	extant
Dio_060	Anomala	Paguroidea	Diogenidae	*Paguristes*	*spinipes*	adult	extant
dro_001	Brachyura	Dromiidae	*Dromiinae*	*Cryptodromia*	*tuberculata*	adult	extant
dro_002	Brachyura	Dromiidae	*Dromiinae*	*Cryptodromia*	*pileifera*	adult	extant
dro_003	Brachyura	Dromiidae	*Dromiinae*	*Takedromia*	*longispina*	adult	extant
dro_004	Brachyura	Dromiidae	*Sphaerodromiinae*	*Sphaerodromia*	*ducoussoi*	adult	extant
dro_005	Brachyura	Dromiidae	*Dromiinae*	*Dromidiopsis*	*richeri*	adult	extant
dro_006	Brachyura	Dromiidae	*Dromiinae*	*Cryptodromia*	*marquesas*	adult	extant
dro_007	Brachyura	Dromiidae	*Dromiinae*	*Cryptodromia*	*erioxylon*	adult	extant
dro_008	Brachyura	Dromiidae	*Dromiinae*	*Lewindromia*	*unidentata*	adult	extant
dro_009	Brachyura	Dromiidae	*Dromiinae*	*Cryptodromia*	*hilgendorfi*	adult	extant
dro_010	Brachyura	Dromiidae	*Dromiinae*	*Cryptodromia*	*bullifera*	adult	extant
dro_011	Brachyura	Dromiidae	*Dromiinae*	*Epigodromia*	*granulata*	adult	extant
dro_012	Brachyura	Dromiidae	*Dromiinae*	*Epigodromia*	*gilesii*	adult	extant
dro_013	Brachyura	Dromiidae	*Dromiinae*	*Epigodromia*	*globosa*	adult	extant
dro_014	Brachyura	Dromiidae	*Dromiinae*	*Petalomera*	sp.	adult	extant
dro_015	Brachyura	Dromiidae	*Dromiinae*	*Ascidiophilus*	*caphyraeformis*	adult	extant
dro_016	Brachyura	Dromiidae	*Sphaerodromiinae*	*Eodromia*	*denticulata*	adult	extant
dro_017	Brachyura	Dromiidae	*Sphaerodromiinae*	*Frodromia*	*atypica*	adult	extant
dro_018	Brachyura	Dromiidae	*Dromiinae*	*Cryptodromia*	*x*	adult	extant
dro_019	Brachyura	Dromiidae	*Dromiinae*	*Takedromia*	*cristatipes*	adult	extant
dro_020	Brachyura	Dromiidae	*Dromiinae*	*Takedromia*	*longispina*	adult	extant
dro_021	Brachyura	Dromiidae	*Dromiinae*	*Epigodromia*	*rotunda*	adult	extant
dro_022	Brachyura	Dromiidae	*Dromiinae*	*Epigodromia*	*rugosa*	adult	extant

no	general_group	major_out	major_group	species_group	species	developmental_phase	status
dro_023	Brachyura	Dromiidae	*Dromiinae*	*Homalodromia*	*coppingeri*	adult	extant
dro_024	Brachyura	Dromiidae	*Dromiinae*	*Conchoecetes*	*intermedius*	adult	extant
dro_025	Brachyura	Dromiidae	*Dromiinae*	*Conchoecetes*	*intermedius*	adult	extant
dro_026	Brachyura	Dromiidae	*Dromiinae*	*Conchoecetes*	*artificiosus*	adult	extant
dro_027	Brachyura	Dromiidae	*Dromiinae*	*Cryptodromia*	*stimpson*	adult	extant
dro_028	Brachyura	Dromiidae	*Dromiinae*	*Cryptodromia*	sp.	adult	extant
dro_029	Brachyura	Dromiidae	*Dromiinae*	*Cryptodromia*	sp.	adult	extant
dro_030	Brachyura	Dromiidae	*Dromiinae*	*Cryptodromia*	sp.	adult	extant
dro_031	Brachyura	Dromiidae	*Dromiinae*	*Cryptodromia*	sp.	adult	extant

no	general_group	major_out	major_group	species_group	species	developmental_phase	status
dro_032	Brachyura	Dromiidae	*Dromiinae*	*Cryptodromia*	*longipes*	adult	extant
dro_033	Brachyura	Dromiidae	*Dromiinae*	*Cryptodromia*	*pitiensis*	adult	extant
dro_034	Brachyura	Dromiidae	*Dromiinae*	*Cryptodromia*	sp.	adult	extant
dro_035	Brachyura	Dromiidae	*Dromiinae*	*Dromia*	*bucklandii*	adult	fossil
dro_036	Brachyura	Dromiidae	*Dromiinae*	*Dromia*	*marmorea*	adult	extant
dro_037	Brachyura	Dromiidae	*Dromiinae*	*Dromia*	*lator*	adult	extant
dro_038	Brachyura	Dromiidae	*Dromiinae*	*Dromia*	*marmorea*	adult	extant

no	general_group	major_out	major_group	species_group	species	developmental_phase	status
dro_039	Brachyura	Dromiidae	*Dromiinae*	*Dromia*	*bollorei*	adult	extant
dro_040	Brachyura	Dromiidae	*Dromiinae*	*Dromia*	*bollorei*	adult	extant
dro_041	Brachyura	Dromiidae	*Dromiinae*	*Dromia*	*marmorea*	adult	extant
dro_042	Brachyura	Dromiidae	*Dromiinae*	*Dromia*	*marmorea*	adult	extant
dro_043	Brachyura	Dromiidae	*Dromiinae*	*Dromia*	*marmorea*	adult	extant
dro_044	Brachyura	Dromiidae	*Dromiinae*	*Dromia*	*marmorea*	adult	extant
dro_045	Brachyura	Dromiidae	*Dromiinae*	*Dromidia*	*lepidopta*	adult	extant

no	general_group	major_out	major_group	species_group	species	developmental_phase	status
dro_046	Brachyura	Dromiidae	*Dromiinae*	*Dromidia*	*hirsutissima*	adult	extant
dro_047	Brachyura	Dromiidae	*Dromiinae*	*Dromidiopsis*	*tridentata*	adult	extant
dro_048	Brachyura	Dromiidae	*Dromiinae*	*Dromidiopsis*	sp.	adult	extant
dro_049	Brachyura	Dromiidae	*Dromiinae*	*Dromidiopsis*	sp.	adult	extant
dro_050	Brachyura	Dromiidae	*Dromiinae*	*Dromidiopsis*	sp.	adult	extant
dro_051	Brachyura	Dromiidae	*Dromiinae*	*Dromidiopsis*	*edwardsi*	adult	extant
dro_052	Brachyura	Dromiidae	*Dromiinae*	*Haledromia*	*bicavernosa*	adult	extant
dro_053	Brachyura	Dromiidae	*Dromiinae*	*Hemisphaerodromia*	*abellana*	adult	extant

no	general_group	major_out	major_group	species_group	species	developmental_phase	status
dro_054	Brachyura	Dromiidae	*Dromiinae*	*Epigrodromia*	*rotunda*	adult	extant
dro_055	Brachyura	Dromiidae	*Dromiinae*	*Lauridromia*	*intermedia*	adult	extant
dro_056	Brachyura	Dromiidae	*Dromiinae*	*Mclaydromia*	*colini*	adult	extant
dro_057	Brachyura	Dromiidae	*Dromiinae*	*Mclaydromia*	*colini*	adult	extant
dro_058	Brachyura	Dromiidae	*Dromiinae*	*Mclaydromia*	*colini*	adult	extant
dro_059	Brachyura	Dromiidae	*Dromiinae*	*Mclaydromia*	*colini*	adult	extant
dro_060	Brachyura	Dromiidae	*Dromiinae*	*Mclaydromia*	*colini*	adult	extant

no	general_group	major_out	major_group	species_group	species	developmental_phase	status
dro_061	Brachyura	Dromiidae	*Dromiinae*	*Moreiradromia*	*antillensis*	adult	extant
dro_062	Brachyura	Dromiidae	*Dromiinae*	*Moreiradromia*	*antillensis*	adult	extant
dro_063	Brachyura	Dromiidae	*Dromiinae*	*Paradromia*	sp.	adult	extant
dro_064	Brachyura	Dromiidae	*Dromiinae*	*Stimdromia*	*lateralis*	adult	extant
dro_065	Brachyura	Dromiidae	*Dromiinae*	*Dromidiopsis*	*plumosa*	adult	extant

no	general_group	major_out	major_group	species_group	species	developmental_phase	status
dro_066	Brachyura	Dromiidae	*Dromiinae*	*Stebbingdromia*	*plumosa*	adult	extant
dro_067	Brachyura	Dromiidae	*Dromiinae*	*Dromia*	*monodi*	adult	extant
dro_068	Brachyura	Dromiidae	*Dromiinae*	*Stimdromia*	sp.	adult	extant
dro_069	Brachyura	Dromiidae	*Dromiinae*	*Stimdromia*	sp.	adult	extant
dro_070	Brachyura	Dromiidae	*Dromiinae*	*Platydromia*	*spongiosa*	adult	extant
dro_071	Brachyura	Dromiidae	*Dromiinae*	*Platydromia*	*spongiosa*	adult	extant
dro_072	Brachyura	Dromiidae	*Dromiinae*	*Platydromia*	*spongiosa*	adult	extant

no	general_group	major_out	major_group	species_group	species	developmental_phase	status
dro_073	Brachyura	Dromiidae	*Dromiinae*	*Platydromia*	*spongiosa*	adult	extant
dro_074	Brachyura	Dromiidae	*Dromiinae*	*Platydromia*	*spongiosa*	adult	extant
dro_075	Brachyura	Dromiidae	*Dromiinae*	*Platydromia*	*spongiosa*	adult	extant
dro_076	Brachyura	Dromiidae	*Dromiinae*	*Platydromia*	*spongiosa*	adult	extant
dro_079	Brachyura	Dromiidae	*Sphaerodromiinae*	*Dromilites*	*bucklandii*	adult	fossil
dro_080	Brachyura	Dromiidae	*Sphaerodromiinae*	*Dromilites*	*bucklandii*	adult	fossil
dro_081	Brachyura	Dromiidae	*Sphaerodromiinae*	*Dromilites*	*subglosa*	adult	fossil
dro_082	Brachyura	Dromiidae	*Sphaerodromiinae*	*Ferricorda*	*kimberleyae*	adult	fossil
dro_083	Brachyura	Dromiidae	*Dromiinae*	*Quinquerugatus*	*holthuisi*	adult	fossil
dro_084	Brachyura	Dromiidae	*Dromiinae*	*Quinquerugatus*	*holthuisi*	adult	fossil
dro_085	Brachyura	Dromiidae	*Dromiinae*	*Moreiradromia*	*bedetteae*	adult	fossil
dro_086	Brachyura	Dromiidae	*Dromiinae*	*Basadromia*	*longifrons*	adult	fossil
dro_087	Brachyura	Dromiidae	*Dromiidae*	*Kierionopsis*	*nodosa*	adult	fossil
dro_088	Brachyura	Dromiidae	*Dromiinae*	*Dromidia*	*bedetteae*	adult	fossil

no	general_group	major_out	major_group	species_group	species	developmental_phase	status
dro_089	Brachyura	Dromiidae	*Dromiinae*	*Dromidia*	*antillensis*	zoea	extant
dro_090	Brachyura	Dromiidae	*Dromiinae*	*Dromidia*	*antillensis*	zoea	extant
dro_091	Brachyura	Dromiidae	*Dromiinae*	*Dromidia*	*antillensis*	zoea	extant
dro_092	Brachyura	Dromiidae	*Dromiinae*	*Dromidia*	*antillensis*	zoea	extant
dro_093	Brachyura	Dromiidae	*Dromiinae*	*Dromidia*	*antillensis*	zoea	extant
dro_094	Brachyura	Dromiidae	*Dromiinae*	*Dromidia*	*antillensis*	zoea	extant
dro_095	Brachyura	Dromiidae	*Dromiinae*	*Dromidia*	*antillensis*	megalopa	extant
dro_096	Brachyura	Dromiidae	*Dromiinae*	*Petalomera*	*japonica*	zoea	extant
dro_097	Brachyura	Dromiidae	*Dromiinae*	*Petalomera*	*japonica*	zoea	extant
dro_098	Brachyura	Dromiidae	*Dromiinae*	*Petalomera*	*japonica*	megalopa	extant
dro_099	Brachyura	Dromiidae	*Dromiinae*	*Dromia*	*personata*	zoea	extant
dro_100	Brachyura	Dromiidae	*Dromiinae*	*Dromia*	*personata*	zoea	extant
dro_101	Brachyura	Dromiidae	*Dromiinae*	*Dromia*	*personata*	zoea	extant
dro_102	Brachyura	Dromiidae	*Dromiinae*	*Dromia*	*personata*	megalopa	extant
dro_103	Brachyura	Dromiidae	*Dromiinae*	*Dromia*	*erythropus*	zoea	extant
dro_104	Brachyura	Dromiidae	*Dromiinae*	*Dromia*	*erythropus*	zoea	extant
dro_105	Brachyura	Dromiidae	*Dromiinae*	*Dromia*	*erythropus*	zoea	extant
dro_106	Brachyura	Dromiidae	*Dromiinae*	*Dromia*	*erythropus*	zoea	extant
dro_107	Brachyura	Dromiidae	*Dromiinae*	*Dromia*	*erythropus*	zoea	extant
dro_108	Brachyura	Dromiidae	*Dromiinae*	*Dromia*	*erythropus*	megalopa	extant
dro_109	Brachyura	Dromiidae	*Dromiinae*	*Cryptodromia*	*pileifera*	megalopa	extant
dro_110	Brachyura	Dromiidae	*Dromiinae*	*Petalomera*	*wilsoni*	zoea	extant
dro_111	Brachyura	Dromiidae	*Dromiinae*	*Petalomera*	*wilsoni*	zoea	extant

no	general_group	major_out	major_group	species_group	species	developmental_phase	status
dro_112	Brachyura	Dromiidae	*Sphaerodromiinae*	*Eodromia*	*denticulata*	adult	extant
dro_113	Brachyura	Dromiidae	*Sphaerodromiinae*	*Eodromia*	*denticulata*	adult	extant
dro_114	Brachyura	Dromiidae	*Hypoconchinae*	*Hypoconcha*	sp.	adult	extant
dro_115	Brachyura	Dromiidae	*Sphaerodromiinae*	*Sphaerodromia*	*brizops*	adult	extant
dro_116	Brachyura	Dromiidae	*Sphaerodromiinae*	*Sphaerodromia*	*ducoussoi*	adult	extant
dro_117	Brachyura	Dromiidae	*Dromiinae*	*Dromidiopsis*	*plumosa*	adult	extant
dro_118	Brachyura	Dromiidae	*Dromiinae*	*Stimdromia*	sp.	adult	extant

no	general_group	major_out	major_group	species_group	species	developmental_phase	status
dro_119	Brachyura	Dromiidae	*Dromiinae*	*Stimdromia*	sp.	adult	extant
dro_120	Brachyura	Dromiidae	*Dromiinae*	*Dromia*	*monodi*	adult	extant
dro_121	Brachyura	Dromiidae	*Dromiinae*	*Takedromia*	*longispina*	adult	extant
dro_122	Brachyura	Dromiidae	*Sphaerodromiinae*	*Sphaerodromia*	*ducoussoi*	adult	extant
gal_001	Anomala	Galatheidae	Galatheidae	unknown	unknown	zoea	extant
gal_002	Anomala	Galatheidae	Galatheidae	unknown	unknown	zoea	extant
gal_003	Anomala	Galatheidae	Galatheidae	unknown	unknown	zoea	extant
gal_004	Anomala	Galatheidae	Galatheidae	unknown	unknown	zoea	extant
gal_005	Anomala	Galatheidae	Galatheidae	unknown	unknown	zoea	extant
gal_006	Anomala	Galatheidae	Galatheidae	unknown	unknown	zoea	extant

no	general_group	major_out	major_group	species_group	species	developmental_phase	status
gal_007	Anomala	Galatheidae	Galatheidae	unknown	unknown	zoea	extant
gal_009	Anomala	Galatheidae	Galatheidae	unknown	unknown	zoea	extant
gal_010	Anomala	Galatheidae	Galatheidae	*Galathea*	*intermedia*	zoea	extant
gal_011	Anomala	Galatheidae	Galatheidae	*Galathea*	*intermedia*	zoea	extant
gal_012	Anomala	Galatheidae	Galatheidae	*Galathea*	*intermedia*	zoea	extant
gal_013	Anomala	Galatheidae	Galatheidae	*Galathea*	*intermedia*	zoea	extant
gal_014	Anomala	Galatheidae	Galatheidae	*Pleuroncodes*	*monodon*	zoea	extant
gal_015	Anomala	Galatheidae	Galatheidae	*Pleuroncodes*	*monodon*	zoea	extant
gal_016	Anomala	Galatheidae	Galatheidae	*Pleuroncodes*	*monodon*	zoea	extant
gal_017	Anomala	Galatheidae	Galatheidae	*Pleuroncodes*	*monodon*	zoea	extant
gal_018	Anomala	Galatheidae	Galatheidae	*Pleuroncodes*	*monodon*	zoea	extant
gal_019	Anomala	Galatheidae	Galatheidae	*Galathea*	*inflata*	zoea	extant
gal_020	Anomala	Galatheidae	Galatheidae	*Galathea*	*inflata*	zoea	extant
gal_021	Anomala	Galatheidae	Galatheidae	*Galathea*	*inflata*	zoea	extant
gal_022	Anomala	Galatheidae	Galatheidae	*Galathea*	*inflata*	zoea	extant
gal_023	Anomala	Galatheidae	Galatheidae	*Galathea*	*inflata*	zoea	extant
gal_024	Anomala	Galatheidae	Galatheidae	*Galathea*	*amboinensis*	zoea	extant
gal_025	Anomala	Galatheidae	Galatheidae	*Galathea*	*amboinensis*	zoea	extant
gal_026	Anomala	Galatheidae	Galatheidae	*Galathea*	*amboinensis*	zoea	extant
gal_027	Anomala	Galatheidae	Galatheidae	*Galathea*	*amboinensis*	zoea	extant
gal_028	Anomala	Galatheidae	Galatheidae	*Munida*	*rugosa*	zoea	extant
gal_029	Anomala	Galatheidae	Galatheidae	*Munida*	*rugosa*	zoea	extant

no	general_group	major_out	major_group	species_group	species	developmental_phase	status
gal_030	Anomala	Galatheidae	Galatheidae	*Galathea*	*strigosa*	zoea	extant
gal_031	Anomala	Galatheidae	Galatheidae	*Galathea*	*strigosa*	zoea	extant
gal_032	Anomala	Galatheidae	Galatheidae	*Galathea*	*strigosa*	zoea	extant
gal_033	Anomala	Galatheidae	Galatheidae	*Galathea*	*strigosa*	zoea	extant
gal_034	Anomala	Galatheidae	Galatheidae	*Galathea*	*dispersa*	zoea	extant
gal_035	Anomala	Galatheidae	Galatheidae	*Galathea*	*dispersa*	zoea	extant
gal_036	Anomala	Galatheidae	Galatheidae	*Galathea*	*dispersa*	zoea	extant
gal_037	Anomala	Galatheidae	Galatheidae	*Galathea*	*dispersa*	zoea	extant
gal_038	Anomala	Galatheidae	Galatheidae	*Galathea*	*dispersa*	zoea	extant
gal_039	Anomala	Galatheidae	Galatheidae	*Sadayoshia*	*edwardsii*	zoea	extant
gal_040	Anomala	Galatheidae	Galatheidae	*Sadayoshia*	*edwardsii*	zoea	extant
gal_041	Anomala	Galatheidae	Galatheidae	*Sadayoshia*	*edwardsii*	zoea	extant
gal_042	Anomala	Galatheidae	Galatheidae	*Sadayoshia*	*edwardsii*	zoea	extant
gal_043	Anomala	Galatheidae	Galatheidae	*Lauriea*	*gardineri*	zoea	extant
gal_044	Anomala	Galatheidae	Galatheidae	*Phylladiorhynchus*	*integrirostris*	zoea	extant
gal_045	Anomala	Galatheidae	Galatheidae	*Allogalathea*	*elegans*	zoea	extant
gal_046	Anomala	Galatheidae	Galatheidae	*Allogalathea*	*elegans*	zoea	extant
gal_048	Anomala	Galatheidae	Galatheidae	*Allogalathea*	*elegans*	zoea	extant
gal_049	Anomala	Galatheidae	Galatheidae	*Agononida*	*incerta*	zoea	extant
gal_050	Anomala	Galatheidae	Galatheidae	*Munida*	*striola*	zoea	extant
gal_051	Anomala	Galatheidae	Galatheidae	*Phylladiorhynchus*	*pusillus*	zoea	extant
gal_052	Anomala	Galatheidae	Galatheidae	*Phylladiorhynchus*	*pusillus*	zoea	extant
gal_053	Anomala	Galatheidae	Galatheidae	*Phylladiorhynchus*	*pusillus*	zoea	extant
gal_054	Anomala	Galatheidae	Galatheidae	*Phylladiorhynchus*	*pusillus*	zoea	extant

no	general_group	major_out	major_group	species_group	species	developmental_phase	status
gal_055	Anomala	Galatheidae	Galatheidae	*Phylladiorhynchus*	*pusillus*	zoea	extant
gal_056	Anomala	Galatheidae	Galatheidae	*Galathea*	*squamifera*	zoea	extant
gal_057	Anomala	Galatheidae	Galatheidae	*Galathea*	*squamifera*	zoea	extant
gal_058	Anomala	Galatheidae	Galatheidae	*Galathea*	*squamifera*	zoea	extant
gal_059	Anomala	Galatheidae	Galatheidae	*Galathea*	*squamifera*	zoea	extant
gal_060	Anomala	Galatheidae	Galatheidae	*Galathea*	*intermedia*	zoea	extant
gal_061	Anomala	Galatheidae	Galatheidae	*Galathea*	*intermedia*	zoea	extant
gal_062	Anomala	Galatheidae	Galatheidae	*Galathea*	*intermedia*	zoea	extant
gal_063	Anomala	Galatheidae	Galatheidae	*Galathea*	*tanegashimae*	adult	extant
gal_064	Anomala	Galatheidae	Galatheidae	*Phylladiorhynchus*	*integrirostris*	adult	extant
gal_065	Anomala	Galatheidae	Galatheidae	*Phylladiorhynchus*	*cf pusillus*	adult	extant
gal_066	Anomala	Galatheidae	Galatheidae	*Allogalathea*	*babai*	adult	extant
gal_067	Anomala	Galatheidae	Galatheidae	*Allogalathea*	*elegans*	adult	extant
gal_068	Anomala	Galatheidae	Galatheidae	*Allogalathea*	*elegans*	adult	extant
gal_069	Anomala	Galatheidae	Galatheidae	*Allogalathea*	*inermis*	adult	extant
gal_070	Anomala	Galatheidae	Galatheidae	*Allogalathea*	*longimana*	adult	extant
gal_071	Anomala	Galatheidae	Galatheidae	*Galathea*	*aegyptica*	adult	extant
gal_072	Anomala	Galatheidae	Galatheidae	*Galathea*	*aegyptica*	adult	extant

no	general_group	major_out	major_group	species_group	species	developmental_phase	status
gal_073	Anomala	Galatheidae	Galatheidae	*Galathea*	*ahyongi*	adult	extant
gal_074	Anomala	Galatheidae	Galatheidae	*Galathea*	*ahyongi*	adult	extant
gal_075	Anomala	Galatheidae	Galatheidae	*Galathea*	sp.	adult	extant
gal_076	Anomala	Galatheidae	Galatheidae	*Galathea*	*aegyptica*	adult	extant
gal_077	Anomala	Galatheidae	Galatheidae	*Galathea*	*ahyongi*	adult	extant
gal_078	Anomala	Galatheidae	Galatheidae	*Galathea*	*aegyptica*	adult	extant
gal_079	Anomala	Galatheidae	Galatheidae	*Galathea*	sp.	adult	extant

no	general_group	major_out	major_group	species_group	species	developmental_phase	status
gal_080	Anomala	Galatheidae	Galatheidae	*Galathea*	sp.	adult	extant
gal_081	Anomala	Galatheidae	Galatheidae	*Galathea*	sp.	adult	extant
gal_082	Anomala	Galatheidae	Galatheidae	*Galathea*	*senta*	adult	extant
gal_083	Anomala	Galatheidae	Galatheidae	*Galathea*	*senta*	adult	extant
gal_084	Anomala	Galatheidae	Galatheidae	*Galathea*	*senta*	adult	extant
gal_085	Anomala	Galatheidae	Galatheidae	*Galathea*	*senta*	adult	extant
gal_086	Anomala	Galatheidae	Galatheidae	*Allogalathea*	*elegans*	adult	extant

no	general_group	major_out	major_group	species_group	species	developmental_phase	status
gal_087	Anomala	Galatheidae	Galatheidae	Lauriea	gardineri	adult	extant
gal_088	Anomala	Galatheidae	Galatheidae	*Alainius*	*crosnieri*	adult	extant
gal_089	Anomala	Galatheidae	Galatheidae	*Alainius*	*crosnieri*	adult	extant
gal_090	Anomala	Galatheidae	Galatheidae	*Alainius*	*crosnieri*	adult	extant
gal_091	Anomala	Galatheidae	Galatheidae	*Alainius*	*crosnieri*	adult	extant
gal_092	Anomala	Galatheidae	Galatheidae	*Allogalathea*	*inermis*	adult	extant
gal_093	Anomala	Galatheidae	Galatheidae	*Allogalathea*	*elegans*	adult	extant
gal_094	Anomala	Galatheidae	Galatheidae	*Allogalathea*	*elegans*	adult	extant

no	general_group	major_out	major_group	species_group	species	developmental_phase	status
gal_095	Anomala	Galatheidae	Galatheidae	*Allogalathea*	*babai*	adult	extant
gal_096	Anomala	Galatheidae	Galatheidae	*Allogalathea*	*inermis*	adult	extant
gal_097	Anomala	Galatheidae	Galatheidae	*Allogalathea*	*elegans*	adult	extant
gal_098	Anomala	Galatheidae	Galatheidae	*Allogalathea*	*inermis*	adult	extant
gal_099	Anomala	Galatheidae	Galatheidae	*Allomunida*	*magnicheles*	adult	extant
gal_100	Anomala	Galatheidae	Galatheidae	*Allomunida*	*magnicheles*	adult	extant
gal_101	Anomala	Galatheidae	Galatheidae	*Coralliogalathea*	sp.	adult	extant
gal_102	Anomala	Galatheidae	Galatheidae	*Coralliogalathea*	*humilis*	adult	extant

no	general_group	major_out	major_group	species_group	species	developmental_phase	status
gal_103	Anomala	Galatheidae	Galatheidae	*Coralliogalathea*	sp.	adult	extant
gal_104	Anomala	Galatheidae	Galatheidae	*Coralliogalathea*	*humilis*	adult	extant
gal_105	Anomala	Galatheidae	Galatheidae	*Coralliogalathea*	*humilis*	adult	extant
gal_106	Anomala	Galatheidae	Galatheidae	*Coralliogalathea*	*humilis*	adult	extant
gal_107	Anomala	Galatheidae	Galatheidae	*Coralliogalathea*	*humilis*	adult	extant
gal_108	Anomala	Galatheidae	Galatheidae	*Coralliogalathea*	*humilis*	adult	extant
gal_109	Anomala	Galatheidae	Galatheidae	*Fennerogalathea*	*cultrata*	adult	extant

no	general_group	major_out	major_group	species_group	species	developmental_phase	status
gal_110	Anomala	Galatheidae	Galatheidae	*Fennerogalathea*	*cultrata*	adult	extant
gal_111	Anomala	Galatheidae	Galatheidae	*Fennerogalathea*	*ensifera*	adult	extant
gal_112	Anomala	Galatheidae	Galatheidae	*Fennerogalathea*	*ensifera*	adult	extant
gal_113	Anomala	Galatheidae	Galatheidae	*Fennerogalathea*	*cultrata*	adult	extant
gal_114	Anomala	Galatheidae	Galatheidae	*Fennerogalathea*	*chani*	adult	extant
gal_115	Anomala	Galatheidae	Galatheidae	*Janetogalathea*	sp.	adult	extant
gal_116	Anomala	Galatheidae	Galatheidae	*Lauriea*	*gardineri*	adult	extant

no	general_group	major_out	major_group	species_group	species	developmental_phase	status
gal_117	Anomala	Galatheidae	Galatheidae	*Lauriea*	*gardineri*	adult	extant
gal_118	Anomala	Galatheidae	Galatheidae	*Lauriea*	*gardineri*	adult	extant
gal_119	Anomala	Galatheidae	Galatheidae	*Lauriea*	*siagiani*	adult	extant
gal_120	Anomala	Galatheidae	Galatheidae	*Lauriea*	*simulata*	adult	extant
gal_121	Anomala	Galatheidae	Galatheidae	*Lauriea*	*simulata*	adult	extant
gal_122	Anomala	Galatheidae	Galatheidae	*Lauriea*	*gardineri*	adult	extant
gal_123	Anomala	Galatheidae	Galatheidae	*Lauriea*	*gardineri*	adult	extant

no	general_group	major_out	major_group	species_group	species	developmental_phase	status
gal_124	Anomala	Galatheidae	Galatheidae	*Lauriea*	*siagiani*	adult	extant
gal_125	Anomala	Galatheidae	Galatheidae	*Lauriea*	*gardineri*	adult	extant
gal_126	Anomala	Galatheidae	Galatheidae	*Lauriea*	*gardineri*	adult	extant
gal_127	Anomala	Galatheidae	Galatheidae	*Lauriea*	*gardineri*	adult	extant
gal_128	Anomala	Galatheidae	Galatheidae	*Lauriea*	*gardineri*	adult	extant
gal_129	Anomala	Galatheidae	Galatheidae	*Lauriea*	*gardineri*	adult	extant
gal_130	Anomala	Galatheidae	Galatheidae	*Lauriea*	*gardineri*	adult	extant
gal_131	Anomala	Galatheidae	Galatheidae	*Lauriea*	*gardineri*	adult	extant
gal_132	Anomala	Galatheidae	Galatheidae	*Lauriea*	*gardineri*	adult	extant

no	general_group	major_out	major_group	species_group	species	developmental_phase	status
gal_133	Anomala	Galatheidae	Galatheidae	*Lauriea*	*gardineri*	adult	extant
gal_134	Anomala	Galatheidae	Galatheidae	*Lauriea*	*gardineri*	adult	extant
gal_135	Anomala	Galatheidae	Galatheidae	*Macrothea*	*bouchardi*	adult	extant
gal_136	Anomala	Galatheidae	Galatheidae	*Macrothea*	*bouchardi*	adult	extant
gal_137	Anomala	Galatheidae	Galatheidae	*Phylladiorhynchus*	sp.	adult	extant
gal_138	Anomala	Galatheidae	Galatheidae	*Phylladiorhynchus*	sp.	adult	extant
gal_139	Anomala	Galatheidae	Galatheidae	*Phylladiorhynchus*	*integrirostris*	adult	extant
gal_140	Anomala	Galatheidae	Galatheidae	*Triodonthea*	*setosa*	adult	extant

no	general_group	major_out	major_group	species_group	species	developmental_phase	status
gal_141	Anomala	Galatheidae	Galatheidae	*Triodonthea*	*setosa*	adult	extant
gal_142	Anomala	Galatheidae	Galatheidae	*Triodonthea*	*setosa*	adult	extant
gal_143	Anomala	Galatheidae	Galatheidae	*Triodonthea*	*setosa*	adult	extant
gal_144	Anomala	Galatheidae	Galatheidae	*Galathea*	*amboinensis*	megalopa	extant
gal_145	Anomala	Galatheidae	Galatheidae	*Galathea*	*inflata*	megalopa	extant
gal_146	Anomala	Galatheidae	Galatheidae	*Galathea*	*intermedia*	megalopa	extant
gal_147	Anomala	Galatheidae	Galatheidae	*Galathea*	*rostrata*	megalopa	extant
gal_148	Anomala	Galatheidae	Galatheidae	*Allogalathea*	*elegans*	megalopa	extant
gal_149	Anomala	Galatheidae	Galatheidae	Phylladiorhynchus	pusillus	megalopa	extant
gal_150	Anomala	Galatheidae	Galatheidae	*Galathea*	*berica*	adult	fossil

no	general_group	major_out	major_group	species_group	species	developmental_phase	status
gal_151	Anomala	Galatheidae	Galatheidae	*Galathea*	*valmaranensis*	adult	fossil
gal_152	Anomala	Galatheidae	Galatheidae	*Galathea*	*cf. weinfurteri*	adult	fossil
gal_153	Anomala	Galatheidae	Galatheidae	Luisogalathea	*tomitai*	adult	fossil
gal_154	Anomala	Galatheidae	Galatheidae	*Galathea*	*sahariana*	adult	fossil
gal_155	Anomala	Galatheidae	Galatheidae	*Galathea*	*acerata*	adult	extant
gal_156	Anomala	Galatheidae	Galatheidae	*Galathea*	*acis*	adult	extant
gal_157	Anomala	Galatheidae	Galatheidae	*Galathea*	*aculeata*	adult	extant
gal_158	Anomala	Galatheidae	Galatheidae	*Galathea*	*aegyptica*	adult	extant
gal_159	Anomala	Galatheidae	Galatheidae	*Galathea*	*aequata*	adult	extant
gal_160	Anomala	Galatheidae	Galatheidae	*Galathea*	*ahyongi*	adult	extant
gal_161	Anomala	Galatheidae	Galatheidae	*Galathea*	*anoplos*	adult	extant
gal_162	Anomala	Galatheidae	Galatheidae	*Galathea*	*anouchkae*	adult	extant
gal_163	Anomala	Galatheidae	Galatheidae	*Galathea*	*argus*	adult	extant
gal_164	Anomala	Galatheidae	Galatheidae	*Galathea*	*atua*	adult	extant
gal_165	Anomala	Galatheidae	Galatheidae	*Galathea*	*australiensis*	adult	extant
gal_166	Anomala	Galatheidae	Galatheidae	*Galathea*	*autahi*	adult	extant
gal_167	Anomala	Galatheidae	Galatheidae	*Galathea*	*barbata*	adult	extant
gal_168	Anomala	Galatheidae	Galatheidae	*Galathea*	*boisselierae*	adult	extant

no	general_group	major_out	major_group	species_group	species	developmental_phase	status
gal_169	Anomala	Galatheidae	Galatheidae	*Galathea*	*boucheti*	adult	extant
gal_170	Anomala	Galatheidae	Galatheidae	*Galathea*	*bracteosa*	adult	extant
gal_171	Anomala	Galatheidae	Galatheidae	*Galathea*	*brevimana*	adult	extant
gal_172	Anomala	Galatheidae	Galatheidae	*Galathea*	*caesariata*	adult	extant
gal_173	Anomala	Galatheidae	Galatheidae	*Galathea*	*celiae*	adult	extant
gal_174	Anomala	Galatheidae	Galatheidae	*Galathea*	*cephyra*	adult	extant
gal_175	Anomala	Galatheidae	Galatheidae	*Galathea*	*ceti*	adult	extant
gal_176	Anomala	Galatheidae	Galatheidae	*Galathea*	*ciliosa*	adult	extant
gal_177	Anomala	Galatheidae	Galatheidae	*Galathea*	*clarki*	adult	extant
gal_178	Anomala	Galatheidae	Galatheidae	*Galathea*	*connudata*	adult	extant
gal_179	Anomala	Galatheidae	Galatheidae	*Galathea*	*consobrina*	adult	extant
gal_180	Anomala	Galatheidae	Galatheidae	*Galathea*	*cornariae*	adult	extant
gal_181	Anomala	Galatheidae	Galatheidae	*Galathea*	*crinita*	adult	extant
gal_182	Anomala	Galatheidae	Galatheidae	*Galathea*	*cymo*	adult	extant
gal_183	Anomala	Galatheidae	Galatheidae	*Galathea*	*cymothoe*	adult	extant
gal_184	Anomala	Galatheidae	Galatheidae	*Galathea*	*eione*	adult	extant
gal_185	Anomala	Galatheidae	Galatheidae	*Galathea*	*eridani*	adult	extant
gal_186	Anomala	Galatheidae	Galatheidae	*Galathea*	*erythrina*	adult	extant

no	general_group	major_out	major_group	species_group	species	developmental_phase	status
gal_187	Anomala	Galatheidae	Galatheidae	*Galathea*	*eucrante*	adult	extant
gal_188	Anomala	Galatheidae	Galatheidae	*Galathea*	*eulimene*	adult	extant
gal_189	Anomala	Galatheidae	Galatheidae	*Galathea*	*eupompe*	adult	extant
gal_190	Anomala	Galatheidae	Galatheidae	*Galathea*	*formosa*	adult	extant
gal_191	Anomala	Galatheidae	Galatheidae	*Galathea*	*furfurea*	adult	extant
gal_192	Anomala	Galatheidae	Galatheidae	*Galathea*	*galene*	adult	extant
gal_193	Anomala	Galatheidae	Galatheidae	*Galathea*	*ganindo*	adult	extant
gal_194	Anomala	Galatheidae	Galatheidae	*Galathea*	*gladiola*	adult	extant
gal_195	Anomala	Galatheidae	Galatheidae	*Galathea*	*gnoma*	adult	extant
gal_196	Anomala	Galatheidae	Galatheidae	*Galathea*	*gruis*	adult	extant
gal_197	Anomala	Galatheidae	Galatheidae	*Galathea*	*halia*	adult	extant
gal_198	Anomala	Galatheidae	Galatheidae	*Galathea*	*hispidissima*	adult	extant
gal_199	Anomala	Galatheidae	Galatheidae	*Galathea*	*homologa*	adult	extant
gal_200	Anomala	Galatheidae	Galatheidae	*Galathea*	*hydrae*	adult	extant
gal_201	Anomala	Galatheidae	Galatheidae	*Galathea*	*imitata*	adult	extant
gal_202	Anomala	Galatheidae	Galatheidae	*Galathea*	*inconspicua*	adult	extant
gal_203	Anomala	Galatheidae	Galatheidae	*Galathea*	*inermis*	adult	extant
gal_204	Anomala	Galatheidae	Galatheidae	*Galathea*	*labidolepta*	adult	extant

no	general_group	major_out	major_group	species_group	species	developmental_phase	status
gal_205	Anomala	Galatheidae	Galatheidae	*Galathea*	*latirostris*	adult	extant
gal_206	Anomala	Galatheidae	Galatheidae	*Galathea*	*lemaitrei*	adult	extant
gal_207	Anomala	Galatheidae	Galatheidae	*Galathea*	*lemniscata*	adult	extant
gal_208	Anomala	Galatheidae	Galatheidae	*Galathea*	*lepidota*	adult	extant
gal_209	Anomala	Galatheidae	Galatheidae	*Galathea*	*leporis*	adult	extant
gal_210	Anomala	Galatheidae	Galatheidae	*Galathea*	*lingadua*	adult	extant
gal_211	Anomala	Galatheidae	Galatheidae	*Galathea*	*longimana*	adult	extant
gal_212	Anomala	Galatheidae	Galatheidae	*Galathea*	*longimanoides*	adult	extant
gal_213	Anomala	Galatheidae	Galatheidae	*Galathea*	*longioculata*	adult	extant
gal_214	Anomala	Galatheidae	Galatheidae	*Galathea*	*machaera*	adult	extant
gal_215	Anomala	Galatheidae	Galatheidae	*Galathea*	*machordomae*	adult	extant
gal_216	Anomala	Galatheidae	Galatheidae	*Galathea*	*magnifica*	adult	extant
Gra_001	Brachyura	Grapsidae	Grapsidae	*Leptograpsus*	*variegatus*	adult	extant?
Gra_002	Brachyura	Grapsidae	Grapsidae	*Leptograpsus*	*variegatus*	adult	extant?
Gra_003	Brachyura	Grapsidae	Grapsidae	*Goniopsis*	*cruentata*	adult	extant?
Gra_004	Brachyura	Grapsidae	Grapsidae	*Goniopsis*	*cruentata*	adult	extant?
Gra_005	Brachyura	Grapsidae	Grapsidae	*Goniopsis*	*pelii*	adult	extant?
Gra_006	Brachyura	Grapsidae	Grapsidae	*Goniopsis*	*pelii*	adult	extant?
Gra_007	Brachyura	Grapsidae	Grapsidae	*Goniopsis*	*pelii*	adult	extant?
Gra_008	Brachyura	Grapsidae	Grapsidae	*Goniopsis*	*pelii*	adult	extant?
Gra_009	Brachyura	Grapsidae	Grapsidae	*Planes*	*marinus*	adult	extant?

no	general_group	major_out	major_group	species_group	species	developmental_phase	status
Gra_010	Brachyura	Grapsidae	Grapsidae	*Planes*	*minutus*	adult	extant?
Gra_011	Brachyura	Grapsidae	Grapsidae	*Planes*	*minutus*	adult	extant?
Gra_012	Brachyura	Grapsidae	Grapsidae	*Planes*	*marinus*	adult	extant?
Gra_016	Brachyura	Grapsidae	Grapsidae	*Planes*	*minutus*	adult	extant?
Gra_017	Brachyura	Grapsidae	Grapsidae	*Planes*	*marinus*	adult	extant?
Gra_018	Brachyura	Grapsidae	Grapsidae	*Geograpsus*	*grayi*	adult	extant?
Gra_019	Brachyura	Grapsidae	Grapsidae	*Metopograpsus*	*thukuhar*	adult	extant?
Gra_020	Brachyura	Grapsidae	Grapsidae	*Pachygrapsus*	*minutus*	adult	extant?
Gra_021	Brachyura	Grapsidae	Grapsidae	*Pachygrapsus*	*plicatus*	adult	extant?
Gra_022	Brachyura	Grapsidae	Grapsidae	*Pachygrapsus*	*socius*	adult	extant?
Gra_023	Brachyura	Grapsidae	Grapsidae	*Planes*	*marinus*	adult	extant?
Gra_024	Brachyura	Grapsidae	Grapsidae	*Planes*	*minutus*	adult	extant?
Gra_026	Brachyura	Grapsidae	Grapsidae	*Planes*	*marinus*	adult	extant?
Gra_038	Brachyura	Grapsidae	Grapsidae	*Geograpsus*	*crinipes*	adult	extant?
Gra_039	Brachyura	Grapsidae	Grapsidae	*Metopograpsus*	*quadridentatus*	adult	extant?
Gra_040	Brachyura	Grapsidae	Grapsidae	*Metopograpsus*	*latifrons*	adult	extant?
Gra_041	Brachyura	Grapsidae	Grapsidae	*Geograpsus*	*crinipes*	adult	extant?
Gra_042	Brachyura	Grapsidae	Grapsidae	*Geograpsus*	*grayi*	adult	extant?
Gra_043	Brachyura	Grapsidae	Grapsidae	*Grapsus*	*tenuicrustatus*	adult	extant?
Gra_044	Brachyura	Grapsidae	Grapsidae	*Pachygrapsus*	*plicatus*	adult	extant?
Gra_045	Brachyura	Grapsidae	Grapsidae	*Geograpsus*	*crinipes*	adult	extant?
Gra_046	Brachyura	Grapsidae	Grapsidae	*Geograpsus*	*grayi*	adult	extant?
Gra_047	Brachyura	Grapsidae	Grapsidae	*Grapsus*	*fourmanoiri*	adult	extant?
Gra_048	Brachyura	Grapsidae	Grapsidae	*Grapsus*	*tenuicrustatus*	adult	extant?
Gra_049	Brachyura	Grapsidae	Grapsidae	*Metopograpsus*	*thukuhar*	adult	extant?
Gra_050	Brachyura	Grapsidae	Grapsidae	*Metopograpsus*	*thukuhar*	adult	extant?
Gra_051	Brachyura	Grapsidae	Grapsidae	*Pachygrapsus*	*minutus*	adult	extant?
Gra_052	Brachyura	Grapsidae	Grapsidae	*Pachygrapsus*	*planifrons*	adult	extant?
Gra_053	Brachyura	Grapsidae	Grapsidae	*Pachygrapsus*	*plicatus*	adult	extant?
Gra_054	Brachyura	Grapsidae	Grapsidae	*Geograpsus*	*crinipes*	juvenile	extant?

no	general_group	major_out	major_group	species_group	species	developmental_phase	status
Gra_055	Brachyura	Grapsidae	Grapsidae	*Geograpsus*	*crinipes*	adult	extant?
Gra_056	Brachyura	Grapsidae	Grapsidae	*Geograpsus*	*grayi*	adult	extant?
Gra_057	Brachyura	Grapsidae	Grapsidae	*Geograpsus*	*stormi*	adult	extant?
Gra_058	Brachyura	Grapsidae	Grapsidae	*Grapsus*	*albolineatus*	adult	extant?
Gra_059	Brachyura	Grapsidae	Grapsidae	*Grapsus*	*tenuicrustatus*	adult	extant?
Gra_060	Brachyura	Grapsidae	Grapsidae	*Grapsus*	*longitarsis*	adult	extant?
Gra_061	Brachyura	Grapsidae	Grapsidae	*Pachygrapsus*	*plicatus*	adult	extant?
Gra_062	Brachyura	Grapsidae	Grapsidae	*Grapsus*	*tenuicrustatus*	adult	extant?
Gra_063	Brachyura	Grapsidae	Grapsidae	*Grapsus*	*fourmanoiri*	adult	extant?
Gra_064	Brachyura	Grapsidae	Grapsidae	*Metopograpsus*	*thukuhar*	adult	extant?
Gra_065	Brachyura	Grapsidae	Grapsidae	*Grapsus*	*longitarsis*	adult	extant?
Gra_066	Brachyura	Grapsidae	Grapsidae	*Grapsus*	*tenuicrustatus*	adult	extant?
Gra_067	Brachyura	Grapsidae	Grapsidae	*Grapsus*	*longitarsis*	adult	extant?
Gra_068	Brachyura	Grapsidae	Grapsidae	*Grapsus*	*albolineatus*	adult	extant?
Gra_069	Brachyura	Grapsidae	Grapsidae	*Grapsus*	*intermedius*	adult	extant?
Gra_070	Brachyura	Grapsidae	Grapsidae	*Geograpsus*	*crinipes*	adult	extant?
Gra_072	Brachyura	Grapsidae	Grapsidae	*Geograpsus*	*lividus*	adult	extant?
Gra_073	Brachyura	Grapsidae	Grapsidae	*Metopograpsus*	*messor*	adult	extant?
Gra_074	Brachyura	Grapsidae	Grapsidae	*Metopograpsus*	*latifrons*	adult	extant?
Gra_075	Brachyura	Grapsidae	Grapsidae	*Metopograpsus*	*frontalis*	adult	extant?
Gra_076	Brachyura	Grapsidae	Grapsidae	*Metopograpsus*	*oceanicus*	adult	extant?
Gra_077	Brachyura	Grapsidae	Grapsidae	*Metopograpsus*	*quadridentatus*	adult	extant?
Gra_078	Brachyura	Grapsidae	Grapsidae	*Geograpsus*	*grayi*	adult	extant?
Gra_079	Brachyura	Grapsidae	Grapsidae	*Grapsus*	*longitarsis*	adult	extant?
Gra_080	Brachyura	Grapsidae	Grapsidae	*Grapsus*	*granulosus*	adult	extant?
Gra_081	Brachyura	Grapsidae	Grapsidae	*Grapsus*	*albolineatus*	adult	extant?
Gra_082	Brachyura	Grapsidae	Grapsidae	*Grapsus*	*longitarsis*	adult	extant?
Gra_083	Brachyura	Grapsidae	Grapsidae	*Pachygrapsus*	*fakaravensis*	adult	extant?
Gra_090	Brachyura	Grapsidae	Grapsidae	*Metopograpsus*	*latifrons*	megalopa	extant?
Gra_092	Brachyura	Grapsidae	Grapsidae	*Metopograpsus*	*oceanicus*	adult	extant?
Gra_093	Brachyura	Grapsidae	Grapsidae	*Metopograpsus*	*oceanicus*	adult	extant?

no	general_group	major_out	major_group	species_group	species	developmental_phase	status
Gra_094	Brachyura	Grapsidae	Grapsidae	*Metopograpsus*	*thukuhar*	adult	extant?
Gra_100	Brachyura	Grapsidae	Grapsidae	*Metopograpsus*	*latifrons*	megalopa	extant?
Gra_101	Brachyura	Grapsidae	Grapsidae	*Metopograpsus*	*cannicci*	adult	extant?
Gra_102	Brachyura	Grapsidae	Grapsidae	*Metopograpsus*	*cannicci*	adult	extant?
Gra_103	Brachyura	Grapsidae	Grapsidae	*Metopograpsus*	*thukuhar*	adult	extant?
Gra_104	Brachyura	Grapsidae	Grapsidae	*Metopograpsus*	*thukuhar*	adult	extant?
Gra_105	Brachyura	Grapsidae	Grapsidae	*Metopograpsus*	*thukuhar*	adult	extant?
Gra_106	Brachyura	Grapsidae	Grapsidae	*Metopograpsus*	*thukuhar*	adult	extant?
Gra_107	Brachyura	Grapsidae	Grapsidae	*Metopograpsus*	*thukuhar*	adult	extant?
Gra_108	Brachyura	Grapsidae	Grapsidae	*Metopograpsus*	*thukuhar*	adult	extant?
Gra_109	Brachyura	Grapsidae	Grapsidae	*Metopograpsus*	*thukuhar*	adult	extant?
Gra_110	Brachyura	Grapsidae	Grapsidae	*Pachygrapsus*	*crassipes*	adult	extant?
Gra_111	Brachyura	Grapsidae	Grapsidae	*Metopograpsus*	*thukuhar*	adult	extant?
Gra_112	Brachyura	Grapsidae	Grapsidae	*Metopograpsus*	*messor*	adult	extant?
Gra_113	Brachyura	Grapsidae	Grapsidae	*Metopograpsus*	*latifrons*	adult	extant?
Gra_114	Brachyura	Grapsidae	Grapsidae	*Metopograpsus*	*messor*	adult	extant?
Gra_115	Brachyura	Grapsidae	Grapsidae	*Metopograpsus*	*latifrons*	adult	extant?
Gra_116	Brachyura	Grapsidae	Grapsidae	*Metopograpsus*	*messor*	adult	extant?
Gra_117	Brachyura	Grapsidae	Grapsidae	*Metopograpsus*	*thukuhar*	adult	extant?
Gra_122	Brachyura	Grapsidae	Grapsidae	*Goniopsis*	*cruentata*	adult	extant?
Gra_123	Brachyura	Grapsidae	Grapsidae	*Grapsus*	*albolineatus*	adult	extant?
Gra_124	Brachyura	Grapsidae	Grapsidae	*Metopograpsus*	*frontalis*	adult	extant?
Gra_125	Brachyura	Grapsidae	Grapsidae	*Metopograpsus*	*messor*	adult	extant?
Gra_126	Brachyura	Grapsidae	Grapsidae	*Metopograpsus*	*oceanicus*	adult	extant?
Gra_127	Brachyura	Grapsidae	Grapsidae	*Pachygrapsus*	*marmoratus*	megalopa	extant?
Gra_128	Brachyura	Grapsidae	Grapsidae	*Pachygrapsus*	*marmoratus*	juvenile	extant?
Gra_129	Brachyura	Grapsidae	Grapsidae	*Pachygrapsus*	*marmoratus*	juvenile	extant?

no	general_group	major_out	major_group	species_group	species	developmental_phase	status
Gra_130	Brachyura	Grapsidae	Grapsidae	*Pachygrapsus*	*marmoratus*	juvenile	extant?
Gra_131	Brachyura	Grapsidae	Grapsidae	*Pachygrapsus*	*marmoratus*	juvenile	extant?
Gra_132	Brachyura	Grapsidae	Grapsidae	*Pachygrapsus*	*marmoratus*	juvenile	extant?
Gra_133	Brachyura	Grapsidae	Grapsidae	*Metopograpsus*	*thukuhar*	zoea	extant?
Gra_134	Brachyura	Grapsidae	Grapsidae	*Metopograpsus*	*latifrons*	adult	extant?
Gra_135	Brachyura	Grapsidae	Grapsidae	*Metopograpsus*	*latifrons*	adult	extant?
Gra_136	Brachyura	Grapsidae	Grapsidae	*Metopograpsus*	*messor*	adult	extant?
Gra_137	Brachyura	Grapsidae	Grapsidae	*Metopograpsus*	*thukuhar*	adult	extant?
Gra_138	Brachyura	Grapsidae	Grapsidae	*Grapsus*	*albolineatus*	adult	extant?
Gra_139	Brachyura	Grapsidae	Grapsidae	*Grapsus*	*granulosus*	adult	extant?
Gra_140	Brachyura	Grapsidae	Grapsidae	*Metopograpsus*	*messor*	adult	extant?
Gra_141	Brachyura	Grapsidae	Grapsidae	*Metopograpsus*	*thukuhar*	adult	extant?
Gra_144	Brachyura	Grapsidae	Grapsidae	*Grapsus*	*tenuicrustatus*	adult	extant?
Gra_145	Brachyura	Grapsidae	Grapsidae	*Grapsus*	*albolineatus*	adult	extant?
Gra_146	Brachyura	Grapsidae	Grapsidae	*Grapsus*	*granulosus*	adult	extant?
Gra_147	Brachyura	Grapsidae	Grapsidae	*Grapsus*	*intermedius*	adult	extant?
Gra_148	Brachyura	Grapsidae	Grapsidae	*Grapsus*	*fourmanoiri*	adult	extant?
Gra_149	Brachyura	Grapsidae	Grapsidae	*Pachygrapsus*	*transversus*	adult	extant?
Gra_151	Brachyura	Grapsidae	Grapsidae	*Geograpsus*	*lividus*	adult	extant?
Gra_152	Brachyura	Grapsidae	Grapsidae	*Goniopsis*	*pulchra*	adult	extant?
Gra_154	Brachyura	Grapsidae	Grapsidae	*Goniopsis*	*cruentata*	adult	extant?
Gra_155	Brachyura	Grapsidae	Grapsidae	*Grapsus*	*grapsus*	adult	extant?
Gra_156	Brachyura	Grapsidae	Grapsidae	*Grapsus*	*grapsus*	adult	extant?
Gra_157	Brachyura	Grapsidae	Grapsidae	*Grapsus*	*grapsus*	adult	extant?
Gra_158	Brachyura	Grapsidae	Grapsidae	*Grapsus*	*adscensionis*	adult	extant?
Gra_159	Brachyura	Grapsidae	Grapsidae	*Grapsus*	*adscensionis*	adult	extant?
Gra_162	Brachyura	Grapsidae	Grapsidae	*Pachygrapsus*	*marmoratus*	adult	extant?

no	general_group	major_out	major_group	species_group	species	developmental_phase	status
Gra_163	Brachyura	Grapsidae	Grapsidae	*Pachygrapsus*	*transversus*	megalopa	extant?
Gra_170	Brachyura	Grapsidae	Grapsidae	*Pachygrapsus*	*marmoratus*	adult	extant?
Gra_171	Brachyura	Grapsidae	Grapsidae	*Pachygrapsus*	*transversus*	adult	extant?
Gra_172	Brachyura	Grapsidae	Grapsidae	*Pachygrapsus*	*transversus*	adult	extant?
Gra_173	Brachyura	Grapsidae	Grapsidae	*Pachygrapsus*	*maurus*	adult	extant?
Gra_175	Brachyura	Grapsidae	Grapsidae	*Pachygrapsus*	*gracilis*	megalopa	extant?
Gra_176	Brachyura	Grapsidae	Grapsidae	*Pachygrapsus*	*gracilis*	juvenile	extant?
Gra_177	Brachyura	Grapsidae	Grapsidae	*Pachygrapsus*	*gracilis*	juvenile	extant?
Gra_178	Brachyura	Grapsidae	Grapsidae	*Pachygrapsus*	*gracilis*	juvenile	extant?
Gra_179	Brachyura	Grapsidae	Grapsidae	*Pachygrapsus*	*gracilis*	juvenile	extant?
Gra_180	Brachyura	Grapsidae	Grapsidae	*Pachygrapsus*	*gracilis*	juvenile	extant?
Gra_181	Brachyura	Grapsidae	Grapsidae	*Pachygrapsus*	*gracilis*	juvenile	extant?
Gra_182	Brachyura	Grapsidae	Grapsidae	*Pachygrapsus*	*gracilis*	juvenile	extant?
Gra_183	Brachyura	Grapsidae	Grapsidae	*Pachygrapsus*	*transversus*	adult	extant?
Gra_184	Brachyura	Grapsidae	Grapsidae	*Pachygrapsus*	*gracilis*	megalopa	extant?
Gra_185	Brachyura	Grapsidae	Grapsidae	*Goniopsis*	*cruentata*	adult	extant?
Gra_186	Brachyura	Grapsidae	Grapsidae	*Pachygrapsus*	*gracilis*	adult	extant?
Gra_187	Brachyura	Grapsidae	Grapsidae	*Planes*	*minutus*	adult	extant?
Gra_188	Brachyura	Grapsidae	Grapsidae	*Pachygrapsus*	*gracilis*	adult	extant?
Gra_189	Brachyura	Grapsidae	Grapsidae	*Geograpsus*	*lividus*	adult	extant?
Gra_190	Brachyura	Grapsidae	Grapsidae	*Goniopsis*	*cruentata*	adult	extant?
Gra_191	Brachyura	Grapsidae	Grapsidae	*Grapsus*	*grapsus*	adult	extant?
Gra_192	Brachyura	Grapsidae	Grapsidae	*Pachygrapsus*	*gracilis*	adult	extant?
Gra_193	Brachyura	Grapsidae	Grapsidae	*Pachygrapsus*	*minutus*	adult	extant?

no	general_group	major_out	major_group	species_group	species	developmental_phase	status
Gra_194	Brachyura	Grapsidae	Grapsidae	*Pachygrapsus*	*loveridgei*	adult	extant?
Gra_195	Brachyura	Grapsidae	Grapsidae	*Pachygrapsus*	*corrugatus*	adult	extant?
Gra_196	Brachyura	Grapsidae	Grapsidae	*Pachygrapsus*	*propinquus*	adult	extant?
Gra_197	Brachyura	Grapsidae	Grapsidae	*Planes*	*minutus*	adult	extant?
Gra_198	Brachyura	Grapsidae	Grapsidae	*Planes*	*marinus*	adult	extant?
Gra_199	Brachyura	Grapsidae	Grapsidae	*Pachygrapsus*	*crassipes*	adult	extant?
Gra_207	Brachyura	Grapsidae	Grapsidae	*Pachygrapsus*	*marmoratus*	zoea	extant?
Gra_208	Brachyura	Grapsidae	Grapsidae	*Sesarma*	*catenata*	zoea	extant?
Gra_209	Brachyura	Grapsidae	Grapsidae	*Sesarma*	*catenata*	zoea	extant?
Gra_210	Brachyura	Grapsidae	Grapsidae	*Sesarma*	*catenata*	zoea	extant?
Gra_211	Brachyura	Grapsidae	Grapsidae	*Sesarma*	*catenata*	zoea	extant?
Gra_213	Brachyura	Grapsidae	Grapsidae	*Cyclograpsus*	*integer*	zoea	extant?

no	general_group	major_out	major_group	species_group	species	developmental_phase	status
Gra_214	Brachyura	Grapsidae	Grapsidae	*Chasmagnathus*	*granulata*	zoea	extant?
Gra_215	Brachyura	Grapsidae	Grapsidae	*Chasmagnathus*	*granulata*	zoea	extant?
Gra_216	Brachyura	Grapsidae	Grapsidae	*Chasmagnathus*	*granulata*	zoea	extant?
Gra_217	Brachyura	Grapsidae	Grapsidae	*Hemigrapsus*	*sanguineus*	zoea	extant?
Gra_218	Brachyura	Grapsidae	Grapsidae	*Geograpsus*	*lividus*	megalopa	extant
Hi_001	Anomala	Hippoidea	Hippidae	Emerita	analoga	adult	extant
Hi_002	Anomala	Hippoidea	Hippidae	*Emerita*	*analoga*	zoea	extant
Hi_003	Anomala	Hippoidea	Hippidae	*Emerita*	*brasiliensis*	adult	extant
Hi_004	Anomala	Hippoidea	Hippidae	*Emerita*	*emiritus*	adult	extant
Hi_005	Anomala	Hippoidea	Hippidae	*Emerita*	*holthuisi*	adult	extant
Hi_006	Anomala	Hippoidea	Hippidae	*Emerita*	*holthuisi*	megalopa	extant
Hi_007	Anomala	Hippoidea	Hippidae	*Emerita*	*holthuisi*	zoea	extant
Hi_008	Anomala	Hippoidea	Hippidae	*Emerita*	*holthuisi*	zoea	extant
Hi_009	Anomala	Hippoidea	Hippidae	*Emerita*	*holthuisi*	zoea	extant
Hi_010	Anomala	Hippoidea	Hippidae	*Emerita*	*holthuisi*	zoea	extant
Hi_011	Anomala	Hippoidea	Hippidae	*Emerita*	*holthuisi*	zoea	extant
Hi_012	Anomala	Hippoidea	Hippidae	*Emerita*	*holthuisi*	zoea	extant
Hi_013	Anomala	Hippoidea	Hippidae	*Emerita*	*portoricensis*	adult	extant

no	general_group	major_out	major_group	species_group	species	developmental_phase	status
Hi_014	Anomala	Hippoidea	Hippidae	*Emerita*	*rathbunae*	megalopa	extant
Hi_015	Anomala	Hippoidea	Hippidae	*Emerita*	*rathbunae*	zoea	extant
Hi_016	Anomala	Hippoidea	Hippidae	*Emerita*	*rathbunae*	zoea	extant
Hi_017	Anomala	Hippoidea	Hippidae	*Emerita*	*sp.*	megalopa	extant
Hi_018	Anomala	Hippoidea	Hippidae	*Emerita*	*sp.*	zoea	extant
Hi_019	Anomala	Hippoidea	Hippidae	*Emerita*	*sp.*	zoea	extant
Hi_020	Anomala	Hippoidea	Hippidae	*Emerita*	*sp.*	zoea	extant
Hi_021	Anomala	Hippoidea	Hippidae	*Emerita*	*sp.*	zoea	extant
Hi_022	Anomala	Hippoidea	Hippidae	*Emerita*	*sp.*	zoea	extant
Hi_023	Anomala	Hippoidea	Hippidae	*Emerita*	*sp.*	zoea	extant
Hi_024	Anomala	Hippoidea	Hippidae	*Emerita*	*taiwanensis*	adult	extant
Hi_025	Anomala	Hippoidea	Hippidae	*Emerita*	*talpoida*	adult	extant
Hi_026	Anomala	Hippoidea	Hippidae	*Hippa*	*adactyla*	adult	extant
Hi_027	Anomala	Hippoidea	Hippidae	*Hippa*	*granulatus*	adult	extant
Hi_028	Anomala	Hippoidea	Hippidae	*Hippa*	*indica*	adult	extant
Hi_029	Anomala	Hippoidea	Hippidae	*Hippa*	*marmorata*	adult	extant
Hi_030	Anomala	Hippoidea	Hippidae	*Hippa*	*ovalis*	adult	extant
Hi_031	Anomala	Hippoidea	Hippidae	*Hippa*	*strigillata*	adult	extant
Hi_032	Anomala	Hippoidea	Hippidae	*Hippa*	*truncatifrons*	adult	extant
Hi_033	Anomala	Hippoidea	Hippidae	*Hippa*	*truncatifrons*	megalopa	extant
Hi_034	Anomala	Hippoidea	Hippidae	*Mastigochirus*	*gracilis*	adult	extant
Hi_035	Anomala	Hippoidea	Hippidae	*Mastigochirus*	*quadrilobatus*	adult	extant

no	general_group	major_out	major_group	species_group	species	developmental_phase	status
Hi_036	Anomala	Hippoidea	Hippidae	Unknown	Unknown	zoea	extant
Hi_037	Anomala	Hippoidea	Hippidae	Unknown	Unknown	zoea	extant
Hi_038	Anomala	Hippoidea	Hippidae	Unknown	Unknown	zoea	extant
Hi_039	Anomala	Hippoidea	Hippidae	Unknown	Unknown	zoea	extant
Hi_040	Anomala	Hippoidea	Hippidae	Unknown	Unknown	zoea	extant
Hi_041	Anomala	Hippoidea	Hippidae	Unknown	Unknown	zoea	extant
Hi_042	Anomala	Hippoidea	Hippidae	Unknown	Unknown	zoea	extant
Hi_043	Anomala	Hippoidea	Hippidae	Unknown	Unknown	zoea	extant
Hi_044	Anomala	Hippoidea	Hippidae	Unknown	Unknown	zoea	extant
Hi_045	Anomala	Hippoidea	Hippidae	Unknown	Unknown	zoea	extant
Hi_046	Anomala	Hippoidea	Hippidae	Unknown	Unknown	zoea	extant
Hi_047	Anomala	Hippoidea	Hippidae	Unknown	Unknown	zoea	extant
Hi_048	Anomala	Hippoidea	Hippidae	Unknown	Unknown	zoea	extant
Hi_049	Anomala	Hippoidea	Hippidae	Unknown	Unknown	zoea	extant
Hi_050	Anomala	Hippoidea	Hippidae	Unknown	Unknown	zoea	extant
Hi_051	Anomala	Hippoidea	Albuneidae	*Albunea*	*carabus*	adult	extant
Hi_052	Anomala	Hippoidea	Albuneidae	*Albunea*	*carabus*	zoea	extant
Hi_053	Anomala	Hippoidea	Albuneidae	*Albunea*	*carabus*	zoea	extant

no	general_group	major_out	major_group	species_group	species	developmental_phase	status
Hi_054	Anomala	Hippoidea	Albuneidae	*Albunea*	*carabus*	zoea	extant
Hi_055	Anomala	Hippoidea	Albuneidae	*Albunea*	*elioti*	adult	extant
Hi_056	Anomala	Hippoidea	Albuneidae	*Albunea*	*occulta*	adult	extant
Hi_057	Anomala	Hippoidea	Albuneidae	*Albunea*	*symmysta*	adult	extant
Hi_058	Anomala	Hippoidea	Albuneidae	*Austrolepidopa*	*schmitti*	adult	extant
Hi_059	Anomala	Hippoidea	Albuneidae	*Austrolepidopa*	*trigonops*	adult	extant
Hi_060	Anomala	Hippoidea	Albuneidae	*Lepidopa*	*benedicti*	adult	extant
Hi_061	Anomala	Hippoidea	Albuneidae	*Lepidopa*	*benedicti*	adult	extant
Hi_062	Anomala	Hippoidea	Albuneidae	*Lepidopa*	*benedicti*	adult	extant
Hi_063	Anomala	Hippoidea	Albuneidae	*Lepidopa*	*benedicti*	megalopa	extant
Hi_064	Anomala	Hippoidea	Albuneidae	*Lepidopa*	*benedicti*	zoea	extant
Hi_065	Anomala	Hippoidea	Albuneidae	*Lepidopa*	*benedicti*	zoea	extant
Hi_066	Anomala	Hippoidea	Albuneidae	*Lepidopa*	*benedicti*	zoea	extant
Hi_067	Anomala	Hippoidea	Albuneidae	*Lepidopa*	*benedicti*	zoea	extant
Hi_068	Anomala	Hippoidea	Albuneidae	*Lepidopa*	*websteri*	adult	extant
Hi_069	Anomala	Hippoidea	Albuneidae	*Paraleucolepidopa*	*myops*	adult	extant
Hi_070	Anomala	Hippoidea	Albuneidae	*Paraleucolepidopa*	*myops*	zoea	extant
Hi_071	Anomala	Hippoidea	Albuneidae	*Stemonopa*	*insignis*	adult	extant
Hi_072	Anomala	Hippoidea	Albuneidae	Unknown	Unknown	zoea	extant
Hi_073	Anomala	Hippoidea	Albuneidae	Unknown	Unknown	zoea	extant

no	general_group	major_out	major_group	species_group	species	developmental_phase	status
Hi_074	Anomala	Hippoidea	Albuneidae	Unknown	Unknown	zoea	extant
Hi_075	Anomala	Hippoidea	Blepharipodidae	*Blepharipoda*	*doelloi*	adult	extant
Hi_076	Anomala	Hippoidea	Blepharipodidae	*Blepharipoda*	*doelloi*	adult	extant
Hi_077	Anomala	Hippoidea	Blepharipodidae	*Blepharipoda*	*doelloi*	megalopa	extant
Hi_078	Anomala	Hippoidea	Blepharipodidae	*Blepharipoda*	*liberata*	adult	extant
Hi_079	Anomala	Hippoidea	Blepharipodidae	*Blepharipoda*	*occidentalis*	adult	extant
Hi_080	Anomala	Hippoidea	Blepharipodidae	*Blepharipoda*	*occidentalis*	zoea	extant
Hi_081	Anomala	Hippoidea	Blepharipodidae	*Blepharipoda*	*occidentalis*	zoea	extant
Hi_082	Anomala	Hippoidea	Blepharipodidae	*Blepharipoda*	*spinosa*	adult	extant
Hi_083	Anomala	Hippoidea	Blepharipodidae	*Lophomastix*	*japonica*	adult	extant
Hi_084	Anomala	Hippoidea	Blepharipodidae	*Lophomastix*	*japonica*	megalopa	extant
Hom_101	Astacidea	Homarida	Nephropidae	*Homarus*	*americanus*	adult	extant
Hom_118	Astacidea	Homarida	Nephropidae	*Homarus*	*americanus*	megalopa	extant
Hom_119	Astacidea	Homarida	Nephropidae	*Homarus*	*americanus*	juvenile	extant
Hom_125	Astacidea	Homarida	Nephropidae	*Homarus*	*americanus*	megalopa	extant
Hom_126	Astacidea	Homarida	Nephropidae	*Homarus*	*americanus*	zoea	extant
Hom_143	Astacidea	Homarida	Nephropidae	*Homarus*	*americanus*	adult	extant
Hom_160	Astacidea	Homarida	Nephropidae	*Homarus*	*gammarus*	zoea	extant
Hom_161	Astacidea	Homarida	Nephropidae	*Homarus*	*gammarus*	zoea	extant
Hom_162	Astacidea	Homarida	Nephropidae	*Homarus*	*gammarus*	zoea	extant
Hom_163	Astacidea	Homarida	Nephropidae	*Homarus*	*gammarus*	megalopa	extant
Hom_165	Astacidea	Homarida	Nephropidae	*Homarus*	*gammarus*	adult	extant
Hom_193	Astacidea	Homarida	Nephropidae	*Metanephrops*	*challengeri*	zoea	extant
Hom_194	Astacidea	Homarida	Nephropidae	*Metanephrops*	*challengeri*	adult	extant
Hom_300	Astacidea	Homarida	Nephropidae	*Metanephrops*	*challengeri*	zoea	extant

no	general_group	major_out	major_group	species_group	species	developmental_phase	status
Hom_301	Astacidea	Homarida	Nephropidae	*Nephropsis*	*grandis*	adult	extant
Hom_302	Astacidea	Homarida	Nephropidae	*Nephropsis*	*grandis*	adult	extant
Hom_304	Astacidea	Homarida	Nephropidae	*Nephropsis*	*ensirostris*	adult	extant
Hom_305	Astacidea	Homarida	Nephropidae	*Nephropsis*	*suhmi*	adult	extant
Hom_306	Astacidea	Homarida	Nephropidae	*Nephropsis*	*occidentalis*	adult	extant
Hom_307	Astacidea	Homarida	Nephropidae	*Nephropsis*	*acanthura*	adult	extant
Hom_308	Astacidea	Homarida	Nephropidae	*Nephropsis*	*stewarti*	adult	extant
Hom_309	Astacidea	Homarida	Nephropidae	*Nephropsis*	*carpenteri*	adult	extant
Hom_310	Astacidea	Homarida	Nephropidae	*Nephropsis*	*sulcata*	adult	extant
Hom_311	Astacidea	Homarida	Nephropidae	*Nephropsis*	*atlantica*	adult	extant
Hom_312	Astacidea	Homarida	Nephropidae	*Nephropsis*	*holthuisi*	adult	extant
Hom_313	Astacidea	Homarida	Nephropidae	*Nephropsis*	*serrata*	adult	extant
Hom_314	Astacidea	Homarida	Nephropidae	Metanephrops	neptunus	adult	extant
Hom_315	Astacidea	Homarida	Nephropidae	Metanephrops	challengeri	zoea	extant
Hom_324	Astacidea	Homarida	Nephropidae	Acanthacaris	*tenuimana*	adult	extant
Hom_325	Astacidea	Homarida	Nephropidae	*Dinochelus*	*ausubeli*	adult	extant
Hom_326	Astacidea	Homarida	Nephropidae	*Metanephrops*	*andamanicus*	adult	extant
Hom_40	Astacidea	Homarida	Nephropidae	*Thaumastochelopsis*	*wardi*	adult	extant
Hom_44	Astacidea	Homarida	Nephropidae	*Acanthacaris*	*caeca*	adult	extant
Hom_54	Astacidea	Homarida	Nephropidae	*Nephropsis*	*aculeata*	adult	extant
Hom_78	Astacidea	Homarida	Nephropidae	*Nephropsis*	*stewarti*	adult	extant
Hom_87	Astacidea	Homarida	Nephropidae	*Thymopsis*	*nilenta*	adult	extant
jur_002	Brachyura	Earliest	Earliest	*Rathbunopon*	*obesum*	adult	fossil
jur_003	Brachyura	Earliest	Earliest	*Protuberosa*	*protuberosa*	adult	fossil
jur_004	Brachyura	Earliest	Earliest	*Acareprospon*	*bouvieri*	adult	fossil
jur_005	Brachyura	Earliest	Earliest	*Prosopon*	*tuberosum*	adult	fossil
jur_006	Brachyura	Earliest	Earliest	*Dromiacea*	*Dromiidae*	adult	fossil

no	general_group	major_out	major_group	species_group	species	developmental_phase	status
jur_007	Brachyura	Earliest	Earliest	*Tanidromites*	*muelleri*	adult	fossil
jur_008	Brachyura	Earliest	Earliest	*Prolecythocaris*	*rieberi*	adult	fossil
jur_009	Brachyura	Earliest	Earliest	*Planoprosopon*	*quadratum*	adult	fossil
jur_010	Brachyura	Earliest	Earliest	*Planoprosopon*	*thiedeae*	adult	fossil
jur_011	Brachyura	Earliest	Earliest	*Planoprosopon*	*thiedeae*	adult	fossil
jur_012	Brachyura	Earliest	Earliest	*Tanidromites*	*lithuanicus*	adult	fossil
jur_013	Brachyura	Earliest	Earliest	*Tanidromites*	*richardsoni*	adult	fossil
jur_014	Brachyura	Earliest	Earliest	*Tanidromites*	*insignis*	adult	fossil
jur_015	Brachyura	Earliest	Earliest	*Tanidromites*	insignis	adult	fossil

no	general_group	major_out	major_group	species_group	species	developmental_phase	status
jur_016	Brachyura	Earliest	Earliest	*Tanidromites*	*sculpta*	adult	fossil
jur_017	Brachyura	Earliest	Earliest	*Tanidromites*	*scheffnerae*	adult	fossil
jur_018	Brachyura	Earliest	Earliest	*Eodromites*	*aequilatus*	adult	fossil
jur_019	Brachyura	Earliest	Earliest	*Prolecythocaris*	*hauckei*	adult	fossil
jur_020	Brachyura	Earliest	Earliest	*Prolecythocaris*	*hauckei*	adult	fossil
jur_021	Brachyura	Earliest	Earliest	*Lecythocaris*	*paradoxa*	adult	fossil
jur_022	Brachyura	Earliest	Earliest	*Goniodromites*	*bidentatus*	adult	fossil
jur_023	Brachyura	Earliest	Earliest	*Goniodromites*	*polyodon*	adult	fossil
jur_024	Brachyura	Earliest	Earliest	*Goniodromites*	*dentatus*	adult	fossil
jur_025	Brachyura	Earliest	Earliest	*Goniodromites*	*serratus*	adult	fossil
jur_026	Brachyura	Earliest	Earliest	*Goniodromites*	*aliquantulus*	adult	fossil

no	general_group	major_out	major_group	species_group	species	developmental_phase	status
jur_027	Brachyura	Earliest	Earliest	*Pithonoton*	*marginatum*	adult	fossil
jur_028	Brachyura	Earliest	Earliest	*Pithonoton*	*elongatum*	adult	fossil
jur_029	Brachyura	Earliest	Earliest	*Prosopon*	*hungaricum*	adult	fossil
jur_030	Brachyura	Earliest	Earliest	*Pithonoton*	*moutieri*	adult	fossil
jur_031	Brachyura	Earliest	Earliest	*Pithonoton*	*simplex*	adult	fossil
jur_032	Brachyura	Earliest	Earliest	*Eodromites*	*grandis*	adult	fossil
jur_033	Brachyura	Earliest	Earliest	*Sabellidromites*	*scarabaea*	adult	fossil
jur_034	Brachyura	Earliest	Earliest	*Tanidromites*	*lingulata*	adult	fossil
jur_035	Brachyura	Earliest	Earliest	*Eodromites*	*depressus*	adult	fossil

no	general_group	major_out	major_group	species_group	species	developmental_phase	status
jur_036	Brachyura	Earliest	Earliest	*Eodromites*	*polyphemi*	adult	fossil
jur_037	Brachyura	Earliest	Earliest	*Eoprosopon*	sp.	adult	fossil
jur_038	Brachyura	Earliest	Earliest	Unknown	sp.	megalopa	fossil
jur_039	Brachyura	Earliest	Earliest	*Eocarcinus*	sp.	adult	fossil
Lit_001	Anomala	Lithodidae	Lithodidae	Paralomis	*granulosa*	zoea	extant
Lit_002	Anomala	Lithodidae	Lithodidae	*Paralomis*	*granulosa*	zoea	extant
Lit_003	Anomala	Lithodidae	Lithodidae	*Paralomis*	*granulosa*	megalopa	extant
Lit_004	Anomala	Lithodidae	Lithodidae	*Paralomis*	*granulosa*	zoea	extant
Lit_005	Anomala	Lithodidae	Lithodidae	*Paralomis*	*granulosa*	megalopa	extant
Lit_006	Anomala	Lithodidae	Lithodidae	*Paralomis*	*granulosa*	juvenile	extant
Lit_007	Anomala	Lithodidae	Lithodidae	*Paralomis*	*spinosissima*	zoea	extant
Lit_008	Anomala	Lithodidae	Lithodidae	*Paralomis*	*spinosissima*	zoea	extant
Lit_009	Anomala	Lithodidae	Lithodidae	*Paralomis*	*spinosissima*	megalopa	extant
Lit_010	Anomala	Lithodidae	Lithodidae	*Paralomis*	*spinosissima*	juvenile	extant
Lit_011	Anomala	Lithodidae	Lithodidae	*Cryptolithodes*	*expansus*	zoea	extant
Lit_012	Anomala	Lithodidae	Lithodidae	*Cryptolithodes*	*expansus*	zoea	extant
Lit_013	Anomala	Lithodidae	Lithodidae	*Cryptolithodes*	*expansus*	zoea	extant
Lit_014	Anomala	Lithodidae	Lithodidae	*Cryptolithodes*	*expansus*	zoea	extant
Lit_015	Anomala	Lithodidae	Lithodidae	*Cryptolithodes*	*expansus*	megalopa	extant
Lit_016	Anomala	Lithodidae	Lithodidae	*Rhinolithodes*	*wosnessenskii*	zoea	extant
Lit_017	Anomala	Lithodidae	Lithodidae	*Rhinolithodes*	*wosnessenskii*	zoea	extant
Lit_018	Anomala	Lithodidae	Lithodidae	*Rhinolithodes*	*wosnessenskii*	zoea	extant
Lit_019	Anomala	Lithodidae	Lithodidae	*Rhinolithodes*	*wosnessenskii*	zoea	extant
Lit_020	Anomala	Lithodidae	Lithodidae	*Rhinolithodes*	*wosnessenskii*	megalopa	extant
Lit_021	Anomala	Lithodidae	Lithodidae	*Lopholithodes*	*mandtii*	zoea	extant
Lit_022	Anomala	Lithodidae	Lithodidae	*Cryptolithodes*	*typicus*	zoea	extant
Lit_023	Anomala	Lithodidae	Lithodidae	*Cryptolithodes*	*typicus*	zoea	extant
Lit_024	Anomala	Lithodidae	Lithodidae	*Cryptolithodes*	*typicus*	zoea	extant
Lit_025	Anomala	Lithodidae	Lithodidae	*Cryptolithodes*	*typicus*	zoea	extant

no	general_group	major_out	major_group	species_group	species	developmental_phase	status
Lit_026	Anomala	Lithodidae	Lithodidae	*Cryptolithodes*	*typicus*	megalopa	extant
Lit_027	Anomala	Lithodidae	Lithodidae	*Cryptolithodes*	*typicus*	juvenile	extant
Lit_028	Anomala	Lithodidae	Lithodidae	Acantholithodes	hispidus	zoea	extant
Lit_029	Anomala	Lithodidae	Lithodidae	*Lopholithodes*	*foraminatus*	megalopa	extant
Lit_030	Anomala	Lithodidae	Lithodidae	*Lopholithodes*	*mandtii*	megalopa	extant
Lit_031	Anomala	Lithodidae	Lithodidae	*Lopholithodes*	*mandtii*	juvenile	extant
Lit_032	Anomala	Lithodidae	Lithodidae	*Lithodes*	*santolla*	megalopa	extant
Lit_033	Anomala	Lithodidae	Lithodidae	*Lithodes*	*santolla*	juvenile	extant
Lit_034	Anomala	Lithodidae	Lithodidae	*Lopholithodes*	*foraminatus*	juvenile	extant
Lit_035	Anomala	Lithodidae	Lithodidae	*Lopholithodes*	*foraminatus*	juvenile	extant
Pag_0001	Anomala	Paguroidea	Coenobitidae	*Coenobita*	*compressus*	zoea	extant
Pag_0003	Anomala	Paguroidea	Coenobitidae	*Coenobita*	*compressus*	zoea	extant
Pag_0005	Anomala	Paguroidea	Coenobitidae	*Coenobita*	*compressus*	zoea	extant
Pag_0007	Anomala	Paguroidea	Coenobitidae	*Coenobita*	*compressus*	zoea	extant
Pag_0009	Anomala	Paguroidea	Coenobitidae	*Coenobita*	*compressus*	zoea	extant
Pag_0011	Anomala	Paguroidea	Coenobitidae	*Coenobita*	*compressus*	megalopa	extant
Pag_0014	Anomala	Paguroidea	Coenobitidae	*Coenobita*	*lila*	adult	extant

no	general_group	major_out	major_group	species_group	species	developmental_phase	status
Pag_0018	Anomala	Paguroidea	Coenobitidae	*Coenobita*	*scaevola*	adult	extant
Pag_0019	Anomala	Paguroidea	Coenobitidae	*Coenobita*	*perlatus*	adult	extant
Pag_0020	Anomala	Paguroidea	Coenobitidae	*Birgus*	*latro*	zoea	extant
Pag_0021	Anomala	Paguroidea	Coenobitidae	*Birgus*	*latro*	zoea	extant
Pag_0022	Anomala	Paguroidea	Coenobitidae	*Birgus*	*latro*	zoea	extant
Pag_0023	Anomala	Paguroidea	Coenobitidae	*Birgus*	*latro*	zoea	extant
Pag_0024	Anomala	Paguroidea	Coenobitidae	*Birgus*	*latro*	zoea	extant
Pag_0030	Anomala	Paguroidea	Coenobitidae	*Birgus*	*latro*	adult	extant
Pag_0031	Anomala	Paguroidea	Paguridae	*Pagurus*	*alatus*	zoea	extant
Pag_0032	Anomala	Paguroidea	Paguridae	*Pagurus*	*alatus*	zoea	extant
Pag_0033	Anomala	Paguroidea	Paguridae	*Pagurus*	*alatus*	zoea	extant
Pag_0034	Anomala	Paguroidea	Paguridae	*Pagurus*	*alatus*	zoea	extant
Pag_0040	Anomala	Paguroidea	Paguridae	*Pagurus*	*alatus*	megalopa	extant
Pag_0042	Anomala	Paguroidea	Paguridae	*Pagurus*	*proximus*	zoea	extant
Pag_0044	Anomala	Paguroidea	Paguridae	*Pagurus*	*proximus*	zoea	extant
Pag_0046	Anomala	Paguroidea	Paguridae	*Pagurus*	*proximus*	zoea	extant
Pag_0048	Anomala	Paguroidea	Paguridae	*Pagurus*	*proximus*	zoea	extant
Pag_0050	Anomala	Paguroidea	Paguridae	*Pagurus*	*proximus*	megalopa	extant

no	general_group	major_out	major_group	species_group	species	developmental_phase	status
Pag_0052	Anomala	Paguroidea	Paguridae	*Pagurus*	*pectinatus*	zoea	extant
Pag_0054	Anomala	Paguroidea	Paguridae	*Pagurus*	*pectinatus*	zoea	extant
Pag_0056	Anomala	Paguroidea	Paguridae	*Pagurus*	*pectinatus*	zoea	extant
Pag_0058	Anomala	Paguroidea	Paguridae	*Pagurus*	*pectinatus*	zoea	extant
Pag_0060	Anomala	Paguroidea	Paguridae	*Pagurus*	*pectinatus*	megalopa	extant
Pag_0062	Anomala	Paguroidea	Paguridae	*Discorsopagurus*	*schmitti*	zoea	extant
Pag_0065	Anomala	Paguroidea	Paguridae	*Discorsopagurus*	*schmitti*	zoea	extant
Pag_0067	Anomala	Paguroidea	Paguridae	*Discorsopagurus*	*schmitti*	zoea	extant
Pag_0069	Anomala	Paguroidea	Paguridae	*Discorsopagurus*	*schmitti*	megalopa	extant
Pag_0072	Anomala	Paguroidea	Paguridae	*Discorsopagurus*	*schmitti*	juvenile	extant
Pag_0075	Anomala	Paguroidea	Paguridae	*Pagurus*	*caurinus*	zoea	extant
Pag_0089	Anomala	Paguroidea	Paguridae	*Discorsopagurus*	*schmitti*	adult	extant
Pag_0090	Anomala	Paguroidea	Paguridae	*Pagurus*	*caurinus*	adult	extant
Pag_0091	Anomala	Paguroidea	Paguridae	*Pagurus*	*stevensae*	zoea	extant
Pag_0093	Anomala	Paguroidea	Paguridae	*Pagurus*	*stevensae*	zoea	extant
Pag_0095	Anomala	Paguroidea	Paguridae	*Pagurus*	*stevensae*	zoea	extant
Pag_0097	Anomala	Paguroidea	Paguridae	*Pagurus*	*stevensae*	zoea	extant
Pag_0099	Anomala	Paguroidea	Paguridae	*Pagurus*	*stevensae*	megalopa	extant
Pag_0101	Anomala	Paguroidea	Paguridae	*Pagurus*	*stevensae*	juvenile	extant
Pag_0102	Anomala	Paguroidea	Paguridae	*Pagurus*	*stevensae*	juvenile	extant

no	general_group	major_out	major_group	species_group	species	developmental_phase	status
Pag_0103	Anomala	Paguroidea	Paguridae	*Pagurus*	*stevensae*	juvenile	extant
Pag_0111	Anomala	Paguroidea	Paguridae	*Pagurus*	*vetaultae*	zoea	extant
Pag_0113	Anomala	Paguroidea	Paguridae	*Pagurus*	*vetaultae*	zoea	extant
Pag_0115	Anomala	Paguroidea	Paguridae	*Pagurus*	*vetaultae*	zoea	extant
Pag_0117	Anomala	Paguroidea	Paguridae	*Pagurus*	*vetaultae*	zoea	extant
Pag_0119	Anomala	Paguroidea	Paguridae	*Pagurus*	*vetaultae*	megalopa	extant
Pag_0122	Anomala	Paguroidea	Paguridae	*Pagurus*	*bernhardus*	zoea	extant
Pag_0127	Anomala	Paguroidea	Paguridae	*Pagurus*	*bernhardus*	megalopa	extant
Pag_0129	Anomala	Paguroidea	Paguridae	*Pagurus*	*pubescens*	zoea	extant
Pag_0134	Anomala	Paguroidea	Paguridae	*Pagurus*	*pubescens*	megalopa	extant
Pag_0136	Anomala	Paguroidea	Paguridae	*Pagurus*	*prideauxi*	zoea	extant
Pag_0141	Anomala	Paguroidea	Paguridae	*Pagurus*	*prideauxi*	megalopa	extant
Pag_0143	Anomala	Paguroidea	Paguridae	*Pagurus*	*cuanensis*	zoea	extant
Pag_0148	Anomala	Paguroidea	Paguridae	*Pagurus*	*cuanensis*	megalopa	extant
Pag_0150	Anomala	Paguroidea	Paguridae	*Pagurus*	*sculptimanus*	zoea	extant
Pag_0152	Anomala	Paguroidea	Paguridae	*Anapagurus*	*laevis*	zoea	extant
Pag_0157	Anomala	Paguroidea	Paguridae	*Anapagurus*	*laevis*	megalopa	extant
Pag_0159	Anomala	Paguroidea	Paguridae	*Anapagurus*	*hyndmanni*	zoea	extant
Pag_0164	Anomala	Paguroidea	Paguridae	*Anapagurus*	*hyndmanni*	megalopa	extant
Pag_0166	Anomala	Paguroidea	Paguridae	*Anapagurus*	*chiroacanthus*	zoea	extant
Pag_0171	Anomala	Paguroidea	Paguridae	*Anapagurus*	*chiroacanthus*	megalopa	extant
Pag_0173	Anomala	Paguroidea	Paguridae	*Pagurus*	*maclaughlinae*	zoea	extant
Pag_0174	Anomala	Paguroidea	Paguridae	*Pagurus*	*maclaughlinae*	megalopa	extant
Pag_0181	Anomala	Paguroidea	Paguridae	*Pagurus*	*lanuginosus*	zoea	extant
Pag_0182	Anomala	Paguroidea	Paguridae	*Pagurus*	*lanuginosus*	zoea	extant
Pag_0183	Anomala	Paguroidea	Paguridae	*Pagurus*	*lanuginosus*	zoea	extant
Pag_0184	Anomala	Paguroidea	Paguridae	*Pagurus*	*lanuginosus*	zoea	extant
Pag_0189	Anomala	Paguroidea	Paguridae	*Pagurus*	*lanuginosus*	megalopa	extant
Pag_0193	Anomala	Paguroidea	Paguridae	*Pagurus*	*longicarpus*	zoea	extant

no	general_group	major_out	major_group	species_group	species	developmental_phase	status
Pag_0195	Anomala	Paguroidea	Paguridae	*Pagurus*	*longicarpus*	zoea	extant
Pag_0197	Anomala	Paguroidea	Paguridae	*Pagurus*	*longicarpus*	zoea	extant
Pag_0199	Anomala	Paguroidea	Paguridae	*Pagurus*	*longicarpus*	zoea	extant
Pag_0202	Anomala	Paguroidea	Paguridae	*Pagurus*	*marshi*	zoea	extant
Pag_0203	Anomala	Paguroidea	Paguridae	*Pagurus*	*marshi*	zoea	extant
Pag_0204	Anomala	Paguroidea	Paguridae	*Pagurus*	*marshi*	zoea	extant
Pag_0205	Anomala	Paguroidea	Paguridae	*Pagurus*	*marshi*	zoea	extant
Pag_0219	Anomala	Paguroidea	Paguridae	*Pagurus*	*stevensae*	adult	extant
Pag_0220	Anomala	Paguroidea	Paguridae	*Pagurus*	*pubescens*	adult	extant
Pag_0221	Anomala	Paguroidea	Paguridae	*Pagurus*	*maclaughlinae*	adult	extant
Pag_0222	Anomala	Paguroidea	Paguridae	*Pagurus*	*maclaughlinae*	adult	extant

no	general_group	major_out	major_group	species_group	species	developmental_phase	status
Pag_0223	Anomala	Paguroidea	Paguridae	*Pagurus*	*pollicaris*	adult	extant
Pag_0228	Anomala	Paguroidea	Paguridae	*Pagurus*	*simulans*	zoea	extant
Pag_0230	Anomala	Paguroidea	Paguridae	*Pagurus*	*simulans*	zoea	extant
Pag_0232	Anomala	Paguroidea	Paguridae	*Pagurus*	*simulans*	zoea	extant
Pag_0234	Anomala	Paguroidea	Paguridae	*Pagurus*	*simulans*	zoea	extant
Pag_0236	Anomala	Paguroidea	Paguridae	*Pagurus*	*simulans*	megalopa	extant
Pag_0239	Anomala	Paguroidea	Paguridae	*Pagurus*	*gracilipes*	zoea	extant
Pag_0241	Anomala	Paguroidea	Paguridae	*Pagurus*	*gracilipes*	zoea	extant
Pag_0244	Anomala	Paguroidea	Paguridae	*Pagurus*	*gracilipes*	megalopa	extant
Pag_0246	Anomala	Paguroidea	Paguridae	*Pagurus*	*granosimanus*	zoea	extant
Pag_0247	Anomala	Paguroidea	Paguridae	*Pagurus*	*granosimanus*	zoea	extant
Pag_0248	Anomala	Paguroidea	Paguridae	*Pagurus*	*granosimanus*	zoea	extant
Pag_0249	Anomala	Paguroidea	Paguridae	*Pagurus*	*granosimanus*	zoea	extant
Pag_0254	Anomala	Paguroidea	Paguridae	*Pagurus*	*granosimanus*	megalopa	extant
Pag_0256	Anomala	Paguroidea	Paguridae	*Pagurus*	*hemphilli*	zoea	extant

no	general_group	major_out	major_group	species_group	species	developmental_phase	status
Pag_0257	Anomala	Paguroidea	Paguridae	*Pagurus*	*hemphilli*	zoea	extant
Pag_0259	Anomala	Paguroidea	Paguridae	*Pagurus*	*hemphilli*	zoea	extant
Pag_0264	Anomala	Paguroidea	Paguridae	*Pagurus*	*hemphilli*	megalopa	extant
Pag_0267	Anomala	Paguroidea	Paguridae	*Pagurus*	*hemphilli*	zoea	extant
Pag_0268	Anomala	Paguroidea	Paguridae	*Pagurus*	*hemphilli*	zoea	extant
Pag_0269	Anomala	Paguroidea	Paguridae	*Pagurus*	*hemphilli*	zoea	extant
Pag_0274	Anomala	Paguroidea	Paguridae	*Pagurus*	*hemphilli*	megalopa	extant
Pag_0276	Anomala	Paguroidea	Paguridae	*Pagurus*	*variabilis*	zoea	extant
Pag_0280	Anomala	Paguroidea	Paguridae	*Pagurus*	*prideauxi*	zoea	extant
Pag_0281	Anomala	Paguroidea	Paguridae	*Pagurus*	*prideauxi*	zoea	extant
Pag_0282	Anomala	Paguroidea	Paguridae	*Pagurus*	*prideauxi*	zoea	extant
Pag_0283	Anomala	Paguroidea	Paguridae	*Pagurus*	*prideauxi*	zoea	extant

no	general_group	major_out	major_group	species_group	species	developmental_phase	status
Pag_0290	Anomala	Paguroidea	Paguridae	*Pagurus*	*ochotensis*	zoea	extant
Pag_0291	Anomala	Paguroidea	Paguridae	*Pagurus*	*ochotensis*	zoea	extant
Pag_0292	Anomala	Paguroidea	Paguridae	*Pagurus*	*ochotensis*	zoea	extant
Pag_0293	Anomala	Paguroidea	Paguridae	*Pagurus*	*ochotensis*	zoea	extant
Pag_0294	Anomala	Paguroidea	Paguridae	*Pagurus*	*ochotensis*	megalopa	extant
Pag_0295	Anomala	Paguroidea	Paguridae	*Pagurus*	*ochotensis*	juvenile	extant
Pag_0296	Anomala	Paguroidea	Paguridae	*Pagurus*	*ochotensis*	juvenile	extant
Pag_0306	Anomala	Paguroidea	Paguridae	*Pagurus*	*arcuatus*	zoea	extant
Pag_0308	Anomala	Paguroidea	Paguridae	*Pagurus*	*arcuatus*	zoea	extant

no	general_group	major_out	major_group	species_group	species	developmental_phase	status
Pag_0310	Anomala	Paguroidea	Paguridae	*Pagurus*	*arcuatus*	zoea	extant
Pag_0312	Anomala	Paguroidea	Paguridae	*Pagurus*	*arcuatus*	zoea	extant
Pag_0314	Anomala	Paguroidea	Paguridae	*Pagurus*	*arcuatus*	megalopa	extant
Pag_0316	Anomala	Paguroidea	Paguridae	*Pagurus*	*armatus*	zoea	extant
Pag_0318	Anomala	Paguroidea	Paguridae	*Pagurus*	*armatus*	zoea	extant
Pag_0320	Anomala	Paguroidea	Paguridae	*Pagurus*	*armatus*	zoea	extant
Pag_0322	Anomala	Paguroidea	Paguridae	*Pagurus*	*armatus*	zoea	extant
Pag_0324	Anomala	Paguroidea	Paguridae	*Pagurus*	*armatus*	megalopa	extant
Pag_0332	Anomala	Paguroidea	Paguridae	*Pagurus*	*hirsutiusculus*	zoea	extant
Pag_0333	Anomala	Paguroidea	Paguridae	*Pagurus*	*hirsutiusculus*	zoea	extant
Pag_0334	Anomala	Paguroidea	Paguridae	*Pagurus*	*hirsutiusculus*	zoea	extant
Pag_0335	Anomala	Paguroidea	Paguridae	*Pagurus*	*hirsutiusculus*	zoea	extant

no	general_group	major_out	major_group	species_group	species	developmental_phase	status
Pag_0336	Anomala	Paguroidea	Paguridae	*Pagurus*	*hirsutiusculus*	megalopa	extant
Pag_0337	Anomala	Paguroidea	Paguridae	*Pagurus*	*kennerlyi*	zoea	extant
Pag_0339	Anomala	Paguroidea	Paguridae	*Pagurus*	*kennerlyi*	zoea	extant
Pag_0342	Anomala	Paguroidea	Paguridae	*Pagurus*	*kennerlyi*	zoea	extant
Pag_0348	Anomala	Paguroidea	Paguridae	*Pagurus*	*kennerlyi*	juvenile	extant
Pag_0352	Anomala	Paguroidea	Paguridae	*Pagurus*	*arenisaxatilis*	zoea	extant
Pag_0353	Anomala	Paguroidea	Paguridae	*Pagurus*	*arenisaxatilis*	zoea	extant
Pag_0354	Anomala	Paguroidea	Paguridae	*Pagurus*	*arenisaxatilis*	zoea	extant
Pag_0355	Anomala	Paguroidea	Paguridae	*Pagurus*	*arenisaxatilis*	zoea	extant
Pag_0356	Anomala	Paguroidea	Paguridae	*Pagurus*	*arenisaxatilis*	megalopa	extant
Pag_0388	Anomala	Paguroidea	Paguridae	*Catapaguroides*	*umbra*	adult	extant
Pag_0392	Anomala	Paguroidea	Paguridae	*Enneophyllus*	*spinirostris*	adult	extant
Pag_0394	Anomala	Paguroidea	Paguridae	*Enneopagurus*	*garciagomezi*	adult	extant
Pag_0396	Anomala	Paguroidea	Paguridae	*Decaphyllus*	*barunajaya*	adult	extant
Pag_0398	Anomala	Paguroidea	Paguridae	*Decaphyllus*	*maci*	adult	extant
Pag_0402	Anomala	Paguroidea	Paguridae	*Catapaguroides*	*declivis*	adult	extant
Pag_0405	Anomala	Paguroidea	Paguridae	*Catapaguroides*	*karubar*	adult	extant
Pag_0409	Anomala	Paguroidea	Paguridae	*Solitariopagurus*	*tuerkayi*	adult	extant
Pag_0412	Anomala	Paguroidea	Paguridae	*Porcellanopagurus*	*jacquesi*	adult	extant

no	general_group	major_out	major_group	species_group	species	developmental_phase	status
Pag_0416	Anomala	Paguroidea	Paguridae	*Alainopaguroides*	*lemaitrei*	adult	extant
Pag_0418	Anomala	Paguroidea	Paguridae	*Turleania*	*senticosa*	adult	extant
Pag_0420	Anomala	Paguroidea	Paguridae	*Turleania*	*multispina*	adult	extant
Pag_0424	Anomala	Paguroidea	Paguridae	*Michelopagurus*	*limatulus*	adult	extant
Pag_0426	Anomala	Paguroidea	Paguridae	*Pseudopagurodes*	*piliferus*	adult	extant
Pag_0428	Anomala	Paguroidea	Paguridae	*Michelopagurus*	*chacei*	adult	extant
Pag_0432	Anomala	Paguroidea	Paguridae	*Icelopagurus*	*crosnieri*	adult	extant
Pag_0434	Anomala	Paguroidea	Paguridae	*Tarrasopagurus*	*rostrodenticulatus*	adult	extant
Pag_0438	Anomala	Paguroidea	Paguridae	*Catapagurus*	*oculocrassus*	adult	extant
Pag_0440	Anomala	Paguroidea	Paguridae	*Catapagurus*	*oculocrassus*	adult	extant
Pag_0441	Anomala	Paguroidea	Paguridae	*Catapagurus*	*tanimbarensis*	adult	extant
Pag_0443	Anomala	Paguroidea	Paguridae	*Catapagurus*	*tanimbarensis*	adult	extant
Pag_0445	Anomala	Paguroidea	Paguridae	*Catapagurus*	*holthuisi*	adult	extant
Pag_0446	Anomala	Paguroidea	Paguridae	*Catapagurus*	*holthuisi*	adult	extant
Pag_0448	Anomala	Paguroidea	Paguridae	*Nematopagurus*	*cf. Indicus*	adult	extant
Pag_0450	Anomala	Paguroidea	Paguridae	*Nematopagurus*	*sp.*	adult	extant
Pag_0452	Anomala	Paguroidea	Paguridae	*Nematopagurus*	*spinulosensoris*	adult	extant
Pag_0454	Anomala	Paguroidea	Paguridae	*Nematopagurus*	*scutelliformis*	adult	extant
Pag_0456	Anomala	Paguroidea	Paguridae	*Nematopagurus*	*ostlingochirus*	adult	extant
Pag_0460	Anomala	Paguroidea	Paguridae	*Nematopagurus*	*alcocki*	adult	extant
Pag_0464	Anomala	Paguroidea	Paguridae	*Australeremus*	*triserratus*	adult	extant
Pag_0466	Anomala	Paguroidea	Paguridae	*Australeremus*	*triserratus*	adult	extant
Pag_0468	Anomala	Paguroidea	Paguridae	*Pagurus*	*kaiensis*	adult	extant
Pag_0470	Anomala	Paguroidea	Paguridae	*Pagurus*	*capsularis*	adult	extant
Pag_0474	Anomala	Paguroidea	Paguridae	*Pagurus*	*haigae*	adult	extant
Pag_0476	Anomala	Paguroidea	Paguridae	*Bathypaguropsis*	*rahayuae*	adult	extant
Pag_0478	Anomala	Paguroidea	Paguridae	*Pylopaguropsis*	*zebra*	adult	extant
Pag_0480	Anomala	Paguroidea	Paguridae	*Pylopaguropsis*	*laevispinosa*	adult	extant
Pag_0482	Anomala	Paguroidea	Paguridae	*Tomopaguropsis*	*crinita*	adult	extant

no	general_group	major_out	major_group	species_group	species	developmental_phase	status
Pag_0488	Anomala	Paguroidea	Parapaguridae	Sympagurus	*dimorphus*	megalopa	extant
Pag_0492	Anomala	Paguroidea	Parapaguridae	Parapagurus	*latimanus*	adult	extant
Pag_0495	Anomala	Paguroidea	Parapaguridae	*Parapagurus*	*pilosimanus*	adult	extant
Pag_0499	Anomala	Paguroidea	Parapaguridae	*Parapagurus*	*alominos*	adult	extant
Pag_0500	Anomala	Paguroidea	Parapaguridae	*Parapagurus*	*nudus*	adult	extant
Pag_0501	Anomala	Paguroidea	Parapaguridae	*Parapagurus*	*abyssorum*	adult	extant
Pag_0504	Anomala	Paguroidea	Parapaguridae	*Sympagurus*	*pictus*	adult	extant
Pag_0510	Anomala	Paguroidea	Parapaguridae	*Sympagurus*	*acinops*	adult	extant
Pag_0513	Anomala	Paguroidea	Parapaguridae	*Sympagurus*	*dimorphus*	adult	extant
Pag_0517	Anomala	Paguroidea	Parapaguridae	*Tylapsis*	*anomala*	adult	extant
Pag_0533	Anomala	Paguroidea	Parapaguridae	*Parapagurus*	*andreui*	adult	extant
Pag_0537	Anomala	Paguroidea	Parapaguridae	*Parapagurus*	*bouvieri*	adult	extant
Pag_0541	Anomala	Paguroidea	Parapaguridae	*Strobopagurus*	*gracilipes*	adult	extant
Pag_0550	Anomala	Paguroidea	Parapaguridae	*Oncopagurus*	*glebosus*	adult	extant
Pag_0554	Anomala	Paguroidea	Parapaguridae	*Strobopagurus*	*sibogae*	adult	extant
Pag_0558	Anomala	Paguroidea	Parapaguridae	*Sympagurus*	*brevipes*	adult	extant
Pag_0561	Anomala	Paguroidea	Parapaguridae	*Sympagurus*	*brevipes*	juvenile	extant
Pag_0565	Anomala	Paguroidea	Parapaguridae	*Sympagurus*	*soela*	adult	extant
Pag_0568	Anomala	Paguroidea	Parapaguridae	*Sympagurus*	*villosus*	adult	extant
Pag_0571	Anomala	Paguroidea	Parapaguridae	*Oncopagurus*	*indicus*	adult	extant
Pag_0576	Anomala	Paguroidea	Parapaguridae	*Oncopagurus*	*monstrosus*	adult	extant
Pag_0581	Anomala	Paguroidea	Parapaguridae	*Oncopagurus*	*minutus*	adult	extant
Pag_0585	Anomala	Paguroidea	Parapaguridae	*Oncopagurus*	*cidaris*	adult	extant

no	general_group	major_out	major_group	species_group	species	developmental_phase	status
Pag_0589	Anomala	Paguroidea	Parapaguridae	*Paragiopagurus*	*diogenes*	adult	extant
Pag_0592	Anomala	Paguroidea	Parapaguridae	*Paragiopagurus*	*acutus*	adult	extant
Pag_0596	Anomala	Paguroidea	Parapaguridae	*Paragiopagurus*	*bicarinatus*	adult	extant
Pag_0600	Anomala	Paguroidea	Parapaguridae	*Paragiopagurus*	*hirsutus*	adult	extant
Pag_0604	Anomala	Paguroidea	Calcinidae	*Calcinus*	*vachoni*	adult	extant
Pag_0609	Anomala	Paguroidea	Pylochelidae	*Trizocheles*	*vaubanae*	adult	extant
Pag_0611	Anomala	Paguroidea	Pylochelidae	*Pomatocheles*	*jeffreysii*	adult	extant
Pag_0613	Anomala	Paguroidea	Pylochelidae	*Cheiroplatea*	*laticauda*	adult	extant
Pag_0614	Anomala	Paguroidea	Pylochelidae	*Cheiroplatea*	*mitoi*	adult	extant
Pag_0617	Anomala	Paguroidea	Pylochelidae	*Pomatocheles*	*gaillard*	adult	extant
Pag_0620	Anomala	Paguroidea	Pylochelidae	*Xylocheles*	*miersi*	adult	extant
Pag_0623	Anomala	Paguroidea	Pylochelidae	*Xylocheles*	*macrops*	adult	extant
Pag_0626	Anomala	Paguroidea	Pylochelidae	*Bathycheles*	*indicus*	adult	extant
Pag_0629	Anomala	Paguroidea	Pylochelidae	*Bathycheles*	*integer*	adult	extant
Pag_0632	Anomala	Paguroidea	Pylochelidae	*Bathycheles*	*phenax*	adult	extant
Pag_0635	Anomala	Paguroidea	Pylochelidae	*Parapylocheles*	*scorpio*	adult	extant
Pag_0638	Anomala	Paguroidea	Pylochelidae	*Trizocheles*	*mendanai*	adult	extant
Pag_0641	Anomala	Paguroidea	Pylochelidae	*Trizocheles*	*hoensonae*	adult	extant
Pag_0645	Anomala	Paguroidea	Pylochelidae	*Trizocheles*	*balssi*	adult	extant
Pag_0648	Anomala	Paguroidea	Pylochelidae	*Trizocheles*	*boasi*	adult	extant

no	general_group	major_out	major_group	species_group	species	developmental_phase	status
Pag_0651	Anomala	Paguroidea	Pylojacquesidae	*Pylojacquesia*	*colemani*	adult	extant
Pag_0655	Anomala	Paguroidea	Xylopaguridae	*Xylopagurus*	*tayrona*	adult	extant
Pag_0660	Anomala	Paguroidea	Parapaguridae	*Parapagurus*	*microps*	adult	extant
Pag_0664	Anomala	Paguroidea	Parapaguridae	*Parapagurus*	*benedicti*	adult	extant
Pag_0668	Anomala	Paguroidea	Parapaguridae	*Parapagurus*	*holthuisi*	adult	extant
Pag_0700	Anomala	Paguroidea	Parapaguridae	*Parapagurus*	*diogenes*	zoea	extant
Pag_0711	Anomala	Paguroidea	Parapaguridae	*Parapagurus*	sp.	zoea	extant
Pag_0716	Anomala	Paguroidea	Parapaguridae	*Parapagurus*	sp.	zoea	extant
Pce_001	Anomala	Porcellanidae	Porcellanidae	*Pachycheles*	*susanae*	adult	extant
Pce_002	Anomala	Porcellanidae	Porcellanidae	*Petrolisthes*	*lindae*	adult	extant
Pce_003	Anomala	Porcellanidae	Porcellanidae	*Petrolisthes*	*armatus*	megalopa	extant

no	general_group	major_out	major_group	species_group	species	developmental_phase	status
Pce_004	Anomala	Porcellanidae	Porcellanidae	*Petrolisthes*	*novaezelanidae*	megalopa	extant
Pce_005	Anomala	Porcellanidae	Porcellanidae	*Petrolisthes*	*elongatus*	megalopa	extant
Pce_006	Anomala	Porcellanidae	Porcellanidae	*Petrolisthes*	*tridentatus*	megalopa	extant
Pce_007	Anomala	Porcellanidae	Porcellanidae	*Petrolisthes*	*laevigatus*	megalopa	extant
Pce_008	Anomala	Porcellanidae	Porcellanidae	*Petrolisthes*	*laevigatus*	adult	extant
Pce_009	Anomala	Porcellanidae	Porcellanidae	*Pachycheles*	*stevensii*	megalopa	extant
Pce_010	Anomala	Porcellanidae	Porcellanidae	*Pachycheles*	*stevensii*	zoea	extant

no	general_group	major_out	major_group	species_group	species	developmental_phase	status
Pce_011	Anomala	Porcellanidae	Porcellanidae	*Pachycheles*	*stevensii*	zoea	extant
Pce_012	Anomala	Porcellanidae	Porcellanidae	*Megalobrachium*	*poeyi*	megalopa	extant
Pce_013	Anomala	Porcellanidae	Porcellanidae	*Megalobrachium*	*poeyi*	megalopa	extant
Pce_014	Anomala	Porcellanidae	Porcellanidae	*Petrolisthes*	*violaceus*	megalopa	extant
Pce_015	Anomala	Porcellanidae	Porcellanidae	*Polyonyx*	*quadriungulatus*	adult	extant
Pce_016	Anomala	Porcellanidae	Porcellanidae	*Pachycheles*	*rudis*	adult	extant

no	general_group	major_out	major_group	species_group	species	developmental_phase	status
Pce_017	Anomala	Porcellanidae	Porcellanidae	*Petrolisthes*	*unilobatus*	megalopa	extant
Pce_018	Anomala	Porcellanidae	Porcellanidae	*Neopisosoma*	*angustifrons*	megalopa	extant
Pce_019	Anomala	Porcellanidae	Porcellanidae	*Neopisosoma*	*neglectum*	megalopa	extant
Pce_020	Anomala	Porcellanidae	Porcellanidae	*Porcellana*	*sayana*	megalopa	extant
Pce_021	Anomala	Porcellanidae	Porcellanidae	*Pachycheles*	*pubescens*	megalopa	extant
Pce_022	Anomala	Porcellanidae	Porcellanidae	*Petrolisthes*	*tonsorius*	megalopa	extant
Pce_023	Anomala	Porcellanidae	Porcellanidae	*Megalobrachium*	*roseum*	megalopa	extant

no	general_group	major_out	major_group	species_group	species	developmental_phase	status
Pce_024	Anomala	Porcellanidae	Porcellanidae	*Enosteoides*	*lobatus*	adult	extant
Pce_025	Anomala	Porcellanidae	Porcellanidae	*Lissoporcellana*	*flagellicola*	adult	extant
Pce_026	Anomala	Porcellanidae	Porcellanidae	*Neopetrolisthes*	*maculatus*	adult	extant
Pce_027	Anomala	Porcellanidae	Porcellanidae	*Novorostrum*	*indicum*	adult	extant
Pce_028	Anomala	Porcellanidae	Porcellanidae	*Pachycheles*	*garciaensis*	adult	extant
Pce_029	Anomala	Porcellanidae	Porcellanidae	*Pachycheles*	*pisoides*	adult	extant

no	general_group	major_out	major_group	species_group	species	developmental_phase	status
Pce_030	Anomala	Porcellanidae	Porcellanidae	*Petrolisthes*	*asiaticis*	adult	extant
Pce_031	Anomala	Porcellanidae	Porcellanidae	*Petrolisthes*	*celebesensis*	adult	extant
Pce_032	Anomala	Porcellanidae	Porcellanidae	*Petrolisthes*	*lamarckii*	adult	extant
Pce_033	Anomala	Porcellanidae	Porcellanidae	*Petrolisthes*	*moluccensis*	adult	extant
Pce_034	Anomala	Porcellanidae	Porcellanidae	*Petrolisthes*	*obtusifrons*	adult	extant
Pce_035	Anomala	Porcellanidae	Porcellanidae	*Petrolisthes*	*pubescens*	adult	extant

no	general_group	major_out	major_group	species_group	species	developmental_phase	status
Pce_036	Anomala	Porcellanidae	Porcellanidae	*Petrolisthes*	*tomentosus*	adult	extant
Pce_037	Anomala	Porcellanidae	Porcellanidae	*Petrolisthes*	*virgatus*	adult	extant
Pce_038	Anomala	Porcellanidae	Porcellanidae	*Megalobrachium*	*soriatum*	megalopa	extant
Pce_039	Anomala	Porcellanidae	Porcellanidae	*Petrolisthes*	*ornatus*	megalopa	extant
Pce_040	Anomala	Porcellanidae	Porcellanidae	*Petrolisthes*	*zacae*	megalopa	extant
Pce_041	Anomala	Porcellanidae	Porcellanidae	*Pachycheles*	*serratus*	megalopa	extant
Pce_042	Anomala	Porcellanidae	Porcellanidae	*Petrolisthes*	*robosonae*	megalopa	extant
Pce_043	Anomala	Porcellanidae	Porcellanidae	*Petrolisthes*	*magfalenesis*	megalopa	extant

no	general_group	major_out	major_group	species_group	species	developmental_phase	status
Pce_044	Anomala	Porcellanidae	Porcellanidae	*Megalobrachium*	*mortenseni*	adult	extant
Pce_045	Anomala	Porcellanidae	Porcellanidae	*Megalobrachium*	*poeyi*	adult	extant
Pce_046	Anomala	Porcellanidae	Porcellanidae	*Megalobrachium*	*roseum*	adult	extant
Pce_047	Anomala	Porcellanidae	Porcellanidae	*Pachycheles*	*chacei*	adult	extant
Pce_048	Anomala	Porcellanidae	Porcellanidae	*Pachycheles*	*cristobalensis*	adult	extant
Pce_049	Anomala	Porcellanidae	Porcellanidae	*Pachycheles*	*serratus*	adult	extant

no	general_group	major_out	major_group	species_group	species	developmental_phase	status
Pce_050	Anomala	Porcellanidae	Porcellanidae	*Petrolisthes*	*caribensis*	adult	extant
Pce_051	Anomala	Porcellanidae	Porcellanidae	*Petrolisthes*	*galathinus*	adult	extant
Pce_052	Anomala	Porcellanidae	Porcellanidae	*Petrolisthes*	*jugosus*	adult	extant
Pce_053	Anomala	Porcellanidae	Porcellanidae	*Porcellana*	*cancrisocialis*	megalopa	extant
Pce_054	Anomala	Porcellanidae	Porcellanidae	*Petrolisthes*	*granulosus*	megalopa	extant
Pce_055	Anomala	Porcellanidae	Porcellanidae	*Petrolisthes*	*cinctipes*	megalopa	extant
Pce_056	Anomala	Porcellanidae	Porcellanidae	*Petrolisthes*	*eriomerus*	megalopa	extant
Pce_057	Anomala	Porcellanidae	Porcellanidae	*Pachycheles*	*pubescens*	megalopa	extant
Pce_058	Anomala	Porcellanidae	Porcellanidae	*Pachycheles*	*rudis*	megalopa	extant
Pce_059	Anomala	Porcellanidae	Porcellanidae	*Pachycheles*	*pubescens*	megalopa	extant
Pce_060	Anomala	Porcellanidae	Porcellanidae	*Psidia*	*dehaanii*	megalopa	extant

no	general_group	major_out	major_group	species_group	species	developmental_phase	status
Pce_061	Anomala	Porcellanidae	Porcellanidae	*Allopetrolisthes*	*punctatus*	zoea	extant
Pce_062	Anomala	Porcellanidae	Porcellanidae	*Allopetrolisthes*	*punctatus*	megalopa	extant
Pce_063	Anomala	Porcellanidae	Porcellanidae	*Petrolisthes*	*armatus*	adult	extant
Pce_064	Anomala	Porcellanidae	Porcellanidae	*Polyonyx*	*tulearis*	adult	extant
Pce_065	Anomala	Porcellanidae	Porcellanidae	*Polyonyx*	*thai*	adult	extant
Pce_066	Anomala	Porcellanidae	Porcellanidae	*Petrolisthes*	*lamarckii*	megalopa	extant
Pce_067	Anomala	Porcellanidae	Porcellanidae	*Allopetrolisthes*	*spinifrons*	adult	extant

no	general_group	major_out	major_group	species_group	species	developmental_phase	status
Pce_068	Anomala	Porcellanidae	Porcellanidae	*Petrolisthes*	*refescens*	megalopa	extant
Pce_069	Anomala	Porcellanidae	Porcellanidae	*Petrolisthes*	*rufescens*	megalopa	extant
Pce_070	Anomala	Porcellanidae	Porcellanidae	*Petrolisthes*	*rufescens*	megalopa	extant
Pce_071	Anomala	Porcellanidae	Porcellanidae	*Psidia*	*gordoni*	megalopa	extant
Pce_072	Anomala	Porcellanidae	Porcellanidae	*Psidia*	*gordoni*	megalopa	extant
Pce_073	Anomala	Porcellanidae	Porcellanidae	*Psidia*	*gordoni*	megalopa	extant

no	general_group	major_out	major_group	species_group	species	developmental_phase	status
Pce_074	Anomala	Porcellanidae	Porcellanidae	*Psidia*	*gordoni*	megalopa	extant
Pce_075	Anomala	Porcellanidae	Porcellanidae	*Petrolisthes*	*platymerus*	megalopa	extant
Pce_076	Anomala	Porcellanidae	Porcellanidae	*Raphidopus*	*persicus*	adult	extant
Pce_077	Anomala	Porcellanidae	Porcellanidae	*Raphidopus*	*persicus*	adult	extant
Pce_078	Anomala	Porcellanidae	Porcellanidae	*Raphidopus*	*persicus*	adult	extant
Pce_079	Anomala	Porcellanidae	Porcellanidae	*Raphidopus*	*persicus*	adult	extant
Pce_080	Anomala	Porcellanidae	Porcellanidae	*Pachycheles*	*meloi*	adult	extant
Pce_081	Anomala	Porcellanidae	Porcellanidae	*Pachycheles*	*meloi*	adult	extant
Pce_082	Anomala	Porcellanidae	Porcellanidae	*Pachycheles*	*riisei*	adult	extant
Pce_083	Anomala	Porcellanidae	Porcellanidae	*Pachycheles*	*meloi*	adult	extant

no	general_group	major_out	major_group	species_group	species	developmental_phase	status
Pce_084	Anomala	Porcellanidae	Porcellanidae	*Pachycheles*	*riisei*	adult	extant
Pce_085	Anomala	Porcellanidae	Porcellanidae	*Petrolisthes*	*amoenus*	adult	extant
Pce_086	Anomala	Porcellanidae	Porcellanidae	*Petrolisthes*	*amoenus*	adult	extant
Pce_087	Anomala	Porcellanidae	Porcellanidae	*Petrolisthes*	*amoenus*	adult	extant
Pce_088	Anomala	Porcellanidae	Porcellanidae	*Petrolisthes*	*marginatus*	adult	extant
Port_001	Brachyura	Portunidae	Portunidae	*Charybdis*	*feriata*	megalopa	extant
Port_002	Brachyura	Portunidae	Portunidae	*Achelous*	*floridanus*	megalopa	extant
Port_003	Brachyura	Portunidae	Portunidae	*Portunus*	*hastatus*	megalopa	extant
Port_004	Brachyura	Portunidae	Portunidae	*Charybdis*	*hellerii*	megalopa	extant
Port_005	Brachyura	Portunidae	Portunidae	*Thalamita*	*admete*	megalopa	extant
Port_006	Brachyura	Portunidae	Portunidae	*Callinectes*	*toxotes*	megalopa	extant
Port_007	Brachyura	Portunidae	Portunidae	*Callinectes*	*arcuatus*	megalopa	extant
Port_008	Brachyura	Portunidae	Portunidae	*Callinectes*	*ornatus*	megalopa	extant

no	general_group	major_out	major_group	species_group	species	developmental_phase	status
Port_009	Brachyura	Portunidae	Portunidae	*Callinectes*	*amnicola*	megalopa	extant
Port_010	Brachyura	Portunidae	Portunidae	*Thalamita*	*crenata*	megalopa	extant
Port_011	Brachyura	Portunidae	Portunidae	*Thalamita*	*crenata*	juvenile	extant
Port_012	Brachyura	Portunidae	Portunidae	*Charybdis*	*hellerii*	juvenile	extant
Port_013	Brachyura	Portunidae	Portunidae	*Charybdis*	*hellerii*	juvenile	extant
Port_014	Brachyura	Portunidae	Portunidae	*Charybdis*	*hellerii*	juvenile	extant
Port_015	Brachyura	Portunidae	Portunidae	*Charybdis*	*truncata*	megalopa	extant
Port_016	Brachyura	Portunidae	Portunidae	*Arenaeus*	*cribrarius*	megalopa	extant
Port_017	Brachyura	Portunidae	Portunidae	*Arenaeus*	*cribrarius*	juvenile	extant
Port_018	Brachyura	Portunidae	Portunidae	*Arenaeus*	*cribrarius*	juvenile	extant
Port_019	Brachyura	Portunidae	Portunidae	*Arenaeus*	*cribrarius*	juvenile	extant
Port_020	Brachyura	Portunidae	Portunidae	*Lissocarcinus*	*orbicularis*	megalopa	extant
Port_021	Brachyura	Portunidae	Portunidae	*Lissocarcinus*	*orbicularis*	juvenile	extant
Port_022	Brachyura	Portunidae	Portunidae	*Charybdis*	*natator*	megalopa	extant
Port_023	Brachyura	Portunidae	Portunidae	*Portunus*	*pelagicus*	megalopa	extant
Port_024	Brachyura	Portunidae	Portunidae	*Thalamita*	*pelsarti*	megalopa	extant
Port_025	Brachyura	Portunidae	Portunidae	*Achelous*	*spinimanus*	megalopa	extant
Port_026	Brachyura	Portunidae	Portunidae	*Achelous*	*gibbesii*	megalopa	extant
Port_027	Brachyura	Portunidae	Portunidae	*Callinectes*	*danae*	juvenile	extant
Port_028	Brachyura	Portunidae	Portunidae	*Portunus*	*segnis*	megalopa	extant
Port_029	Brachyura	Portunidae	Portunidae	*Portunus*	*trituberculatus*	juvenile	extant
Port_030	Brachyura	Portunidae	Portunidae	*Achelous*	*spinimanus*	juvenile	extant
Port_031	Brachyura	Portunidae	Portunidae	*Achelous*	*spinimanus*	juvenile	extant

no	general_group	major_out	major_group	species_group	species	developmental_phase	status
Port_032	Brachyura	Portunidae	Portunidae	*Achelous*	*spinimanus*	juvenile	extant
Port_033	Brachyura	Portunidae	Portunidae	*Callinectes*	*danae*	juvenile	extant
Port_034	Brachyura	Portunidae	Portunidae	*Callinectes*	*danae*	juvenile	extant
Port_035	Brachyura	Portunidae	Portunidae	*Monomia*	*rubromarginatus*	megalopa	extant
Port_036	Brachyura	Portunidae	Portunidae	*Callinectes*	*danae*	juvenile	extant
Port_037	Brachyura	Portunidae	Portunidae	*Callinectes*	*danae*	juvenile	extant
Port_038	Brachyura	Portunidae	Portunidae	*Callinectes*	*danae*	juvenile	extant
Port_039	Brachyura	Portunidae	Portunidae	*Achelous*	*spinimanus*	juvenile	extant
Port_040	Brachyura	Portunidae	Portunidae	*Achelous*	*spinimanus*	juvenile	extant

no	general_group	major_out	major_group	species_group	species	developmental_phase	status
Port_041	Brachyura	Portunidae	Portunidae	*Achelous*	*spinimanus*	juvenile	extant
Port_042	Brachyura	Portunidae	Portunidae	*Callinectes*	*ornatus*	juvenile	extant
Port_043	Brachyura	Portunidae	Portunidae	*Portunus*	*pelagicus*	megalopa	extant
Port_044	Brachyura	Portunidae	Portunidae	*Achelous*	*spinicarpus*	megalopa	extant
Port_045	Brachyura	Portunidae	Portunidae	*Thalamita*	*chaptalii*	juvenile	extant
Port_046	Brachyura	Portunidae	Portunidae	*Portunus*	*sp.*	megalopa	extant
Port_047	Brachyura	Portunidae	Portunidae	*Charybdis*	*sp.*	megalopa	extant
Port_048	Brachyura	Portunidae	Portunidae	*Charybdis*	*sp.*	megalopa	extant
Port_049	Brachyura	Portunidae	Portunidae	*Thalamita*	*sp.*	megalopa	extant
Port_050	Brachyura	Portunidae	Portunidae	*Achelous*	*gibbesii*	megalopa	extant
Port_051	Brachyura	Portunidae	Portunidae	*Achelous*	*sp*	megalopa	extant
Port_052	Brachyura	Portunidae	Portunidae	*Achelous*	*sp*	megalopa	extant

no	general_group	major_out	major_group	species_group	species	developmental_phase	status
Port_053	Brachyura	Portunidae	Portunidae	*Achelous*	*sp*	megalopa	extant
Port_054	Brachyura	Portunidae	Portunidae	*Cronius*	*ruber*	megalopa	extant
Port_055	Brachyura	Portunidae	Portunidae	*Cronius*	*ruber*	megalopa	extant
Port_056	Brachyura	Portunidae	Portunidae	*Portunus*	*sp.*	megalopa	extant
Port_057	Brachyura	Portunidae	Portunidae	*Callinectes*	*sapidus*	megalopa	extant
Port_058	Brachyura	Portunidae	Portunidae	*Callinectes*	*similis*	megalopa	extant
Port_059	Brachyura	Portunidae	Portunidae	*Callinectes*	*sapidus*	megalopa	extant
Port_060	Brachyura	Portunidae	Portunidae	*Callinectes*	*similis*	megalopa	extant
Port_061	Brachyura	Portunidae	Portunidae	*Callinectes*	*sapidus*	juvenile	extant
Port_062	Brachyura	Portunidae	Portunidae	*Callinectes*	*similis*	juvenile	extant
Port_063	Brachyura	Portunidae	Portunidae	*Arenaeus*	*cribrarius*	juvenile	extant
Port_064	Brachyura	Portunidae	Portunidae	*Portunus*	*gibbesii*	juvenile	extant
Port_065	Brachyura	Portunidae	Portunidae	*Charybdis*	*feriata*	adult	extant
Port_066	Brachyura	Portunidae	Portunidae	*Portunus*	*hastatus*	adult	extant

no	general_group	major_out	major_group	species_group	species	developmental_phase	status
Port_067	Brachyura	Portunidae	Portunidae	*Charybdis*	*hellerii*	adult	extant
Port_068	Brachyura	Portunidae	Portunidae	*Thalamita*	*admete*	adult	extant
Port_069	Brachyura	Portunidae	Portunidae	*Callinectes*	*ornatus*	adult	extant
Port_070	Brachyura	Portunidae	Portunidae	*Thalamita*	*crenata*	adult	extant
Port_071	Brachyura	Portunidae	Portunidae	*Thalamita*	*crenata*	adult	extant
Port_072	Brachyura	Portunidae	Portunidae	*Charybdis*	*truncata*	adult	extant
Port_073	Brachyura	Portunidae	Portunidae	*Arenaeus*	*cribarius*	adult	extant
Port_074	Brachyura	Portunidae	Portunidae	*Lissocarcinus*	*orbicularis*	adult	extant

no	general_group	major_out	major_group	species_group	species	developmental_phase	status
Port_075	Brachyura	Portunidae	Portunidae	*Lissocarcinus*	*orbicularis*	adult	extant
Port_076	Brachyura	Portunidae	Portunidae	*Charybdis*	*natator*	adult	extant
Port_077	Brachyura	Portunidae	Portunidae	*Portunus*	*pelagicus*	adult	extant
Port_078	Brachyura	Portunidae	Portunidae	*Thalamita*	*pelsarti*	adult	extant
Port_079	Brachyura	Portunidae	Portunidae	*Achelous*	*spinimanus*	adult	extant
Port_080	Brachyura	Portunidae	Portunidae	*Achelous*	*spinimanus*	adult	extant
Port_081	Brachyura	Portunidae	Portunidae	*Achelous*	*gibbesii*	adult	extant

no	general_group	major_out	major_group	species_group	species	developmental_phase	status
Port_082	Brachyura	Portunidae	Portunidae	*Callinectes*	*danae*	adult	extant
Port_083	Brachyura	Portunidae	Portunidae	*Portunus*	*segnis*	adult	extant
Port_084	Brachyura	Portunidae	Portunidae	*Portunus*	*trituberculatus*	adult	extant
Port_085	Brachyura	Portunidae	Portunidae	*Monomia*	*rubromarginatus*	adult	extant
Port_086	Brachyura	Portunidae	Portunidae	*Achelous*	*spinicarpus*	adult	extant
Port_087	Brachyura	Portunidae	Portunidae	*Achelous*	*spinicarpus*	adult	extant
Port_088	Brachyura	Portunidae	Portunidae	*Thalamita*	*chaptalii*	adult	extant
Port_089	Brachyura	Portunidae	Portunidae	*Cronius*	*ruber*	adult	extant

no	general_group	major_out	major_group	species_group	species	developmental_phase	status
Port_090	Brachyura	Portunidae	Portunidae	*Cronius*	*ruber*	juvenile	extant
Port_091	Brachyura	Portunidae	Portunidae	*Callinectes*	*sapidus*	adult	extant
Port_092	Brachyura	Portunidae	Portunidae	*Callinectes*	*similis*	adult	extant
Port_093	Brachyura	Portunidae	Portunidae	*Charybdis*	*hellerii*	adult	extant
Port_094	Brachyura	Portunidae	Portunidae	*Portunus*	*paralatibrachium*	adult	extant
Port_095	Brachyura	Portunidae	Portunidae	*Portunus*	*lecromi*	adult	extant

no	general_group	major_out	major_group	species_group	species	developmental_phase	status
Port_096	Brachyura	Portunidae	Portunidae	*Portunus*	*iranjae*	adult	extant
Port_097	Brachyura	Portunidae	Portunidae	*Callinectes*	*sapidus*	adult	extant
Port_098	Brachyura	Portunidae	Portunidae	*Callinectes*	*cf. bocourti*	adult	extant
Port_099	Brachyura	Portunidae	Portunidae	*Callinectes*	*maginartus*	adult	extant
Port_100	Brachyura	Portunidae	Portunidae	*Thalamita*	*sima*	adult	extant
Port_101	Brachyura	Portunidae	Portunidae	*Thalamita*	*mitsiensis*	adult	extant
Port_102	Brachyura	Portunidae	Portunidae	*Thalamita*	*quadrilobata*	adult	extant
Port_103	Brachyura	Portunidae	Portunidae	*Thalamita*	*iranica*	adult	extant
Port_104	Brachyura	Portunidae	Portunidae	*Thalamita*	*bandusia*	adult	extant
Port_105	Brachyura	Portunidae	Portunidae	*Thalamita*	*bouvieri*	adult	extant

no	general_group	major_out	major_group	species_group	species	developmental_phase	status
Port_106	Brachyura	Portunidae	Portunidae	*Thalamita*	*gatavakensis*	adult	extant
Port_107	Brachyura	Portunidae	Portunidae	*Thalamita*	*savignyi*	adult	extant
Port_108	Brachyura	Portunidae	Portunidae	*Thalamita*	*picta*	adult	extant
Port_109	Brachyura	Portunidae	Portunidae	*Achelous*	*sebae*	adult	extant
Port_110	Brachyura	Portunidae	Portunidae	*Achelous*	*depressifrons*	adult	extant
Port_111	Brachyura	Portunidae	Portunidae	*Achelous*	*binoculus*	adult	extant
Port_112	Brachyura	Portunidae	Portunidae	*Achelous*	*ordwayi*	adult	extant
Port_113	Brachyura	Portunidae	Portunidae	*Charybdis*	*orientalis*	adult	extant
Port_114	Brachyura	Portunidae	Portunidae	*Charybdis*	*acuta*	adult	extant
Port_115	Brachyura	Portunidae	Portunidae	*Charybdis*	*miles*	adult	extant

no	general_group	major_out	major_group	species_group	species	developmental_phase	status
Port_116	Brachyura	Portunidae	Portunidae	*Charybdis*	*variegata*	adult	extant
Port_117	Brachyura	Portunidae	Portunidae	*Charybdis*	*longicollis*	adult	extant
Port_118	Brachyura	Portunidae	Portunidae	*Thranita*	*rubridens*	adult	extant
Port_119	Brachyura	Portunidae	Portunidae	*Thranita*	*tenuipes*	adult	extant
Port_120	Brachyura	Portunidae	Portunidae	*Lissocarcinus*	*laevis*	adult	extant
Port_121	Brachyura	Portunidae	Portunidae	*Lissocarcinus*	*polybiodes*	adult	extant
Port_122	Brachyura	Portunidae	Portunidae	*Lissocarcinus*	*orbicularis*	adult	extant
Port_125	Brachyura	Portunidae	Portunidae	*Portunus*	*spinicarpus*	zoea	extant
Port_126	Brachyura	Portunidae	Portunidae	*Portunus*	*spinicarpus*	zoea	extant
Port_127	Brachyura	Portunidae	Portunidae	*Portunus*	*spinicarpus*	zoea	extant
Port_128	Brachyura	Portunidae	Portunidae	*Portunus*	*spinicarpus*	zoea	extant
Port_129	Brachyura	Portunidae	Portunidae	*Portunus*	*spinicarpus*	zoea	extant
Port_130	Brachyura	Portunidae	Portunidae	*Portunus*	*spinicarpus*	zoea	extant

no	general_group	major_out	major_group	species_group	species	developmental_phase	status
Port_131	Brachyura	Portunidae	Portunidae	*Portunus*	*spinicarpus*	zoea	extant
Port_132	Brachyura	Portunidae	Portunidae	*Callinectes*	*similis*	zoea	extant
Port_133	Brachyura	Portunidae	Portunidae	*Callinectes*	*similis*	zoea	extant
Port_134	Brachyura	Portunidae	Portunidae	*Callinectes*	*similis*	zoea	extant
Port_135	Brachyura	Portunidae	Portunidae	*Callinectes*	*similis*	zoea	extant
Port_136	Brachyura	Portunidae	Portunidae	*Callinectes*	*similis*	zoea	extant
Port_137	Brachyura	Portunidae	Portunidae	*Callinectes*	*similis*	zoea	extant
Port_138	Brachyura	Portunidae	Portunidae	*Callinectes*	*similis*	zoea	extant
Port_139	Brachyura	Portunidae	Portunidae	*Callinectes*	*similis*	zoea	extant
Port_140	Brachyura	Portunidae	Portunidae	*Callinectes*	*sapidus*	zoea	extant
Port_141	Brachyura	Portunidae	Portunidae	*Callinectes*	*sapidus*	zoea	extant
Port_142	Brachyura	Portunidae	Portunidae	*Callinectes*	*sapidus*	zoea	extant
Port_143	Brachyura	Portunidae	Portunidae	*Callinectes*	*sapidus*	zoea	extant
Port_144	Brachyura	Portunidae	Portunidae	*Callinectes*	*sapidus*	zoea	extant
Port_145	Brachyura	Portunidae	Portunidae	*Callinectes*	*sapidus*	zoea	extant
Port_146	Brachyura	Portunidae	Portunidae	*Callinectes*	*sapidus*	zoea	extant
Port_147	Brachyura	Portunidae	Portunidae	*Callinectes*	*sapidus*	zoea	extant
Port_148	Brachyura	Portunidae	Portunidae	*Charybdis*	*hellerii*	zoea	extant

no	general_group	major_out	major_group	species_group	species	developmental_phase	status
Port_149	Brachyura	Portunidae	Portunidae	*Charybdis*	*hellerii*	zoea	extant
Port_150	Brachyura	Portunidae	Portunidae	*Thalamita*	*pelsarti*	zoea	extant
Port_151	Brachyura	Portunidae	Portunidae	*Thalamita*	*pelsarti*	zoea	extant
Port_152	Brachyura	Portunidae	Portunidae	*Thalamita*	*pelsarti*	zoea	extant
Port_153	Brachyura	Portunidae	Portunidae	*Thalamita*	*pelsarti*	zoea	extant
Port_154	Brachyura	Portunidae	Portunidae	*Thalamita*	*pelsarti*	zoea	extant
Ran_001	Brachyura	Raninidae	Raninidae	*Raninia*	*ranina*	zoea	extant
Ran_002	Brachyura	Raninidae	Raninidae	*Raninia*	*ranina*	megalopa	extant
Ran_003	Brachyura	Raninidae	Raninidae	Raninoides	benedicti	zoea	extant
Ran_004	Brachyura	Raninidae	Raninidae	Raninoides	benedicti	zoea	extant
Ran_005	Brachyura	Raninidae	Raninidae	Raninoides	benedicti	zoea	extant
Ran_006	Brachyura	Raninidae	Raninidae	Raninoides	benedicti	zoea	extant
Ran_007	Brachyura	Raninidae	Raninidae	Raninoides	benedicti	megalopa	extant
Ran_008	Brachyura	Raninidae	Raninidae	Raninoides	benedicti	juvenile	extant
Ran_009	Brachyura	Raninidae	Raninidae	Raninia	ranina	megalopa	extant
Ran_010	Brachyura	Raninidae	Raninidae	Raninia	ranina	juvenile	extant
Ran_011	Brachyura	Raninidae	Raninidae	Notopoides	latus	adult	extant
Ran_012	Brachyura	Raninidae	Raninidae	Notopoides	latus	adult	extant
Ran_013	Brachyura	Raninidae	Raninidae	Ranilia	triruformaculata	adult	extant
Ran_014	Brachyura	Raninidae	Raninidae	Ranilia	tenuiocellus	adult	extant
Ran_015	Brachyura	Raninidae	Raninidae	Ranina	ranina	adult	extant
Ran_016	Brachyura	Raninidae	Raninidae	Ranina	ranina	adult	extant

no	general_group	major_out	major_group	species_group	species	developmental_phase	status
Ran_017	Brachyura	Raninidae	Raninidae	Lyreidus	tridentatus	adult	extant
Ran_018	Brachyura	Raninidae	Raninidae	Lyreidus	tridentatus	adult	extant
Ran_019	Brachyura	Raninidae	Raninidae	Ranilia	muricata	adult	extant
Ran_020	Brachyura	Raninidae	Raninidae	Cosmonotus	grayii	adult	extant
Ran_021	Brachyura	Raninidae	Raninidae	Raninoides	louisianensis	adult	extant
Ran_022	Brachyura	Raninidae	Raninidae	Raninoides	louisianensis	adult	extant
Ran_023	Brachyura	Raninidae	Raninidae	Notosceles	chimonis	adult	extant

no	general_group	major_out	major_group	species_group	species	developmental_phase	status
Ran_024	Brachyura	Raninidae	Raninidae	Symethis	variolosa	adult	extant
Ran_025	Brachyura	Raninidae	Raninidae	Symethis	variolosa	adult	extant
Ran_026	Brachyura	Raninidae	Raninidae	Ranina	ranina	adult	extant
Ran_027	Brachyura	Raninidae	Raninidae	Ranina	ranina	adult	extant
Ran_028	Brachyura	Raninidae	Raninidae	Ranina	ranina	adult	extant
Ran_029	Brachyura	Raninidae	Raninidae	Ranina	ranina	adult	extant
Ran_030	Brachyura	Raninidae	Raninidae	Ranina	ranina	adult	extant
Xant_001	Brachyura	Xanthoidea	Panopeidae	*Eurytium*	*limosum*	adult	extant
Xant_002	Brachyura	Xanthoidea	Panopeidae	*Neopanope (Dyspanopeus)*	*texanus*	adult	extant

no	general_group	major_out	major_group	species_group	species	developmental_phase	status
Xant_003	Brachyura	Xanthoidea	Panopeidae	*Panopeus*	*herbstii*	adult	extant
Xant_004	Brachyura	Xanthoidea	Panopeidae	*Hexapanopeus*	*angustifrons*	adult	extant
Xant_006	Brachyura	Xanthoidea	Panopeidae	*Rhithropanopeus*	*harrisii*	adult	extant
Xant_007	Brachyura	Xanthoidea	Panopeidae	*Panopeus*	*austrobesus*	megalopa	extant
Xant_008	Brachyura	Xanthoidea	Xanthidae	*Banareia*	*fatuhiva*	adult	extant
Xant_009	Brachyura	Xanthoidea	Xanthidae	*Demania*	*garthi*	adult	extant
Xant_010	Brachyura	Xanthoidea	Xanthidae	*Demania*	*mortenseni*	adult	extant

no	general_group	major_out	major_group	species_group	species	developmental_phase	status
Xant_011	Brachyura	Xanthoidea	Xanthidae	*Paraxanthodes*	*polynesiensis*	adult	extant
Xant_012	Brachyura	Xanthoidea	Xanthidae	*Alainodaeus*	*akiaki*	adult	extant
Xant_013	Brachyura	Xanthoidea	Xanthidae	*Alainodaeus*	*rimatara*	adult	extant
Xant_014	Brachyura	Xanthoidea	Xanthidae	*Epistocavea*	*mururoa*	adult	extant
Xant_015	Brachyura	Xanthoidea	Xanthidae	*Medaeus*	*grandis*	adult	extant
Xant_016	Brachyura	Xanthoidea	Xanthidae	*Meractaea*	*tafai*	adult	extant
Xant_017	Brachyura	Xanthoidea	Xanthidae	*Rata*	*tuamotense*	adult	extant
Xant_018	Brachyura	Xanthoidea	Xanthidae	*Lophozozymus*	*bertonciniae*	adult	extant
Xant_019	Brachyura	Xanthoidea	Xanthidae	*Meriola (Lipkemera)*	*rufomaculata*	adult	extant
Xant_020	Brachyura	Xanthoidea	Panopeidae	*Eurytium*	*limosum*	adult	extant

no	general_group	major_out	major_group	species_group	species	developmental_phase	status
Xant_021	Brachyura	Xanthoidea	Panopeidae	*Panopeus*	*lacustris*	adult	extant
Xant_022	Brachyura	Xanthoidea	Panopeidae	*Rhithropanopeus*	*harrisii*	megalopa	extant
Xant_023	Brachyura	Xanthoidea	Panopeidae	*Hexapanopeus*	*carribaeus*	megalopa	extant
Xant_024	Brachyura	Xanthoidea	Panopeidae	*Dyspanopeus*	*sayi*	adult	extant

no	general_group	major_out	major_group	species_group	species	developmental_phase	status
Xant_025	Brachyura	Xanthoidea	Xanthidae	*Zozymodes (Zosimus)*	*aeneus*	adult	extant
Xant_026	Brachyura	Xanthoidea	Panopeidae	*Dyspanopeus*	*sayi*	adult	extant
Xant_027	Brachyura	Xanthoidea	Pseudorhombilidae	*Scopolius*	*nuttingi*	megalopa	extant
Xant_028	Brachyura	Xanthoidea	Chlorodiellinae	*Pilodius (Vellodius)*	*etisoides*	adult	extant
Xant_029	Brachyura	Xanthoidea	Panopeidae	*Lophopanopeus*	*bellus*	megalopa	extant

no	general_group	major_out	major_group	species_group	species	developmental_phase	status
Xant_030	Brachyura	Xanthoidea	Panopeidae	*Rhithropanopeus*	*harrisii*	adult	extant
Xant_031	Brachyura	Xanthoidea	Panopeidae	*Acantholobulus*	*mirafloresensis*	megalopa	extant
Xant_032	Brachyura	Xanthoidea	Panopeidae	*Hexapanopeus*	*angustifrons*	megalopa	extant
Xant_033	Brachyura	Xanthoidea	Panopeidae	*Panopeus*	*occidentalis*	megalopa	extant
Xant_034	Brachyura	Xanthoidea	Panopeidae	*Hexapanopeus*	*paulensis*	megalopa	extant
Xant_035	Brachyura	Xanthoidea	Xanthidae	*Xantho*	*poressa*	megalopa	extant
Xant_036	Brachyura	Xanthoidea	Xanthidae	*Xantho*	*poressa*	adult	extant
Xant_037	Brachyura	Xanthoidea	Xanthidae	*Neoliomera*	*cerasinus*	megalopa	extant

no	general_group	major_out	major_group	species_group	species	developmental_phase	status
Xant_038	Brachyura	Xanthoidea	Xanthidae	*Liomera*	*cinctimanu*	megalopa	extant
Xant_039	Brachyura	Xanthoidea	Xanthidae	*Pseudoliomera*	*variolosa*	megalopa	extant
Xant_040	Brachyura	Xanthoidea	Xanthidae	*Williamstimpsonia*	*stimpsoni*	megalopa	extant
Xant_041	Brachyura	Xanthoidea	Pseudorhombilidae	*Scopolius*	*nuttingi*	megalopa	extant
Xant_042	Brachyura	Xanthoidea	Xanthidae	*Etisus*	*odhneri*	megalopa	extant
Xant_043	Brachyura	Xanthoidea	Panopeidae	*Micropanope*	*sculptipes*	megalopa	extant

no	general_group	major_out	major_group	species_group	species	developmental_phase	status
Xant_044	Brachyura	Xanthoidea	Panopeidae	*Eurypanopeus*	*depressus*	megalopa	extant
Xant_045	Brachyura	Xanthoidea	Panopeidae	*Panopeus*	*africanus*	megalopa	extant
Xant_047	Brachyura	Xanthoidea	Panopeidae	*Dyspanopeus*	*sayi*	megalopa	extant
Xant_048	Brachyura	Xanthoidea	Panopeidae	*Rhithropanopeus*	*harrisii*	megalopa	extant
Xant_050	Brachyura	Xanthoidea	Panopeidae	*Dyspanopeus*	*texanus*	megalopa	extant

no	general_group	major_out	major_group	species_group	species	developmental_phase	status
Xant_051	Brachyura	Xanthoidea	Panopeidae	*Hexapaopeus*	*angustifrons*	megalopa	extant
Xant_052	Brachyura	Xanthoidea	Panopeidae	*Neopanope*	*packardii*	megalopa	extant
Xant_053	Brachyura	Xanthoidea	Panopeidae	*Panopeus*	*lacustris*	megalopa	extant

no	general_group	major_out	major_group	species_group	species	developmental_phase	status
Xant_054	Brachyura	Xanthoidea	Panopeidae	*Panopeus*	*herbstii*	megalopa	extant
Xant_055	Brachyura	Xanthoidea	Panopeidae	*Eurypanopeus*	*canalensis*	megalopa	extant
Xant_056	Brachyura	Xanthoidea	Xanthidae	*Atergatis*	*floridus*	megalopa	extant
Xant_057	Brachyura	Xanthoidea	Xanthidae	*Atergatis*	*subdentatus*	megalopa	extant
Xant_058	Brachyura	Xanthoidea	Xanthidae	*Atergatis*	*reticulatus*	megalopa	extant

no	general_group	major_out	major_group	species_group	species	developmental_phase	status
Xant_059	Brachyura	Xanthoidea	Pseudorhombilidae	*Bathyrhombila*	*sp.*	juvenile	extant
Xant_060	Brachyura	Xanthoidea	Pseudorhombilidae	*Trapezioplax*	*tridentata*	adult	extant
Xant_061	Brachyura	Xanthoidea	Xanthidae	*Xanthias (or Xanthodes)*	*elegans*	megalopa	extant
Xant_062	Brachyura	Xanthoidea	Xanthidae	*Xanthias (or Xanthodes)*	*elegans*	juvenile	extant
Xant_063	Brachyura	Xanthoidea	Panopeidae	*Micropanope*	*sculptipes*	adult	extant

no	general_group	major_out	major_group	species_group	species	developmental_phase	status
Xant_064	Brachyura	Xanthoidea	Xanthidae	*Demania*	*splendida*	adult	extant
Xant_065	Brachyura	Xanthoidea	Xanthidae	*Demania*	*toxica*	adult	extant
Xant_066	Brachyura	Xanthoidea	Xanthidae	*Novactaea*	*pulchella*	megalopa	extant
Xant_067	Brachyura	Xanthoidea	Xanthidae	*Cymo*	*cerasma*	adult	extant
Xant_068	Brachyura	Xanthoidea	Xanthidae	*Cymo*	*deplanatus*	adult	extant

no	general_group	major_out	major_group	species_group	species	developmental_phase	status
Xant_069	Brachyura	Xanthoidea	Xanthidae	*Leptodius*	*exaratus*	megalopa	extant
Xant_070	Brachyura	Xanthoidea	Xanthidae	*Cymo*	*quadrilobatus*	adult	extant
Xant_071	Brachyura	Pilumnidae	Pilumninae	*Pilumnus*	*spinifer*	megalopa	extant
Xant_072	Brachyura	Xanthoidea	Xanthidae	*Danielea*	*noelensis*	adult	extant
Xant_073	Brachyura	Xanthoidea	Xanthidae	*Demania*	*rotundata*	adult	extant

no	general_group	major_out	major_group	species_group	species	developmental_phase	status
Xant_074	Brachyura	Xanthoidea	Xanthidae	*Demania*	*scaberrima*	adult	extant
Xant_075	Brachyura	Xanthoidea	Xanthidae	*Demania*	*scaberrima*	adult	extant

no	general_group	major_out	major_group	species_group	species	developmental_phase	status
Xant_076	Brachyura	Xanthoidea	Xanthidae	*Actaea*	*jacquelinae*	megalopa	extant
Xant_077	Brachyura	Xanthoidea	Xanthidae	*Demania*	*reynaudii*	adult	extant

no	general_group	major_out	major_group	species_group	species	developmental_phase	status
Xant_078	Brachyura	Xanthoidea	Xanthidae	*Epiactaea*	*nodulosa*	adult	extant
Xant_079	Brachyura	Xanthoidea	Xanthidae	*Etisus*	*anaglyptus*	adult	extant
Xant_080	Brachyura	Xanthoidea	Panopeidae	*Panopeus*	*margentus*	megalopa	extant
Xant_081	Brachyura	Xanthoidea	Xanthidae	*Demania*	*cultripes*	adult	extant

**no**	**general_group**	**major_out**	**major_group**	**species_group**	**species**	**developmental_phase**	**status**
Xant_082	Brachyura	Pilumnidae	Pilumninae	*Pilumnus*	*dasypodus*	megalopa	extant
Xant_083	Brachyura	Xanthoidea	Xanthidae	*Demania*	*intermedia*	adult	extant
Xant_084	Brachyura	Xanthoidea	Xanthidae	*Palapedia*	*integra*	zoea	extant
Xant_085	Brachyura	Xanthoidea	Xanthidae	*Palapedia*	*integra*	zoea	extant

no	general_group	major_out	major_group	species_group	species	developmental_phase	status
Xant_086	Brachyura	Xanthoidea	Xanthidae	*Palapedia*	*integra*	zoea	extant
Xant_087	Brachyura	Xanthoidea	Xanthidae	*Palapedia*	*integra*	zoea	extant
Xant_088	Brachyura	Xanthoidea	Xanthidae	*Actaea*	*semblatae*	zoea	extant
Xant_089	Brachyura	Xanthoidea	Xanthidae	*Actaea*	*jacquelinae*	zoea	extant

no	general_group	major_out	major_group	species_group	species	developmental_phase	status
Xant_090	Brachyura	Xanthoidea	Xanthidae	*Xantho*	*hydrophilus*	zoea	extant
Xant_091	Brachyura	Xanthoidea	Xanthidae	*Pseudoliomera*	*speciosa*	zoea	extant
Xant_093	Brachyura	Xanthoidea	Xanthidae	*Actaea*	*jacquelinae*	zoea	extant

no	general_group	major_out	major_group	species_group	species	developmental_phase	status
Xant_094	Brachyura	Xanthoidea	Xanthidae	*Actaea*	*jacquelinae*	zoea	extant
Xant_096	Brachyura	Xanthoidea	Panopeidae	*Panopeus*	*Herbstii*	zoea	extant
Xant_097	Brachyura	Eriphioidea	Oziidae	*Eriphia*	*scabricula*	zoea	extant

no	general_group	major_out	major_group	species_group	species	developmental_phase	status
Xant_098	Brachyura	Eriphioidea	Oziidae	*Epixanthus*	*frontalis*	zoea	extant
Xant_100	Brachyura	Eriphioidea	Oziidae	*Lydia*	*annulipes*	zoea	extant
Xant_101	Brachyura	Xanthoidea	Xanthidae	*Cyclodius*	*paumotensis*	zoea	extant

no	general_group	major_out	major_group	species_group	species	developmental_phase	status
Xant_102	Brachyura	Xanthoidea	Xanthidae	*Lybia*	*plumosa*	zoea	extant
Xant_103	Brachyura	Xanthoidea	Xanthidae	*Leptodius*	*exaratus*	zoea	extant
Xant_104	Brachyura	Xanthoidea	Xanthidae	*Zozymodes*	*xanthoides*	zoea	extant

no	general_group	major_out	major_group	species_group	species	developmental_phase	status
Xant_105	Brachyura	Pilumnidae	Pilumninae	*Eurycarcinus*	*natalensis*	zoea	extant
Xant_106	Brachyura	Pilumnidae	Pilumninae	*Pilumnus*	*longicornis*	zoea	extant
Xant_107	Brachyura	Pilumnidae	Pilumninae	*Pilumnus*	*vespertilio*	zoea	extant
Xant_108	Brachyura	Xanthoidea	Xanthidae	*Lophozozymus*	*pictor*	zoea	extant

no	general_group	major_out	major_group	species_group	species	developmental_phase	status
Xant_109	Brachyura	Eriphioidea	Oziidae	*Ozius*	*truncatus*	zoea	extant
Xant_111	Brachyura	Xanthoidea	Xanthidae	*Platypodiella*	*spectabilis*	zoea	extant
Xant_112	Brachyura	Xanthoidea	Xanthidae	*Gaillardiellus*	*orientalis*	zoea	extant
Xant_113	Brachyura	Xanthoidea	Xanthidae	*Lophozozymus*	*pictor*	zoea	extant
Xant_114	Brachyura	Xanthoidea	Xanthidae	*Epiactaea*	*nodulosa*	zoea	extant
Xant_115	Brachyura	Xanthoidea	Xanthidae	*Novactaea*	*bella*	zoea	extant
Xant_116	Brachyura	Xanthoidea	Xanthidae	*Actaeodes*	*hirutissimus*	zoea	extant

no	general_group	major_out	major_group	species_group	species	developmental_phase	status
Xant_117	Brachyura	Xanthoidea	Xanthidae	*Lophozozymus*	*pictor*	zoea	extant

Supplementary T

no	fossil age	author	Reference number	year	figure	page	link
CaDi_002		WORMS					http://www.marinespecies.org/aphia.php?p=image&tid=107380&pic=75462
CaDi_003				1985			https://science.mnhn.fr/institution/mnhn/collection/iu/item/2008-11871
CaDi_004				1990			https://science.mnhn.fr/institution/mnhn/collection/iu/item/2014-22918
CaDi_005				1982			https://science.mnhn.fr/institution/mnhn/collection/iu/item/2008-11875
CaDi_006							https://science.mnhn.fr/institution/mnhn/collection/iu/item/2008-11835
CaDi_007		WORMS					http://www.marinespecies.org/aphia.php?p=image&tid=107278&pic=1869
CaDi_008		WORMS					http://www.marinespecies.org/aphia.php?p=image&tid=107278&pic=1870

no	fossil age	author	Reference number	year	figure	page	link
CaDi_009		WORMS					http://www.marinespecies.org/aphia.php?p=image&tid=107278&pic=34528
CaDi_010		WORMS					http://www.marinespecies.org/aphia.php?p=image&tid=107400&pic=1491
CaDi_011		WORMS					http://www.marinespecies.org/aphia.php?p=image&tid=107400&pic=3415
CaDi_012		WORMS					http://www.marinespecies.org/aphia.php?p=image&tid=107400&pic=3418
CaDi_013		WORMS					http://www.marinespecies.org/aphia.php?p=image&tid=107400&pic=34529
CaDi_014		WORMS					http://www.marinespecies.org/aphia.php?p=image&tid=107401&pic=138469
CaDi_015							https://science.mnhn.fr/institution/mnhn/collection/iu/item/2014-22996

no	fossil age	author	Reference number	year	figure	page	link
CaDi_016		Beschin and Checchi	[82]	2018	Tavola 1		
CaDi_017		GBIF					https://api.gbif.org/v1/image/unsafe/https%3A%2F%2Finaturalist-open-data.s3.amazonaws.com%2Fphotos%2F132170938%2Foriginal.jpeg%3F1622223794
CaDi_018		GBIF					https://api.gbif.org/v1/image/unsafe/https%3A%2F%2Finaturalist-open-data.s3.amazonaws.com%2Fphotos%2F131783330%2Foriginal.jpg%3F1622062050
CaDi_019		GBIF					https://api.gbif.org/v1/image/unsafe/http%3A%2F%2Fwww.boldsystems.org%2Fpics%2FCBCC%2FIMG_3660%2B1300448797.JPG

no	fossil age	author	Reference number	year	figure	page	link
CaDi_020		GBIF					https://api.gbif.org/v1/image/unsafe/http%3A%2F%2Fwww.boldsystems.org%2Fpics%2FCBCC%2FIMG_3663%2B1300448929.JPG
CaDi_021		GBIF					https://api.gbif.org/v1/image/unsafe/http%3A%2F%2Fwww.boldsystems.org%2Fpics%2FCBCC%2FIMG_3661%2B1300448881.JPG
CaDi_022		GBIF					https://science.mnhn.fr/institution/mnhn/collection/iu/item/2013-2326
CaDi_023		GBIF					https://science.mnhn.fr/institution/mnhn/collection/iu/item/2008-10171
CaDi_024		GBIF					https://science.mnhn.fr/institution/mnhn/collection/iu/item/2008-10173
CaDi_025		GBIF					https://science.mnhn.fr/institution/mnhn/collection/iu/item/2008-10172

no	fossil age	author	Reference number	year	figure	page	link
CaDi_026		GBIF					https://api.gbif.org/v1/image/unsafe/https%3A%2F%2Fkustod.io%2Fs%2F1EttLe9PYwnL0K8AO1mBBM.jpeg
CaDi_027		GBIF					https://api.gbif.org/v1/image/unsafe/https%3A%2F%2Finaturalist-open-data.s3.amazonaws.com%2Fphotos%2F77792796%2Foriginal.jpg%3F1591705959
CaDi_028		GBIF					https://api.gbif.org/v1/image/unsafe/http%3A%2F%2Fwww.observadoresdelmar.es%2Ffotos_observacions%2F4-662--20120812-pirimella1.jpg
CaDi_029		GBIF					https://science.mnhn.fr/institution/mnhn/collection/iu/item/2019-600

no	fossil age	author	Reference number	year	figure	page	link
CaDi_030		GBIF					https://api.gbif.org/v1/image/unsafe/https%3A%2F%2Finaturalist-open-data.s3.amazonaws.com%2Fphotos%2F120014016%2Foriginal.jpeg%3F1618073183
CaDi_031		GBIF					https://api.gbif.org/v1/image/unsafe/https%3A%2F%2Finaturalist-open-data.s3.amazonaws.com%2Fphotos%2F122740153%2Foriginal.jpeg%3F1619281714
CaDi_032		GBIF					https://api.gbif.org/v1/image/unsafe/https%3A%2F%2Finaturalist-open-data.s3.amazonaws.com%2Fphotos%2F146898012%2Foriginal.jpg%3F1627507173
CaDi_033		GBIF					https://api.gbif.org/v1/image/unsafe/https%3A%2F%2Fobservation.org%2Fphotos%2F22410121.jpg

no	fossil age	author	Reference number	year	figure	page	link
CaDi_034		GBIF					https://api.gbif.org/v1/image/unsafe/https%3A%2F%2Finaturalist-open-data.s3.amazonaws.com%2Fphotos%2F130798185%2Foriginal.jpeg%3F1621715453
CaDi_035		GBIF					https://api.gbif.org/v1/image/unsafe/https%3A%2F%2Finaturalist-open-data.s3.amazonaws.com%2Fphotos%2F45866871%2Foriginal.jpeg%3F1563902685
CaDi_036		GBIF					https://www.gbif.org/occurrence/1993787155
CaDi_037		Anosov	[67]	2000	fig. 1 I		
CaDi_038		Anosov	[67]	2000	fig. 1 II		
CaDi_039		Anosov	[67]	2000	fig. 1 XI		
CaDi_040		Paula	[360]	1988	fig. 7		
CaDi_041		Paula	[360]	1988	fig. 10		
CaDi_042		Crosnier	[128]	2002	fig. 1A		
CaDi_043		Crosnier	[128]	2002	fig. 1B		
CaDi_044		Crosnier	[128]	2002	fig. 3A		

no	fossil age	author	Reference number	year	figure	page	link
CaDi_045		Crosnier	[128]	2002	fig. 3B		
CaDi_046		Crosnier	[128]	2002	fig. 5A		
CaDi_047		Crosnier	[128]	2002	fig. 5B		
CaDi_048		Crosnier	[128]	2002	fig. 7A		
CaDi_049		Crosnier	[128]	2002	fig. 7B		
CaDi_050		Crosnier	[128]	2002	fig. 9A		
CaDi_051		Crosnier	[128]	2002	fig. 9B		
CaDi_052		Crosnier	[128]	2002	fig. 11A		
CaDi_053		Crosnier	[128]	2002	fig. 11B		
CaDi_054		Lebour	[276]	1944	fig. 3d		
CaDi_055		Lebour	[276]	1944	fig. 3e		
CaDi_056		Paula	[359]	1987	fig. 4a		
CaDi_057		Flores and Paula	[156]	2000	fig. 9a		
CaDi_058		Marco-Herrero et al.	[300]	2015	Plate 3 fig. 1a		
CaDi_059		Верещака	[81]	1989	fig. 1A		
CaDi_060		Верещака	[81]	1989	fig. 2A		
CaDi_061		Pancucci-Papadopoulou and Naletaki	[355]	2007	fig. 2		
CaDi_062		Pancucci-Papadopoulou and Naletaki	[355]	2007	fig. 3		
CaDi_063		Bedini	[80]	2006	fig. 6		
CaDi_064		Kocatas	[253]	1982	fig. 2		
CaDi_065		Gordon	[195]	1952	fig. 1		

no	fossil age	author	Reference number	year	figure	page	link
CaDi_066		Gordon	[195]	1952	fig. 2		
CaDi_067		Salas et al.	[393]	2001	fig. 1C		
CaDi_068		Reyes	[378]	2015	fig. 1.1		
CaDi_069		Kondylatos	[256]	2020	fig. 3A		
CaDi_070		Raso and Manjon-Cabeza	[374]	1996	fig. 1A		
CaDi_071		Macpherson	[294]	1989	fig. 1a		
CaDi_072		Macpherson	[294]	1989	fig. 1b		
CaDi_073		Pastore	[356]	1977	fig. 4a		
CaDi_074		Langeneck and Franco	[271]	2013	fig. 1.6		
CaDi_075		Langeneck and Franco	[271]	2013	fig. 1.7		
CaDi_076		Crosnier	[126]	1962	fig. 1		
CaDi_077		Crosnier	[126]	1962	fig. 2		
CaDi_078		Crosnier	[126]	1962	fig. 24		
CaDi_079		Fransen	[159]	2014	fig. 3, page 309		
CaDi_080		Fransen	[159]	2014	fig. 4, page 309		
CaDi_081		Fransen	[159]	2014	fig. 11, page 311		
CaDi_082		Rathbun	[375]	1926	Plate 16 fig.9 (page 166)		
CaDi_083		Karasawa and Fudouji	[236]	2000	Fig. 4 2b, page 245		
CaDi_084		Lörenthey & Breulen	[289]	1929	Tafel XV 3a		
CaDi_085		Lörenthey & Breulen	[289]	1929	Tafel XV 1b		
CaDi_086		Beschin et al.	[83]	2016	Tavola 11 5a, page 180		
CaDi_087							
CaDi_088		Evans	[147]	2018	fig. 1B		

no	fossil age	author	Reference number	year	figure	page	link
CaDi_089		Özcan and Ates	[353]	2018	fig. 2		
CaDi_090		Spiridonov	[457]	2020	fig. 4E		
CaDi_091							https://www.gbif.org/occurrence/2397665375
CaDi_092		Özcan et al.	[354]	2008	fig. 2		
CaDi_093		Nunes et al.	[348]	2017	fig. 3		
CaDi_094		Froglia and Manning	[164]	1982	fig. 2		
CaDi_095		Koch and Duris	[254]	2016	fig. 4A		
CaDi_096							https://www.gbif.org/occurrence/2826126812
CaDi_097							https://www.gbif.org/occurrence/3314928524
CaDi_098		Koch and Duris	[254]	2016	fig. 2A		
CaDi_099							https://www.ncbi.nlm.nih.gov/nuccore/KT209205
CaDi_100		Koch and Duris	[254]	2016	fig. 5A		
CaDi_101		Ng	[341]	2002	fig. 3A		
CaDi_102		Ng	[341]	2002	fig. 4A		
CaDi_103	middle Eocene	Schweitzer and Feldmann	[408]	2002	fig. 12B		
CaDi_104	middle Eocene	Schweitzer and Feldmann	[408]	2002	fig. 13C		
CaDi_105	lower Miocene	Feldmann et al.	[152]	2018	fig. 1A		
CaDi_106	middle Miocene (Badenian)	Ósso and Stalennuy	[352]	2011	fig. 7.1		
CaDi_107		Ósso and Stalennuy	[352]	2011	fig. 8.6		

no	fossil age	author	Reference number	year	figure	page	link
CaDi_108		Osso and Stalennuy	[352]	2011	fig. 8.7		
CaDi_109		Abbas et al.	[59]	2016	fig. 5		
CaDi_110		Kim and Hong	[242]	1999	fig. 7B		
CaDi_111	Oligocene	Schweitzer and Feldmann	[403]	2000	fig. 6		
CaDi_112	early Late Oligocene	Kato and Karasawa	[240]	1994	Plate 4		
CaDi_113	middle Eocene	Beschin and Checchi	[82]	2018	Tavola 1b		
CaDi_114	middle Pliocene	van Bakel et al.	[431]	2003	Plate 2.1		
CaDi_115		Guerao and Albello	[204]	2011	fig. 1A		
CaDi_116		Guerao and Albello	[204]	2011	fig. 1B		
CaDi_117		Guerao et al.	[203]	2006	fig.1B		
CaDi_118	Rupelian	Schweitzer and Feldmann	[409]	2010a	fig. 2D		
CaDi_119	Early Eocene	Beschin et al.	[83]	2016	fig. 46		
CaDi_120		Schweitzer et al.	[411]	2021	fig. 5.1		
CaDi_121	late Eocene						https://www.burkemuseum.org/collections-and-research/geology-and-paleontology/collections-database/images.php

no	fossil age	author	Reference number	year	figure	page	link
CaDi_122	late Eocene						https://www.burkemuseum.org/collections-and-research/geology-and-paleontology/collections-database/images.php
CaDi_123							http://bins.boldsystems.org/index.php/Public_RecordView?processid=DECNB303-17
CaDi_124		Ondřej Radosta					https://www.gbif.org/occurrence/1978472405
CaDi_125		Ondřej Radosta					https://www.gbif.org/occurrence/1978472404
CaDi_126							http://bins.boldsystems.org/index.php/Public_RecordView?processid=DECNB201-15
CaDi_127							http://bins.boldsystems.org/index.php/Public_RecordView?processid=SWEMA405-15
CaDi_128							http://bins.boldsystems.org/index.php/Public_RecordView?processid=DECNB009-13

no	fossil age	author	Reference number	year	figure	page	link
CaDi_129							https://science.mnhn.fr/institution/mnhn/collection/iu/item/2000-1063
CaDi_130							https://science.mnhn.fr/institution/mnhn/collection/iu/item/2019-612
CaDi_131							https://science.mnhn.fr/institution/mnhn/collection/iu/item/2014-22933
CaDi_132							https://www.gbif.org/occurrence/1993787146
CaDi_133							http://bins.boldsystems.org/index.php/Public_RecordView?processid=SWEMA549-15
CaDi_134							http://bins.boldsystems.org/index.php/Public_RecordView?processid=BNSDE127-12
CaDi_135		Guerao and Abelló	[450]	1999	fig. 1b		
CaDi_136		Guerao and Abelló	[450]	1999	fig. 5b		
CaDi_137		Guerao and Abelló	[450]	1999	fig. 9b		
CaDi_138		Rice and Ingle	[456]	1975	fig. 2b		
CaDi_139		Rice and Ingle	[456]	1975	fig. 2e		
CaDi_140		Rice and Ingle	[456]	1975	fig. 4b		

no	fossil age	author	Reference number	year	figure	page	link
CaDi_141		Rice and Ingle	[456]	1975	fig. 4e		
CaDi_142		Rice and Ingle	[456]	1975	fig. 5b		
CaDi_143		Rice and Ingle	[456]	1975	fig. 5e		
CaDi_144		Rice and Ingle	[456]	1975	fig. 6b		
CaDi_145		Rice and Ingle	[456]	1975	fig. 6e		
CaDi_146		Rice and Ingle	[456]	1975	fig. 7b		
CaDi_147		Rice and Ingle	[456]	1975	fig. 7e		
car_001		Gillespie et al.	[182]	2007	fig. 1		fig. 1
car_002		Hogarth	[220]	1978	fig. 1A		fig. 1A
car_003		Hogarth	[220]	1978	fig. 1B		fig. 1B
car_004		Hogarth	[220]	1978	fig. 1C		fig. 1C
car_005		Hogarth	[220]	1978	fig. 1D		fig. 1D
car_006		Hogarth	[220]	1978	fig. 1E		fig. 1E
car_007		Hogarth	[220]	1978	fig. 1F		fig. 1F
car_008		Kristensen et al.	[266]	2012	fig. 1a		fig. 1a
car_009		Spitzner et al.	[417]	2018	fig. 1a		fig. 1a
car_010		Todd et al.	[428]	2006	fig. 2a		fig. 2a
car_011		Todd et al.	[428]	2006	fig. 2b		fig. 2b
car_012		Todd et al.	[428]	2006	fig. 2c		fig. 2c
car_013		Todd et al.	[428]	2006	fig. 2d		fig. 2d
car_014		Todd et al.	[428]	2006	fig. 2e		fig. 2e
car_015		Todd et al.	[428]	2006	fig. 2f		fig. 2f
car_016					28D		
car_017					29G		
car_018					29H		
car_019					31A		
car_020					31B		
car_021					31C		
car_022					31D		
car_023					31E		
car_024					31F		

no	fossil age	author	Reference number	year	figure	page	link
car_025					31G		
car_026					31H		
car_027					31I		
car_028					31J		
car_029					2A		
car_030					2B		
car_031					2D		
car_032					31K		
car_033					31L		
car_034					31M		
car_035					31N		
car_036					31O		
car_037					32A		
car_038					33A		
car_039					33B		
car_040					W1		
car_041					W6		
car_042					W7		
car_043					W9		
car_044					W12		
car_045					W13		
car_046					W16		
car_047		Mengedoht			2009-C_maenas_DSC_4154-cu_e520x360		
car_048		Mengedoht			2009-C_maenas_DSC_4218-cu_e520x360		

no	fossil age	author	Reference number	year	figure	page	link
car_049		Mengedoht			2009-C_maenas_DSC_4454-cu_e520x360		
car_050		Mengedoht			2010-07-28_DSC_2532-cu_e520x360		
car_051		Mengedoht			2010-07-28_DSC_2546-cu_e520x360		
car_052		Mengedoht			2010-07-30_DSC_2808-cu_e520x360		
car_053		Mengedoht			2009-C_maenas_DSC_3678-cu_e520x360		
car_054		Mengedoht			2010-07-26_DSC_1707-cu_e520x360		
car_055		WORMS			662_carcinus-maenas-linnaeus-1758		
car_056		WORMS			29951_carcinus-maenas---carapace		
car_057		WORMS			39623_carcinus-maenas		
Dio_001		Bartilotti et al.	[79]	2007	Fig. 1a		
Dio_002		Bartilotti et al.	[79]	2007	Fig. 2g		
Dio_003		Bartilotti et al.	[79]	2007	Fig. 4a		
Dio_004		Nayak	[336]	1981	Fig. 1		
Dio_005		Nayak	[336]	1981	Fig. 12		
Dio_006		Nayak	[336]	1981	Fig. 24		
Dio_007		Nayak	[336]	1981	Fig. 37		

no	fossil age	author	Reference number	year	figure	page	link
Dio_008		Nayak	[336]	1981	Fig. 56		
Dio_009		Nayak and Kakati	[337]	1980	Fig. 1a1		
Dio_010		Nayak and Kakati	[337]	1980	Fig. 2a1		
Dio_011		Nayak and Kakati	[337]	1980	Fig. 4a		
Dio_012		Korn et al.	[261]	2008	Fig. 1a		
Dio_013		Korn et al.	[261]	2008	Fig. 4b		
Dio_014		Korn et al.	[261]	2008	Fig. 5b		
Dio_015		Korn et al.	[261]	2008	Fig. 6b		
Dio_016		Korn et al.	[261]	2008	Fig. 7b		
Dio_017		Korn et al.	[261]	2008	Fig. 8A		
Dio_018		Cházaro-Olvera et al.	[116]	2018	Fig. 3A		
Dio_019		Nishikawa et al.	[347]	2021	Fig. 1		
Dio_020		Nishikawa et al.	[347]	2021	Fig. 9a		
Dio_021		Cházaro-Olvera et al.	[115]	2013	Fig. 2a		
Dio_022		Cházaro-Olvera et al.	[115]	2013	Fig. 4a		
Dio_023		Lee and Ko	[277]	2012	Fig. 1B		
Dio_024		Lee and Ko	[277]	2012	Fig. 4A		
Dio_025		Hebling and Negreiros-Fransozo	[215]	1983	Fig. 1I		
Dio_026		Hebling and Negreiros-Fransozo	[215]	1983	Fig. 1II		
Dio_027		Hebling and Negreiros-Fransozo	[215]	1983	Fig. 1III		
Dio_028		Hebling and Negreiros-Fransozo	[215]	1983	Fig. 1IV		
Dio_029		Brossi-Gracia & Hebling	[101]	1983	Fig. 1I		

no	fossil age	author	Reference number	year	figure	page	link
Dio_030		Brossi-Gracia & Hebling	[101]	1983	Fig. 1II		
Dio_031		Brossi-Gracia & Hebling	[101]	1983	Fig. 1III		
Dio_032		Brossi-Gracia & Hebling	[101]	1983	Fig. 1IV		
Dio_033		Brossi-Gracia & Hebling	[101]	1983	Fig. 1V		
Dio_034		Brossi-Gracia & Hebling	[101]	1983	Fig. 1VI		
Dio_035		McLaughlin	[308]	2005	Fig. 1a		
Dio_036		McLaughlin	[308]	2005	Fig. 1d		
Dio_037		McLaughlin	[308]	2005	Fig. 3a		
Dio_038		McLaughlin	[308]	2005	Fig. 3d		
Dio_039		McLaughlin	[308]	2005	Fig. 4a		
Dio_040		McLaughlin	[308]	2005	Fig. 4d		
Dio_041		McLaughlin	[308]	2005	Fig. 5a		
Dio_042		McLaughlin	[308]	2005	Fig. 5d		
Dio_043		McLaughlin	[308]	2005	Fig. 6d		
Dio_044		Almón_et_al	[65]	2022	Fig. 1a		
Dio_045		Almón_et_al	[65]	2022	Fig. 5a		
Dio_046		Negri et al.	[338]	2012	Fig. 4B		
Dio_047		Negri et al.	[339]	2014	Fig. 3A		
Dio_048		Marin	[303]	2016	Fig. 1b		
Dio_049		Marin	[303]	2016	Fig. 3b		
Dio_050		Rahayu	[455]	2003	Fig. 1a		
Dio_051		Malay et al.	[297]	2004	Fig. 3C		
Dio_052		Malay et al.	[297]	2004	Fig. 3D		
Dio_053		Malay et al.	[297]	2004	Fig. 3E		
Dio_054		Malay et al.	[297]	2004	Fig. 3F		
Dio_055		Jung and Kim	[232]	2019	Fig. 1A		
Dio_056		Jung and Kim	[232]	2019	Fig. 1B		

no	fossil age	author	Reference number	year	figure	page	link
Dio_057		Jung and Kim	[232]	2019	Fig. 1C		
Dio_058		Jung and Kim	[232]	2019	Fig. 1D		
Dio_059		Barros-Alves_et_al	[78]	2015	Fig. 5A		
Dio_060		Lemaitre and Tavares	[286]	2015	Fig. 6A		
dro_001		McLay and Ng	[324]	2005	fig. 2		
dro_002		McLay and Ng	[324]	2005	fig. 3		
dro_003		McLay and Ng	[324]	2005	fig. 4		
dro_004		McLay et al.	[323]	2001	fig. 1A		
dro_005		McLay	[454]	2001	fig. 1A		
dro_006		McLay	[454]	2001	fig. 2A		
dro_007		McLay	[454]	2001	fig. 3A		
dro_008		Lewinsohn	[287]	1977	fig. 1A		
dro_009		Lewinsohn	[287]	1977	fig. 2A		
dro_010		Lewinsohn	[287]	1977	fig. 3A		
dro_011		Lewinsohn	[287]	1977	fig. 5A		
dro_012		Lewinsohn	[287]	1977	fig. 6A		
dro_013		Lewinsohn	[287]	1977	fig. 7A		
dro_014		Lewinsohn	[287]	1977	fig. 8A		
dro_015		Lewinsohn	[287]	1977	fig. 9A		
dro_016		McLay	[321]	1993	fig. 3		
dro_017		McLay	[321]	1993	fig. 6		
dro_018		McLay	[321]	1993	fig. 8		
dro_019		McLay	[321]	1993	fig. 9		
dro_020		McLay	[321]	1993	fig. 10		
dro_021		McLay	[321]	1993	fig. 11		
dro_022		McLay	[321]	1993	fig. 12		

no	fossil age	author	Reference number	year	figure	page	link
dro_023		McLay	[321]	1993	fig. 13		
dro_024		/		/	/		https://science.mnhn.fr/institution/mnhn/collection/iu/item/2014-8099
dro_025		/		/	/		https://science.mnhn.fr/institution/mnhn/collection/iu/item/2008-12865
dro_026		/		/	/		https://science.mnhn.fr/institution/mnhn/collection/iu/item/2014-8631
dro_027		/		/	/		https://science.mnhn.fr/institution/mnhn/collection/iu/item/2014-2197
dro_028		/		/	/		https://science.mnhn.fr/institution/mnhn/collection/iu/item/2013-405
dro_029		/		/	/		https://science.mnhn.fr/institution/mnhn/collection/iu/item/2013-523
dro_030		/		/	/		https://science.mnhn.fr/institution/mnhn/collection/iu/item/2013-948
dro_031		/		/	/		https://science.mnhn.fr/institution/mnhn/collection/iu/item/2013-1332

no	fossil age	author	Reference number	year	figure	page	link
dro_032		/		/	/		https://science.mnhn.fr/institution/mnhn/collection/iu/item/2008-11220
dro_033		/		/	/		https://science.mnhn.fr/institution/mnhn/collection/iu/item/2008-11817
dro_034		/		/	/		https://science.mnhn.fr/institution/mnhn/collection/iu/item/2013-389
dro_035		/		/	/		https://science.mnhn.fr/institution/mnhn/collection/f/item/b21561
dro_036		/		/	/		https://science.mnhn.fr/institution/mnhn/collection/iu/item/2008-11161
dro_037		/		/	/		https://science.mnhn.fr/institution/mnhn/collection/iu/item/2008-12775
dro_038		/		/	/		https://science.mnhn.fr/institution/mnhn/collection/iu/item/2008-11159

no	fossil age	author	Reference number	year	figure	page	link
dro_039		/		/	/		https://science.mnhn.fr/institution/mnhn/collection/iu/item/2008-11162
dro_040		/		/	/		https://science.mnhn.fr/institution/mnhn/collection/iu/item/2008-11166
dro_041		/		/	/		https://science.mnhn.fr/institution/mnhn/collection/iu/item/2008-11164
dro_042		/		/	/		https://science.mnhn.fr/institution/mnhn/collection/iu/item/2008-11158
dro_043		/		/	/		https://science.mnhn.fr/institution/mnhn/collection/iu/item/2008-11165
dro_044		/		/	/		https://science.mnhn.fr/institution/mnhn/collection/iu/item/2014-10059
dro_045		/		/	/		

no	fossil age	author	Reference number	year	figure	page	link
dro_046		/		/	/		https://science.mnhn.fr/institution/mnhn/collection/iu/item/2008-11170
dro_047		/		/	/		https://science.mnhn.fr/institution/mnhn/collection/iu/item/2013-260
dro_048		/		/	/		https://science.mnhn.fr/institution/mnhn/collection/iu/item/2013-531
dro_049		/		/	/		https://science.mnhn.fr/institution/mnhn/collection/iu/item/2013-527
dro_050		/		/	/		https://science.mnhn.fr/institution/mnhn/collection/iu/item/2013-1278
dro_051		/		/	/		https://science.mnhn.fr/institution/mnhn/collection/iu/item/2008-11059
dro_052		/		/	/		https://images.ala.org.au/image/9c00a7c3-a17a-417f-af5a-152b0d2538f9
dro_053		/		/	/		https://science.mnhn.fr/institution/mnhn/collection/iu/item/2008-12927

no	fossil age	author	Reference number	year	figure	page	link
dro_054		/		/	/		https://science.mnhn.fr/institution/mnhn/collection/iu/item/2008-11223
dro_055		/		/	/		https://science.mnhn.fr/institution/mnhn/collection/iu/item/2019-5357
dro_056		/		/	/		https://science.mnhn.fr/institution/mnhn/collection/iu/item/2008-11218
dro_057		/		/	/		https://science.mnhn.fr/institution/mnhn/collection/iu/item/2008-11482
dro_058		/		/	/		https://science.mnhn.fr/institution/mnhn/collection/iu/item/2008-11480
dro_059		/		/	/		https://science.mnhn.fr/institution/mnhn/collection/iu/item/2008-11481
dro_060		/		/	/		https://science.mnhn.fr/institution/mnhn/collection/iu/item/2008-11478

no	fossil age	author	Reference number	year	figure	page	link
dro_061		/		/	/		https://science.mnhn.fr/institution/mnhn/collection/iu/item/2013-2616
dro_062		/		/	/		https://cdn.floridamuseum.ufl.edu/IZ/7b109666-e01b-4a07-85be-f61acbfe4123/
dro_063		/		/	/		https://ala-images.s3.ap-southeast-2.amazonaws.com/store/2/e/d/1/0d2ea687-a8d2-4728-95ed-f07cc4571de2/thumbnail_large
dro_064		/		/	/		https://ala-images.s3.ap-southeast-2.amazonaws.com/store/1/1/8/d/16e5981a-fb48-4ae4-90d0-67ee0c9dd811/thumbnail_large
dro_065		/		/	/		https://science.mnhn.fr/institution/mnhn/collection/iu/item/2008-12942

no	fossil age	author	Reference number	year	figure	page	link
dro_066		/		/	/		https://science.mnhn.fr/institution/mnhn/collection/iu/item/2013-16661
dro_067		/		/	/		https://science.mnhn.fr/institution/mnhn/collection/iu/item/2008-12926
dro_068		/		/	/		https://science.mnhn.fr/institution/mnhn/collection/iu/item/2013-406
dro_069		/		/	/		https://science.mnhn.fr/institution/mnhn/collection/iu/item/2013-407
dro_070		/		/	/		https://science.mnhn.fr/institution/mnhn/collection/iu/item/2013-16662
dro_071		/		/	/		https://science.mnhn.fr/institution/mnhn/collection/iu/item/2013-16662
dro_072		/		/	/		https://science.mnhn.fr/institution/mnhn/collection/iu/item/2013-16662

no	fossil age	author	Reference number	year	figure	page	link
dro_073		/		/	/		https://science.mnhn.fr/institution/mnhn/collection/iu/item/2013-16662
dro_074		/		/	/		https://science.mnhn.fr/institution/mnhn/collection/iu/item/2013-16662
dro_075		/		/	/		https://science.mnhn.fr/institution/mnhn/collection/iu/item/2013-16662
dro_076		/		/	/		https://science.mnhn.fr/institution/mnhn/collection/iu/item/2013-16662
dro_079		Schweitzer and Feldmann	[410]	2010b	fig. 2A		
dro_080		Schweitzer and Feldmann	[410]	2010b	fig. 2B		
dro_081		Schweitzer and Feldmann	[410]	2010b	fig. 3E		
dro_082		Schweitzer and Feldmann	[410]	2010b	fig. 6A		
dro_083		Feldmann et al.	[151]	2010	fig. 3A		
dro_084		Feldmann et al.	[151]	2010	fig. 3E		
dro_085		Frantescu	[162]	2013	fig. 27 1		
dro_086		Artal et al.	[72]	2016	fig. 2		
dro_087		Davidson	[133]	1966	fig. 1		
dro_088		Blow and Manning	[85]	1996	fig. 1: 4		

no	fossil age	author	Reference number	year	figure	page	link
dro_089		Rice and Provenzano	[380]	1966	fig. 1: I		
dro_090		Rice and Provenzano	[380]	1966	fig. 1: II		
dro_091		Rice and Provenzano	[380]	1966	fig. 1: III		
dro_092		Rice and Provenzano	[380]	1966	fig. 1: IV		
dro_093		Rice and Provenzano	[380]	1966	fig. 1: V		
dro_094		Rice and Provenzano	[380]	1966	fig. 1: VI		
dro_095		Rice and Provenzano	[380]	1966	fig. 11		
dro_096		Hong and Williamson	[223]	1986	fig. 3b B		
dro_097		Hong and Williamson	[223]	1986	fig. 3b D		
dro_098		Hong and Williamson	[223]	1986	fig. 7A		
dro_099		Rice et al.	[381]	1970	fig. 1A		
dro_100		Rice et al.	[381]	1970	fig. 1C		
dro_101		Rice et al.	[381]	1970	fig. 2A		
dro_102		Rice et al.	[381]	1970	fig. 6A		
dro_103		Laughlin et al.	[272]	1982	fig. 1: I		
dro_104		Laughlin et al.	[272]	1982	fig. 1: II		
dro_105		Laughlin et al.	[272]	1982	fig. 1: III		
dro_106		Laughlin et al.	[272]	1982	fig. 1: VI		
dro_107		Laughlin et al.	[272]	1982	fig. 1: V		
dro_108		Laughlin et al.	[272]	1982	fig. 10		
dro_109		Tan et al.	[422]	1986	fig. 2A		
dro_110		Wear	[435]	1970	fig. 1		
dro_111		Wear	[435]	1970	fig. 4		

no	fossil age	author	Reference number	year	figure	page	link
dro_112		/		/	/		https://science.mnhn.fr/institution/mnhn/collection/iu/item/2008-11217
dro_113		/		/	/		https://science.mnhn.fr/institution/mnhn/collection/iu/item/2008-11216
dro_114		/		/	/		https://cdn.floridamuseum.ufl.edu/IZ/122ddf73-13c6-48a6-b45a-30ad259b92da/
dro_115		/		/	/		https://science.mnhn.fr/institution/mnhn/collection/iu/item/2008-11342
dro_116		/		/	/		https://science.mnhn.fr/institution/mnhn/collection/iu/item/2008-11180
dro_117		/		/	/		https://science.mnhn.fr/institution/mnhn/collection/iu/item/2008-12942
dro_118		/		/	/		https://science.mnhn.fr/institution/mnhn/collection/iu/item/2013-407

no	fossil age	author	Reference number	year	figure	page	link
dro_119		/		/	/		https://science.mnhn.fr/institution/mnhn/collection/iu/item/2013-406
dro_120		/		/	/		https://science.mnhn.fr/institution/mnhn/collection/iu/item/2008-12926
dro_121		/		/	/		https://science.mnhn.fr/institution/mnhn/collection/iu/item/2008-11222
dro_122		/		/	/		https://science.mnhn.fr/institution/mnhn/collection/iu/item/2008-11179
gal_001		/		/			
gal_002		/		/			
gal_003		/		/			
gal_004		/		/			
gal_005		/		/			
gal_006		/		/			

no	fossil age	author	Reference number	year	figure	page	link
gal_007		/		/			
gal_009		/		/			
gal_010		Christiansen and Anger	[117]	1990	fig. 1A		
gal_011		Christiansen and Anger	[117]	1990	fig. 1B		
gal_012		Christiansen and Anger	[117]	1990	fig. 1C		
gal_013		Christiansen and Anger	[117]	1990	fig. 1D		
gal_014		Fagetti and Campodonico	[148]	1971	fig. 1.1		
gal_015		Fagetti and Campodonico	[148]	1971	fig. 1.2		
gal_016		Fagetti and Campodonico	[148]	1971	fig.1.3		
gal_017		Fagetti and Campodonico	[148]	1971	fig. 1.4		
gal_018		Fagetti and Campodonico	[148]	1971	fig. 3.39		
gal_019		Fujita et al.	[171]	2001	fig. 1A		
gal_020		Fujita et al.	[171]	2001	fig. 3A		
gal_021		Fujita et al.	[171]	2001	fig. 5A		
gal_022		Fujita et al.	[171]	2001	fig. 7A		
gal_023		Fujita et al.	[171]	2001	fig. 9A		
gal_024		Fujita et al.	[173]	2003	fig. 3A		
gal_025		Fujita et al.	[173]	2003	fig. 3B		
gal_026		Fujita et al.	[173]	2003	fig. 3D		
gal_027		Fujita et al.	[173]	2003	fig. 3E		
gal_028		Lebour	[274]	1930	Plate I A, B		
gal_029		Lebour	[274]	1930	Plate I C		

no	fossil age	author	Reference number	year	figure	page	link
gal_030		Lebour	[274]	1930	Plate II A, B		
gal_031		Lebour	[274]	1930	Plate II C		
gal_032		Lebour	[274]	1930	Plate II D		
gal_033		Lebour	[274]	1930	Plate II E		
gal_034		Lebour	[274]	1930	Plate III A		
gal_035		Lebour	[274]	1930	Plate III B		
gal_036		Lebour	[274]	1930	Plate III C		
gal_037		Lebour	[274]	1930	Plate III D		
gal_038		Lebour	[274]	1930	Plate III E		
gal_039		Fujita and Shokita	[170]	2005	fig. 2A		
gal_040		Fujita and Shokita	[170]	2005	fig. 2C		
gal_041		Fujita and Shokita	[170]	2005	fig. 2E		
gal_042		Fujita and Shokita	[170]	2005	fig. 2F		
gal_043		Fujita	[166]	2007	fig. 1B		
gal_044		Fujita	[166]	2007	fig. 3B		
gal_045		Fujita	[167]	2010	fig. 2A		
gal_046		Fujita	[167]	2010	fig. 2B		
gal_048		Fujita	[167]	2010	fig. 2D		
gal_049		Konishi and Saito	[260]	2000	fig. 1A		
gal_050		Konishi and Saito	[260]	2000	fig. 3A		
gal_051		Mujica et al.	[333]	2019	fig. 1		
gal_052		Mujica et al.	[333]	2019	fig. 1		
gal_053		Mujica et al.	[333]	2019	fig. 1		
gal_054		Mujica et al.	[333]	2019	fig. 1		

no	fossil age	author	Reference number	year	figure	page	link
gal_055		Mujica et al.	[333]	2019	fig. 1		
gal_056		Lebour	[275]	1931	Plate 1A		
gal_057		Lebour	[275]	1931	Plate 1B		
gal_058		Lebour	[275]	1931	Plate 1C		
gal_059		Lebour	[275]	1931	Plate 1D		
gal_060		Lebour	[275]	1931	Plate 1F		
gal_061		Lebour	[275]	1931	Plate 1G		
gal_062		Lebour	[275]	1931	Plate 1H		
gal_063		Ahyong	[61]	2007	fig. 7A		
gal_064		Ahyong	[61]	2007	fig. 21A		
gal_065		Ahyong	[61]	2007	fig. 22A		
gal_066		Cabezas et al.	[103]	2010	fig. 1A		
gal_067		Cabezas et al.	[103]	2010	fig. 2A		
gal_068		Cabezas et al.	[103]	2010	fig. 3A		
gal_069		Cabezas et al.	[103]	2010	fig. 4A		
gal_070		Cabezas et al.	[103]	2010	fig. 5A		
gal_071		/		/	/		https://cdn.floridamuseum.ufl.edu/IZ/7053a234-1b9e-4f25-b942-223c9811e7de/
gal_072		/		/	/		https://cdn.floridamuseum.ufl.edu/IZ/2259469c-5e87-424f-ba10-a84cde323413/

no	fossil age	author	Reference number	year	figure	page	link
gal_073		/		/	/		https://cdn.floridamuseum.ufl.edu/IZ/9711f444-e4fb-4d67-bd93-09423af6bd41/
gal_074		/		/	/		https://cdn.floridamuseum.ufl.edu/IZ/ada4dd98-f2fe-4c3a-9597-52ead4b32119/
gal_075		/		/	/		https://cdn.floridamuseum.ufl.edu/IZ/4fcb8576-01ea-47ca-bb4c-aa80a463ce11/
gal_076		/		/	/		https://cdn.floridamuseum.ufl.edu/IZ/015c3bb6-4b3f-450a-ad9d-e38745ab164d/
gal_077		/		/	/		https://cdn.floridamuseum.ufl.edu/IZ/0a66e28b-5cbb-4f15-8764-329ff1b11ddd/
gal_078		/		/	/		https://cdn.floridamuseum.ufl.edu/IZ/066f5225-b18d-4486-94d9-1f7f89fc33c5/
gal_079		/		/	/		https://science.mnhn.fr/institution/mnhn/collection/iu/item/2019-719

no	fossil age	author	Reference number	year	figure	page	link
gal_080		/		/	/		https://science.mnhn.fr/institution/mnhn/collection/iu/item/2019-720
gal_081		/		/	/		https://science.mnhn.fr/institution/mnhn/collection/iu/item/2018-344
gal_082		/		/	/		https://cdn.floridamuseum.ufl.edu/IZ/0cb20512-6d2c-4035-82b1-896de8fbb19b/
gal_083		/		/	/	reddish specimen	https://cdn.floridamuseum.ufl.edu/IZ/dd967cf4-3916-47d3-8c22-63ecd034d93a/
gal_084		/		/	/	greenish specimen	https://cdn.floridamuseum.ufl.edu/IZ/e59019d6-cb4a-4fa9-b6d7-0faec4aa6d41/
gal_085		/		/	/		https://cdn.floridamuseum.ufl.edu/IZ/17e8d6c7-f37a-4c55-b4be-af10d9e88ab5/
gal_086		/		/	/		https://cdn.floridamuseum.ufl.edu/IZ/f71b498b-ff05-407f-ae7f-07d7a5c59dd4/

no	fossil age	author	Reference number	year	figure	page	link
gal_087		/		/	/		https://cdn.floridamuseum.ufl.edu/IZ/de922f98-2a33-4566-aa2e-fe6e60954f2f/
gal_088		/		/	/		https://science.mnhn.fr/institution/mnhn/collection/iu/item/2010-1651
gal_089		/		/	/		https://science.mnhn.fr/institution/mnhn/collection/iu/item/2010-1650
gal_090		/		/	/		https://science.mnhn.fr/institution/mnhn/collection/iu/item/2010-1652
gal_091		/		/	/		https://science.mnhn.fr/institution/mnhn/collection/iu/item/2014-5097
gal_092		/		/	/		https://cdn.floridamuseum.ufl.edu/IZ/cea1bd7a-0c62-4daf-859b-994152a4ce76/
gal_093		/		/	/		https://cdn.floridamuseum.ufl.edu/IZ/18e731b8-ed91-43ae-8f57-f9a0ade78c09
gal_094		/		/	/		https://cdn.floridamuseum.ufl.edu/IZ/ddf6d971-ce95-4071-a4fe-9df0895b339c/

no	fossil age	author	Reference number	year	figure	page	link
gal_095		/		/	/		https://science.mnhn.fr/institution/mnhn/collection/iu/item/2013-211
gal_096		/		/	/		https://science.mnhn.fr/institution/mnhn/collection/iu/item/2013-9974
gal_097		/		/	/		https://science.mnhn.fr/institution/mnhn/collection/iu/item/2013-16033
gal_098		/		/	/		https://science.mnhn.fr/institution/mnhn/collection/iu/item/2013-9968
gal_099		Poore et al.	[365]	2011	fig.2.2C, Seite 44		
gal_100		/		/	/		https://cdn.floridamuseum.ufl.edu/IZ/633eb58b-cf3f-4ca6-93f6-6695fb25679a/
gal_101		/		/	/		https://cdn.floridamuseum.ufl.edu/IZ/c7f7ce0b-8c60-491b-94b3-a20b40d92bd1/
gal_102		/		/	/		https://cdn.floridamuseum.ufl.edu/IZ/2725291d-7dc3-4bc7-b4f4-8b2b78b99b7c/

no	fossil age	author	Reference number	year	figure	page	link
gal_103		/		/	/		https://cdn.floridamuseum.ufl.edu/IZ/2f628641-806a-41d2-b2fa-448b9451174e/
gal_104		/		/	/		https://cdn.floridamuseum.ufl.edu/IZ/2556a56c-09b3-43c5-aaea-071344c705d7/
gal_105		/		/	/		https://cdn.floridamuseum.ufl.edu/IZ/d105d526-f89d-4141-bf93-df66bfabe6da/
gal_106		/		/	/		https://cdn.floridamuseum.ufl.edu/IZ/828bc984-69fb-43da-a405-769e9648e868/
gal_107		/		/	/		https://cdn.floridamuseum.ufl.edu/IZ/27197abd-4704-46be-a0d9-cb111da99c18/
gal_108		/		/	/		https://cdn.floridamuseum.ufl.edu/IZ/2bd9082f-74be-490b-a067-6a4c5c8cb29e/
gal_109		/		/	/		https://science.mnhn.fr/institution/mnhn/collection/iu/item/2013-17419

no	fossil age	author	Reference number	year	figure	page	link
gal_110		/		/	/		https://science.mnhn.fr/institution/mnhn/collection/iu/item/2013-17411
gal_111		/		/	/		https://science.mnhn.fr/institution/mnhn/collection/iu/item/2013-17396
gal_112		/		/	/		https://science.mnhn.fr/institution/mnhn/collection/iu/item/2013-17398
gal_113		/		/	/		https://science.mnhn.fr/institution/mnhn/collection/iu/item/2013-17412
gal_114		/		/	/		https://science.mnhn.fr/institution/mnhn/collection/iu/item/2013-503
gal_115		/		/	/		https://farm2.staticflickr.com/1917/45600635422_79c52754ac_o.png
gal_116		/		/	/		https://cdn.floridamuseum.ufl.edu/IZ/072c2db5-80f1-40b2-84ab-f14875acba39

no	fossil age	author	Reference number	year	figure	page	link
gal_117		/		/	/		https://cdn.floridamuseum.ufl.edu/IZ/8050a092-15ff-4363-893a-8e7d580c0c61/
gal_118		/		/	/		https://cdn.floridamuseum.ufl.edu/IZ/de922f98-2a33-4566-aa2e-fe6e60954f2f
gal_119		/		/	/		https://cdn.floridamuseum.ufl.edu/IZ/f7f8d89d-34e3-4524-8a25-c07b0ae456b2/
gal_120		/		/	/		https://cdn.floridamuseum.ufl.edu/IZ/b15ef6f3-c317-4118-9390-e25f9ad159e3/
gal_121		/		/	/		https://cdn.floridamuseum.ufl.edu/IZ/68cda77d-b713-46a1-96fa-05fe144389ad/
gal_122		/		/	/		https://cdn.floridamuseum.ufl.edu/IZ/3de68544-a2df-4fae-849e-b32e8f54c51b/
gal_123		/		/	/		https://cdn.floridamuseum.ufl.edu/IZ/1f3cc5a1-e4d7-4d1a-a22f-534a4a8aae46/

no	fossil age	author	Reference number	year	figure	page	link
gal_124		/		/	/		https://science.mnhn.fr/institution/mnhn/collection/iu/item/2013-258
gal_125		/		/	/		https://science.mnhn.fr/institution/mnhn/collection/iu/item/2010-5252
gal_126		/		/	/		https://science.mnhn.fr/institution/mnhn/collection/iu/item/2010-5256
gal_127		/		/	/		https://science.mnhn.fr/institution/mnhn/collection/iu/item/2010-5257
gal_128		/		/	/		https://science.mnhn.fr/institution/mnhn/collection/iu/item/2010-5256
gal_129		/		/	/		https://science.mnhn.fr/institution/mnhn/collection/iu/item/2010-5257
gal_130		/		/	/		https://science.mnhn.fr/institution/mnhn/collection/iu/item/2010-5254
gal_131		/		/	/		https://science.mnhn.fr/institution/mnhn/collection/iu/item/2010-5253
gal_132		/		/	/		https://science.mnhn.fr/institution/mnhn/collection/iu/item/2010-5255

no	fossil age	author	Reference number	year	figure	page	link
gal_133		/		/	/		https://science.mnhn.fr/institution/mnhn/collection/iu/item/2010-1116
gal_134		/		/	/		https://science.mnhn.fr/institution/mnhn/collection/iu/item/2010-1018
gal_135		/		/	/		https://science.mnhn.fr/institution/mnhn/collection/iu/item/2014-11139
gal_136		/		/	/		https://science.mnhn.fr/institution/mnhn/collection/iu/item/2009-1351
gal_137		/		/	/		https://cdn.floridamuseum.ufl.edu/IZ/281c60ba-ece0-4d30-95cf-4b0b3657ee3b
gal_138		/		/	/		https://cdn.floridamuseum.ufl.edu/IZ/96789e75-4df4-453b-b79d-fc81d5f253bb/
gal_139		/		/	/		https://cdn.floridamuseum.ufl.edu/IZ/b1d4952b-f8e3-4220-a9d2-dec8a93bd6eb
gal_140		/		/	/		https://science.mnhn.fr/institution/mnhn/collection/iu/item/2010-5264

no	fossil age	author	Reference number	year	figure	page	link
gal_141		/		/	/		https://science.mnhn.fr/institution/mnhn/collection/iu/item/2010-5264
gal_142		/		/	/		https://science.mnhn.fr/institution/mnhn/collection/iu/item/2010-5262
gal_143		/		/	/		https://science.mnhn.fr/institution/mnhn/collection/iu/item/2010-5263
gal_144		Fujita et al.	[173]	2003	fig. 3F		
gal_145		Fujita et al.	[171]	2001	fig. 11A		
gal_146		Christiansen and Anger	[117]	1990	fig. 12A		
gal_147		Gore	[192]	1978	fig. 8B		https://books.google.de/books?hl=en&lr=&id=JhRTP7FxehwC&oi=fnd&pg=PA781&dq=galatheidae%2Bdevelopment&ots=65zPHeS-8E&sig=Q5XQT-YP8ohXSU-NJKFxkMKyXuQ&redir_esc=y#v=onepage&q=galatheidae%2Bdevelopment&f=false
gal_148		Fujita	[167]	2010	fig. 2E		
gal_149		Mujica et al.	[333]	2019	fig. 5A		
gal_150		De Angeli and Garassino	[136]	2003	Fig. 1: 1		

no	fossil age	author	Reference number	year	figure	page	link
gal_151		De Angeli and Garassino	[136]	2003	Fig. 1: 4		
gal_152		De Angeli and Garassino	[136]	2003	Fig. 1: 7		
gal_153		Karasawa and Hayakawa	[237]	2000	Fig. 4: 1		
gal_154		Garassino et al.	[175]	2008	Fig. 11		
gal_155		Macpherson and Robainas-Barcia	[296]	2015	fig. 2A		
gal_156		Macpherson and Robainas-Barcia	[296]	2015	fig. 3A		
gal_157		Macpherson and Robainas-Barcia	[296]	2015	fig. 4A		
gal_158		Macpherson and Robainas-Barcia	[296]	2015	fig. 5A		
gal_159		Macpherson and Robainas-Barcia	[296]	2015	fig. 6A		
gal_160		Macpherson and Robainas-Barcia	[296]	2015	fig. 7A		
gal_161		Macpherson and Robainas-Barcia	[296]	2015	fig. 9A		
gal_162		Macpherson and Robainas-Barcia	[296]	2015	fig. 10A		
gal_163		Macpherson and Robainas-Barcia	[296]	2015	fig. 11A		
gal_164		Macpherson and Robainas-Barcia	[296]	2015	fig. 12A		
gal_165		Macpherson and Robainas-Barcia	[296]	2015	fig. 13A		
gal_166		Macpherson and Robainas-Barcia	[296]	2015	fig. 14A		
gal_167		Macpherson and Robainas-Barcia	[296]	2015	fig. 15A		
gal_168		Macpherson and Robainas-Barcia	[296]	2015	fig. 16A		

no	fossil age	author	Reference number	year	figure	page	link
gal_169		Macpherson and Robainas-Barcia	[296]	2015	fig. 17A		
gal_170		Macpherson and Robainas-Barcia	[296]	2015	fig. 18A		
gal_171		Macpherson and Robainas-Barcia	[296]	2015	fig. 19A		
gal_172		Macpherson and Robainas-Barcia	[296]	2015	fig. 20A		
gal_173		Macpherson and Robainas-Barcia	[296]	2015	fig. 21A		
gal_174		Macpherson and Robainas-Barcia	[296]	2015	fig. 22A		
gal_175		Macpherson and Robainas-Barcia	[296]	2015	fig. 23A		
gal_176		Macpherson and Robainas-Barcia	[296]	2015	fig. 24A		
gal_177		Macpherson and Robainas-Barcia	[296]	2015	fig. 25A		
gal_178		Macpherson and Robainas-Barcia	[296]	2015	fig. 26A		
gal_179		Macpherson and Robainas-Barcia	[296]	2015	fig. 27A		
gal_180		Macpherson and Robainas-Barcia	[296]	2015	fig. 28A		
gal_181		Macpherson and Robainas-Barcia	[296]	2015	fig, 29A		
gal_182		Macpherson and Robainas-Barcia	[296]	2015	fig. 30A		
gal_183		Macpherson and Robainas-Barcia	[296]	2015	fig. 31A		
gal_184		Macpherson and Robainas-Barcia	[296]	2015	fig. 32A		
gal_185		Macpherson and Robainas-Barcia	[296]	2015	fig. 33A		
gal_186		Macpherson and Robainas-Barcia	[296]	2015	fig. 34A		

no	fossil age	author	Reference number	year	figure	page	link
gal_187		Macpherson and Robainas-Barcia	[296]	2015	fig. 35A		
gal_188		Macpherson and Robainas-Barcia	[296]	2015	fig. 36A		
gal_189		Macpherson and Robainas-Barcia	[296]	2015	fig. 37A		
gal_190		Macpherson and Robainas-Barcia	[296]	2015	fig. 38A		
gal_191		Macpherson and Robainas-Barcia	[296]	2015	fig. 39A		
gal_192		Macpherson and Robainas-Barcia	[296]	2015	fig. 40A		
gal_193		Macpherson and Robainas-Barcia	[296]	2015	fig. 41A		
gal_194		Macpherson and Robainas-Barcia	[296]	2015	fig. 42A		
gal_195		Macpherson and Robainas-Barcia	[296]	2015	fig. 43A		
gal_196		Macpherson and Robainas-Barcia	[296]	2015	fig. 44A		
gal_197		Macpherson and Robainas-Barcia	[296]	2015	fig. 45A		
gal_198		Macpherson and Robainas-Barcia	[296]	2015	fig. 46A		
gal_199		Macpherson and Robainas-Barcia	[296]	2015	fig. 47A		
gal_200		Macpherson and Robainas-Barcia	[296]	2015	fig. 48A		
gal_201		Macpherson and Robainas-Barcia	[296]	2015	fig. 49A		
gal_202		Macpherson and Robainas-Barcia	[296]	2015	fig. 50A		
gal_203		Macpherson and Robainas-Barcia	[296]	2015	fig. 52A		
gal_204		Macpherson and Robainas-Barcia	[296]	2015	fig. 53A		

no	fossil age	author	Reference number	year	figure	page	link
gal_205		Macpherson and Robainas-Barcia	[296]	2015	fig. 54A		
gal_206		Macpherson and Robainas-Barcia	[296]	2015	fig. 55A		
gal_207		Macpherson and Robainas-Barcia	[296]	2015	fig. 56A		
gal_208		Macpherson and Robainas-Barcia	[296]	2015	fig. 57A		
gal_209		Macpherson and Robainas-Barcia	[296]	2015	fig, 58A		
gal_210		Macpherson and Robainas-Barcia	[296]	2015	fig. 59A		
gal_211		Macpherson and Robainas-Barcia	[296]	2015	fig. 60A		
gal_212		Macpherson and Robainas-Barcia	[296]	2015	fig. 61A		
gal_213		Macpherson and Robainas-Barcia	[296]	2015	fig. 62A		
gal_214		Macpherson and Robainas-Barcia	[296]	2015	fig. 63A		
gal_215		Macpherson and Robainas-Barcia	[296]	2015	fig. 64A		
gal_216		Macpherson and Robainas-Barcia	[296]	2015	fig. 65A		
Gra_001		Bacon	[73]	1971	Fig. 2		
Gra_002		Griffin	[199]	1973	Fig. 6A		
Gra_003		Maciel & Alves	[292]	2009	Fig. 4		
Gra_004		Souza	[416]	2008	Fig. 7		
Gra_005		Eteobong et al.	[146]	2016	Pl. 1		
Gra_006		Lawal-Are et al.	[273]	2019	Pl. 2		
Gra_007		Onadeko et al.	[350]	2015	Pl. 5		
Gra_008		Takeda & Sugiyama	[425]	2018	Fig. 3E		
Gra_009		Chace	[107]	1951	Fig. 1c		

no	fossil age	author	Reference number	year	figure	page	link
Gra_010		Chace	[107]	1951	Fig. 1a		
Gra_011		Chace	[107]	1951	Fig. 1b		
Gra_012		Arnaud et al.	[70]	1972	Pl. 2 Fig. 3		
Gra_016		Yaghmour & Al Naqbi	[444]	2020	Fig. 2C		
Gra_017		Dell	[137]	1963	Fig. 1		
Gra_018		Crosnier	[127]	1965	Fig. 13		
Gra_019		Crosnier	[127]	1965	Fig. 21		
Gra_020		Crosnier	[127]	1965	Fig. 23		
Gra_021		Crosnier	[127]	1965	Fig. 24		
Gra_022		Schubart et al.	[399]	2005	Fig. 5		
Gra_023		Crosnier	[127]	1965	Fig. 33		
Gra_024		Crosnier	[127]	1965	Fig. 35		
Gra_026		Takeda & Kurata	[426]	1977	Fig. 6A		
Gra_038		McLay and Ryan	[322]	2012	Fig. 2A		
Gra_039		Tweedie	[430]	1936	Pl. XIV Fig. 1		
Gra_040		Tweedie	[430]	1936	Pl. XIV Fig. 2		
Gra_041		Fujita	[168]	2016	Fig. 7B		
Gra_042		Fujita	[168]	2016	Fig. 7C		
Gra_043		Fujita	[168]	2016	Fig. 7D		
Gra_044		Fujita	[168]	2016	Fig. 7F		
Gra_045		Bouchard et al.	[96]	2013	Fig. 20A		
Gra_046		Bouchard et al.	[96]	2013	Fig. 20B		
Gra_047		Bouchard et al.	[96]	2013	Fig. 20C		
Gra_048		Bouchard et al.	[96]	2013	Fig. 20E		
Gra_049		Bouchard et al.	[96]	2013	Fig. 21A		
Gra_050		Bouchard et al.	[96]	2013	Fig. 21C		
Gra_051		Bouchard et al.	[96]	2013	Fig. 22A		
Gra_052		Bouchard et al.	[96]	2013	Fig. 22C		
Gra_053		Bouchard et al.	[96]	2013	Fig. 23A		
Gra_054		Nakano & Minato	[335]	2020	Fig. 3H		

no	fossil age	author	Reference number	year	figure	page	link
Gra_055		Fujita	[169]	2017	Fig. 5E		
Gra_056		Fujita	[169]	2017	Fig. 5F		
Gra_057		Fujita	[169]	2017	Fig. 5G		
Gra_058		Fujita	[169]	2017	Fig. 5H		
Gra_059		Fujita	[169]	2017	Fig. 6A		
Gra_060		Fujita	[169]	2017	Fig. 6B		
Gra_061		Fujita	[169]	2017	Fig. 6D		
Gra_062		Poupin et al.	[366]	2013	Fig. 9A		
Gra_063		Poupin et al.	[366]	2013	Fig. 9C		
Gra_064		Poupin et al.	[366]	2013	Fig. 9D		
Gra_065		Poupin et al.	[366]	2013	Fig. 9E		
Gra_066		Banerjee	[77]	1960	Fig. 1a		
Gra_067		Banerjee	[77]	1960	Fig. 1b		
Gra_068		Banerjee	[77]	1960	Fig. 1c		
Gra_069		Banerjee	[77]	1960	Fig. 1d		
Gra_070		Banerjee	[77]	1960	Fig. 1e		
Gra_072		Banerjee	[77]	1960	Fig. 5b		
Gra_073		Banerjee	[77]	1960	Fig. 5c		
Gra_074		Banerjee	[77]	1960	Fig. 5d		
Gra_075		Banerjee	[77]	1960	Fig. 5e		
Gra_076		Banerjee	[77]	1960	Fig. 5f		
Gra_077		Banerjee	[77]	1960	Fig. 5g		
Gra_078		Stimpson	[419]	1907	Pl. XVI Fig. 3		
Gra_079		Stimpson	[419]	1907	Pl. XVI Fig. 4		
Gra_080		Zaouali et al.	[448]	2008	Fig. 1		
Gra_081		Raghunathan	[371]	2015	Pl. 14(B)		
Gra_082		Raghunathan	[371]	2015	Pl. 14(C)		
Gra_083		Ng et al.	[343]	2001	Fig. 8e		
Gra_090		Kakati	[235]	1982	Fig. 46		
Gra_092		Paulay	[361]	2007	Fig. 1A		
Gra_093		Paulay	[361]	2007	Fig. 1C		

no	fossil age	author	Reference number	year	figure	page	link
Gra_094		Paulay	[361]	2007	Fig. 1D		
Gra_100		Pasupathi & Kannupandi	[357]	1986	Fig. 6a		
Gra_101		Innocenti et al.	[226]	2020	Fig. 1A		
Gra_102		Innocenti et al.	[226]	2020	Fig. 1B		
Gra_103		Innocenti et al.	[226]	2020	Fig. 6A		
Gra_104		Innocenti et al.	[226]	2020	Fig. 6B		
Gra_105		Innocenti et al.	[226]	2020	Fig. 6C		
Gra_106		Innocenti et al.	[226]	2020	Fig. 6D		
Gra_107		Innocenti et al.	[226]	2020	Fig. 6E		
Gra_108		Itoh	[229]	2020	Fig. 2A		
Gra_109		Itoh	[229]	2020	Fig. 2D		
Gra_110		Itoh	[229]	2020	Fig. 2F		
Gra_111		Priyadashani et al.	[367]	2008	Fig. 3a		
Gra_112		Harshith et al.	[208]	2016	Pl. 1		
Gra_113		Bandekar	[76]	2021	Pl. 1 1		
Gra_114		Bandekar	[76]	2021	Pl. 1 2		
Gra_115		Apreshgi & Kurian	[68]	2019	Fig. 2 5		
Gra_116		Apreshgi & Kurian	[68]	2019	Fig. 2 6		
Gra_117		Apreshgi & Kurian	[68]	2019	Fig. 2 7		
Gra_122		Pawar	[362]	2017	Fig. 2 (1)		
Gra_123		Pawar	[362]	2017	Fig. 2 (2)		
Gra_124		Pawar	[362]	2017	Fig. 2 (3)		
Gra_125		Pawar	[362]	2017	Fig. 2 (4)		
Gra_126		Pawar	[362]	2017	Fig. 2 (5)		
Gra_127		Guerao et al.	[200]	1997	Fig. 1A		
Gra_128		Guerao et al.	[200]	1997	Fig. 4A		
Gra_129		Guerao et al.	[200]	1997	Fig. 4F		

no	fossil age	author	Reference number	year	figure	page	link
Gra_130		Guerao et al.	[200]	1997	Fig. 4F		
Gra_131		Guerao et al.	[200]	1997	Fig. 4F		
Gra_132		Guerao et al.	[200]	1997	Fig. 4F		
Gra_133		Ghory & Siddiqui	[181]	2007	Fig. 7G		
Gra_134		Widyastuti	[441]	2016	Fig. P. 32		
Gra_135		Kurian and Apreshgi	[268]	2020	Im. 5		
Gra_136		Kurian and Apreshgi	[268]	2020	Im. 6		
Gra_137		Kurian and Apreshgi	[268]	2020	Im. 7		
Gra_138		Ebrahimi et al.	[142]	2016	Fig. 2		
Gra_139		Ebrahimi et al.	[142]	2016	Fig. 3		
Gra_140		Ebrahimi et al.	[142]	2016	Fig. 4		
Gra_141		Ebrahimi et al.	[142]	2016	Fig. 5		
Gra_144		Crosnier	[127]	1965	Pl. I Fig. 1		
Gra_145		Crosnier	[127]	1965	Pl. I Fig. 2		
Gra_146		Crosnier	[127]	1965	Pl. II Fig. 1		
Gra_147		Crosnier	[127]	1965	Pl. II Fig. 2		
Gra_148		Crosnier	[127]	1965	Pl. III Fig. 1		
Gra_149		Ximenez	[458]	2020	Fig. 1		
Gra_151		Cely-Moque	[106]	2018	Fig. 5C		
Gra_152		Cely-Moque	[106]	2018	Fig. 5D		
Gra_154		Moraes	[330]	2019	Pl. 1		
Gra_155		Freire et al.	[163]	2010	Fig. 1a		
Gra_156		Arai et al.	[69]	2017	Fig. 2E		
Gra_157		Teschima	[427]	2012	Fig. P. 58 d		
Gra_158		Ramirez & Haroun	[373]	2014	Fig. 2 (oben)		
Gra_159		Ramirez & Haroun	[373]	2014	Fig. 2 (unten)		
Gra_162		Cetin	[105]	2015	Fig. 1.4.		

no	fossil age	author	Reference number	year	figure	page	link
Gra_163		Flores et al.	[157]	1998	Fig. 1A		
Gra_170		Crocetta et al.	[125]	2011	Fig. 1A		
Gra_171		Crocetta et al.	[125]	2011	Fig. 1B		
Gra_172		Crocetta et al.	[125]	2011	Fig. 1C		
Gra_173		Crocetta et al.	[125]	2011	Fig. 1D		
Gra_175		Arruda & Abrunhosa	[71]	2011	Fig. 7		
Gra_176		Arruda & Abrunhosa	[71]	2011	Fig. 20		
Gra_177		Arruda & Abrunhosa	[71]	2011	Fig. 30 II		
Gra_178		Arruda & Abrunhosa	[71]	2011	Fig. 30 III		
Gra_179		Arruda & Abrunhosa	[71]	2011	Fig. 30 IV		
Gra_180		Arruda & Abrunhosa	[71]	2011	Fig. 30 V		
Gra_181		Arruda & Abrunhosa	[71]	2011	Fig. 30 VI		
Gra_182		Arruda & Abrunhosa	[71]	2011	Fig. 30 VII		
Gra_183		Garcia	[176]	1994	Fig. 2		
Gra_184		Cházaro-Olvera & Rocha-Ramírez	[113]	2007	Fig. 2A		
Gra_185		Diele et al.	[138]	2010	Fig. 16.1a		
Gra_186		Diele et al.	[138]	2010	Fig. 16.1a		
Gra_187		Diele et al.	[138]	2010	Fig. 16.1a		
Gra_188		Cuesta et al.	[131]	2016	Fig. 2C		
Gra_189		Chace & Hobbs	[109]	1969	Fig. 48		
Gra_190		Chace & Hobbs	[109]	1969	Fig. 49		
Gra_191		Chace & Hobbs	[109]	1969	Fig. 50		
Gra_192		Chace & Hobbs	[109]	1969	Fig. 51		
Gra_193		Kensley	[241]	1970	Fig. 1a		

no	fossil age	author	Reference number	year	figure	page	link
Gra_194		Chace	[108]	1966	Fig. 9		
Gra_195		Manning & Chace	[298]	1990	Fig. 43a		
Gra_196		De Man	[298]	1909	Pl. xviii Fig. 2		
Gra_197		Wicksten	[440]	2011	Fig. p. 388		
Gra_198		Wicksten	[440]	2011	Fig. p. 388		
Gra_199		Wicksten	[440]	2011	Fig. p. 388		
Gra_207		Cuesta and Rodriguez	[129]	2000	1C		https://link.springer.com/article/10.1023/A:1026576614590
Gra_208		Lago	[269]	1987	4A		https://journals.co.za/doi/abs/10.10520/AJA00445096_1047
Gra_209		Lago	[269]	1987	5A		https://journals.co.za/doi/abs/10.10520/AJA00445096_1047
Gra_210		Lago	[269]	1987	6A		https://journals.co.za/doi/abs/10.10520/AJA00445096_1047
Gra_211		Lago	[269]	1987	7A		https://journals.co.za/doi/abs/10.10520/AJA00445096_1047
Gra_213		Gore and Scotto	[194]	1982	2a		https://repository.si.edu/bitstream/handle/10088/8744/SMS-Gore-1982.pdf

no	fossil age	author	Reference number	year	figure	page	link
Gra_214		Boschi et al	[95]	1967	10a		https://aquadocs.org/bitstream/handle/1834/23373/BolIBM_12.pdf?sequence=1&isAllowed=y
Gra_215		Boschi et al	[95]	1967	10b		https://aquadocs.org/bitstream/handle/1834/23373/BolIBM_12.pdf?sequence=1&isAllowed=y
Gra_216		Boschi et al	[95]	1967	10c		https://aquadocs.org/bitstream/handle/1834/23373/BolIBM_12.pdf?sequence=1&isAllowed=y
Gra_217		Kornienko et al.	[264]	2008	1A"		
Gra_218		Cuesta et al.	[130]	2011	Fig. 8A		
Hi_001		Uribe et al.	[431]	2013	*Emerita analoga* (Stimpson, 1857)		
Hi_002		Puls	[369]	2001	fig. 18C		
Hi_003		Scelzo	[405]	2004	fig. 1B		
Hi_004		Gomalanon	[184]	2016	รูปที่6ง (Fig6ง)		
Hi_005		Sankolli	[396]	1965	fig. 1A		
Hi_006		Harvey et al.	[207]	2014	fig. 53.6B		
Hi_007		Harvey et al.	[207]	2014	fig. 53.1C		
Hi_008		Siddiqi & Ghory	[415]	2006	fig. 2A		
Hi_009		Harvey et al.	[207]	2014	fig. 53.1D		
Hi_010		Siddiqi & Ghory	[415]	2006	fig. 4A		
Hi_011		Siddiqi & Ghory	[415]	2006	fig. 5A		
Hi_012		Siddiqi & Ghory	[415]	2006	fig. 6A		
Hi_013		Schmitt	[406]	1935	fig. 72B		

no	fossil age	author	Reference number	year	figure	page	link
Hi_014		Knight	[248]	1967	fig. 36		
Hi_015		Knight	[248]	1967	fig. 7		
Hi_016		Knight	[248]	1967	fig. 8		
Hi_017		Fonghoy	[158]	2015	รูปที่. 37A (fig. 37A)		
Hi_018		Fonghoy	[158]	2015	รูปที่. 25A (fig. 25A)		
Hi_019		Fonghoy	[158]	2015	รูปที่. 27A (fig. 27A)		
Hi_020		Fonghoy	[158]	2015	รูปที่. 29A (fig. 29A)		
Hi_021		Fonghoy	[158]	2015	รูปที่. 31A (fig. 31A)		
Hi_022		Fonghoy	[158]	2015	รูปที่. 33A (fig. 33A)		
Hi_023		Fonghoy	[158]	2015	รูปที่. 35A (fig. 35A)		
Hi_024		Hsueh	[224]	2015	fig. 2A		
Hi_025		Gomalanon	[184]	2016	รูปที่. 9 (fig. 9)		
Hi_026		Boyko and McLauglin	[98]	2010	fig. 1G		
Hi_027		Borradaile	[94]	1902	fig. 1A		
Hi_028		Haig et al.	[205]	1986	fig. 1A		
Hi_029		Boyko and McLauglin	[98]	2010	fig. 1H		
Hi_030		Boyko and McLauglin	[98]	2010	fig. 1I		
Hi_031		Miers	[325]	1878	fig. 3		
Hi_032		Boyko and McLauglin	[98]	2010	fig. 1J		
Hi_033		Kato & Suzuki	[239]	1992	図. 7A (fig. 7A)		
Hi_034		Miers	[325]	1878	fig. 7		
Hi_035		Miers	[325]	1878	fig. 8		

no	fossil age	author	Reference number	year	figure	page	link
Hi_036		Braig et al.	[99]	2021	Fig. 4A–B		
Hi_037		Braig et al.	[99]	2021	Fig. 4C–D		
Hi_038		Braig et al.	[99]	2021	Fig. 3E–F		
Hi_039		Braig et al.	[99]	2021	Fig. 3G–I		
Hi_040		Braig et al.	[99]	2021	Fig. 3J–L		
Hi_041		Braig et al.	[99]	2021	Fig. 3A–D		
Hi_042		Braig et al.	[99]	2021	Fig. 4E–H		
Hi_043		Braig et al.	[99]	2021	Fig. 1		
Hi_044		Rudolf et al.	[389]	2016	fig. 5		
Hi_045		Rudolf et al.	[389]	2016	fig. 5		
Hi_046		Rudolf et al.	[389]	2016	fig. 5		
Hi_047		Rudolf et al.	[389]	2016	fig. 5		
Hi_048		Rudolf et al.	[389]	2016	fig. 5		
Hi_049		Rudolf et al.	[389]	2016	fig. 5		
Hi_050		Rudolf et al.	[389]	2016	fig. 5		
Hi_051		Abdelsalam & Ramadan	[60]	2017	fig. 2A		
Hi_052		Seridji	[412]	1988	fig. 1A		
Hi_053		Seridji	[412]	1988	fig. 2A		

no	fossil age	author	Reference number	year	figure	page	link
Hi_054		Seridji	[412]	1988	fig. 3A		
Hi_055		Boyko and McLauglin	[98]	2010	fig. 1A		
Hi_056		Boyko and McLauglin	[98]	2010	fig. 1B		
Hi_057		Mashar et al.	[305]	2015	fig. 3A		
Hi_058		Efford & Haig	[145]	1968	fig. 1		
Hi_059		Efford & Haig	[145]	1968	fig. 5		
Hi_060		Faulkes	[149]	2017	fig. 5B (right)		
Hi_061		Faulkes	[149]	2017	fig. 5B (middle)		
Hi_062		Faulkes	[149]	2017	fig. 5B (left)		
Hi_063		Harvey et al.	[207]	2014	fig. 53.6A		
Hi_064		Stuck & Truesdale	[420]	1986	fig. 1B		
Hi_065		Stuck & Truesdale	[420]	1986	fig. 2A		
Hi_066		Stuck & Truesdale	[420]	1986	fig. 3A		
Hi_067		Stuck & Truesdale	[420]	1986	fig. 4B		
Hi_068		Boyko and McLauglin	[98]	2010	fig. 1C		
Hi_069		Boyko and McLauglin	[98]	2010	fig. 1D		
Hi_070		Harvey et al.	[207]	2014	fig. 53.1A		
Hi_071		Efford & Haig	[145]	1968	fig. 8		
Hi_072		Braig et al.	[99]	2021	Fig. 2G–H		
Hi_073		Braig et al.	[99]	2021	Fig. 2A–C		

no	fossil age	author	Reference number	year	figure	page	link
Hi_074		Braig et al.	[99]	2021	Fig. 2D–F		
Hi_075		Schmitt	[406]	1942	fig. 1		
Hi_076		Schmitt	[406]	1942	fig. 3		
Hi_077		Harvey et al.	[207]	2014	fig. 53.6C		
Hi_078		Shen	[413]	1949	Plate XIV		
Hi_079		Boyko and McLauglin	[98]	2010	fig. 1E		
Hi_080		Harvey et al.	[207]	2014	fig. 53.1E		
Hi_081		Johnson and Lewis	[230]	1942	Plate IV, fig. 1		
Hi_082		Milne-Edwards & Lucas	[326]	1841	Plate XXVIII, fig. 1		
Hi_083		Duruflé	[141]	1889	*Blephacopoda Japonica* (Duru.) [sic]		
Hi_084		Konishi	[257]	1987a	fig. 7A	p. 25	
Hom_101		Herrick	[216]	1895	*fig. 36*	pl. 23	
Hom_118		Herrick	[216]	1895	*fig. 37*	pl. 24	
Hom_119		Herrick	[216]	1895	*fig. 9*	p. 261	
Hom_125		Herrick	[216]	1895	*fig. 1*	pl. XXVIII	
Hom_126		Holthuis	[221]	1991	*fig. 106*	p. 58	
Hom_143		Nichols & Lawton		1978	*fig. 2*	p. 237	
Hom_160		Nichols & Lawton	[346]	1978	*fig. 3*	p. 238	
Hom_161		Nichols & Lawton	[346]	1978	*fig. 4*	p. 238	
Hom_162		Nichols & Lawton	[346]	1978	*fig. 5*	p. 239	
Hom_163		Holthuis	[221]	1991	*fig. 110*	p. 60	
Hom_165		Lieven et al.	[288]	2021	*fig. 2 left*	p. 4	
Hom_193		Holthuis	[221]	1991	*fig. 140*	p. 72	
Hom_194		Holthuis	[221]	1991	*fig. 164*	p. 85	
Hom_300		Wear	[436]	1976	fig. 2A	p. 116	

no	fossil age	author	Reference number	year	figure	page	link
Hom_301		Chang et al.	[112]	2020	fig. 1A	p. 42	10.3897/zookeys.1008.59966
Hom_302		Chang et al.	[112]	2020	fig. 2A	p. 47	10.3897/zookeys.1008.59967
Hom_304		Macpherson	[295]	1990	fig. 5A	p. 304	
Hom_305		Macpherson	[295]	1990	fig. 5B	p. 304	
Hom_306		Macpherson	[295]	1990	fig. 5C	p. 304	
Hom_307		Macpherson	[295]	1990	fig. 5D	p. 304	
Hom_308		Macpherson	[295]	1990	fig. 5E	p. 304	
Hom_309		Macpherson	[295]	1990	fig. 5F	p. 304	
Hom_310		Macpherson	[295]	1990	fig. 15A	p. 321	
Hom_311		Macpherson	[295]	1990	fig. 15C	p. 321	
Hom_312		Chang & Chan	[110]	2019	fig. 5	p. 51	
Hom_313		Chang & Chan	[110]	2019	fig. 6	p. 51	
Hom_314		Huang & Kawai	[225]	2020	fig. 2B	p. 190	
Hom_315		Heasman and Jeffs	[214]	2019	fig. 3	p. 4	
Hom_324		Chang et al.	[111]	2015	fig. 1B	p. 597	
Hom_325		Ahyong	[62]	2010	fig. 4B	p. 533	
Hom_326		Tshudy	[429]	2007	fig. 1	p. 464	
Hom_40		Holthuis	[221]	1991	*fig. 40*	p. 27	
Hom_44		Holthuis	[221]	1991	*fig. 63*	p. 36	
Hom_54		Holthuis	[221]	1991	*fig. 80*	p. 45	
Hom_78		Holthuis	[221]	1991	*fig. 88*	p. 49	
Hom_87		Herrick	[216]	1895	*fig. 7*	pl. 6	
jur_002		van Bakel et al.	[75]	2021	fig. 3B		
jur_003		van Bakel et al.	[75]	2021	fig. 3D		
jur_004		van Bakel et al.	[75]	2021	fig. 3F		
jur_005		van Bakel et al.	[75]	2021	fig. 3G		
jur_006		Scholtz	[397]	2014	fig. 6		

no	fossil age	author	Reference number	year	figure	page	link
jur_007		Krobicki and Zaton	[267]	2016	fig. 2		
jur_008		Schweigert	[400]	2019	fig.2		
jur_009		Schweigert and Koppka	[401]	2011	fig.2		
jur_010		Schweigert and Koppka	[401]	2011	fig.3 A		
jur_011		Schweigert and Koppka	[401]	2011	fig.3 B		
jur_012		Schweigert and Koppka	[401]	2011	fig.4 A		
jur_013		Schweigert and Koppka	[401]	2011	fig.5 A		
jur_014		Schweigert and Koppka	[401]	2011	fig.6 B		
jur_015		Schweigert and Koppka	[401]	2011	fig.6 D		

no	fossil age	author	Reference number	year	figure	page	link
jur_016		Schweigert and Koppka	[401]	2011	fig.6 E		
jur_017		Schweigert and Koppka	[401]	2011	fig. 8		
jur_018		Schweigert and Koppka	[401]	2011	fig. 9 A		
jur_019		Schweigert and Robins	[402]	2016	fig. 2 A		
jur_020		Schweigert and Robins	[402]	2016	fig. 2 B		
jur_021		Schweigert and Robins	[402]	2016	fig. 2 C		
jur_022		Schweitzer & Feldmann	[404]	2007	plate 2, A		https://link.springer.com/content/pdf/10.1007/BF03043911.pdf
jur_023		Schweitzer & Feldmann	[404]	2007	plate 2, C		https://link.springer.com/content/pdf/10.1007/BF03043911.pdf
jur_024		Schweitzer & Feldmann	[404]	2007	plate 2, E		https://link.springer.com/content/pdf/10.1007/BF03043911.pdf
jur_025		Schweitzer & Feldmann	[404]	2007	plate 2, F		https://link.springer.com/content/pdf/10.1007/BF03043911.pdf
jur_026		Schweitzer & Feldmann	[404]	2007	plate 2, G		https://link.springer.com/content/pdf/10.1007/BF03043911.pdf

no	fossil age	author	Reference number	year	figure	page	link
jur_027		Schweitzer & Feldmann	[404]	2007	plate 3, A		https://link.springer.com/content/pdf/10.1007/BF03043911.pdf
jur_028		Schweitzer & Feldmann	[404]	2007	plate 3, K		https://link.springer.com/content/pdf/10.1007/BF03043911.pdf
jur_029		Schweitzer & Feldmann	[404]	2007	plate 3, O		https://link.springer.com/content/pdf/10.1007/BF03043911.pdf
jur_030		Schweitzer & Feldmann	[404]	2007	plate 3, P		https://link.springer.com/content/pdf/10.1007/BF03043911.pdf
jur_031		Schweitzer & Feldmann	[404]	2007	plate 3, R		https://link.springer.com/content/pdf/10.1007/BF03043911.pdf
jur_032		Schweitzer & Feldmann	[404]	2007	plate 4, F		https://link.springer.com/content/pdf/10.1007/BF03043911.pdf
jur_033		Schweitzer & Feldmann	[404]	2007	plate 4, B		https://link.springer.com/content/pdf/10.1007/BF03043911.pdf
jur_034		Schweitzer & Feldmann	[404]	2007	plate 6, I		https://link.springer.com/content/pdf/10.1007/BF03043911.pdf
jur_035		Schweitzer & Feldmann	[404]	2007	plate 4, H		https://link.springer.com/content/pdf/10.1007/BF03043911.pdf

no	fossil age	author	Reference number	year	figure	page	link
jur_036		Schweitzer & Feldmann	[404]	2007	plate 4, K		https://link.springer.com/content/pdf/10.1007/BF03043911.pdf
jur_037		Haug and Haug	[207]	2014			
jur_038		Haug et al.	[212]	2015			
jur_039		Scholtz	[398]	2020			
Lit_001		Campodonico	[104]	1979	fig. 1B		
Lit_002		Campodonico	[104]	1979	fig, 2B		
Lit_003		Campodonico	[104]	1979	fig. 3A		
Lit_004		McLaughlin et al.	[452]	2001	fig. 1A		
Lit_005		McLaughlin et al.	[452]	2001	fig. 1B		
Lit_006		McLaughlin et al.	[452]	2001	fig. 1D		
Lit_007		Watts	[433]	2006	fig. 1B		
Lit_008		Watts	[433]	2006	fig. 2B		
Lit_009		Watts	[433]	2006	fig. 3B		
Lit_010		Watts	[433]	2006	fig. 4B		
Lit_011		Kim and Hong	[243]	2000	fig. 2A		
Lit_012		Kim and Hong	[243]	2000	fig. 3A		
Lit_013		Kim and Hong	[243]	2000	fig. 4A		
Lit_014		Kim and Hong	[243]	2000	fig. 5A		
Lit_015		Kim and Hong	[243]	2000	fig. 6B		
Lit_016		Kim and Hong	[245]	2010	fig. 1A		
Lit_017		Kim and Hong	[245]	2010	fig. 2A		
Lit_018		Kim and Hong	[245]	2010	fig. 3A		
Lit_019		Kim and Hong	[245]	2010	fig. 4A		
Lit_020		Kim and Hong	[245]	2010	fig. 5A		
Lit_021		Haynes	[213]	1993	fig. 1B		
Lit_022		Hart	[210]	1965	fig. 1 I		
Lit_023		Hart	[210]	1965	fig. 1II		
Lit_024		Hart	[210]	1965	fig. 1III		
Lit_025		Hart	[210]	1965	fig. 1IV		

no	fossil age	author	Reference number	year	figure	page	link
Lit_026		Hart	[210]	1965	fig. 3		
Lit_027		Hart	[210]	1965	fig. 3		
Lit_028		Harvey et al.	[207]	2014	fig. 53.1 EE		
Lit_029		Harvey et al.	[207]	2014	fig. 53.7 H		
Lit_030		Crain and McLaughlin	[124]	2000	fig. 11		
Lit_031		Crain and McLaughlin	[124]	2000	fig. 14		
Lit_032		McLaughlin et al.	[453]	2001	fig. 1A		
Lit_033		McLaughlin et al.	[453]	2001	fig. 1B		
Lit_034		Duguid and Page	[140]	2009	fig. 8A		
Lit_035		Duguid and Page	[140]	2009	fig. 11E		
Pag_0001		Brodie and Harvey	[100]	2001	fig. 2A	718	https://doi.org/10.1163/20021975-99990169
Pag_0003		Brodie and Harvey	[100]	2001	fig. 2B	718	https://doi.org/10.1163/20021975-99990169
Pag_0005		Brodie and Harvey	[100]	2001	fig. 2C	718	https://doi.org/10.1163/20021975-99990169
Pag_0007		Brodie and Harvey	[100]	2001	fig. 2D	718	https://doi.org/10.1163/20021975-99990169
Pag_0009		Brodie and Harvey	[100]	2001	fig. 2E	718	https://doi.org/10.1163/20021975-99990169
Pag_0011		Brodie and Harvey	[100]	2001	fig. 2F	718	https://doi.org/10.1163/20021975-99990169
Pag_0014		Rahayu et al.	[372]	2016	fig. 1A	473	

no	fossil age	author	Reference number	year	figure	page	link
Pag_0018							https://cdn.floridamuseum.ufl.edu/IZ/5337a2cb-c0b8-4c2a-b86e-4de0588340f0/
Pag_0019							https://cdn.floridamuseum.ufl.edu/IZ/41389835-bbff-4be3-820c-8e511be23b04/
Pag_0020		Reese and Kinzie	[376]	1968	fig. 1.1	124	
Pag_0021		Reese and Kinzie	[376]	1968	fig. 1.2	124	
Pag_0022		Reese and Kinzie	[376]	1968	fig. 1.3	124	
Pag_0023		Reese and Kinzie	[376]	1968	fig. 1.4	124	
Pag_0024		Reese and Kinzie	[376]	1968	fig. 1.5	124	
Pag_0030		Arria Belli					https://commons.wikimedia.org/wiki/File:Birgus_latro.jpg
Pag_0031		Bookhout	[90]	1972	fig. 1.I	217	
Pag_0032		Bookhout	[90]	1972	fig. 1.II	217	
Pag_0033		Bookhout	[90]	1972	fig. 1.III	217	
Pag_0034		Bookhout	[90]	1972	fig. 1.IV	217	
Pag_0040		Bookhout	[90]	1972	fig. 12	230	
Pag_0042		Kornienko and Korn	[262]	2006	fig. 1B	370	
Pag_0044		Kornienko and Korn	[262]	2006	fig. 2B	371	
Pag_0046		Kornienko and Korn	[262]	2006	fig. 3B	372	
Pag_0048		Kornienko and Korn	[262]	2006	fig. 4B	373	
Pag_0050		Kornienko and Korn	[262]	2006	fig. 6A	375	

no	fossil age	author	Reference number	year	figure	page	link
Pag_0052		Kim and Hong	[244]	2005	fig. 2A	93	
Pag_0054		Kim and Hong	[244]	2005	fig. 3A	94	
Pag_0056		Kim and Hong	[244]	2005	fig. 4A	95	
Pag_0058		Kim and Hong	[244]	2005	fig. 5A	96	
Pag_0060		Kim and Hong	[244]	2005	fig. 6A	97	
Pag_0062		Gherardi and McLaughlin	[180]	1995	fig. 1A	260	
Pag_0065		Gherardi and McLaughlin	[180]	1995	fig. 3A	264	
Pag_0067		Gherardi and McLaughlin	[180]	1995	fig. 4A	265	
Pag_0069		Gherardi and McLaughlin	[180]	1995	fig. 5A	267	
Pag_0072		Gherardi and McLaughlin	[180]	1995	fig. 8A	271	
Pag_0075		Bidle and McLaughlin	[84]	1992	fig. 2A	228	
Pag_0089							https://cdn.floridamuseum.ufl.edu/IZ/785a7066-c2db-4b91-99a0-b71ffe56fd9f/
Pag_0090							https://cdn.floridamuseum.ufl.edu/IZ/00192973-5aaa-43cb-a01d-b45fa1d2e74c/
Pag_0091		McLaughlin et al.	[318]	1993	fig. 2B	324	
Pag_0093		McLaughlin et al.	[318]	1993	fig. 2C	324	
Pag_0095		McLaughlin et al.	[318]	1993	fig. 2D	324	
Pag_0097		McLaughlin et al.	[318]	1993	fig. 2E	324	
Pag_0099		McLaughlin et al.	[318]	1993	fig. 2F	324	
Pag_0101		McLaughlin et al.	[318]	1993	fig. 2G	324	
Pag_0102		McLaughlin et al.	[318]	1993	fig. 2H	324	

no	fossil age	author	Reference number	year	figure	page	link
Pag_0103		McLaughlin et al.	[318]	1993	fig. 2I	324	
Pag_0111		McLaughlin et al.	[315]	1991a	fig. 1b	279	
Pag_0113		McLaughlin et al.	[315]	1991a	fig. 1c	279	
Pag_0115		McLaughlin et al.	[315]	1991a	fig. 1d	279	
Pag_0117		McLaughlin et al.	[315]	1991a	fig. 1e	279	
Pag_0119		McLaughlin et al.	[315]	1991a	fig. 1f	279	
Pag_0122		Macdonald et al.	[291]	1957	fig. 2a	221	
Pag_0127		Macdonald et al.	[291]	1957	fig. 2f	221	
Pag_0129		Macdonald et al.	[291]	1957	fig. 4a	227	
Pag_0134		Macdonald et al.	[291]	1957	fig. 4f	227	
Pag_0136		Macdonald et al.	[291]	1957	fig. 5a	230	
Pag_0141		Macdonald et al.	[291]	1957	fig. 5f	230	
Pag_0143		Macdonald et al.	[291]	1957	fig. 6a	233	
Pag_0148		Macdonald et al.	[291]	1957	fig. 6f	233	
Pag_0150		Macdonald et al.	[291]	1957	fig. 7a	236	
Pag_0152		Macdonald et al.	[291]	1957	fig. 8a	238	
Pag_0157		Macdonald et al.	[291]	1957	fig. 8f	238	
Pag_0159		Macdonald et al.	[291]	1957	fig. 9a	242	
Pag_0164		Macdonald et al.	[291]	1957	fig. 9f	242	
Pag_0166		Macdonald et al.	[291]	1957	fig. 10a	244	
Pag_0171		Macdonald et al.	[291]	1957	fig. 10f	244	
Pag_0173		McLaughlin and Gore	[309]	1988	fig. 1a	263	
Pag_0174		McLaughlin and Gore	[309]	1988	fig. 1d	263	
Pag_0181		Hong	[222]	1969	fig. 1I	4	
Pag_0182		Hong	[222]	1969	fig. 1.II	4	
Pag_0183		Hong	[222]	1969	fig. 1.III	4	
Pag_0184		Hong	[222]	1969	fig. 1.IV	4	
Pag_0189		Hong	[222]	1969	fig. 6A	10	
Pag_0193		Roberts	[383]	1970	fig. 1A	190	

no	fossil age	author	Reference number	year	figure	page	link
Pag_0195		Roberts	[383]	1970	fig. 2A	192	
Pag_0197		Roberts	[383]	1970	fig. 3A	193	
Pag_0199		Roberts	[383]	1970	fig. 4A	195	
Pag_0202		Provenzano and Rice	[368]	1964	fig. 1.I	219	
Pag_0203		Provenzano and Rice	[368]	1964	fig. 1.II	219	
Pag_0204		Provenzano and Rice	[368]	1964	fig. 1.III	219	
Pag_0205		Provenzano and Rice	[368]	1964	fig. 1.IV	219	
Pag_0219							https://cdn.floridamuseum.ufl.edu/IZ/1ad4d5e0-14a7-4252-89a8-c202c2aaed1e
Pag_0220							https://specify-attachments.science.ku.dk/static/NHMD_Invertebrate_Zoology/originals/sp6398514199057378545.att.tif
Pag_0221							https://science.mnhn.fr/institution/mnhn/collection/iu/item/2013-5330
Pag_0222							https://science.mnhn.fr/institution/mnhn/collection/iu/item/2013-6763

no	fossil age	author	Reference number	year	figure	page	link
Pag_0223							https://www.gbif.org/occurrence/3455227312
Pag_0228		Kim et al.	[246]	2007	fig. 2a	330	
Pag_0230		Kim et al.	[246]	2007	fig. 3a	333	
Pag_0232		Kim et al.	[246]	2007	fig. 4a	335	
Pag_0234		Kim et al.	[246]	2007	fig. 5a	336	
Pag_0236		Kim et al.	[246]	2007	fig. 6a	338	
Pag_0239		Kornienko and Korn	[263]	2007	fig. 1b	33	https://doi.org/10.1080/07924259.2007.9652224
Pag_0241		Kornienko and Korn	[263]	2007	fig. 2b	34	https://doi.org/10.1080/07924259.2007.9652224
Pag_0244		Kornienko and Korn	[263]	2007	fig. 5a	38	https://doi.org/10.1080/07924259.2007.9652224
Pag_0246		Hall	[206]	1972	fig. 1.I	12	https://scholarlycommons.pacific.edu/uop_etds/1779
Pag_0247		Hall	[206]	1972	fig. 1.II	12	https://scholarlycommons.pacific.edu/uop_etds/1779
Pag_0248		Hall	[206]	1972	fig. 1.III	12	https://scholarlycommons.pacific.edu/uop_etds/1779
Pag_0249		Hall	[206]	1972	fig. 1.IV	12	https://scholarlycommons.pacific.edu/uop_etds/1779
Pag_0254		Hall	[206]	1972	fig. 7	18	https://scholarlycommons.pacific.edu/uop_etds/1779
Pag_0256		Hall	[206]	1972	fig. 9.I	28	https://scholarlycommons.pacific.edu/uop_etds/1779

no	fossil age	author	Reference number	year	figure	page	link
Pag_0257		Hall	[206]	1972	fig. 9.II	28	https://scholarlycommons.pacific.edu/uop_etds/1779
Pag_0259		Hall	[206]	1972	fig. 9.IV	28	https://scholarlycommons.pacific.edu/uop_etds/1779
Pag_0264		Hall	[206]	1972	fig. 15	34	https://scholarlycommons.pacific.edu/uop_etds/1779
Pag_0267		Hall	[206]	1972	fig. 17.II	45	https://scholarlycommons.pacific.edu/uop_etds/1779
Pag_0268		Hall	[206]	1972	fig. 17.III	45	https://scholarlycommons.pacific.edu/uop_etds/1779
Pag_0269		Hall	[206]	1972	fig. 17.IV	45	https://scholarlycommons.pacific.edu/uop_etds/1779
Pag_0274		Hall	[206]	1972	fig. 23	51	https://scholarlycommons.pacific.edu/uop_etds/1779
Pag_0276		Samuelsen	[395]	1972	fig. 1a	4	https://doi.org/10.1080/00364827.1972.10411192
Pag_0280		Goldstein and Bookhout	[183]	1972	fig. 1.I	264	https://doi.org/10.1163/156854072X00165
Pag_0281		Goldstein and Bookhout	[183]	1972	fig. 1.II	264	https://doi.org/10.1163/156854072X00165
Pag_0282		Goldstein and Bookhout	[183]	1972	fig. 1.III	264	https://doi.org/10.1163/156854072X00165
Pag_0283		Goldstein and Bookhout	[183]	1972	fig. 1.IV	264	https://doi.org/10.1163/156854072X00165

no	fossil age	author	Reference number	year	figure	page	link
Pag_0290		McLaughlin et al.	[317]	1992	fig. 1a	509	https://research.nhm.org/pdfs/19686/19686.pdf
Pag_0291		McLaughlin et al.	[317]	1992	fig. 1b	509	https://research.nhm.org/pdfs/19686/19686.pdf
Pag_0292		McLaughlin et al.	[317]	1992	fig. 1c	509	https://research.nhm.org/pdfs/19686/19686.pdf
Pag_0293		McLaughlin et al.	[317]	1992	fig. 1d	509	https://research.nhm.org/pdfs/19686/19686.pdf
Pag_0294		McLaughlin et al.	[317]	1992	fig. 1e	509	https://research.nhm.org/pdfs/19686/19686.pdf
Pag_0295		McLaughlin et al.	[317]	1992	fig. 1f	509	https://research.nhm.org/pdfs/19686/19686.pdf
Pag_0296		McLaughlin et al.	[317]	1992	fig. 1g	509	https://research.nhm.org/pdfs/19686/19686.pdf
Pag_0306		Squires	[418]	1996	fig. 1b	45	https://citeseerx.ist.psu.edu/viewdoc/download?doi=10.1.1.515.6363&rep=rep1&type=pdf
Pag_0308		Squires	[418]	1996	fig. 2b	47	https://citeseerx.ist.psu.edu/viewdoc/download?doi=10.1.1.515.6363&rep=rep1&type=pdf

no	fossil age	author	Reference number	year	figure	page	link
Pag_0310		Squires	[418]	1996	fig. 3b	49	https://citeseerx.ist.psu.edu/viewdoc/download?doi=10.1.1.515.6363&rep=rep1&type=pdf
Pag_0312		Squires	[418]	1996	fig. 4b	51	https://citeseerx.ist.psu.edu/viewdoc/download?doi=10.1.1.515.6363&rep=rep1&type=pdf
Pag_0314		Squires	[418]	1996	fig. 5b	53	https://citeseerx.ist.psu.edu/viewdoc/download?doi=10.1.1.515.6363&rep=rep1&type=pdf
Pag_0316		McLaughlin and Gore	[310]	1992	fig. 2b	450	https://doi.org/10.2307/1549039
Pag_0318		McLaughlin and Gore	[310]	1992	fig. 3a	452	https://doi.org/10.2307/1549039
Pag_0320		McLaughlin and Gore	[310]	1992	fig. 4a	453	https://doi.org/10.2307/1549039
Pag_0322		McLaughlin and Gore	[310]	1992	fig. 5a	455	https://doi.org/10.2307/1549039
Pag_0324		McLaughlin and Gore	[310]	1992	fig. 6a	456	https://doi.org/10.2307/1549039
Pag_0332		Fitch and Lindgren	[155]	1979	fig. 1e	79	
Pag_0333		Fitch and Lindgren	[155]	1979	fig. 1f	79	
Pag_0334		Fitch and Lindgren	[155]	1979	fig. 1g	79	
Pag_0335		Fitch and Lindgren	[155]	1979	fig. 1h	79	

no	fossil age	author	Reference number	year	figure	page	link
Pag_0336		Fitch and Lindgren	[155]	1979	fig. 1j	79	
Pag_0337		McLaughlin et al.	[314]	1989	fig. 1b	628	https://doi.org/10.1163/193724089X00638
Pag_0339		McLaughlin et al.	[314]	1989	fig. 2a	630	https://doi.org/10.1163/193724089X00638
Pag_0342		McLaughlin et al.	[314]	1989	fig. 4a	632	https://doi.org/10.1163/193724089X00638
Pag_0348		McLaughlin et al.	[314]	1989	fig. 7a	637	https://doi.org/10.1163/193724089X00638
Pag_0352		McLaughlin et al.	[316]	1991b	fig. 1a	417	https://doi.org/10.2307/1548467
Pag_0353		McLaughlin et al.	[316]	1991b	fig. 1b	417	https://doi.org/10.2307/1548467
Pag_0354		McLaughlin et al.	[316]	1991b	fig. 1c	417	https://doi.org/10.2307/1548467
Pag_0355		McLaughlin et al.	[316]	1991b	fig. 1d	417	https://doi.org/10.2307/1548467
Pag_0356		McLaughlin et al.	[316]	1991b	fig. 1e	417	https://doi.org/10.2307/1548467
Pag_0388		Komai	[255]	2009	fig. 1a	199	
Pag_0392		McLaughlin	[307]	1997	fig. 1b	441	
Pag_0394		McLaughlin	[307]	1997	fig. 2c	445	
Pag_0396		McLaughlin	[307]	1997	fig. 4a	450	
Pag_0398		McLaughlin	[307]	1997	fig. 5a	452	
Pag_0402		McLaughlin	[307]	1997	fig. 6a	456	
Pag_0405		McLaughlin	[307]	1997	fig. 7a	459	
Pag_0409		McLaughlin	[307]	1997	fig. 8a	462	
Pag_0412		McLaughlin	[307]	1997	fig. 9a	466	

no	fossil age	author	Reference number	year	figure	page	link
Pag_0416		McLaughlin	[307]	1997	fig. 10f	472	
Pag_0418		McLaughlin	[307]	1997	fig. 11h	475	
Pag_0420		McLaughlin	[307]	1997	fig. 12a	480	
Pag_0424		McLaughlin	[307]	1997	fig. 13c	484	
Pag_0426		McLaughlin	[307]	1997	fig. 13e	484	
Pag_0428		McLaughlin	[307]	1997	fig. 14a	486	
Pag_0432		McLaughlin	[307]	1997	fig. 15e	490	
Pag_0434		McLaughlin	[307]	1997	fig. 16d	493	
Pag_0438		McLaughlin	[307]	1997	fig. 17a	497	
Pag_0440		McLaughlin	[307]	1997	fig. 17b	497	
Pag_0441		McLaughlin	[307]	1997	fig. 18a	500	
Pag_0443		McLaughlin	[307]	1997	fig. 18b	500	
Pag_0445		McLaughlin	[307]	1997	fig. 19a	503	
Pag_0446		McLaughlin	[307]	1997	fig. 19b	503	
Pag_0448		McLaughlin	[307]	1997	fig. 20a	507	
Pag_0450		McLaughlin	[307]	1997	fig. 20b	507	
Pag_0452		McLaughlin	[307]	1997	fig. 20d	507	
Pag_0454		McLaughlin	[307]	1997	fig. 21a	513	
Pag_0456		McLaughlin	[307]	1997	fig. 22	516	
Pag_0460		McLaughlin	[307]	1997	fig. 23a	518	
Pag_0464		McLaughlin	[307]	1997	fig. 24a	523	
Pag_0466		McLaughlin	[307]	1997	fig. 24b	523	
Pag_0468		McLaughlin	[307]	1997	fig. 25a	528	
Pag_0470		McLaughlin	[307]	1997	fig. 26a	531	
Pag_0474		McLaughlin	[307]	1997	fig. 27a	534	
Pag_0476		McLaughlin	[307]	1997	fig. 29a	540	
Pag_0478		McLaughlin	[307]	1997	fig. 30a	544	
Pag_0480		McLaughlin	[307]	1997	fig. 30b	544	
Pag_0482		McLaughlin	[307]	1997	fig. 31a	548	

no	fossil age	author	Reference number	year	figure	page	link
Pag_0488		Lemaitre and McLaughlin	[285]	1992	fig. 6A	755	https://doi.org/10.1080/00222939200770471
Pag_0492		Lemaitre and McLaughlin	[285]	1992	fig. 9A	763	https://doi.org/10.1080/00222939200770471
Pag_0495		Lemaitre	[278]	1989	fig. 3A	14	ISSN 0024-1652
Pag_0499		Lemaitre	[278]	1989	fig. 7A	22	ISSN 0024-1652
Pag_0500		Lemaitre	[278]	1989	fig. 10A	25	ISSN 0024-1652
Pag_0501		Lemaitre	[278]	1989	fig. 13A	31	ISSN 0024-1652
Pag_0504		Lemaitre	[278]	1989	fig. 15A	39	ISSN 0024-1652
Pag_0510		Lemaitre	[278]	1989	fig. 24A	53	ISSN 0024-1652
Pag_0513		Lemaitre	[278]	1989	fig. 36A	72	ISSN 0024-1652
Pag_0517		Lemaitre	[282]	1998	fig. 2A	292	
Pag_0533		Lemaitre	[279]	1990	fig. 1A	222	https://doi.org/10.1080/00222939000770141
Pag_0537		Lemaitre	[279]	1990	fig. 2A	224	https://doi.org/10.1080/00222939000770141
Pag_0541		Lemaitre	[279]	1990	fig. 3A	226	https://doi.org/10.1080/00222939000770141
Pag_0550		Lemaitre	[281]	1997	fig. 3A	581	
Pag_0554		Lemaitre	[280]	1996	fig. 1A	168	
Pag_0558		Lemaitre	[280]	1996	fig. 2A	171	
Pag_0561		Lemaitre	[280]	1996	fig. 6A	175	
Pag_0565		Lemaitre	[280]	1996	fig. 12A	187	
Pag_0568		Lemaitre	[280]	1996	fig. 15A	191	
Pag_0571		Lemaitre	[280]	1996	fig. 17A	196	
Pag_0576		Lemaitre	[280]	1996	fig. 19A	200	
Pag_0581		Lemaitre	[280]	1996	fig. 21A	203	
Pag_0585		Lemaitre	[280]	1996	fig. 22A	205	

no	fossil age	author	Reference number	year	figure	page	link
Pag_0589		Lemaitre	[280]	1996	fig. 24A	210	
Pag_0592		Lemaitre	[280]	1996	fig. 25A	212	
Pag_0596		Lemaitre	[280]	1996	fig. 27A	215	
Pag_0600		Lemaitre	[280]	1996	fig. 28A	216	
Pag_0604		Jung and Park	[233]	2022	fig. 2A	10	
Pag_0609		McLaughlin and Lemaitre	[312]	2008	fig. 5A	63	
Pag_0611		Konishi and Imafuku	[259]	2000	fig. 2A	67	
Pag_0613		McLaughlin and Lemaitre	[313]	2009	fig. 3A	172	
Pag_0614		McLaughlin and Lemaitre	[313]	2009	fig. 4A	175	
Pag_0617		McLaughlin and Lemaitre	[313]	2009	fig. 4B	175	
Pag_0620		McLaughlin and Lemaitre	[313]	2009	fig. 5A	181	
Pag_0623		McLaughlin and Lemaitre	[313]	2009	fig. 5B	181	
Pag_0626		McLaughlin and Lemaitre	[313]	2009	fig. 6A	185	
Pag_0629		McLaughlin and Lemaitre	[313]	2009	fig. 6B	185	
Pag_0632		McLaughlin and Lemaitre	[313]	2009	fig. 7A	187	
Pag_0635		McLaughlin and Lemaitre	[313]	2009	fig. 10A	197	
Pag_0638		McLaughlin and Lemaitre	[313]	2009	fig. 11A	201	
Pag_0641		McLaughlin and Lemaitre	[313]	2009	fig. 13A	206	
Pag_0645		McLaughlin and Lemaitre	[313]	2009	fig. 16A	220	
Pag_0648		McLaughlin and Lemaitre	[313]	2009	fig. 17	224	

no	fossil age	author	Reference number	year	figure	page	link
Pag_0651		McLaughlin and Lemaitre	[311]	2001	fig. 2A	1065	https://doi.org/10.1163/20021975-99990198
Pag_0655		Lemaitre and Campos	[284]	1993	fig. 3A	560	
Pag_0660		Lemaitre	[283]	1999	fig. 10A	323	
Pag_0664		Lemaitre	[283]	1999	fig. 12A	326	
Pag_0668		Lemaitre	[283]	1999	fig. 16A	331	
Pag_0700		Williamson and Levetzow	[442]	1967	fig. 1A	180	
Pag_0711		Williamson and Levetzow	[442]	1967	fig. 3I	186	
Pag_0716		Williamson and Levetzow	[442]	1967	fig. 4H	189	
Pce_001		Gore and Abele	[193]	1973	fig.1	https://www.ingentaconnect.com/content/umrsmas/bullmar/1973/00000023/00000003/art00008#	
Pce_002		Gore and Abele	[193]	1973	fig.2	https://www.ingentaconnect.com/content/umrsmas/bullmar/1973/00000023/00000003/art00008#	
Pce_003		Gore and Abele	[449]	1969	fig.2c	https://www.brill.com/view/journals/cr/18/1/article-p75_8.xml?ebody=previewpdf-60547	

no	fossil age	author	Reference number	year	figure	page	link
Pce_004		Greewnwood	[196]	1965	fig.7a	https://brill.com/view/journals/cr/8/3/article-p285_6.xml?ebody=article%20details	
Pce_005		Greewnwood	[196]	1965	fig.7b	https://brill.com/view/journals/cr/8/3/article-p285_6.xml?ebody=article%20details	
Pce_006		Gore	[186]	1971a	fig.2c	https://www.journals.uchicago.edu/doi/epdf/10.2307/1540263	
Pce_007		Albornoz and Wehrtmann	[64]	1996	fig.2	https://d1wqtxts1xzle7.cloudfront.net	
Pce_008		Albornoz and Wehrtmann	[64]	1996	fig.2	https://d1wqtxts1xzle7.cloudfront.net	
Pce_009		Konishi	[258]	1987b	fig.2e	https://academic.oup.com/jcb/article-abstract/7/3/481/2327636?login=false	
Pce_010		Konishi	[258]	1987b	fig.2a	https://academic.oup.com/jcb/article-abstract/7/3/481/2327636?login=false	

no	fossil age	author	Reference number	year	figure	page	link
Pce_011		Konishi	[258]	1987b	fig.2b	https://academic.oup.com/jcb/article-abstract/7/3/481/2327636?login=false	
Pce_012		Gore	[187]	1971b	fig.3c	https://scholarspace.manoa.hawaii.edu/server/api/core/bitstreams/290c6831-1ce5	
Pce_013		Gore	[187]	1971b	fig.4c	https://scholarspace.manoa.hawaii.edu/server/api/core/bitstreams/290c6831-1ce5	
Pce_014		Wehrtmann et al.	[438]	1997	fig.2c	https://brill.com/view/journals/cr/70/5/article-p562_5.xml?ebody=article%20details	
Pce_015		Knight	[247]	1966	fig.6	https://brill.com/view/journals/cr/10/1/article-p75_9.xml?ebody=article%20details	
Pce_016		Knight	[247]	1966	fig.43	https://brill.com/view/journals/cr/10/1/article-p75_9.xml?ebody=article%20details	

no	fossil age	author	Reference number	year	figure	page	link
Pce_017		Fujita et al.	[172]	2002	fig.5a	https://academic.oup.com/jcb/article/22/3/567/2679750?login=false	
Pce_018		Gore	[191]	1977	fig.2c	https://brill.com/view/journals/cr/33/3/article-p284_7.xml?ebody=article%20details	
Pce_019		Werding and Müller	[439]	1990	fig.2c	https://link.springer.com/content/pdf/10.1007/BF02365473.pdf	
Pce_020		Hernández et al.	[217]	1998	fig.3e	http://crustacea.org.br/wp-content/uploads/2014/02/nauplius-v06n1a09.Hernandez.et_.al_.pdf	
Pce_021		MacMillan	[293]	1972	fig.5	https://www.journals.uchicago.edu/doi/epdf/10.2307/1540246	
Pce_022		Pellegrini and Gamba	[363]	1985	fig.3d	https://brill.com/view/journals/cr/49/1-3/article-p251_45.xml?ebody=article%20details	
Pce_023		Hernández et al.	[218]	2002	fig.1c	https://academic.oup.com/jcb/article/22/1/113/2664357?login=false	

no	fossil age	author	Reference number	year	figure	page	link
Pce_024		Osawa and McLaughlin	[351]	2010	fig.1b	https://lkcnhm.nus.edu.sg/wp-content/uploads/sites/10/app/uploads/2017/04/s23rbz109-129.pdf	
Pce_025		Osawa and McLaughlin	[351]	2010	fig.1d	https://lkcnhm.nus.edu.sg/wp-content/uploads/sites/10/app/uploads/2017/04/s23rbz109-129.pdf	
Pce_026		Osawa and McLaughlin	[351]	2010	fig.1e	https://lkcnhm.nus.edu.sg/wp-content/uploads/sites/10/app/uploads/2017/04/s23rbz109-129.pdf	
Pce_027		Osawa and McLaughlin	[351]	2010	fig.1f	https://lkcnhm.nus.edu.sg/wp-content/uploads/sites/10/app/uploads/2017/04/s23rbz109-129.pdf	
Pce_028		Osawa and McLaughlin	[351]	2010	fig.1g	https://lkcnhm.nus.edu.sg/wp-content/uploads/sites/10/app/uploads/2017/04/s23rbz109-129.pdf	
Pce_029		Osawa and McLaughlin	[351]	2010	fig.1h	https://lkcnhm.nus.edu.sg/wp-content/uploads/sites/10/app/uploads/2017/04/s23rbz109-129.pdf	

no	fossil age	author	Reference number	year	figure	page	link
Pce_030		Osawa and McLaughlin	[351]	2010	fig.1i	https://lkcnhm.nus.edu.sg/wp-content/uploads/sites/10/app/uploads/2017/04/s23rbz109-129.pdf	
Pce_031		Osawa and McLaughlin	[351]	2010	fig.2a	https://lkcnhm.nus.edu.sg/wp-content/uploads/sites/10/app/uploads/2017/04/s23rbz109-129.pdf	
Pce_032		Osawa and McLaughlin	[351]	2010	fig.2b	https://lkcnhm.nus.edu.sg/wp-content/uploads/sites/10/app/uploads/2017/04/s23rbz109-129.pdf	
Pce_033		Osawa and McLaughlin	[351]	2010	fig.2c	https://lkcnhm.nus.edu.sg/wp-content/uploads/sites/10/app/uploads/2017/04/s23rbz109-129.pdf	
Pce_034		Osawa and McLaughlin	[351]	2010	fig.2d	https://lkcnhm.nus.edu.sg/wp-content/uploads/sites/10/app/uploads/2017/04/s23rbz109-129.pdf	
Pce_035		Osawa and McLaughlin	[351]	2010	fig.2e	https://lkcnhm.nus.edu.sg/wp-content/uploads/sites/10/app/uploads/2017/04/s23rbz109-129.pdf	

no	fossil age	author	Reference number	year	figure	page	link
Pce_036		Osawa and McLaughlin	[351]	2010	fig.2f	https://lkcnhm.nus.edu.sg/wp-content/uploads/sites/10/app/uploads/2017/04/s23rbz109-129.pdf	
Pce_037		Osawa and McLaughlin	[351]	2010	fig.2g	https://lkcnhm.nus.edu.sg/wp-content/uploads/sites/10/app/uploads/2017/04/s23rbz109-129.pdf	
Pce_038		Gore	[189]	1973	fig.4a	https://docserver.ingentaconnect.com	
Pce_039		Yaqoob	[445]	1977	fig.1c	https://brill.com/view/journals/cr/32/3/article-p241_3.xml?Tab%20Menu=article-metadata	
Pce_040		Gore	[190]	1975	fig.5	https://repository.si.edu/bitstream/handle/10088/8737/SMS-Gore-1975.pdf	
Pce_041		Rodriguez et al.	[387]	2004	fig.4a	https://academic.oup.com/jcb/article/24/2/291/2670409?login=false	
Pce_042		Garcia-Guerrero et al.	[177]	2005	fig.1c	https://www.cambridge.org/core/	
Pce_043		Müller and Werding	[334]	1990	fig.2c	https://www.vliz.be/imisdocs/publications/286809.pdf	

no	fossil age	author	Reference number	year	figure	page	link
Pce_044		Rodriguez et al.	[388]	2005	fig.1a	https://repository.si.edu/bitstream/handle/10088/3954/Rodriguez_Hernandez_and_Felder_2005.pdf	
Pce_045		Rodriguez et al.	[388]	2005	fig.1b	https://repository.si.edu/bitstream/handle/10088/3954/Rodriguez_Hernandez_and_Felder_2005.pdf	
Pce_046		Rodriguez et al.	[388]	2005	fig.1c	https://repository.si.edu/bitstream/handle/10088/3954/Rodriguez_Hernandez_and_Felder_2005.pdf	
Pce_047		Rodriguez et al.	[388]	2005	fig.1d	https://repository.si.edu/bitstream/handle/10088/3954/Rodriguez_Hernandez_and_Felder_2005.pdf	
Pce_048		Rodriguez et al.	[388]	2005	fig.1e	https://repository.si.edu/bitstream/handle/10088/3954/Rodriguez_Hernandez_and_Felder_2005.pdf	
Pce_049		Rodriguez et al.	[388]	2005	fig.1f	https://repository.si.edu/bitstream/handle/10088/3954/Rodriguez_Hernandez_and_Felder_2005.pdf	

no	fossil age	author	Reference number	year	figure	page	link
Pce_050		Rodriguez et al.	[388]	2005	fig.1g	https://repository.si.edu/bitstream/handle/10088/3954/Rodriguez_Hernandez_and_Felder_2005.pdf	
Pce_051		Rodriguez et al.	[388]	2005	fig.1h	https://repository.si.edu/bitstream/handle/10088/3954/Rodriguez_Hernandez_and_Felder_2005.pdf	
Pce_052		Rodriguez et al.	[388]	2005	fig.1i	https://repository.si.edu/bitstream/handle/10088/3954/Rodriguez_Hernandez_and_Felder_2005.pdf	
Pce_053		Garcia-Guerrero et al.	[179]	2006	fig.1c	https://d1wqtxts1xzle7.cloudfront.net	
Pce_054		Saelzer et al.	[392]	1986	fig.5a	https://www.researchgate.net/profile	
Pce_055		Gonor and Gonor	[185]	1973	fig.6a	https://books.google.de/books?hl=	
Pce_056		Gonor and Gonor	[185]	1973	fig.6d	https://books.google.de/books?hl=	
Pce_057		Gonor and Gonor	[185]	1973	fig.13a	https://books.google.de/books?hl=	
Pce_058		Gonor and Gonor	[185]	1973	fig.13d	https://books.google.de/books?hl=	
Pce_059		Gonor and Gonor	[185]	1973	fig.4	https://books.google.de/books?hl=	
Pce_060		Yaqoob	[446]	1979a	fig.1b	https://www.jstor.org/stable/25027485	

no	fossil age	author	Reference number	year	figure	page	link
Pce_061		Retamal and Santa Cruz	[377]	2018	fig.2a	https://www.scielo.cl/pdf/lajar/v46n4/0718-560X-lajar-46-04-0820.pdf	
Pce_062		Retamal and Santa Cruz	[377]	2018	fig.4a	https://www.scielo.cl/pdf/lajar/v46n4/0718-560X-lajar-46-04-0820.pdf	
Pce_063		Pinheiro et al.	[364]	2017	fig.1d	https://repositorio.unesp.br/bitstream/handle/11449/174235/2-s2.0-85013106533.pdf?sequence=1	
Pce_064		Werding	[437]	2001	fig.1	https://www.researchgate.net	
Pce_065		Werding	[437]	2001	fig.3	https://www.researchgate.net	
Pce_066		Yaqoob	[447]	1979b	fig.1c	https://brill.com/view/journals/cr/37/3/article-p253_4.xml?Tab%20Menu=article-metadata	
Pce_067		Hiller et al.	[219]	2010	fig.3	http://www.crustacea.org.br/wp-content/uploads/2014/02/nauplius-v18n1a10.Hiller.et_.al_.pdf	

no	fossil age	author	Reference number	year	figure	page	link
Pce_068		Paul et al.	[358]	1993	fig.1a	https://brill.com/view/journals/cr/65/3/article-p346_7.xml?Tab%20Menu=article-metadata	
Pce_069		Paul et al.	[358]	1993	fig.2aII	https://brill.com/view/journals/cr/65/3/article-p346_7.xml?Tab%20Menu=article-metadata	
Pce_070		Paul et al.	[358]	1993	fig.2aIII	https://brill.com/view/journals/cr/65/3/article-p346_7.xml?Tab%20Menu=article-metadata	
Pce_071		Paul et al.	[358]	1993	fig.3aI	https://brill.com/view/journals/cr/65/3/article-p346_7.xml?Tab%20Menu=article-metadata	
Pce_072		Paul et al.	[358]	1993	fig.4aII	https://brill.com/view/journals/cr/65/3/article-p346_7.xml?Tab%20Menu=article-metadata	
Pce_073		Paul et al.	[358]	1993	fig.4aIII	https://brill.com/view/journals/cr/65/3/article-p346_7.xml?Tab%20Menu=article-metadata	

no	fossil age	author	Reference number	year	figure	page	link
Pce_074		Paul et al.	[358]	1993	fig.4aIV	https://brill.com/view/journals/cr/65/3/article-p346_7.xml?Tab%20Menu=article-metadata	
Pce_075		Gore	[188]	1972	fig.2c	https://docserver.ingentaconnect.com/deliver	
Pce_076		Ng et al.	[345]	2012	fig.1a	https://www.researchgate.net/profile/	
Pce_077		Ng et al.	[345]	2012	fig.1b	https://www.researchgate.net/profile/	
Pce_078		Ng et al.	[345]	2012	fig.1d	https://www.researchgate.net/profile/	
Pce_079		Ng et al.	[345]	2012	fig.2a	https://www.researchgate.net/profile/	
Pce_080		Ferreira and Tavares	[153]	2017	fig.1a	https://www.researchgate.net/profile/	
Pce_081		Ferreira and Tavares	[153]	2017	fig.1b	https://www.researchgate.net/profile/	
Pce_082		Ferreira and Tavares	[153]	2017	fig.1d	https://www.researchgate.net/profile/	
Pce_083		Ferreira and Tavares	[153]	2017	fig.2a	https://www.researchgate.net/profile/	

no	fossil age	author	Reference number	year	figure	page	link
Pce_084		Ferreira and Tavares	[153]	2017	fig.4a	https://www.researchgate.net/profile/	
Pce_085		Ferreira and Tavares	[153]	2017	fig.5a	https://www.researchgate.net/profile/	
Pce_086		Ferreira and Tavares	[153]	2017	fig.5b	https://www.researchgate.net/profile/	
Pce_087		Ferreira and Tavares	[153]	2017	fig.5c	https://www.researchgate.net/profile/	
Pce_088		Ferreira and Tavares	[153]	2017	fig.5e	https://www.researchgate.net/profile/	
Port_001	-	Fielder et al.	[154]	1984	fig. 1e		
Port_002	-	Marco-Herrero et al.	[302]	2021	fig. 1a		
Port_003	-	Marco-Herrero et al.	[302]	2021	fig. 1c		
Port_004	-	Marco-Herrero et al.	[302]	2021	fig. 1d		
Port_005	-	Marco-Herrero et al.	[302]	2021	fig. 1e		
Port_006	-	Marco-Herrero et al.	[302]	2021	fig. 1f		
Port_007	-	Marco-Herrero et al.	[302]	2021	fig. 1g		
Port_008	-	Marco-Herrero et al.	[302]	2021	fig. 1h		

no	fossil age	author	Reference number	year	figure	page	link
Port_009	-	Marco-Herrero et al.	[302]	2021	fig. 1i		
Port_010	-	Krishnan and Kannupandi	[265]	1990	fig. 6A		
Port_011	-	Krishnan and Kannupandi	[265]	1990	fig. 8A		
Port_012	-	Dineen et al.	[139]	2001	fig. 24a		
Port_013	-	Dineen et al.	[139]	2001	fig. 24b		
Port_014	-	Dineen et al.	[139]	2001	fig. 24c		
Port_015	-	Greenwood and Fielder	[197]	1983	fig. 1I		
Port_016	-	Stuck and Truesdale	[420]	1988	fig. 11A		
Port_017	-	Stuck and Truesdale	[420]	1988	fig. 13D		
Port_018	-	Stuck and Truesdale	[420]	1988	fig. 13D		
Port_019	-	Stuck and Truesdale	[420]	1988	fig. 13D		
Port_020	-	Lyskin et al.	[290]	2001	fig. 1a		
Port_021	-	Lyskin et al.	[290]	2001	fig. 2k		
Port_022	-	Islam et al.	[227]	2000	fig. 7a		
Port_023	-	Juwana et al.	[234]	1987	fig. 9a		
Port_024	-	Islam et al.	[228]	2005	fig. 6A		
Port_025	-	Negreiros-Fransozo et al.	[340]	2007	fig. 1		
Port_026	-	Negreiros-Fransozo et al.	[340]	2007	fig. 6		
Port_027	-	Bolla Jr. et al.	[88]	2014	fig. 1		
Port_028	-	Al-Aidaroos et al.	[63]	2019	fig. 1e		
Port_029	-	Dan et al.	[132]	2016	fig. 1a		
Port_030	-	Bolla Jr. et al.	[89]	2016	fig. 1A		
Port_031	-	Bolla Jr. et al.	[89]	2016	fig. 6A2		

no	fossil age	author	Reference number	year	figure	page	link
Port_032	-	Bolla Jr. et al.	[89]	2016	fig. 6A3		
Port_033	-	Bolla Jr. et al.	[88]	2014	fig. 5a2		
Port_034	-	Bolla Jr. et al.	[88]	2014	fig. 5a3		
Port_035	-	Greenwood and Fielder	[197]	1979	fig. 1d		
Port_036	-	Bolla Jr.	[86]	2010	fig. 11.1		https://repositorio.unesp.br/bitstream/handle/11449/99437/bollajunior_ea_me_botib.pdf?sequence=1
Port_037	-	Bolla Jr.	[86]	2010	fig. 11.2		https://repositorio.unesp.br/bitstream/handle/11449/99437/bollajunior_ea_me_botib.pdf?sequence=1
Port_038	-	Bolla Jr.	[86]	2010	fig. 11.3		https://repositorio.unesp.br/bitstream/handle/11449/99437/bollajunior_ea_me_botib.pdf?sequence=1
Port_039	-	Bolla Jr.	[86]	2010	fig. 19A		https://repositorio.unesp.br/bitstream/handle/11449/99437/bollajunior_ea_me_botib.pdf?sequence=1
Port_040	-	Bolla Jr.	[86]	2010	fig. 28.2		https://repositorio.unesp.br/bitstream/handle/11449/99437/bollajunior_ea_me_botib.pdf?sequence=1

no	fossil age	author	Reference number	year	figure	page	link
Port_041	-	Bolla Jr.	[86]	2010	fig. 28.3		https://repositorio.unesp.br/bitstream/handle/11449/99437/bollajunior_ea_me_botib.pdf?sequence=1
Port_042	-	Bolla Jr. et al.	[87]	2008	fig. 2a		
Port_043	-	Josileen and Menon	[230]	2004	fig. 7a		
Port_044	-	Bookhout and Costlow Jr.	[91]	1974	fig. 11b		
Port_045	-	Effendy et al.	[144]	2022	fig. 1a		
Port_046	-	Gamô and Muraoka	[174]	1977	Plate I, fig. 7		
Port_047	-	Gamô and Muraoka	[174]	1977	Plate II, fig. 22		
Port_048	-	Gamô and Muraoka	[174]	1977	Plate III, fig. 28		
Port_049	-	Gamô and Muraoka	[174]	1977	Plate III, fig. 40		
Port_050	-	Knight	[250]	2014	fig. 20		https://aquila.usm.edu/cgi/viewcontent.cgi?article=1015&context=masters_theses
Port_051	-	Knight	[250]	2014	fig. 31		https://aquila.usm.edu/cgi/viewcontent.cgi?article=1015&context=masters_theses
Port_052	-	Knight	[250]	2014	fig. 37		https://aquila.usm.edu/cgi/viewcontent.cgi?article=1015&context=masters_theses

no	fossil age	author	Reference number	year	figure	page	link
Port_053	-	Knight	[250]	2014	fig. 40		https://aquila.usm.edu/cgi/viewcontent.cgi?article=1015&context=masters_theses
Port_054	-	Knight	[250]	2014	fig. 55D		https://aquila.usm.edu/cgi/viewcontent.cgi?article=1015&context=masters_theses
Port_055	-	Rice and Kristensen	[382]	1982	fig. 1A		
Port_056	-	Knight	[250]	2014	fig. 58		https://aquila.usm.edu/cgi/viewcontent.cgi?article=1015&context=masters_theses
Port_057	-	Costlow and Bookhout	[122]	1959	fig. 9e		
Port_058	-	Bookhout and Costlow Jr.	[92]	1977	fig. 11b		
Port_059	-	Ogburn et al.	[349]	2011	fig. 3		
Port_060	-	Ogburn et al.	[349]	2011	fig. 3		
Port_061	-	Ogburn et al.	[349]	2011	fig. 5a		
Port_062	-	Ogburn et al.	[349]	2011	fig. 5b		
Port_063	-	Ogburn et al.	[349]	2011	fig. 5c		
Port_064	-	Ogburn et al.	[349]	2011	fig. 5e		
Port_065	-						https://www.gbif.org/occurrence/1456347738
Port_066	-						https://www.gbif.org/occurrence/1993787138

no	fossil age	author	Reference number	year	figure	page	link
Port_067	-						https://www.gbif.org/occurrence/1931545251
Port_068	-						https://science.mnhn.fr/institution/mnhn/collection/iu/item/2019-5447
Port_069	-						https://science.mnhn.fr/institution/mnhn/collection/iu/item/2013-2470
Port_070	-						https://www.gbif.org/occurrence/3028092029
Port_071	-						https://www.gbif.org/occurrence/3028055592
Port_072	-						https://science.mnhn.fr/institution/mnhn/collection/iu/item/2013-738
Port_073	-						https://science.mnhn.fr/institution/mnhn/collection/iu/item/2013-5196?listIndex=1&listCount=2
Port_074	-						https://science.mnhn.fr/institution/mnhn/collection/iu/item/2019-5245

no	fossil age	author	Reference number	year	figure	page	link
Port_075	-						https://collections.nmnh.si.edu/search/iz/?ark=ark:/65665/33a43e3b55cd14cff95554d5536747bac
Port_076	-						https://www.gbif.org/occurrence/3398651328
Port_077	-	Ondřej Radosta					https://crabdatabase.info/en/crabs/brachyura/eubrachyura/heterotremata/portunoidea/portunidae/portunus/portunus-pelagicus-13271
Port_078	-						https://www.gbif.org/occurrence/1135623717
Port_079	-	Bruno Ogata					https://crabdatabase.info/en/crabs/brachyura/eubrachyura/heterotremata/portunoidea/portunidae/achelous/achelous-spinimanus-3313
Port_080	-						https://www.gbif.org/occurrence/1324560973
Port_081	-						https://www.gbif.org/occurrence/350708879

no	fossil age	author	Reference number	year	figure	page	link
Port_082	-						https://www.gbif.org/occurrence/2447149864
Port_083	-						https://www.gbif.org/occurrence/1944631098
Port_084	-	Ondřej Radosta					https://crabdatabase.info/en/crabs/brachyura/eubrachyura/heterotremata/portunoidea/portunidae/portunus/portunus-trituberculatus-13263
Port_085	-						https://www.gbif.org/occurrence/1272739795
Port_086	-						https://www.gbif.org/occurrence/1135625095
Port_087	-						https://science.mnhn.fr/institution/mnhn/collection/iu/item/2013-2533
Port_088	-						https://science.mnhn.fr/institution/mnhn/collection/iu/item/2019-5397
Port_089	-						https://science.mnhn.fr/institution/mnhn/collection/iu/item/2014-8211

no	fossil age	author	Reference number	year	figure	page	link
Port_090	-						https://science.mnhn.fr/institution/mnhn/collection/iu/item/2014-8332
Port_091	-	Ondřej Radosta					https://crabdatabase.info/en/crabs/brachyura/eubrachyura/heterotremata/portunoidea/portunidae/callinectes/callinectes-sapidus-3288
Port_092	-						https://collections.nmnh.si.edu/search/iz/?ark=ark:/65665/3823584cad60e4f8f9d59adc53675d8d8
Port_093	-						https://www.gbif.org/occurrence/2447155276
Port_094	-						https://science.mnhn.fr/institution/mnhn/collection/iu/item/2008-11852?listIndex=10&listCount=495
Port_095	-						https://science.mnhn.fr/institution/mnhn/collection/iu/item/2008-11268?listIndex=8&listCount=495

no	fossil age	author	Reference number	year	figure	page	link
Port_096	-						https://science.mnhn.fr/institution/mnhn/collection/iu/item/2008-10693?listIndex=7&listCount=495
Port_097	-						https://www.gbif.org/occurrence/1931545190
Port_098	-						https://science.mnhn.fr/institution/mnhn/collection/iu/item/2014-8369
Port_099	-						https://science.mnhn.fr/institution/mnhn/collection/iu/item/2013-6819
Port_100	-						https://www.gbif.org/occurrence/2433415027
Port_101	-						https://www.gbif.org/occurrence/1675371870
Port_102	-						https://www.gbif.org/occurrence/3382933440
Port_103	-						https://www.gbif.org/occurrence/3382947368
Port_104	-						https://www.gbif.org/occurrence/3382950338
Port_105	-						https://science.mnhn.fr/institution/mnhn/collection/iu/item/2019-5462

no	fossil age	author	Reference number	year	figure	page	link
Port_106	-						https://www.gbif.org/occurrence/1213351317
Port_107	-						https://www.gbif.org/occurrence/1944631227
Port_108	-						https://www.gbif.org/occurrence/899673043
Port_109	-						https://www.gbif.org/occurrence/2857972306
Port_110	-						https://www.gbif.org/occurrence/2857980321
Port_111	-						https://science.mnhn.fr/institution/mnhn/collection/iu/item/2013-6860
Port_112	-						https://science.mnhn.fr/institution/mnhn/collection/iu/item/2013-4921
Port_113	-						https://www.gbif.org/occurrence/3382939391
Port_114	-						https://www.gbif.org/occurrence/2433415139
Port_115	-						https://science.mnhn.fr/institution/mnhn/collection/iu/item/2013-633

no	fossil age	author	Reference number	year	figure	page	link
Port_116	-						https://science.mnhn.fr/institution/mnhn/collection/iu/item/2010-7972
Port_117	-						https://science.mnhn.fr/institution/mnhn/collection/iu/item/2010-6268
Port_118	-						https://www.gbif.org/occurrence/1213351389
Port_119	-						https://www.gbif.org/occurrence/899688582
Port_120	-						https://www.gbif.org/occurrence/1213350519
Port_121	-						https://www.gbif.org/occurrence/1213350402
Port_122	-						https://www.gbif.org/occurrence/1213351393
Port_125		Bookhout & Costlow	[91]	1974	fig. 4B	p. 28	
Port_126		Bookhout & Costlow	[91]	1974	fig. 5B	p. 31	
Port_127		Bookhout & Costlow	[91]	1974	fig. 6B	p. 33	
Port_128		Bookhout & Costlow	[91]	1974	fig. 7B	p. 34	
Port_129		Bookhout & Costlow	[91]	1974	fig. 8B	p. 37	
Port_130		Bookhout & Costlow	[91]	1974	fig. 9B	p. 39	

no	fossil age	author	Reference number	year	figure	page	link
Port_131		Bookhout & Costlow	[91]	1974	fig. 10B	p. 40	
Port_132		Bookhout & Costlow	[92]	1977	fig. 3B	p. 709	
Port_133		Bookhout & Costlow	[92]	1977	fig. 4B	p. 712	
Port_134		Bookhout & Costlow	[92]	1977	fig. 5B	p. 713	
Port_135		Bookhout & Costlow	[92]	1977	fig. 6B	p. 716	
Port_136		Bookhout & Costlow	[92]	1977	fig. 7B	p. 718	
Port_137		Bookhout & Costlow	[92]	1977	fig. 8B	p. 720	
Port_138		Bookhout & Costlow	[92]	1977	fig. 9B	p. 721	
Port_139		Bookhout & Costlow	[92]	1977	fig. 10B	p. 722	
Port_140		Costlow and Bookhout	[122]	1959	fig. 1B	p. 376	
Port_141		Costlow and Bookhout	[122]	1959	fig. 2B	p. 377	
Port_142		Costlow and Bookhout	[122]	1959	fig. 3B	p. 378	
Port_143		Costlow and Bookhout	[122]	1959	fig. 4B	p. 379	
Port_144		Costlow and Bookhout	[122]	1959	fig. 5B	p. 381	
Port_145		Costlow and Bookhout	[122]	1959	fig. 6B	p. 382	
Port_146		Costlow and Bookhout	[122]	1959	fig. 7B	p. 383	
Port_147		Costlow and Bookhout	[122]	1959	fig. 8B	p. 384	
Port_148		Dineen et al.	[139]	2001	fig. 4b	p. 780	

no	fossil age	author	Reference number	year	figure	page	link
Port_149		Dineen et al.	[139]	2001	fig. 5a	p. 781	
Port_150		Islam et al.	[228]	2005	fig. 1B	p. 80	
Port_151		Islam et al.	[228]	2005	fig. 2B	p. 81	
Port_152		Islam et al.	[228]	2005	fig. 3B	p. 82	
Port_153		Islam et al.	[228]	2005	fig. 4B	p. 83	
Port_154		Islam et al.	[228]	2005	fig. 5B	p. 85	
Ran_001		Rice and Ingle	[379]	1977	fig. 1	p. 95	
Ran_002		Rice and Ingle	[379]	1977	fig. 2k	p. 96	
Ran_003		Knight	[249]	1968	fig. 5	p. 153	
Ran_004		Knight	[249]	1968	fig. 7	p. 153	
Ran_005		Knight	[249]	1968	fig. 9	p. 153	
Ran_006		Knight	[249]	1968	fig. 11	p. 153	
Ran_007		Knight	[249]	1968	fig. 14	p. 154	
Ran_008		Knight	[249]	1968	fig. 15	p. 154	
Ran_009		Minigawa	[328]	1990	fig. 9A	p. 585	
Ran_010		Minigawa	[328]	1990	fig. 10A	p. 586	
Ran_011		Bruce	[102]	1972	fig. 1		
Ran_012		Davie	[134]	1989	fig. 4a		
Ran_013		Davie	[134]	1989	fig. 7a		
Ran_014		Davie	[134]	1989	fig. 7b		
Ran_015		Feldmannand and Schweitzer	[150]	2007	fig. 1a		https://doi.org/10.2992/0097-4463(2007)76[39:SDIEAE]2.0.CO;2
Ran_016		Feldmannand and Schweitzer	[150]	2007	fig. 1c		https://doi.org/10.2992/0097-4463(2007)76[39:SDIEAE]2.0.CO;2

no	fossil age	author	Reference number	year	figure	page	link
Ran_017		Feldmannand and Schweitzer	[150]	2007	fig. 4a		https://doi.org/10.2992/0097-4463(2007)76[39:SDIEAE]2.0.CO;2
Ran_018		Feldmannand and Schweitzer	[150]	2007	fig. 4c		https://doi.org/10.2992/0097-4463(2007)76[39:SDIEAE]2.0.CO;2
Ran_019		Feldmannand and Schweitzer	[150]	2007	fig. 5a		https://doi.org/10.2992/0097-4463(2007)76[39:SDIEAE]2.0.CO;2
Ran_020		Feldmannand and Schweitzer	[150]	2007	fig. 6a		https://doi.org/10.2992/0097-4463(2007)76[39:SDIEAE]2.0.CO;2
Ran_021		Feldmannand and Schweitzer	[150]	2007	fig. 7a		https://doi.org/10.2992/0097-4463(2007)76[39:SDIEAE]2.0.CO;2
Ran_022		Feldmannand and Schweitzer	[150]	2007	fig. 7c		https://doi.org/10.2992/0097-4463(2007)76[39:SDIEAE]2.0.CO;2
Ran_023		Feldmannand and Schweitzer	[150]	2007	fig. 8		https://doi.org/10.2992/0097-4463(2007)76[39:SDIEAE]2.0.CO;2

no	fossil age	author	Reference number	year	figure	page	link
Ran_024		Feldmannand and Schweitzer	[150]	2007	fig. 9a		https://doi.org/10.2992/0097-4463(2007)76[39:SDIEAE]2.0.CO;2
Ran_025		Feldmannand and Schweitzer	[150]	2007	fig. 9c		https://doi.org/10.2992/0097-4463(2007)76[39:SDIEAE]2.0.CO;2
Ran_026		Kasinathan et al.	[238]	2007	fig. 1		
Ran_027		Matondo & Demayo	[306]	2015	fig. 3a		
Ran_028		Matondo & Demayo	[306]	2015	fig. 4a		
Ran_029		Matondo & Demayo	[306]	2015	fig. 5a		
Ran_030		Matondo & Demayo	[306]	2015	fig. 5b		
Xant_001		Ryan	[390]	1956	fig. 1A		https://www-jstor-org.emedien.ub.uni-muenchen.de/stable/pdf/2422450.pdf
Xant_002		Ryan	[390]	1956	fig. 1B		https://www-jstor-org.emedien.ub.uni-muenchen.de/stable/pdf/2422450.pdf

no	fossil age	author	Reference number	year	figure	page	link
Xant_003		Ryan	[390]	1956	fig. 1C		https://www-jstor-org.emedien.ub.uni-muenchen.de/stable/pdf/2422450.pdf
Xant_004		Ryan	[390]	1956	fig. 2A		https://www-jstor-org.emedien.ub.uni-muenchen.de/stable/pdf/2422450.pdf
Xant_006		Ryan	[390]	1956	2C		https://www-jstor-org.emedien.ub.uni-muenchen.de/stable/pdf/2422450.pdf
Xant_007		Montù	[329]	1988	11A		https://academic-oup-com.emedien.ub.uni-muenchen.de/jcb/article/8/4/594/2327726?login=true
Xant_008		Davie	[135]	1993	1A		https://research.nhm.org/pdfs/15251/15251.pdf
Xant_009		Davie	[135]	1993	2A		https://research.nhm.org/pdfs/15251/15251.pdf
Xant_010		Davie	[135]	1993	3A		https://research.nhm.org/pdfs/15251/15251.pdf

no	fossil age	author	Reference number	year	figure	page	link
Xant_011		Davie	[135]	1993	4A		https://research.nhm.org/pdfs/15251/15251.pdf
Xant_012		Davie	[135]	1993	5A		https://research.nhm.org/pdfs/15251/15251.pdf
Xant_013		Davie	[135]	1993	6A		https://research.nhm.org/pdfs/15251/15251.pdf
Xant_014		Davie	[135]	1993	7A		https://research.nhm.org/pdfs/15251/15251.pdf
Xant_015		Davie	[135]	1993	8A		https://research.nhm.org/pdfs/15251/15251.pdf
Xant_016		Davie	[135]	1993	9A		https://research.nhm.org/pdfs/15251/15251.pdf
Xant_017		Davie	[135]	1993	10A		https://research.nhm.org/pdfs/15251/15251.pdf
Xant_018		Davie	[135]	1993	11A		https://research.nhm.org/pdfs/15251/15251.pdf
Xant_019		Davie	[135]	1993	12A		https://research.nhm.org/pdfs/15251/15251.pdf
Xant_020		Ondřej Radosta					https://crabdatabase.info/en/crabs/brachyura/eubrachyura/heterotremata/xanthoidea/panopeidae/eurytium/eurytium-limosum-2783

no	fossil age	author	Reference number	year	figure	page	link
Xant_021		Ondřej Radosta					https://crabdatabase.info/en/crabs/brachyura/eubrachyura/heterotremata/xanthoidea/panopeidae/panopeus/panopeus-lacustris-2740
Xant_022		Marco-Herrero et al.	[301]	2014	1B		https://hmr.biomedcentral.com/track/pdf/10.1007/s10152-014-0381-8.pdf
Xant_023		Vieira and Rieger	[432]	2004	9A		https://academic.oup.com/plankt/article/26/10/1175/1550152?login=false
Xant_024		Micu	[327]	2010	2B		https://www.cambridge.org/core/journals/marine-biodiversity-records/article/abs/first-record-of-says-mud-crab-dyspanopeus-sayi-brachyura-xanthoidea-panopeidae-from-the-black-sea/19AAE250A391620D6A37DF66AD85BAE6

no	fossil age	author	Reference number	year	figure	page	link
Xant_025		Edmondson	[143]	1962	2A		http://citeseerx.ist.psu.edu/viewdoc/download?doi=10.1.1.730.7322&rep=rep1&type=pdf
Xant_026		Froglia and Speranza	[165]	1993	2A		https://www.researchgate.net/publication/284777981_First_record_of_Dyspanopeus_sayi_Smith_1869_in_the_Mediterranean_Sea_Crustacea_Decapoda_Xanthidae
Xant_027		Marco-Herrero et al.	[302]	2021	5e		https://www.nature.com/articles/s41598-021-99486-4#Fig5
Xant_028		Ng and Yang	[342]	1998	2A		https://www.tandfonline.com/doi/pdf/10.1080/00222939800771201?needAccess=true
Xant_029		Hart	[209]	1935	2A		https://cdnsciencepub.com/doi/abs/10.1139/cjr35-036

no	fossil age	author	Reference number	year	figure	page	link
Xant_030		Arthur Anker					https://crabdatabase.info/en/crabs/brachyura/eubrachyura/heterotremata/xanthoidea/panopeidae/rhithropanopeus/rhithropanopeus-harrisii-2732
Xant_031		Salgado-Barragan	[394]	2005	5A		https://www.tandfonline.com/doi/abs/10.1080/07924259.2005.9652154
Xant_032		Martin	[304]	1988	2A		https://research.nhm.org/pdfs/4120/4120.pdf
Xant_033		Martin	[304]	1988	2C		https://research.nhm.org/pdfs/4120/4120.pdf
Xant_034		Fransozo et al.	[160]	1991	6A		https://www.scielo.br/j/rbzool/a/9mhcCjmxfPMDpj9DRNGSvts/?format=pdf&lang=en
Xant_035		Rodriguez & Martin	[385]	1997	7A		https://doi.org/10.1163/193724097X00142
Xant_036		Rodriguez & Martin	[385]	1997	8		https://doi.org/10.1163/193724097X00142
Xant_037		Marco-Herroro et al	[302]	2021	5A		https://www.nature.com/articles/s41598-021-99486-4.pdf

no	fossil age	author	Reference number	year	figure	page	link
Xant_038		Marco-Herroro et al	[302]	2021	5B		https://www.nature.com/articles/s41598-021-99486-4.pdf
Xant_039		Marco-Herroro et al	[302]	2021	5C		https://www.nature.com/articles/s41598-021-99486-4.pdf
Xant_040		Marco-Herroro et al	[302]	2021	5D		https://www.nature.com/articles/s41598-021-99486-4.pdf
Xant_041		Marco-Herroro et al	[302]	2021	5E		https://www.nature.com/articles/s41598-021-99486-4.pdf
Xant_042		Marco-Herroro et al	[302]	2021	5F		https://www.nature.com/articles/s41598-021-99486-4.pdf
Xant_043		Andryszac & Gore	[66]	1981	6A		https://books.google.de/books?hl=en&lr=&id=BXogAAAAMAAJ&oi=fnd&pg=PA487&dq=xanthidae+shell+megalopa+&ots=503SSUIM-Q&sig=g3sDpl_J44TA-s9zwQwAx6Qau4E&redir_esc=y#v=onepage&q=xanthidae%20shell%20megalopa&f=false

no	fossil age	author	Reference number	year	figure	page	link
Xant_044		Cházaro-Olvera et al.	[114]	2006	2C		https://brill.com/view/journals/cr/79/7/article-p865_6.xml?casa_token=sJUwg_yrrCoAAAAA:c_vIwPydB8Oa6Nh3jrUIQ1x7hNqSxS5fvA1UmCueKuKajn665fafjoPlV2okltS5-MNYmXb4Pw
Xant_045		Rodriguez & Paula	[384]	1993	8A		https://academic.oup.com/jcb/article-abstract/13/2/296/2328146
Xant_047		Marco-Herrero	[299]	2014	fig. 4C	p. 345	https://roderic.uv.es/handle/10550/47114
Xant_048		Marco-Herrero	[299]	2014	fig. 1B	p. 203	https://roderic.uv.es/handle/10550/47114
Xant_050		Cházaro-Olvera et al.	[114]	2006	2D		https://brill.com/view/journals/cr/79/7/article-p865_6.xml?casa_token=sJUwg_yrrCoAAAAA:c_vIwPydB8Oa6Nh3jrUIQ1x7hNqSxS5fvA1UmCueKuKajn665fafjoPlV2okltS5-MNYmXb4Pw

no	fossil age	author	Reference number	year	figure	page	link
Xant_051		Cházaro-Olvera et al.	[114]	2006	2E		https://brill.com/view/journals/cr/79/7/article-p865_6.xml?casa_token=sJUwg_yrrCoAAAAA:c_vIwPydB8Oa6Nh3jrUIQ1x7hNqSxS5fvA1UmCueKuKajn665fafjoPlV2okltS5-MNYmXb4Pw
Xant_052		Cházaro-Olvera et al.	[114]	2006	2F		https://brill.com/view/journals/cr/79/7/article-p865_6.xml?casa_token=sJUwg_yrrCoAAAAA:c_vIwPydB8Oa6Nh3jrUIQ1x7hNqSxS5fvA1UmCueKuKajn665fafjoPlV2okltS5-MNYmXb4Pw
Xant_053		Cházaro-Olvera et al.	[114]	2006	2G		https://brill.com/view/journals/cr/79/7/article-p865_6.xml?casa_token=sJUwg_yrrCoAAAAA:c_vIwPydB8Oa6Nh3jrUIQ1x7hNqSxS5fvA1UmCueKuKajn665fafjoPlV2okltS5-MNYmXb4Pw

no	fossil age	author	Reference number	year	figure	page	link
Xant_054		Cházaro-Olvera et al.	[114]	2006	2H		https://brill.com/view/journals/cr/79/7/article-p865_6.xml?casa_token=sJUwg_yrrCoAAAAA:c_vIwPydB8Oa6Nh3jrUIQ1x7hNqSxS5fvA1UmCueKuKajn665fafjoPlV2okltS5-MNYmXb4Pw
Xant_055		Garcia-Guerrero et al.	[178]	2005	7A		https://digital.csic.es/bitstream/10261/2375/1/Eurypanopeus.pdf
Xant_056		Tanaka and Konishi	[423]	2001	1E		https://www.jstage.jst.go.jp/article/crustacea/30/0/30_KJ00003243456/_article/-char/ja/
Xant_057		Tanaka et al.	[424]	2010	5A		https://www.jstage.jst.go.jp/article/crustacea/39/0/39_KJ00007729123/_pdf/-char/ja
Xant_058		Tanaka et al.	[424]	2010	11A		https://www.jstage.jst.go.jp/article/crustacea/39/0/39_KJ00007729123/_pdf/-char/ja

no	fossil age	author	Reference number	year	figure	page	link
Xant_059		Moraes and Negreira-Fransozo	[331]	2008	2		https://repositorio.unesp.br/bitstream/handle/11449/120051/moraes_jcb_tcc_bot.pdf?sequence=1
Xant_060		Ng et. al	[344]	2008	146 (p. 192)		http://citeseerx.ist.psu.edu/viewdoc/download?doi=10.1.1.649.2033&rep=rep1&type=pdf
Xant_061		Quintana and Takeda	[370]	1988	1A		https://www.kahaku.go.jp/research/publication/zoology/download/14-1/BNSM140103.pdf
Xant_062		Quintana and Takeda	[370]	1988	2H		https://www.kahaku.go.jp/research/publication/zoology/download/14-1/BNSM140103.pdf
Xant_063		Moraes et. al	[331]	2022	7D		http://rev.mex.biodivers.unam.mx/wp-content/uploads/2022-2/vol-93/93-1-feb-2022/3753.pdf

no	fossil age	author	Reference number	year	figure	page	link
Xant_064		Ondřej Radosta					https://crabdatabase.info/en/crabs/brachyura/eubrachyura/heterotremata/xanthoidea/xanthidae/demania/demania-splendida-2224
Xant_065		Hsi-Te Shih					https://crabdatabase.info/en/crabs/brachyura/eubrachyura/heterotremata/xanthoidea/xanthidae/demania/demania-toxica-2223
Xant_066		Ko	[251]	2006	fig. 3A	p. 11	https://www.tandfonline.com/doi/abs/10.1080/17386357.2006.9647278
Xant_067		Tan Heok Hui and Tohru Naruse					https://crabdatabase.info/en/crabs/brachyura/eubrachyura/heterotremata/xanthoidea/xanthidae/cymo/cymo-cerasma-2506
Xant_068		Gustav Paulay and Arthur Anker					https://crabdatabase.info/en/crabs/brachyura/eubrachyura/heterotremata/xanthoidea/xanthidae/cymo/cymo-deplanatus-2505

no	fossil age	author	Reference number	year	figure	page	link
Xant_069		Saba	[391]	1976			https://www.jstage.jst.go.jp/article/rcustacea/7/0/7_KJ00003289372/_pdf/-char/ja
Xant_070		Ondřej Radosta					https://crabdatabase.info/en/crabs/brachyura/eubrachyura/heterotremata/xanthoidea/xanthidae/cymo/cymo-quadrilobatus-2502
Xant_071		Guerao et al.	[202]	2005	2C		https://www.tandfonline.com/doi/abs/10.1080/00222930500256235
Xant_072		Robert Lasley					https://crabdatabase.info/en/crabs/brachyura/eubrachyura/heterotremata/xanthoidea/xanthidae/danielea/danielea-noelensis-2456
Xant_073		Ondřej Radosta		2016			https://crabdatabase.info/en/crabs/brachyura/eubrachyura/heterotremata/xanthoidea/xanthidae/demania/demania-rotundata-2227

no	fossil age	author	Reference number	year	figure	page	link
Xant_074		Ondřej Radosta		2017			https://crabdatabase.info/en/crabs/brachyura/eubrachyura/heterotremata/xanthoidea/xanthidae/demania/demania-scaberrima-2226
Xant_075		Hsi-Te Shih					https://crabdatabase.info/en/crabs/brachyura/eubrachyura/heterotremata/xanthoidea/xanthidae/demania/demania-japonica-2230

no	fossil age	author	Reference number	year	figure	page	link
Xant_076		Siddiqui and Ghory	[414]	1999	fig. 4A	p. 222	https://www.researchgate.net/profile/Farhana-Ghory/publication/269223716_Studies_on_the_complete_larval_development_of_Actaea_jacquelinae_Guinot1976_Decapoda_Brachyura_Xanthidae_reared_in_the_laboratory/links/55a48d1b08aef604aa03e015/Studies-on-the-complete-larval-development-of-Actaea-jacquelinae-Guinot1976-Decapoda-Brachyura-Xanthidae-reared-in-the-laboratory.pdf
Xant_077		Ondřej Radosta					https://crabdatabase.info/en/crabs/brachyura/eubrachyura/heterotremata/xanthoidea/xanthidae/demania/demania-reynaudii-2228

no	fossil age	author	Reference number	year	figure	page	link
Xant_078		Gustav Paulay					https://crabdatabase.info/en/crabs/brachyura/eubrachyura/heterotremata/xanthoidea/xanthidae/epiactaea/epiactaea-nodulosa-2655
Xant_079		Gustav Paulay					https://crabdatabase.info/en/crabs/brachyura/eubrachyura/heterotremata/xanthoidea/xanthidae/etisus/etisus-anaglyptus-2498
Xant_080		Rodriguez and Spivak	[386]	2001	fig. 7A	p. 816	https://academic.oup.com/jcb/article/21/3/806/2679923?login=false
Xant_081		Hsi-Te Shih					https://crabdatabase.info/en/crabs/brachyura/eubrachyura/heterotremata/xanthoidea/xanthidae/demania/demania-cultripes-2233

no	fossil age	author	Reference number	year	figure	page	link
Xant_082		Bookhout and Costlow	[93]	1979	fig. 6B	p. 12	https://www.jstor.org/stable/25027477?casa_token=qCInem5xkQ8AAAAA%3ApCrR6v5gqT-xXeCAfcKECR8-QXUvLsQe0bUHjxMs_VwRsUJ5G0LXhPbAO8wJrun8NE1brYFusjom3oL81yGgjPY73u6aWdEp9UZN3PbEoQame6l0jxU&seq=1
Xant_083		Hsi-Te Shih					https://crabdatabase.info/en/crabs/brachyura/eubrachyura/heterotremata/xanthoidea/xanthidae/demania/demania-intermedia-2231
Xant_084		Ko et al.	[252]	2004	2b		https://academic.oup.com/jcb/article/24/4/637/2670461
Xant_085		Ko et al.	[252]	2004	4b		https://academic.oup.com/jcb/article/24/4/637/2670461

no	fossil age	author	Reference number	year	figure	page	link
Xant_086		Ko et al.	[252]	2004	6b		https://academic.oup.com/jcb/article/24/4/637/2670461
Xant_087		Ko et al.	[252]	2004	8b		https://academic.oup.com/jcb/article/24/4/637/2670461
Xant_088		Ko et al.	[451]	2002	3b		https://www.researchgate.net/publication/264178946_zoeal_Stages_of_Actaea_semblatae_Cruistacea_Decapoda_Xanthidae_with_a_Key_to_the_Known_Xanthid_zoeas_of_Korea
Xant_089		Siddiqui and Ghory	[414]	1999	1b		https://www.researchgate.net/publication/269223716_Studies_on_the_complete_larval_development_of_Actaea_jacquelinae_Guinot1976_Decapoda_Brachyura_Xanthidae_reared_in_the_laboratory

no	fossil age	author	Reference number	year	figure	page	link
Xant_090		Lai et al.	[270]	2011	2a		https://www.researchgate.net/publication/238499961_Xanthidae_MacLeay_1838_Decapoda_Brachyura_Xanthoidea_systematics_A_multi-gene_approach_with_support_from_adult_and_zoeal_morphology
Xant_091		Clark and Galil	[119]	1998	1a		https://sciencepress.mnhn.fr/sites/default/files/articles/pdf/z1998n2a6.pdf#viewer.action=download
Xant_093		Siddiqui and Ghory	[414]	1999	2a		https://www.researchgate.net/publication/269223716_Studies_on_the_complete_larval_development_of_Actaea_jacquelinae_Guinot1976_Decapoda_Brachyura_Xanthidae_reared_in_the_laboratory

no	fossil age	author	Reference number	year	figure	page	link
Xant_094		Siddiqui and Ghory	[414]	1999	3a		https://www.researchgate.net/publication/269223716_Studies_on_the_complete_larval_development_of_Actaea_jacquelinae_Guinot1976_Decapoda_Brachyura_Xanthidae_reared_in_the_laboratory
Xant_096		Costlow and Bookhout	[123]	1961	1b		https://www.jstor.org/stable/pdf/24334445.pdf?refreqid=excelsior%3Aabc460827970b92ee3b71de475b6789&ab_segments=&origin=&acceptTC=1
Xant_097		Clark and Paula	[121]	2003	1a		https://www.researchgate.net/publication/237535784_Descriptions_of_ten_xanthoidean_Crustacea_Decapoda_Brachyura_first_stage_zoeas_from_Inhaca_Island_Mozambique

no	fossil age	author	Reference number	year	figure	page	link
Xant_098		Clark and Paula	[121]	2003	5a		https://www.researchgate.net/publication/237535784_Descriptions_of_ten_xanthoidean_Crustacea_Decapoda_Brachyura_first_stage_zoeas_from_Inhaca_Island_Mozambique
Xant_100		Clark and Paula	[121]	2003	9a		https://www.researchgate.net/publication/237535784_Descriptions_of_ten_xanthoidean_Crustacea_Decapoda_Brachyura_first_stage_zoeas_from_Inhaca_Island_Mozambique
Xant_101		Clark and Paula	[121]	2003	13a		https://www.researchgate.net/publication/237535784_Descriptions_of_ten_xanthoidean_Crustacea_Decapoda_Brachyura_first_stage_zoeas_from_Inhaca_Island_Mozambique

no	fossil age	author	Reference number	year	figure	page	link
Xant_102		Clark and Paula	[121]	2003	17a		https://www.researchgate.net/publication/237535784_Descriptions_of_ten_xanthoidean_Crustacea_Decapoda_Brachyura_first_stage_zoeas_from_Inhaca_Island_Mozambique
Xant_103		Clark and Paula	[121]	2003	21a		https://www.researchgate.net/publication/237535784_Descriptions_of_ten_xanthoidean_Crustacea_Decapoda_Brachyura_first_stage_zoeas_from_Inhaca_Island_Mozambique
Xant_104		Clark and Paula	[121]	2003	25a		https://www.researchgate.net/publication/237535784_Descriptions_of_ten_xanthoidean_Crustacea_Decapoda_Brachyura_first_stage_zoeas_from_Inhaca_Island_Mozambique

no	fossil age	author	Reference number	year	figure	page	link
Xant_105		Clark and Paula	[121]	2003	29a		https://www.researchgate.net/publication/237535784_Descriptions_of_ten_xanthoidean_Crustacea_Decapoda_Brachyura_first_stage_zoeas_from_Inhaca_Island_Mozambique
Xant_106		Clark and Paula	[121]	2003	33a		https://www.researchgate.net/publication/237535784_Descriptions_of_ten_xanthoidean_Crustacea_Decapoda_Brachyura_first_stage_zoeas_from_Inhaca_Island_Mozambique
Xant_107		Clark and Paula	[121]	2003	37a		https://www.researchgate.net/publication/237535784_Descriptions_of_ten_xanthoidean_Crustacea_Decapoda_Brachyura_first_stage_zoeas_from_Inhaca_Island_Mozambique
Xant_108		Clark and Ng	[120]	1998	1b		https://sciencepress.mnhn.fr/sites/default/files/articles/pdf/z1998n2a7_0.pdf

no	fossil age	author	Reference number	year	figure	page	link
Xant_109		Wear	[434]	1968	32		https://www.tandfonline.com/doi/pdf/10.1080/00288330.1968.9515239?needAccess=true
Xant_111		Fransozo et al.	[161]	2001	3a		https://aquila.usm.edu/cgi/viewcontent.cgi?article=1322&context=gcr
Xant_112		Lai et al.	[270]	2011	4b		https://www.sciencedirect.com/science/article/abs/pii/S0044523111000519
Xant_113		Lai et al.	[270]	2011	4c		https://www.sciencedirect.com/science/article/abs/pii/S0044523111000520
Xant_114		Lai et al.	[270]	2011	4a		https://www.sciencedirect.com/science/article/abs/pii/S0044523111000521
Xant_115		Lai et al.	[270]	2011	4d		https://www.sciencedirect.com/science/article/abs/pii/S0044523111000522
Xant_116		Clark and Al-Aidaroos	[118]	1996	1a		https://www.kau.edu.sa/Files/320/Researches/48133_19372.pdff

no	fossil age	author	Reference number	year	figure	page	link
Xant_117		Clark and Ng	[120]	1998	1a		https://sciencepress.mnhn.fr/sites/default/files/articles/pdf/z1998n2a7_0.pdf

Supplementary T

no	CC License	accession number	museum	geographic information	cruise
CaDi_002				Italy	
CaDi_003		MNHN-IU-2008-11871	MNHN Paris	20° 42' 56.8764'' S ; 167° 1' 29.9784'' E	BIOCAL cruise 1985, station CP84
CaDi_004		MNHN-IU-2014-22918	MNHN Paris	20° 45' 42.0012'' S ; 139° 10' 6.006'' O	SMCB cruise 1990, station CAS331
CaDi_005			MNHN Paris	21° 1' 23.9988'' S ; 55° 10' 17.9976'' E	MD32 (REUNION) cruise 1982, station CP177
CaDi_006			MNHN Paris	9° 41' 24'' S ; 139° 3' 48.0204'' O	MUSORSTOM 9 cruise 1997, station CP1238
CaDi_007					
CaDi_008					

no	CC License	accession number	museum	geographic information	cruise
CaDi_009					
CaDi_010					
CaDi_011					
CaDi_012					
CaDi_013					
CaDi_014					
CaDi_015			MNHN Paris	(Hors campagne INVMAR) cruise,	

no	CC License	accession number	museum	geographic information	cruise
CaDi_016					
CaDi_017	4	https://www.inatu...rvations/80735429			
CaDi_018	4	https://www.inatu...rvations/80406923			
CaDi_019	4	http://bins.bolds...cessid=CBCC025-11			

no	CC License	accession number	museum	geographic information	cruise
CaDi_020	4	http://bins.bolds...cessid=CBCC028-11			
CaDi_021	4	http://bins.bolds...cessid=CBCC026-11			
CaDi_022		MNHN-IU-2013-2326	MNHN Paris	02°30'S ; 150°40'E	MADEEP/CP4253
CaDi_023		MNHN-IU-2008-10171	MNHN Paris	25°56'S ; 33°07'E	MAINBAZA/CP3131
CaDi_024		MNHN-IU-2008-10173	MNHN Paris	25° 52' 41.988'' S ; 33° 6' 56.412'' E	MAINBAZA/CP3130
CaDi_025		MNHN-IU-2008-10172	MNHN Paris	23° 31' 40.188'' S ; 35° 45' 45.612'' E	MAINBAZA/CP3143

no	CC License	accession number	museum	geographic information	cruise
CaDi_026		IEO-CD-MZ07/1909	IEO-Centro Oceanográfico de Cádiz	South East African Coast (Mozambique), 51- Indian Ocean, Western	
CaDi_027	4	49011196		Ards, Northern Ireland, United Kingdom	
CaDi_028	4	ICM-CSIC:observadoresdelmar:368		Spain	
CaDi_029		MNHN-IU-2019-600	MNHN Paris	42° 42' 41.508'' N ; 9° 27' 32.22'' E	CORSICABENTHOS 1/CS07

no	CC License	accession number	museum	geographic information	cruise
CaDi_030	4	73508587		Netherlands	
CaDi_031	4	https://www.inatu...rvations/75053798		Pellestrina (Venice), Italy	
CaDi_032	4	https://www.inatu...rvations/88988280		Vila Real de Santo António, Portugal	
CaDi_033	4	https://observati...rvation/177179126		South Netherlands	

no	CC License	accession number	museum	geographic information	cruise
CaDi_034	4	https://www.inatu... rvations/79838924		Greece	
CaDi_035	4	https://www.inatu... rvations/29391685		Greece	
CaDi_036	4	ICM-CSIC:observadoresdelmar:9610	ICM-CSIC	Spain	
CaDi_037					
CaDi_038					
CaDi_039					
CaDi_040					
CaDi_041					
CaDi_042		MNHN-B 27748		New Caledonia	BATHUS 2/CP737
CaDi_043		MNHN-B 27749		Madagascar	Vauban, chalutage 11,
CaDi_044		NHM 1884.31.2		Indonesia	Challenger, stn 192

no	CC License	accession number	museum	geographic information	cruise
CaDi_045		NHM 1884.31.3		Indonesia	Challenger, stn 192
CaDi_046		MNHN-B 27755		9°41,4’S, 139°03,8’W	MUSORSTOM 9/CP1238
CaDi_047		MNHN-B 28443		20°43’S, 167°01’E	BIOCAL/CP84
CaDi_048		ZRC 2001.341		Philippines	
CaDi_049		MNHN-B 28449		1°01,4’S, 55°10,3’E,	MD 32, stn CP 177
CaDi_050		USNM 29674		21°04’05”N, 157°10’35”W	Albatross, stn 3838
CaDi_051		MNHN-B 27754		20°45,7’S, 139°10,1’W	SMSRB, stn 331
CaDi_052		MNHN-B 28446		5°36,63’S, 167°16,34’E	MUSORSTOM 8/CP 1071
CaDi_053		MNHN-B 28440		1°44,174’S, 166°35,354’E	HALIPRO 1/CP 855
CaDi_054					
CaDi_055					
CaDi_056					
CaDi_057					
CaDi_058					
CaDi_059					
CaDi_060					
CaDi_061					
CaDi_062					
CaDi_063					
CaDi_064					
CaDi_065					

no	CC License	accession number	museum	geographic information	cruise
CaDi_066					
CaDi_067					
CaDi_068					
CaDi_069					
CaDi_070					
CaDi_071					
CaDi_072					
CaDi_073					
CaDi_074					
CaDi_075					
CaDi_076					
CaDi_077					
CaDi_078					
CaDi_079					
CaDi_080					
CaDi_081					
CaDi_082					
CaDi_083					
CaDi_084					
CaDi_085					
CaDi_086					
CaDi_087		UF 27553	Florida Museum of Natural History		
CaDi_088		UF 40176	Florida Museum of Natural History	Guam	

no	CC License	accession number	museum	geographic information	cruise
CaDi_089					
CaDi_090		SMF 38490		North Sea	
CaDi_091		GBIF 31947150		Scotland	
CaDi_092				Turkey	
CaDi_093		MOUFPE 15259		Brazil	
CaDi_094				Adriatic	
CaDi_095					
CaDi_096					
CaDi_097		NHMD 82549	NHMD		
CaDi_098					
CaDi_099		MT01254	Deutsches Zentrum fuer Marine Biodiversitaetsforschung		
CaDi_100					
CaDi_101		BPBM 2664			
CaDi_102		MNHN-B 13674			
CaDi_103		SDSNH 81058			
CaDi_104					
CaDi_105		UWBM 108651			
CaDi_106		TKMP 1701 95840			
CaDi_107					

no	CC License	accession number	museum	geographic information	cruise
CaDi_108					
CaDi_109					
CaDi_110					
CaDi_111		USNM 507813			
CaDi_112		KMNH IvP300.020			
CaDi_113					
CaDi_114		EW.9506			
CaDi_115					
CaDi_116					
CaDi_117					
CaDi_118		MNHN R03778			
CaDi_119					
CaDi_120		USNM 50786			
CaDi_121		UWBM_IP_96978_1	UWBM, Seattle		

no	CC License	accession number	museum	geographic information	cruise
CaDi_122		UWBM_IP_96978_2	UWBM, Seattle		
CaDi_123		BOLD:AAC9108	University of Bergen, NaturalHistory Collections		
CaDi_124	C.C. BY-4.0		GBIF		
CaDi_125	C.C. BY-4.0		GBIF		
CaDi_126			University of Bergen, NaturalHistory Collections		
CaDi_127		Crustacea 18213	Goteborg Natural History Museum		
CaDi_128		ZMBN89738	University of Bergen, NaturalHistory Collections		

no	CC License	accession number	museum	geographic information	cruise
CaDi_129		MNHN-IU-2000-1063	MNHN Paris	Mediterranean	
CaDi_130		MNHN-IU-2019-612	MNHN Paris	Mediterranean	CORSICABENTHOS 1, stat. CD06
CaDi_131		MNHN-IU-2014-22933	MNHN Paris	South Atlantic	INVMAR, stat. 108
CaDi_132			ICM-CSIC		
CaDi_133		SWEMA549-15	Goteborg Natural History Museum		
CaDi_134		BNSDE127-12	Senckenberg am Meer		
CaDi_135					
CaDi_136					
CaDi_137					
CaDi_138					
CaDi_139					
CaDi_140					

no	CC License	accession number	museum	geographic information	cruise
CaDi_141					
CaDi_142					
CaDi_143					
CaDi_144					
CaDi_145					
CaDi_146					
CaDi_147					
car_001					
car_002					
car_003					
car_004					
car_005					
car_006					
car_007					
car_008					
car_009					
car_010					
car_011					
car_012					
car_013					
car_014					
car_015					
car_016			AWI, Helgoland		
car_017			AWI, Helgoland		
car_018			AWI, Helgoland		
car_019			AWI, Helgoland		
car_020			AWI, Helgoland		
car_021			AWI, Helgoland		
car_022			AWI, Helgoland		
car_023			AWI, Helgoland		
car_024			AWI, Helgoland		

no	CC License	accession number	museum	geographic information	cruise
car_025			AWI, Helgoland		
car_026			AWI, Helgoland		
car_027			AWI, Helgoland		
car_028			AWI, Helgoland		
car_029			AWI, Helgoland		
car_030			AWI, Helgoland		
car_031			AWI, Helgoland		
car_032			AWI, Helgoland		
car_033			AWI, Helgoland		
car_034			AWI, Helgoland		
car_035			AWI, Helgoland		
car_036			AWI, Helgoland		
car_037			AWI, Helgoland		
car_038			AWI, Helgoland		
car_039			AWI, Helgoland		
car_040			AWI, Helgoland		
car_041			AWI, Helgoland		
car_042			AWI, Helgoland		
car_043			AWI, Helgoland		
car_044			AWI, Helgoland		
car_045			AWI, Helgoland		
car_046			AWI, Helgoland		
car_047					
car_048					

no	CC License	accession number	museum	geographic information	cruise
car_049					
car_050					
car_051					
car_052					
car_053					
car_054					
car_055					
car_056					
car_057					
Dio_001					
Dio_002					
Dio_003					
Dio_004					
Dio_005					
Dio_006					
Dio_007					

no	CC License	accession number	museum	geographic information	cruise
Dio_008					
Dio_009					
Dio_010					
Dio_011					
Dio_012					
Dio_013					
Dio_014					
Dio_015					
Dio_016					
Dio_017					
Dio_018					
Dio_019					
Dio_020					
Dio_021					
Dio_022					
Dio_023					
Dio_024					
Dio_025					
Dio_026					
Dio_027					
Dio_028					
Dio_029					

no	CC License	accession number	museum	geographic information	cruise
Dio_030					
Dio_031					
Dio_032					
Dio_033					
Dio_034					
Dio_035					
Dio_036					
Dio_037					
Dio_038					
Dio_039					
Dio_040					
Dio_041					
Dio_042					
Dio_043					
Dio_044					
Dio_045					
Dio_046					
Dio_047					
Dio_048					
Dio_049					
Dio_050					
Dio_051					
Dio_052					
Dio_053					
Dio_054					
Dio_055					
Dio_056					

no	CC License	accession number	museum	geographic information	cruise
Dio_057					
Dio_058					
Dio_059					
Dio_060					
dro_001		ZRC 2002.630		Phillipines	
dro_002					
dro_003					
dro_004					
dro_005					
dro_006					
dro_007		MNHN B26473	MNHM Paris		MUSORSTOM 9, stn CP 1228
dro_008					
dro_009					
dro_010					
dro_011					
dro_012					
dro_013					
dro_014					
dro_015					
dro_016					
dro_017					
dro_018					
dro_019					
dro_020					
dro_021					
dro_022					

no	CC License	accession number	museum	geographic information	cruise
dro_023					
dro_024	C.C. BY 4.0	MNHN-IU-2014-8099	MNHM Paris	2° 33' 24.5988'' S ; 150° 41' 20.382'' E	KAVIENG 2014, stat. CP4457
dro_025	C.C. BY 4.0	MNHN-IU-2008-12865	MNHM Paris	Madagascar	INVMAR
dro_026	C.C. BY 4.0	MNHN-IU-2014-8631	MNHM Paris	Madagascar	INVMAR
dro_027	C.C. BY 4.0	MNHN-IU-2014-2197	MNHM Paris	2° 34' 0.5412'' S ; 150° 47' 5.0784'' E	KAVIENG 2014, stat. KB14
dro_028	C.C. BY 4.0	MNHN-IU-2013-405	MNHM Paris	5° 12' 27.7776'' S ; 145° 49' 4.062'' E	
dro_029	C.C. BY 4.0	MNHN-IU-2013-523	MNHM Paris	5° 11' 0.6'' S ; 145° 48' 24.0156'' E	Papua Niugini, stat. PB08
dro_030	C.C. BY 4.0	MNHN-IU-2013-948	MNHM Paris	5° 11' 55.3812'' S ; 145° 49' 36.8004'' E	Papua Niugini, stat. PB28
dro_031	C.C. BY 4.0	MNHN-IU-2013-1332	MNHM Paris	5° 11' 15.6588'' S ; 145° 49' 33.3948'' E	Papua Niugini, stat. PB47

no	CC License	accession number	museum	geographic information	cruise
dro_032	C.C. BY 4.0	MNHN-IU-2008-11220	MNHM Paris	19° 18' 3.6036'' S ; 158° 48' 51.5916'' E	CORAIL 2, stat. CP111
dro_033	C.C. BY 4.0	MNHN-IU-2008-11817	MNHM Paris	Ile de Guam	INVMAR
dro_034	C.C. BY 4.0	MNHN-IU-2013-389	MNHM Paris	PACIFIQUE ORIENTAL	Papua Niugini
dro_035	C.C. BY 4.0	MNHN.F.B21561	MNHM Paris	Ile de Sheppey	
dro_036	C.C. BY 4.0	MNHN-IU-2008-11161	MNHM Paris	Cape Vert - Santiago	INVMAR, stat. 137
dro_037	C.C. BY 4.0	MNHN-IU-2008-12775	MNHM Paris		
dro_038	C.C. BY 4.0	MNHN-IU-2008-11159	MNHM Paris	Sénégal	INVMAR

no	CC License	accession number	museum	geographic information	cruise
dro_039	C.C. BY 4.0	MNHN-IU-2008-11162	MNHM Paris	Mauritanie	INVMAR
dro_040	C.C. BY 4.0	MNHN-IU-2008-11166	MNHM Paris	Côte d'Ivoire	INVMAR
dro_041	C.C. BY 4.0	MNHN-IU-2008-11164	MNHM Paris	Congo - Brazzaville	INVMAR
dro_042	C.C. BY 4.0	MNHN-IU-2008-11158	MNHM Paris	Sénégal	INVMAR
dro_043	C.C. BY 4.0	MNHN-IU-2008-11165	MNHM Paris	Congo - Brazzaville	INVMAR
dro_044	C.C. BY 4.0	MNHN-IU-2014-10059	MNHM Paris	Canaries - La Palma - Santa Cruz	INVMAR
dro_045	C.C. BY 4.0	CRU-007385	NHMD Copenhagen	South Africa	

no	CC License	accession number	museum	geographic information	cruise
dro_046	C.C. BY 4.0	MNHN-IU-2008-11170	MNHM Paris	South Africa	
dro_047	C.C. BY 4.0	MNHN-IU-2013-260	MNHM Paris	5° 9' 56.4012'' S ; 145° 50' 25.1952'' E	Papua Niugini, stat. PR08
dro_048	C.C. BY 4.0	MNHN-IU-2013-531	MNHM Paris	5° 12' 2.9988'' S ; 145° 48' 7.812'' E	Papua Niugini, stat. PR14
dro_049	C.C. BY 4.0	MNHN-IU-2013-527	MNHM Paris	5° 12' 27.7776'' S ; 145° 49' 4.062'' E	Papua Niugini, stat. PR16
dro_050	C.C. BY 4.0	MNHN-IU-2013-1278	MNHM Paris	5° 9' 35.3988'' S ; 145° 50' 8.9916'' E	Papua Niugini, stat. PR187
dro_051	C.C. BY 4.0	MNHN-IU-2008-11059	MNHM Paris		
dro_052	C.C. BY 3.0	P.93027	AM Sydney	Australia	
dro_053	C.C. BY 4.0	MNHN-IU-2008-12927	MNHM Paris	Madagascar	INVMAR

no	CC License	accession number	museum	geographic information	cruise
dro_054	C.C. BY 4.0	MNHN-IU-2008-11223	MNHM Paris	22° 39' 0" S ; 167° 7' 24.0132" E	MUSORSTOM 4, stat. DW207
dro_055	C.C. BY 4.0	MNHN-IU-2019-5357	MNHM Paris	20° 33' 48.348" S ; 164° 14' 23.928" E	KOUMAC 2.3, stat. KD506
dro_056	C.C. BY 4.0	MNHN-IU-2008-11218	MNHM Paris	22° 3' 11.9988" S ; 166° 54' 12.0096" E	LAGON, stat. DW619
dro_057	C.C. BY 4.0	MNHN-IU-2008-11482	MNHM Paris	22° 48' 48.0024" S ; 166° 58' 54.0264" E	LAGON, stat. DW569
dro_058	C.C. BY 4.0	MNHN-IU-2008-11480	MNHM Paris	22° 24' 18.0036" S ; 166° 47' 41.9928" E	LAGON, stat. DW111
dro_059	C.C. BY 4.0	MNHN-IU-2008-11481	MNHM Paris	21° 52' 53.9976" S ; 165° 49' 53.994" E	LAGON, stat. DW215
dro_060	C.C. BY 4.0	MNHN-IU-2008-11478	MNHM Paris	22° 37' 59.9988" S ; 166° 49' 5.9844" E	LAGON, stat. DW303

no	CC License	accession number	museum	geographic information	cruise
dro_061	C.C. BY 4.0	MNHN-IU-2013-2616	MNHM Paris	5° 45' 29.9988'' N ; 52° 31' 24.006'' O	GUYANE 2014, stat. CP4385
dro_062	C.C. BY 4.0	46248-Arthropoda	FM Florida	Panama	
dro_063	C.C. BY 3.0	P.93021	AM Sydney	New South Wales	
dro_064	C.C. BY 4.0	J20986	NMV Victoria	Victoria	
dro_065	C.C. BY 4.0	MNHN-IU-2008-12942	MNHM Paris	5° 24' 0'' S ; 57° 1' 23.9916'' E	REVES 2, stat. CH1

no	CC License	accession number	museum	geographic information	cruise
dro_066	C.C. BY 4.0	MNHN-IU-2013-16661	MNHM Paris	19° 12' 0.0036'' S ; 158° 56' 48.0156'' E	CORAIL 2, stat. DW84
dro_067	C.C. BY 4.0	MNHN-IU-2008-12926	MNHM Paris	Senegal	INVMAR
dro_068	C.C. BY 4.0	MNHN-IU-2013-406	MNHM Paris	5° 12' 27.7776'' S ; 145° 49' 4.062'' E	Papua Niugini, stat. PB11
dro_069	C.C. BY 4.0	MNHN-IU-2013-407	MNHM Paris	Papua Niugini	
dro_070	C.C. BY 4.0	MNHN-IU-2013-16662	MNHM Paris		
dro_071	C.C. BY 4.0	MNHN-IU-2013-16662	MNHM Paris		
dro_072	C.C. BY 4.0	MNHN-IU-2013-16662	MNHM Paris		

no	CC License	accession number	museum	geographic information	cruise
dro_073	C.C. BY 4.0	MNHN-IU-2013-16662	MNHM Paris		
dro_074	C.C. BY 4.0	MNHN-IU-2013-16662	MNHM Paris		
dro_075	C.C. BY 4.0	MNHN-IU-2013-16662	MNHM Paris		
dro_076	C.C. BY 4.0	MNHN-IU-2013-16662	MNHM Paris		
dro_079		BMNH 59089			
dro_080		BMNH 59091			
dro_081		M91-127			
dro_082		SDSM 10184			
dro_083		PI 15222			
dro_084		PI 15206			
dro_085		PI 18695			
dro_086		MPZ2011.185			
dro_087					
dro_088		USNM 484531			

no	CC License	accession number	museum	geographic information	cruise
dro_089					
dro_090					
dro_091					
dro_092					
dro_093					
dro_094					
dro_095					
dro_096					
dro_097					
dro_098					
dro_099					
dro_100					
dro_101					
dro_102					
dro_103					
dro_104					
dro_105					
dro_106					
dro_107					
dro_108					
dro_109					
dro_110					
dro_111					

no	CC License	accession number	museum	geographic information	cruise
dro_112	C.C. BY 4.0	MNHN-IU-2008-11217	MNHM Paris	21° 23' 37.2012'' S ; 167° 59' 12.5952'' E	MUSORSTOM 6, stat. DW485
dro_113	C.C. BY 4.0	MNHN-IU-2008-11216	MNHM Paris	23° 2' 12.0012'' S ; 168° 15' 48.0096'' E	SMIB 5, stat. DW98
dro_114	C.C. BY 4.0	44480-Arthropoda	FM Florida	Panama	
dro_115	C.C. BY 4.0	MNHN-IU-2008-11342	MNHM Paris	Seychelles	CEPROS, stat. 13-67
dro_116	C.C. BY 4.0	MNHN-IU-2008-11180	MNHM Paris		
dro_117	C.C. BY 4.0	MNHN-IU-2008-12942	MNHM Paris	5° 24' 0'' S ; 57° 1' 23.9916'' E	REVES 2, Stat. CH1
dro_118	C.C. BY 4.0	MNHN-IU-2013-407	MNHM Paris		Papua Niugini

no	CC License	accession number	museum	geographic information	cruise
dro_119	C.C. BY 4.0	MNHN-IU-2013-406	MNHM Paris	5° 12' 27.7776'' S ; 145° 49' 4.062'' E	Papua Niugini, sat. PB11
dro_120	C.C. BY 4.0	MNHN-IU-2008-12926	MNHM Paris	Senegal	INVMAR, stat. 11
dro_121	C.C. BY 4.0	MNHN-IU-2008-11222	MNHM Paris	19° 33' 18'' S ; 158° 30' 18.018'' E	CHALCAL1, stat. DC31
dro_122	C.C. BY 4.0	MNHN-IU-2008-11179	MNHM Paris	16° 37' 17.9976'' S ; 144° 13' 17.9904'' O	SMCB, stat. CAS253
gal_001		MNHN-IU-2014-5463	MNHN Paris	Hors campagne INVMAR, stat. 15A	
gal_002		MNHN-IU-2014-5473A	MNHN Paris		
gal_003		MNHN-IU-2014-5473B	MNHN Paris		
gal_004		MNHN-IU-2014-5513A	MNHN Paris	Hors campagne INVMAR, stat. 309	
gal_005		MNHN-IU-2014-5513B	MNHN Paris	Hors campagne INVMAR, stat. 309	
gal_006		MNHN-IU-2014-5515	MNHN Paris	Hors campagne INVMAR, stat. 311	

no	CC License	accession number	museum	geographic information	cruise
gal_007		MNHN-IU-2014-5516A	MNHN Paris	Hors campagne INVMAR, stat. 309	
gal_009		MNHN-IU-2014-5520	MNHN Paris		
gal_010					
gal_011					
gal_012					
gal_013					
gal_014					
gal_015					
gal_016					
gal_017					
gal_018					
gal_019					
gal_020					
gal_021					
gal_022					
gal_023					
gal_024					
gal_025					
gal_026					
gal_027					
gal_028					
gal_029					

no	CC License	accession number	museum	geographic information	cruise
gal_030					
gal_031					
gal_032					
gal_033					
gal_034					
gal_035					
gal_036					
gal_037					
gal_038					
gal_039					
gal_040					
gal_041					
gal_042					
gal_043					
gal_044					
gal_045					
gal_046					
gal_048					
gal_049					
gal_050					
gal_051					
gal_052					
gal_053					
gal_054					

no	CC License	accession number	museum	geographic information	cruise
gal_055					
gal_056					
gal_057					
gal_058					
gal_059					
gal_060					
gal_061					
gal_062					
gal_063		TAN0308/67 (NIWA 28076)			
gal_064		TAN0308/67 (NIWA 28066)			
gal_065		TAN0308/126 (NIWA 28065)			
gal_066					
gal_067					
gal_068					
gal_069					
gal_070					
gal_071	CC 4.0	58677-Arthropoda	FM Florida		ARA01
gal_072	CC 4.0	58779-Arthropoda	FM Florida		ARA02

no	CC License	accession number	museum	geographic information	cruise
gal_073	CC 4.0	58434-Arthropoda	FM Florida		
gal_074	CC 4.0	58470-Arthropoda	FM Florida		
gal_075	CC 4.0	58840-Arthropoda	FM Florida		ARA02
gal_076	CC 4.0	58552-Arthropoda	FM Florida		ARA08
gal_077	CC 4.0	58953-Arthropoda	FM Florida		
gal_078	CC 4.0	58873-Arthropoda	FM Florida		ARA05
gal_079	CC 4.0	MNHN-IU-2019-719	MNHN Paris	42° 55' 5.988'' N ; 9° 31' 28.812'' E	CORSICABENTHOS 1, stat. CD03

no	CC License	accession number	museum	geographic information	cruise
gal_080	CC 4.0	MNHN-IU-2019-720	MNHN Paris	42° 59' 49.308'' N ; 9° 27' 12.132'' E	CORSICABENTHOS 1, stat. CS04
gal_081	CC 4.0	MNHN-IU-2018-344	MNHN Paris	20° 33' 15.012'' S ; 164° 12' 59.004'' E	KOUMAC 2.1, stat. KR200
gal_082	CC 4.0	52957-Arthropoda	FM Florida		
gal_083	CC 4.0	52969-Arthropoda	FM Florida		
gal_084	CC 4.0	52969-Arthropoda	FM Florida		
gal_085	CC 4.0	52956-Arthropoda	FM Florida		
gal_086	CC 4.0	58929-Arthropoda	FM Florida		

no	CC License	accession number	museum	geographic information	cruise
gal_087	CC 4.0	58432-Arthropoda	FM Florida		
gal_088	CC 4.0	MNHN-IU-2010-1651	MNHN Paris		
gal_089	CC 4.0	MNHN-IU-2010-1650	MNHN Paris	21° 8' 18.0024'' S ; 167° 54' 30.024'' E	MUSORSTROM 6, stat. DW471
gal_090	CC 4.0	MNHN-IU-2010-1652	MNHN Paris	21° 1' 15.0024'' S ; 167° 31' 36.0192'' E	MUSORSTROM 6, stat. CP464
gal_091	CC 4.0	MNHN-IU-2014-5097	MNHN Paris	20° 36' 0'' S ; 167° 12' 59.976'' E	CALSUB, stat. PL03
gal_092	CC 4.0	44677-Arthropoda	FM Florida		
gal_093	CC 4.0	43110-Arthropoda	FM Florida		
gal_094	CC 4.0	38035-Arthropoda	FM Florida		

no	CC License	accession number	museum	geographic information	cruise
gal_095	CC 4.0	MNHN-IU-2013-211	MNHN Paris	5° 9' 56.4012'' S ; 145° 50' 25.1952'' E	PAPUA NIUGINI, stat. PR08
gal_096	CC 4.0	MNHN-IU-2013-9974	MNHN Paris	25° 1' 16.1976'' S ; 47° 0' 28.7964'' E	ATMO VATAE, stat. TB02-TB03
gal_097	CC 4.0	MNHN-IU-2013-16033	MNHN Paris	24° 59' 46.8024'' S ; 47° 5' 44.394'' E	ATMO VATAE, stat. TB01
gal_098	CC 4.0	MNHN-IU-2013-9968	MNHN Paris	25° 1' 31.1376'' S ; 46° 59' 58.326'' E	ATMO VATAE, stat. TB12
gal_099					
gal_100	CC 4.0	41568-Arthropoda	FM Florida		
gal_101	CC 4.0	43029-Arthropoda	FM Florida		
gal_102	CC 4.0	42374-Arthropoda	FM Florida		

no	CC License	accession number	museum	geographic information	cruise
gal_103	CC 4.0	43301-Arthropoda	FM Florida		
gal_104	CC 4.0	40269-Arthropoda	FM Florida		
gal_105	CC 4.0	48571-Arthropoda	FM Florida		
gal_106	CC 4.0	36151-Arthropoda	FM Florida		
gal_107	CC 4.0	38193-Arthropoda	FM Florida		
gal_108	CC 4.0	37156-Arthropoda	FM Florida		
gal_109	CC 4.0	MNHN-IU-2013-17419	MNHN Paris		

no	CC License	accession number	museum	geographic information	cruise
gal_110	CC 4.0	MNHN-IU-2013-17411	MNHN Paris		
gal_111	CC 4.0	MNHN-IU-2013-17396	MNHN Paris		
gal_112	CC 4.0	MNHN-IU-2013-17398	MNHN Paris		
gal_113	CC 4.0	MNHN-IU-2013-17412	MNHN Paris		
gal_114	CC 4.0	MNHN-IU-2013-503	MNHN Paris		
gal_115	CC 4.0	H1443_225538_Janetogalathea_sp_inc_californiensis_.png			
gal_116	CC 4.0	58483-Arthropoda	FM Florida		

no	CC License	accession number	museum	geographic information	cruise
gal_117	CC 4.0	58473-Arthropoda	FM Florida		
gal_118	CC 4.0	58432-Arthropoda	FM Florida		
gal_119	CC 4.0	42977-Arthropoda	FM Florida		
gal_120	CC 4.0	43010-Arthropoda	FM Florida		
gal_121	CC 4.0	43010-Arthropoda	FM Florida		
gal_122	CC 4.0	38327-Arthropoda	FM Florida		
gal_123	CC 4.0	36433-Arthropoda	FM Florida		

no	CC License	accession number	museum	geographic information	cruise
gal_124	CC 4.0	MNHN-IU-2013-258	MNHN Paris	5° 9' 56.4012'' S ; 145° 50' 25.1952'' E	PAPUA NIUGINI, stat. PR08
gal_125	CC 4.0	MNHN-IU-2010-5252	MNHN Paris	25° 1' 16.1976'' S ; 47° 0' 28.7964'' E	ATIMO VATAE, stat. TB02-TB03
gal_126	CC 4.0	MNHN-IU-2010-5256	MNHN Paris	25° 36' 59.4036'' S ; 46° 18' 1.206'' E	ATIMO VATAE, stat. DW3563
gal_127	CC 4.0	MNHN-IU-2010-5257	MNHN Paris	25° 54' 29.9988'' S ; 44° 51' 0.5976'' E	ATIMO VATAE, stat. DW3605
gal_128	CC 4.0	MNHN-IU-2010-5256	MNHN Paris	25° 36' 59.4036'' S ; 46° 18' 1.206'' E	ATIMO VATAE, stat. DW3563
gal_129	CC 4.0	MNHN-IU-2010-5257	MNHN Paris	25° 54' 29.9988'' S ; 44° 51' 0.5976'' E	ATIMO VATAE, stat. DW3605
gal_130	CC 4.0	MNHN-IU-2010-5254	MNHN Paris	25° 38' 4.2036'' S ; 45° 56' 58.2036'' E	ATIMO VATAE, stat. DW3624
gal_131	CC 4.0	MNHN-IU-2010-5253	MNHN Paris	25° 38' 4.2036'' S ; 45° 56' 58.2036'' E	ATIMO VATAE, stat. DW3624
gal_132	CC 4.0	MNHN-IU-2010-5255	MNHN Paris	25° 54' 29.4012'' S ; 45° 33' 11.9988'' E	ATIMO VATAE, stat. CP3579

no	CC License	accession number	museum	geographic information	cruise
gal_133	CC 4.0	MNHN-IU-2010-1116	MNHN Paris	13° 24' 55.8'' S ; 47° 56' 58.812'' E	MRIKY, stat. DW3230
gal_134	CC 4.0	MNHN-IU-2010-1018	MNHN Paris	14° 52' 3'' S ; 46° 58' 4.8'' E	MRIKY, stat. DW3246
gal_135	CC 4.0	MNHN-IU-2014-11139	MNHN Paris	25° 11' 43.7964'' S ; 47° 12' 31.2048'' E	ATIMO VATAE, stat. CP3572
gal_136	CC 4.0	MNHN-IU-2009-1351	MNHN Paris	12° 40' 56.91'' S ; 44° 57' 51.6312'' E	KUW MAYOTTE 2009, stat. 23
gal_137	CC 4.0	40205-Arthropoda	FM Florida	Saudi Arabia	
gal_138	CC 4.0	39269-Arthropoda	FM Florida	Kiribati	
gal_139	CC 4.0	15270-Arthropoda	FM Florida	Oahu Island	
gal_140	CC 4.0	MNHN-IU-2010-5264	MNHN Paris	23° 0' 31.2012'' S ; 168° 19' 48.0072'' E	NORFOLK 2, stat. CP2141

no	CC License	accession number	museum	geographic information	cruise
gal_141	CC 4.0	MNHN-IU-2010-5264	MNHN Paris	23° 0' 31.2012'' S ; 168° 19' 48.0072'' E	NORFOLK 2, stat. CP2141
gal_142	CC 4.0	MNHN-IU-2010-5262	MNHN Paris	10° 17' 0.0024'' S ; 161° 42' 59.976'' E	SALOMON 1, stat. DW1840
gal_143	CC 4.0	MNHN-IU-2010-5263	MNHN Paris	23° 22' 23.9988'' S ; 168° 5' 12.012'' E	SMIB 5, stat. DW100
gal_144					
gal_145					
gal_146					
gal_147					
gal_148					
gal_149					
gal_150					

no	CC License	accession number	museum	geographic information	cruise
gal_151					
gal_152					
gal_153					
gal_154					
gal_155				Australia	
gal_156				Vanuatu	
gal_157				Australia	
gal_158				Red Sea	
gal_159				French Ploynesia	
gal_160				Red Sea	
gal_161				Solomon Islands	
gal_162				Fiji	
gal_163				Australia	
gal_164				French Ploynesia	
gal_165				Australia	
gal_166				French Ploynesia	
gal_167				New Caledonia	
gal_168				New Caledonia	

no	CC License	accession number	museum	geographic information	cruise
gal_169				Madagascar	
gal_170				French Ploynesia	
gal_171				Red Sea	
gal_172				Vanuatu	
gal_173				Red Sea	
gal_174				New Caledonia	
gal_175				New Caledonia	
gal_176				Vanuatu	
gal_177				South China Sea	
gal_178				Wallis and Futuna	
gal_179				Indonesia	
gal_180				Vanuatu	
gal_181				New Caledonia	
gal_182				Vanuatu	
gal_183				New Caledonia	
gal_184				Fiji	
gal_185				Mozambique	
gal_186				Australia	

no	CC License	accession number	museum	geographic information	cruise
gal_187				Papua New Gunea	
gal_188				Scattered Islands	
gal_189				Australia	
gal_190				Indonesia	
gal_191				New Caledonia	
gal_192				Vanuatu	
gal_193				Vanuatu	
gal_194				Indonesia	
gal_195				Indonesia	
gal_196				Vanuatu	
gal_197				Solomon Islands	
gal_198				Vanuatu	
gal_199				Mozambique	
gal_200				Australia	
gal_201				Australia	
gal_202				Vanuatu	
gal_203				Solomon Islands	
gal_204				South Africa	

no	CC License	accession number	museum	geographic information	cruise
gal_205				Fiji	
gal_206				Red Sea	
gal_207				New Caledonia	
gal_208				Philippines	
gal_209				Vanuatu	
gal_210				Fiji	
gal_211				Red Sea	
gal_212				Thailand	
gal_213				Vanuatu	
gal_214				Wallis and Futuna	
gal_215				New Caledonia	
gal_216				Australia	
Gra_001					
Gra_002					
Gra_003					
Gra_004					
Gra_005					
Gra_006					
Gra_007					
Gra_008					
Gra_009					

no	CC License	accession number	museum	geographic information	cruise
Gra_010					
Gra_011					
Gra_012					
Gra_016					
Gra_017					
Gra_018					
Gra_019					
Gra_020					
Gra_021					
Gra_022					
Gra_023					
Gra_024					
Gra_026					
Gra_038					
Gra_039					
Gra_040					
Gra_041					
Gra_042					
Gra_043					
Gra_044					
Gra_045					
Gra_046					
Gra_047					
Gra_048					
Gra_049					
Gra_050					
Gra_051					
Gra_052					
Gra_053					
Gra_054					

no	CC License	accession number	museum	geographic information	cruise
Gra_055					
Gra_056					
Gra_057					
Gra_058					
Gra_059					
Gra_060					
Gra_061					
Gra_062					
Gra_063					
Gra_064					
Gra_065					
Gra_066					
Gra_067					
Gra_068					
Gra_069					
Gra_070					
Gra_072					
Gra_073					
Gra_074					
Gra_075					
Gra_076					
Gra_077					
Gra_078					
Gra_079					
Gra_080					
Gra_081					
Gra_082					
Gra_083					
Gra_090					
Gra_092					
Gra_093					

no	CC License	accession number	museum	geographic information	cruise
Gra_094					
Gra_100					
Gra_101					
Gra_102					
Gra_103					
Gra_104					
Gra_105					
Gra_106					
Gra_107					
Gra_108					
Gra_109					
Gra_110					
Gra_111					
Gra_112					
Gra_113					
Gra_114					
Gra_115					
Gra_116					
Gra_117					
Gra_122					
Gra_123					
Gra_124					
Gra_125					
Gra_126					
Gra_127					
Gra_128					
Gra_129					

no	CC License	accession number	museum	geographic information	cruise
Gra_130					
Gra_131					
Gra_132					
Gra_133					
Gra_134					
Gra_135					
Gra_136					
Gra_137					
Gra_138					
Gra_139					
Gra_140					
Gra_141					
Gra_144					
Gra_145					
Gra_146					
Gra_147					
Gra_148					
Gra_149					
Gra_151					
Gra_152					
Gra_154					
Gra_155					
Gra_156					
Gra_157					
Gra_158					
Gra_159					
Gra_162					

no	CC License	accession number	museum	geographic information	cruise
Gra_163					
Gra_170					
Gra_171					
Gra_172					
Gra_173					
Gra_175					
Gra_176					
Gra_177					
Gra_178					
Gra_179					
Gra_180					
Gra_181					
Gra_182					
Gra_183					
Gra_184					
Gra_185					
Gra_186					
Gra_187					
Gra_188					
Gra_189					
Gra_190					
Gra_191					
Gra_192					
Gra_193					

no	CC License	accession number	museum	geographic information	cruise
Gra_194					
Gra_195					
Gra_196					
Gra_197					
Gra_198					
Gra_199					
Gra_207					
Gra_208					
Gra_209					
Gra_210					
Gra_211					
Gra_213					

no	CC License	accession number	museum	geographic information	cruise
Gra_214					
Gra_215					
Gra_216					
Gra_217					
Gra_218					
Hi_001					
Hi_002					
Hi_003					
Hi_004					
Hi_005					
Hi_006					
Hi_007					
Hi_008					
Hi_009					
Hi_010					
Hi_011					
Hi_012					
Hi_013					

no	CC License	accession number	museum	geographic information	cruise
Hi_014					
Hi_015					
Hi_016					
Hi_017					
Hi_018					
Hi_019					
Hi_020					
Hi_021					
Hi_022					
Hi_023					
Hi_024					
Hi_025					
Hi_026					
Hi_027					
Hi_028					
Hi_029					
Hi_030					
Hi_031					
Hi_032					
Hi_033					
Hi_034					
Hi_035					

no	CC License	accession number	museum	geographic information	cruise
Hi_036		MNHN-IU-2014-5475A	MNHN Paris	3°38'S 9°22'E, west of Gabun	Ombango 1960, c. 12, station 301
Hi_037		MNHN-IU-2014-5475B	MNHN Paris	3°38'S 9°22'E, west of Gabun	Ombango 1960, c. 12, station 301
Hi_038		MNHN-IU-2014-5524A	MNHN Paris	23°07'S - 43°11'W, south of Brazil	Calypso 1961-62, station 108
Hi_039		MNHN-IU-2014-5524B	MNHN Paris	23°07'S - 43°11'W, south of Brazil	Calypso 1961-62, station 108
Hi_040		MNHN-IU-2014-5526	MNHN Paris	24°03'S - 46°22'W, south of Brazil	Calypso 1961-62, station 139
Hi_041		ZMH-K07448A	CeNak Hamburg	20°S 73°W, west of Chile	–
Hi_042		ZMH-K07448B	CeNak Hamburg	20°S 73°W, west of Chile	–
Hi_043		ZMH-K16356	CeNak Hamburg	Sansibar	–
Hi_044		MNHN-IU-2014-5468	MNHN Paris		
Hi_045		SMF-Mu_267	CeNak Hamburg		
Hi_046		ZMUC-CRU-8679	NHMD Copenhagen		
Hi_047		ZMUC-CRU-8680	NHMD Copenhagen		
Hi_048		ZMUC-CRU-8682	NHMD Copenhagen		
Hi_049		ZMUC-CRU-8683	NHMD Copenhagen		
Hi_050		ZMUC-CRU-8684	NHMD Copenhagen		
Hi_051					
Hi_052					
Hi_053					

no	CC License	accession number	museum	geographic information	cruise
Hi_054					
Hi_055					
Hi_056					
Hi_057					
Hi_058					
Hi_059					
Hi_060					
Hi_061					
Hi_062					
Hi_063					
Hi_064					
Hi_065					
Hi_066					
Hi_067					
Hi_068					
Hi_069					
Hi_070					
Hi_071					
Hi_072		MNHN-IU-2014-5518	MNHN Paris	–	Calypso 1961-62, station 153
Hi_073		MNHN-IU-2014-5523	MNHN Paris	08°25'S - 34°48'W, east of Brazil	Calypso 1961-62, station 26

no	CC License	accession number	museum	geographic information	cruise
Hi_074		MNHN-IU-2014-5527	MNHN Paris	24°03'S - 46°22'W, south of Brazil	Calypso 1961-62, station 139
Hi_075					
Hi_076					
Hi_077					
Hi_078					
Hi_079					
Hi_080					
Hi_081					
Hi_082					
Hi_083					
Hi_084					
Hom_101					
Hom_118					
Hom_119					
Hom_125					
Hom_126					
Hom_143					
Hom_160					
Hom_161					
Hom_162					
Hom_163					
Hom_165					
Hom_193					
Hom_194					
Hom_300					

no	CC License	accession number	museum	geographic information	cruise
Hom_301					
Hom_302					
Hom_304					
Hom_305					
Hom_306					
Hom_307					
Hom_308					
Hom_309					
Hom_310					
Hom_311					
Hom_312					
Hom_313					
Hom_314					
Hom_315					
Hom_324					
Hom_325					
Hom_326					
Hom_40					
Hom_44					
Hom_54					
Hom_78					
Hom_87					
jur_002				Calvados, Franc	
jur_003				Calvados, Franc	
jur_004				Calvados, Franc	
jur_005				Calvados, Franc	
jur_006				unknown	

no	CC License	accession number	museum	geographic information	cruise
jur_007				Kawodrza Górna, near Cz˛estochowa, central PolandKawodrza Górna, near Cz˛estochowa, central PolandKawodrza Górna, near Cz˛estochowa, central Poland	
jur_008				southwest of Germany	
jur_009				West Pomerania, Germany	
jur_010				Geising an der Donau, Germany	
jur_011				Storzingen near Sigmarien, Germany	
jur_012				Lithuania	
jur_013				Göllersreuth near Thalmässing, Bavaria, S Germany	
jur_014				Fürsitz/Braunenberg near Aalen-Wasseralfingen, SW Germany	
jur_015				Engen, SW Germany	

no	CC License	accession number	museum	geographic information	cruise
jur_016				Biburg, Bavaria, lower Kimmeridgian	
jur_017				Treuchtlingen, Bavaria	
jur_018				Braunenberg near Aalen-Wasseralfingen, SW Germany	
jur_019				Titting, Bavaria, Germany	
jur_020				Titting, Bavaria, Germany	
jur_021				Oerlinger Tal near Ulm, Germany	
jur_022				Austria, Czech Republic	
jur_023				Czech Republic	
jur_024				Hungary	
jur_025				France	
jur_026				Romania	

no	CC License	accession number	museum	geographic information	cruise
jur_027				Germany	
jur_028				Swabia, Germany	
jur_029				Hungary	
jur_030				France	
jur_031				Swabia, Germany	
jur_032					
jur_033					
jur_034					
jur_035					

no	CC License	accession number	museum	geographic information	cruise
jur_036					
jur_037					
jur_038					
jur_039					
Lit_001	Timo				
Lit_002					
Lit_003					
Lit_004					
Lit_005					
Lit_006					
Lit_007					
Lit_008					
Lit_009					
Lit_010					
Lit_011					
Lit_012					
Lit_013					
Lit_014					
Lit_015					
Lit_016					
Lit_017					
Lit_018					
Lit_019					
Lit_020					
Lit_021					
Lit_022					
Lit_023					
Lit_024					
Lit_025					

no	CC License	accession number	museum	geographic information	cruise
Lit_026					
Lit_027					
Lit_028					
Lit_029					
Lit_030					
Lit_031					
Lit_032					
Lit_033					
Lit_034					
Lit_035					
Pag_0001					
Pag_0003					
Pag_0005					
Pag_0007					
Pag_0009					
Pag_0011					
Pag_0014					

no	CC License	accession number	museum	geographic information	cruise
Pag_0018	CC BY-NC 4.0	48501-Arthropoda	Florida Museum of Natural History (UF)		
Pag_0019	CC BY-NC 4.0	39079-Arthropoda	Florida Museum of Natural History (UF)		
Pag_0020					
Pag_0021					
Pag_0022					
Pag_0023					
Pag_0024					
Pag_0030	CC BY-NC 2.0				
Pag_0031					
Pag_0032					
Pag_0033					
Pag_0034					
Pag_0040					
Pag_0042					
Pag_0044					
Pag_0046					
Pag_0048					
Pag_0050					

no	CC License	accession number	museum	geographic information	cruise
Pag_0052					
Pag_0054					
Pag_0056					
Pag_0058					
Pag_0060					
Pag_0062					
Pag_0065					
Pag_0067					
Pag_0069					
Pag_0072					
Pag_0075					
Pag_0089	CC BY-NC 4.0	52417-Arthropoda	Florida Museum of Natural History (UF)		
Pag_0090	CC BY-NC 4.0	52641-Arthropoda	Florida Museum of Natural History (UF)		
Pag_0091					
Pag_0093					
Pag_0095					
Pag_0097					
Pag_0099					
Pag_0101					
Pag_0102					

no	CC License	accession number	museum	geographic information	cruise
Pag_0103					
Pag_0111					
Pag_0113					
Pag_0115					
Pag_0117					
Pag_0119					
Pag_0122					
Pag_0127					
Pag_0129					
Pag_0134					
Pag_0136					
Pag_0141					
Pag_0143					
Pag_0148					
Pag_0150					
Pag_0152					
Pag_0157					
Pag_0159					
Pag_0164					
Pag_0166					
Pag_0171					
Pag_0173					
Pag_0174					
Pag_0181					
Pag_0182					
Pag_0183					
Pag_0184					
Pag_0189					
Pag_0193					

no	CC License	accession number	museum	geographic information	cruise
Pag_0195					
Pag_0197					
Pag_0199					
Pag_0202					
Pag_0203					
Pag_0204					
Pag_0205					
Pag_0219	CC BY-NC 4.0	47672-Arthropoda	Florida Museum of Natural History (UF)		
Pag_0220	CC BY-NC 4.0	NHMD85723	Natural History Museum Denmark (NHMD)		
Pag_0221	CC BY-NC 4.0	MNHN-IU-2013-5330	Muséum National d'Histoire Naturelle (MNHN), Paris		
Pag_0222	CC BY-NC 4.0	MNHN-IU-2013-6763	Muséum National d'Histoire Naturelle (MNHN), Paris		

no	CC License	accession number	museum	geographic information	cruise
Pag_0223	CC BY-NC 4.0		GBIF.org		
Pag_0228					
Pag_0230					
Pag_0232					
Pag_0234					
Pag_0236					
Pag_0239					
Pag_0241					
Pag_0244					
Pag_0246					
Pag_0247					
Pag_0248					
Pag_0249					
Pag_0254					
Pag_0256					

no	CC License	accession number	museum	geographic information	cruise
Pag_0257					
Pag_0259					
Pag_0264					
Pag_0267					
Pag_0268					
Pag_0269					
Pag_0274					
Pag_0276					
Pag_0280					
Pag_0281					
Pag_0282					
Pag_0283					

no	CC License	accession number	museum	geographic information	cruise
Pag_0290					
Pag_0291					
Pag_0292					
Pag_0293					
Pag_0294					
Pag_0295					
Pag_0296					
Pag_0306					
Pag_0308					

no	CC License	accession number	museum	geographic information	cruise
Pag_0310					
Pag_0312					
Pag_0314					
Pag_0316					
Pag_0318					
Pag_0320					
Pag_0322					
Pag_0324					
Pag_0332					
Pag_0333					
Pag_0334					
Pag_0335					

no	CC License	accession number	museum	geographic information	cruise
Pag_0336					
Pag_0337					
Pag_0339					
Pag_0342					
Pag_0348					
Pag_0352					
Pag_0353					
Pag_0354					
Pag_0355					
Pag_0356					
Pag_0388					
Pag_0392					
Pag_0394					
Pag_0396					
Pag_0398					
Pag_0402					
Pag_0405					
Pag_0409					
Pag_0412					

no	CC License	accession number	museum	geographic information	cruise
Pag_0416					
Pag_0418					
Pag_0420					
Pag_0424					
Pag_0426					
Pag_0428					
Pag_0432					
Pag_0434					
Pag_0438					
Pag_0440					
Pag_0441					
Pag_0443					
Pag_0445					
Pag_0446					
Pag_0448					
Pag_0450					
Pag_0452					
Pag_0454					
Pag_0456					
Pag_0460					
Pag_0464					
Pag_0466					
Pag_0468					
Pag_0470					
Pag_0474					
Pag_0476					
Pag_0478					
Pag_0480					
Pag_0482					

no	CC License	accession number	museum	geographic information	cruise
Pag_0488					
Pag_0492					
Pag_0495					
Pag_0499					
Pag_0500					
Pag_0501					
Pag_0504					
Pag_0510					
Pag_0513					
Pag_0517					
Pag_0533					
Pag_0537					
Pag_0541					
Pag_0550					
Pag_0554					
Pag_0558					
Pag_0561					
Pag_0565					
Pag_0568					
Pag_0571					
Pag_0576					
Pag_0581					
Pag_0585					

no	CC License	accession number	museum	geographic information	cruise
Pag_0589					
Pag_0592					
Pag_0596					
Pag_0600					
Pag_0604					
Pag_0609					
Pag_0611					
Pag_0613					
Pag_0614					
Pag_0617					
Pag_0620					
Pag_0623					
Pag_0626					
Pag_0629					
Pag_0632					
Pag_0635					
Pag_0638					
Pag_0641					
Pag_0645					
Pag_0648					

no	CC License	accession number	museum	geographic information	cruise
Pag_0651					
Pag_0655					
Pag_0660					
Pag_0664					
Pag_0668					
Pag_0700					
Pag_0711					
Pag_0716					
Pce_001			Caribbean coast.		University of Miami - Rosenstiel School of Marine and Atmospheric Science
Pce_002			Pacific mouth of the Panama Canal.		University of Miami - Rosenstiel School of Marine and Atmospheric Science
Pce_003			tropical American coasts and west coast of Africa		University of Miami

no	CC License	accession number	museum	geographic information	cruise
Pce_004			New Zealand, Tasmania and Australia		University of Queensland, Brisbane, Australia
Pce_005			New Zealand, Tasmania and Australia		University of Queensland, Brisbane, Australia
Pce_006			Panamanian Isthmus, Bahama Islands		University of Miami
Pce_007			Chilean Coast		Universidad de Chile
Pce_008			Chilean Coast		Universidad de Chile
Pce_009			Japan		The Curstacean Society
Pce_010			Japan		The Curstacean Society

no	CC License	accession number	museum	geographic information	cruise
Pce_011			Japan		The Curstacean Society
Pce_012			Pacific Specimen		University of Miami - Rosenstiel School of Marine and Atmospheric Science
Pce_013			Atlantic Specimen		University of Miami - Rosenstiel School of Marine and Atmospheric Science
Pce_014			South-eastern Pacific		Universidad Austral de Chile, Universidad Catolica del Norte
Pce_015			Islands of the Californian Coast, Chaetopterus tubes from Mission Bay		University of California
Pce_016			Islands of the Californian Coast, Chaetopterus tubes from Mission Bay		University of California

no	CC License	accession number	museum	geographic information	cruise
Pce_017			rocky Shore of Okinawa Islands in the Ryukyu Islands		Universtiy of Ryukyus
Pce_018			Tropical Western North Antlantic		Smithsonian Institution
Pce_019			northern coasst of South America and Lesser Antilles		Justus-Liebig-Universität Gießen
Pce_020			Western Atlantic Waters		Universidad de Oriente
Pce_021			Californian Coast north of San francisco		University of the Pacific
Pce_022			Gulf of Ecuador to Ecuador and the Galapagos		Universidad Simon Bolivar
Pce_023			Caribbean Sea		Universidad de Oriente

no	CC License	accession number	museum	geographic information	cruise
Pce_024			Ryuku Islands, Japan		National University of Singapore
Pce_025			Ryuku Islands, Japan		National University of Singapore
Pce_026			Philippines		National University of Singapore
Pce_027			Ryuku Islands, Japan		National University of Singapore
Pce_028			Ryuku Islands, Japan		National University of Singapore
Pce_029			Ryuku Islands, Japan		National University of Singapore

no	CC License	accession number	museum	geographic information	cruise
Pce_030			Ryuku Islands, Japan		National University of Singapore
Pce_031			Ryuku Islands, Japan		National University of Singapore
Pce_032			Ryuku Islands, Japan		National University of Singapore
Pce_033			Ryuku Islands, Japan		National University of Singapore
Pce_034			Ryuku Islands, Japan		National University of Singapore
Pce_035			Ryuku Islands, Japan		National University of Singapore

no	CC License	accession number	museum	geographic information	cruise
Pce_036			Ryuku Islands, Japan		National University of Singapore
Pce_037			Ryuku Islands, Japan		National University of Singapore
Pce_038			Western Atlantic Waters		Smithonian Institution
Pce_039			Manora Islands, Karachi		University of Karachi, Pakistan
Pce_040			Eastern Pacific		Pacific Science 1975
Pce_041			along many rocky shorts p.e. Puerto Rico		University of Lousiana
Pce_042			Coastal and shallow water habitats		Instituto del Mar y Lomnologia
Pce_043			Southern Caribbean Sea		Justus-Liebig-Universität Gießen

no	CC License	accession number	museum	geographic information	cruise
Pce_044			Western Atlantic		University of Puerto Rico
Pce_045			Western Atlantic		University of Puerto Rico
Pce_046			Western Atlantic		University of Puerto Rico
Pce_047			Western Atlantic		University of Puerto Rico
Pce_048			Western Atlantic		University of Puerto Rico
Pce_049			Western Atlantic		University of Puerto Rico

no	CC License	accession number	museum	geographic information	cruise
Pce_050			Western Atlantic		University of Puerto Rico
Pce_051			Western Atlantic		University of Puerto Rico
Pce_052			Western Atlantic		University of Puerto Rico
Pce_053			Eastern Pacific		Instituto del Mar y Lomnologia
Pce_054			Peru, Chile		The Curstacean Society
Pce_055			Pacific Coast		Oregon State University
Pce_056			Pacific Coast		Oregon State University
Pce_057			Pacific Coast		Oregon State University
Pce_058			Pacific Coast		Oregon State University
Pce_059			Pacific Coast		Oregon State University
Pce_060			Red Sea		University of Karachi, Pakistan

no	CC License	accession number	museum	geographic information	cruise
Pce_061			Chilean Waters		Universidad de Concepcion, Chile
Pce_062			Chilean Waters		Universidad de Concepcion, Chile
Pce_063			north rocky coast of Praia Grande beach		Universidade Paulista
Pce_064			Madagascar		Institut für Allgemeine und Spezielle Zoologie Gießen
Pce_065			Gulf of Thailand		Institut für Allgemeine und Spezielle Zoologie Gießen
Pce_066			South Africa to southern Japan and Polynesia		Directorate of Fisheris, Government of Sind, Pakistan
Pce_067			Tropical Regions of all Oceans		Justus-Liebig-Universität Gießen

no	CC License	accession number	museum	geographic information	cruise
Pce_068			Karwar, west coast of India		Gulbarga University & Konkan Agricultur University
Pce_069			Karwar, west coast of India		Gulbarga University & Konkan Agricultur University
Pce_070			Karwar, west coast of India		Gulbarga University & Konkan Agricultur University
Pce_071			Karwar, west coast of India		Gulbarga University & Konkan Agricultur University
Pce_072			Karwar, west coast of India		Gulbarga University & Konkan Agricultur University
Pce_073			Karwar, west coast of India		Gulbarga University & Konkan Agricultur University

no	CC License	accession number	museum	geographic information	cruise
Pce_074			Karwar, west coast of India		Gulbarga University & Konkan Agricultur University
Pce_075			littoral zone of the Pacific		University of Miami
Pce_076			Persian Gulf		National University of Singapore
Pce_077			Persian Gulf		National University of Singapore
Pce_078			Persian Gulf		National University of Singapore
Pce_079			Persian Gulf		National University of Singapore
Pce_080			inner continental sheld between Florida and Sao Paulo		Universidad de Sao Paulo
Pce_081			inner continental sheld between Florida and Sao Paulo		Universidad de Sao Paulo
Pce_082			inner continental sheld between Florida and Sao Paulo		Universidad de Sao Paulo
Pce_083			inner continental sheld between Florida and Sao Paulo		Universidad de Sao Paulo

no	CC License	accession number	museum	geographic information	cruise
Pce_084			inner continental sheld between Florida and Sao Paulo		Universidad de Sao Paulo
Pce_085			inner continental sheld between Florida and Sao Paulo		Universidad de Sao Paulo
Pce_086			inner continental sheld between Florida and Sao Paulo		Universidad de Sao Paulo
Pce_087			inner continental sheld between Florida and Sao Paulo		Universidad de Sao Paulo
Pce_088			inner continental sheld between Florida and Sao Paulo		Universidad de Sao Paulo
Port_001					
Port_002					
Port_003					
Port_004					
Port_005					
Port_006					
Port_007					
Port_008					

no	CC License	accession number	museum	geographic information	cruise
Port_009					
Port_010					
Port_011					
Port_012					
Port_013					
Port_014					
Port_015					
Port_016					
Port_017					
Port_018					
Port_019					
Port_020					
Port_021					
Port_022					
Port_023					
Port_024					
Port_025					
Port_026					
Port_027					
Port_028					
Port_029					
Port_030					
Port_031					

no	CC License	accession number	museum	geographic information	cruise
Port_032					
Port_033					
Port_034					
Port_035					
Port_036					
Port_037					
Port_038					
Port_039					
Port_040					

no	CC License	accession number	museum	geographic information	cruise
Port_041					
Port_042					
Port_043					
Port_044					
Port_045					
Port_046					
Port_047					
Port_048					
Port_049					
Port_050					
Port_051					
Port_052					

no	CC License	accession number	museum	geographic information	cruise
Port_053					
Port_054					
Port_055					
Port_056					
Port_057					
Port_058					
Port_059					
Port_060					
Port_061					
Port_062					
Port_063					
Port_064					
Port_065		USNM 1421164	Smithsonian Insitution, National Museum of Natural History		
Port_066		ICM-CSIC 9408	Instituto de Ciencias del Mar, CSIC		

no	CC License	accession number	museum	geographic information	cruise
Port_067		UF 48263-Arthropoda	Florida Museum of Natural History, UF		
Port_068		MNHN-IU-2019-5447	Museum National D'Histoire Naturel, MNHN		
Port_069		MNHN-IU-2013-2470	Museum National D'Histoire Naturel, MNHN		
Port_070		USNM 1609478	Smithsonian Insitution, National Museum of Natural History		
Port_071		USNM 1610704	Smithsonian Insitution, National Museum of Natural History		
Port_072		MNHN-IU-2013-738	Museum National D'Histoire Naturel, MNHN		
Port_073		MNHN-IU-2013-5196	Museum National D'Histoire Naturel, MNHN		
Port_074		MNHN-IU-2019-5245	Museum National D'Histoire Naturel, MNHN		

no	CC License	accession number	museum	geographic information	cruise
Port_075		USNM 1609286	Smithsonian Insitution, National Museum of Natural History		
Port_076	CC. BY 4.0	GBIF 4941485			
Port_077	CC. BY 4.0	GBIF 28219396			
Port_078		UF 41408-Arthropoda	Florida Museum of Natural History, UF		
Port_079	CC. BY 4.0				
Port_080		UF 43879-Arthropoda	Florida Museum of Natural History, UF		
Port_081		YPM IZ 040635	Yale University Peabody Museum		

no	CC License	accession number	museum	geographic information	cruise
Port_082		UF 46566-Arthropoda	Florida Museum of Natural History, UF		
Port_083		UF 49111-Arthropoda	Florida Museum of Natural History, UF		
Port_084	CC. BY 4.0				
Port_085		NMV J45533	Museums Victoria		
Port_086		UF 41715-Arthropoda	Florida Museum of Natural History, UF		
Port_087		MNHN-IU-2013-2533	Museum National D'Histoire Naturel, MNHN		
Port_088		MNHN-IU-2019-5397	Museum National D'Histoire Naturel, MNHN		
Port_089		MNHN-IU-2014-8211	Museum National D'Histoire Naturel, MNHN		

no	CC License	accession number	museum	geographic information	cruise
Port_090		MNHN-IU-2014-8332	Museum National D'Histoire Naturel, MNHN		
Port_091	CC. BY 4.0				
Port_092		USNM 1499671	Smithsonian Insitution, National Museum of Natural History		
Port_093		UF 46472-Arthropoda	Florida Museum of Natural History, UF		
Port_094		MNHN-IU-2008-11852	Museum National D'Histoire Naturel, MNHN		
Port_095		MNHN-IU-2008-11268	Museum National D'Histoire Naturel, MNHN		

no	CC License	accession number	museum	geographic information	cruise
Port_096		MNHN-IU-2008-10693	Museum National D'Histoire Naturel, MNHN		
Port_097		UF 48179-Arthropoda	Florida Museum of Natural History, UF		
Port_098		MNHN-IU-2014-8369	Museum National D'Histoire Naturel, MNHN		
Port_099		MNHN-IU-2013-6819	Museum National D'Histoire Naturel, MNHN		
Port_100		UF 53073-Arthropoda	Florida Museum of Natural History, UF		
Port_101		UF 44682-Arthropoda	Florida Museum of Natural History, UF		
Port_102		UF 58586-Arthropoda	Florida Museum of Natural History, UF		
Port_103		UF 58951-Arthropoda	Florida Museum of Natural History, UF		
Port_104		UF 58427-Arthropoda	Florida Museum of Natural History, UF		
Port_105		MNHN-IU-2019-5462	Museum National D'Histoire Naturel, MNHN		

no	CC License	accession number	museum	geographic information	cruise
Port_106		UF 43265-Arthropoda	Florida Museum of Natural History, UF		
Port_107		UF 48536-Arthropoda	Florida Museum of Natural History, UF		
Port_108		UF 35965-Arthropoda	Florida Museum of Natural History, UF		
Port_109		UF 53896-Arthropoda	Florida Museum of Natural History, UF		
Port_110		UF 53889-Arthropoda	Florida Museum of Natural History, UF		
Port_111		MNHN-IU-2013-6860	Museum National D'Histoire Naturel, MNHN		
Port_112		MNHN-IU-2013-4921	Museum National D'Histoire Naturel, MNHN		
Port_113		UF 58753-Arthropoda	Florida Museum of Natural History, UF		
Port_114		UF 52986-Arthropoda	Florida Museum of Natural History, UF		
Port_115		MNHN-IU-2013-633	Museum National D'Histoire Naturel, MNHN		

no	CC License	accession number	museum	geographic information	cruise
Port_116		MNHN-IU-2010-7972	Museum National D'Histoire Naturel, MNHN		
Port_117		MNHN-IU-2010-6268	Museum National D'Histoire Naturel, MNHN		
Port_118		UF 43314-Arthropoda	Florida Museum of Natural History, UF		
Port_119		UF 38032-Arthropoda	Florida Museum of Natural History, UF		
Port_120		UF 42984-Arthropoda	Florida Museum of Natural History, UF		
Port_121		UF 42958-Arthropoda	Florida Museum of Natural History, UF		
Port_122		UF 43299-Arthropoda	Florida Museum of Natural History, UF		
Port_125					
Port_126					
Port_127					
Port_128					
Port_129					
Port_130					

no	CC License	accession number	museum	geographic information	cruise
Port_131					
Port_132					
Port_133					
Port_134					
Port_135					
Port_136					
Port_137					
Port_138					
Port_139					
Port_140					
Port_141					
Port_142					
Port_143					
Port_144					
Port_145					
Port_146					
Port_147					
Port_148					

no	CC License	accession number	museum	geographic information	cruise
Port_149					
Port_150					
Port_151					
Port_152					
Port_153					
Port_154					
Ran_001					
Ran_002					
Ran_003					
Ran_004					
Ran_005					
Ran_006					
Ran_007					
Ran_008					
Ran_009					
Ran_010					
Ran_011					
Ran_012					
Ran_013					
Ran_014					
Ran_015					
Ran_016					

no	CC License	accession number	museum	geographic information	cruise
Ran_017					
Ran_018					
Ran_019					
Ran_020					
Ran_021					
Ran_022					
Ran_023					

no	CC License	accession number	museum	geographic information	cruise
Ran_024					
Ran_025					
Ran_026					
Ran_027					
Ran_028					
Ran_029					
Ran_030					
Xant_001					
Xant_002					

no	CC License	accession number	museum	geographic information	cruise
Xant_003					
Xant_004					
Xant_006					
Xant_007					
Xant_008					
Xant_009					
Xant_010					

no	CC License	accession number	museum	geographic information	cruise
Xant_011					
Xant_012					
Xant_013					
Xant_014					
Xant_015					
Xant_016					
Xant_017					
Xant_018					
Xant_019					
Xant_020					

no	CC License	accession number	museum	geographic information	cruise
Xant_021					
Xant_022					
Xant_023					
Xant_024					

no	CC License	accession number	museum	geographic information	cruise
Xant_025					
Xant_026					
Xant_027					
Xant_028					
Xant_029					

no	CC License	accession number	museum	geographic information	cruise
Xant_030					
Xant_031					
Xant_032					
Xant_033					
Xant_034					
Xant_035					
Xant_036					
Xant_037					

no	CC License	accession number	museum	geographic information	cruise
Xant_038					
Xant_039					
Xant_040					
Xant_041					
Xant_042					
Xant_043					

no	CC License	accession number	museum	geographic information	cruise
Xant_044					
Xant_045					
Xant_047					
Xant_048					
Xant_050					

no	CC License	accession number	museum	geographic information	cruise
Xant_051					
Xant_052					
Xant_053					

no	CC License	accession number	museum	geographic information	cruise
Xant_054					
Xant_055					
Xant_056					
Xant_057					
Xant_058					

no	CC License	accession number	museum	geographic information	cruise
Xant_059					
Xant_060					
Xant_061					
Xant_062					
Xant_063					

no	CC License	accession number	museum	geographic information	cruise
Xant_064					
Xant_065					
Xant_066					
Xant_067					
Xant_068					

no	CC License	accession number	museum	geographic information	cruise
Xant_069					
Xant_070					
Xant_071					
Xant_072					
Xant_073					

no	CC License	accession number	museum	geographic information	cruise
Xant_074					
Xant_075					

no	CC License	accession number	museum	geographic information	cruise
Xant_076					
Xant_077					

no	CC License	accession number	museum	geographic information	cruise
Xant_078					
Xant_079					
Xant_080					
Xant_081					

no	CC License	accession number	museum	geographic information	cruise
Xant_082					
Xant_083					
Xant_084					
Xant_085					

no	CC License	accession number	museum	geographic information	cruise
Xant_086					
Xant_087					
Xant_088					
Xant_089					

no	CC License	accession number	museum	geographic information	cruise
Xant_090					
Xant_091					
Xant_093					

no	CC License	accession number	museum	geographic information	cruise
Xant_094					
Xant_096					
Xant_097					

no	CC License	accession number	museum	geographic information	cruise
Xant_098					
Xant_100					
Xant_101					

no	CC License	accession number	museum	geographic information	cruise
Xant_102					
Xant_103					
Xant_104					

no	CC License	accession number	museum	geographic information	cruise
Xant_105					
Xant_106					
Xant_107					
Xant_108					

no	CC License	accession number	museum	geographic information	cruise
Xant_109					
Xant_111					
Xant_112					
Xant_113					
Xant_114					
Xant_115					
Xant_116					

no	CC License	accession number	museum	geographic information	cruise
Xant_117					
