# Supplementary material for: Morphological diversity in true and false crabs reveals the plesiomorphy of the megalopa phase
Source: Sci Rep. 2024 Apr 15;14:8682. doi: 10.1038/s41598-024-58780-7 (PMC11018780; doi:10.1038/s41598-024-58780-7)
Supplement: Supplementary file 3 — Supplementary Information 3. [file 41598_2024_58780_MOESM3_ESM.pdf]

Supplementary References S3: Reference list of material used in this study.

List of Collections and museums

AM, Sydney
CSIC, Instituto de Ciencias del Mar
FM, Florida
MNHM, Paris
NHMD, Copenhagen
NMV, Victoria
USNM, Smithsonian Insitution, National Museum of Natural History
YPM, Yale University Peabody Museum

Open Data Bases

GBIF.org

List of private collectors and photographers

Arria Belli, Wikimedia
Arthur Anker, crabdatabase.info
Gustav Paulay, crabdatabase.info
Hsi-Te Shih, crabdatabase.info
Ondřej Radosta, crabdatabase.info
Robert Lasley, crabdatabase.info
Tan Heok Hui, crabdatabase.info
Tohru Naruse, crabdatabase.info

List of References

59 Abbas, E.M., Abdelsalam, K.M., Mohammed-Geba, K., Ahmed, H.O., and Kato, M. 2016. Genetic and morphological identification of some crabs from the Gulf of Suez, Northern Red Sea, Egypt. The Egyptian Journal of Aquatic Research, 42(3):319-329. https://doi.org/10.1016/j.ejar.2016.08.003

60 Abdelsalam, K. and Ramadan, S.E. 2017. First record of two crab species from the Egyptian Mediterranean Sea. Cahiers de Biologie Marine, 58: 17-23.

61 Ahyong, S. T. (2007). Decapod Crustacea collected by the NORFANZ expedition: Galatheidae and Polychelidae. Zootaxa, 1593(1), 1-54.

62 Ahyong, S. T., Chan, T. Y., & Bouchet, P. (2010). Mighty claws: a new genus and species of lobster from the Philippine deep sea (Crustacea, Decapoda, Nephropidae). Zoosystema, 32(3), 525-535.

63 Al-Aidaroos, A. M., Kumar, A. A. J., Al-Haj, A. E., Al-Sofyani, A., Crosby, M. P., & El-Sherbiny, M. M. (2019). Morphology of the complete larval stages of Portunus segnis (Forskål, 1775)(Crustacea: Brachyura: Portunidae) from the Gulf of Aqaba, Saudi Arabia. Zootaxa, 4638(2), zootaxa-4638.

64 Albornoz, L., & Wehrtmann, I. S. (1996). Aspects of the reproductive biology of Petrolisthes laevigatus (Guérin, 1835)(Decapoda, Anomura, Porcellanidae). Part II: Description of the larval development, including the first crab stage, cultivated under laboratory conditions. Archive of Fishery and Marine Research, 43, 137-157.

65 Almón, B., Cuesta, J. A., & García‐Raso, J. E. (2022). Two new hermit crab species of Diogenes (Crustacea: Decapoda: Diogenidae) from Atlanto‐Mediterranean coasts of Iberian Peninsula: Poleward migrants or merely overlooked indigenous species?. Ecology and Evolution, 12(5), e8844.

66 Andryszak, B. L., & Gore, R. H. (1981). The complete larval development in the laboratory of Micropanope sculptipes (Crustacea, Decapoda, Xanthidae) with a comparison of larval characters in western atlantic xnathid genera. Fishery Bulletin, 79(3-4): 487-506.

67 Anosov, S. 2000. Keys to the identification of brachyuran larvae of the Black Sea. Crustaceana, 73(10):1239-1246. https://www.jstor.org/stable/20106395

68 Apreshgi, K. P., & Abraham, K. M. (2019). Brachyuran crab diversity in an isolated mangrove patch of the Cochin backwater, central Kerala, India. Journal of Aquafic Biology and Fisheries, 7(1&2), 8-14.

69 Arai, T. I., Brandão, M. C., & Freire, A. S. (2017). First record of Grapsus grapsus (Linnaeus, 1758)(Brachyura: Decapoda: Grapsidae) in freshwater habitat. Nauplius, 25, e2017032.

70 Arnaud, P. M., Beurois, J., & Noel, P. (1972). Portunidae et Grapsidae des îles Saint Paul et Amsterdam, Océan Indien (Decapoda, Brachyura). Beaufortia, 20(259), 7-14.

71 Arruda, D. C., & Abrunhosa, F. A. (2011). Redescription of megalopa and juvenile development of Pachygrapsus gracilis (Decapoda: Grapsidae) from the Amazon region, reared in the laboratory. Zoologia (Curitiba), 28, 465-478.

72 Artal, P., Van Bakel, B.W.M., Domínguez, J.L. & Gómez, G. (2016): A new dromiid crab (Crustacea, Brachyura, Dromioidea) from the Upper Eocene of Huesca (Aragón, northern Spain) Zootaxa 4061 (4): 438–446.

73 Bacon, M. R. (1971). Zonation and habits of the grapsid crabs. Plagusia capensis, 123-127.

74 van Bakel, B.W., Jagt, J.W., Fraaije, R.H., and Wille, E.R. 2004. Piacenzian (Pliocene) decapod crustacean faunules from northwest Belgium. Bulletin of the Mizunami Fossil Museum, 30:97-108.

75 van Bakel, B. W., Maerten, L., Jagt, J. W., & Fraaije, R. H. (2021). Bajoprosopon piardi n. gen. and sp. from the Middle Jurassic of France, with a revised diagnosis of the family Prosopidae von Meyer, 1860 (Brachyura, Podotremata) and notes on the availability of names introduced by Hermann von Meyer (1835, 1857). Palaeontologia Electronica, 24(2), 1-15.

76 Bandekar, P. D. (2021). Tree-climbing mangrove crabs of Karwar west coast of India. International Journal of Fisheries and Aquatic Studies, 9(3): 186-189.

77 Banerjee, S. K. (1960). Biological results of the Snellius Expedition. XVIII. The genera Grapsus, Geograpsus and Metopograpsus (Crustacea, Brachyura). Temminckia, 10, 132-199.

78 Barros-Alves, S. D. P., Alves, D. F. R., Silva, S. L. R. D., Guimarães, C. R. P., & Hirose, G. L. (2015). New records of decapod crustaceans from the coast of Sergipe state, Brazil.

79 Bartilotti, C., Calado, R., & Dos Santos, A. (2007). Complete larval development of the hermit crabs Clibanarius aequabilis and Clibanarius erythropus (Decapoda: Anomura: Diogenidae), under laboratory conditions, with a revision of the larval features of genus Clibanarius. Helgoland Marine Research, 62, 103-121.

80 Bedini, R. 2006. Polymorphism of colour patterns related to environmental factors: correlation between habitat, sight, and mimicry in Sirpus zariquieyi Gordon, 1953. Crustaceana, 79(1):53-67.

81 Верещака, А.Л. 1989. Новый вид краба – Sirpus ponticus (Crustacea, Pirimelidae) в Черном море. Зоологическийжурнал, 68(8):41-47.

82 Beschin, C., and Checchi, A. 2018. Nuovo genere e nuova specie di Carcinidae (Crustacea, Decapoda, Portunoidea) nell'Eocene dei monti lessini (Italia nordorientale). Studi e Ricerche - Associazione Amici del Museo - Museo Civico "G. Zannato" Montecchio Maggiore (Vicenza), 25:27-31.

83 Beschin, C., Busulini, A., Tessier, G., and Zorzin, R. 2016. I crostacei associati a coralli nell'Eocene inferiore dell'area di Bolca: Verona e Vicenza, Italia nordorientale. Memorie del Museo Civico di Storia Naturale di Verona - 2. serie. Sezione Scienze della Terra - N. 9.

84 Bidle, K. D., & McLaughlin, P. A. (1992). Development in the hermit crab Pagurus caurinus Hart (Decapoda: Anomura: Paguridae) reared in the laboratory. Part I. Zoeal and megalopal stages. Journal of Crustacean Biology, 12(2), 224-238.

85 Blow, W. C., & Manning, R. B. (1996). Preliminary descriptions of 25 new decapod crustaceans from the middle Eocene of the Carolinas, USA. Tulane Studies in Geology and Paleontology, 29(1), 1-26.

86 Bolla Jr., E. A. (2010). Desenvolvimento juvenil de duas espécies de siri (Crustacea, Decapoda, Portunidae) de importância econômica.

87 Bolla Jr., E. A., Negreiros-Fransozo, M. L., & Fransozo, A. (2008). Juvenile development of Callinectes ornatus Ordway, 1863 (Crustacea: Decapoda: Portunidae), from megalopae obtained in the neuston. Zootaxa, 1, 20.

88 Bolla Jr., Eduardo, A., Fransozo, V., & Negreiros-Fransozo, M. L. (2014). Juvenile development of Callinectes danae Smith, 1869 (Crustacea, Decapoda, Brachyura, Portunidae) under laboratory conditions. Anais da Academia Brasileira de Ciências, 86, 211-228.

89 Bolla Jr., E. A., & Fransozo, M. L. N. (2016). Morphology of juvenile phase of Achelous spinimanus (Latreille, 1819)(Crustacea, Decapoda, Portunidae) reared in laboratory. Journal of the Marine Biological Association of the United Kingdom, 96(3), 615-631.

90 Bookhout, C. G. (1972). Larval development of the hermit crab, Pagurus alatus Fabricius, reared in the laboratory (Decapoda, Paguridae). Crustaceana, 215-238.

91 Bookhout, C. G., & Costlow Jr, J. D. (1974). Larval development of Portunus spinicarpus reared in the laboratory. Bulletin of Marine Science, 24(1), 20-51.

92 Bookhout, C. G., & Costlow Jr, J. D. (1977). Larval development of Callinectes similis reared in the laboratory. Bulletin of Marine Science, 27(4), 704-728.

93 Bookhout, C. G., & Costlow Jr, J. D. (1979). Larval development of Pilumnus dasypodus and Pilumnus sayi reared in the laboratory (Decapoda Brachyura, Xanthidae). Crustaceana Supplement, 5: 1-16.

94 Borradaile, L.A. 1904. Marine crustaceans. XIII. The Hippidea, Thalassinidea and Scyllaridea. p. 750-754. In: J. S. Gardiner (ed), The fauna and geography of the Maldive and Laccadive Archipelagoes 2. Cambridge, Cambridge University Press.

95 Boschi, E. E., Scelzo, M. A., & Goldstein, B. (1967). Desarrollo larval de dos especies de Crustaceos Decapodos en el laboratorio, Pachycheles haigae Rodrigues da Costa (Porcellanidae) y Chasmagnathus granulata Dana (Grapsidae). Boletín del Instituto de Biología Marina, 12: 1-46. http://hdl.handle.net/1834/23373

96 Bouchard, J. M., Poupin, J., Cleva, R., Dumas, J., & Dinhut, V. (2013). Land, mangrove and freshwater decapod crustaceans of Mayotte region (Crustacea, Decapoda). Atoll Research Bulletin, 592, 1-69.

98 Boyko, C. B., & McLaughlin, P. A. (2010). Annotated checklist of anomuran decapod crustaceans of the world (exclusive of the Kiwaoidea and families Chirostylidae and Galatheidae of the Galatheoidea). Part IV–Hippoidea. The Raffles Bulletin of Zoology, 23, 139-151.

99 Braig, F., Zuluaga, V. P., Haug, C., & Haug, J. T. (2021). Diversity of hippoidean crabs-considering ontogeny, quantifiable morphology, and phenotypic plasticity. Nauplius, 29, e2021027.

100 Brodie, R., & Harvey, A. W. (2001). Larval development of the land hermit crab Coenobita compressus H. Milne Edwards reared in the laboratory. Journal of Crustacean Biology, 21(3), 715-732.

101 Brossi-Garcia, A. L., & Hebling, N. J. (1983). Desenvolvimento pós-embrionário de Clibanarius antillensis Stimpson, 1859 (Crustacea, Diogenidae), em laboratório. Boletim de Zoologia, 6(6), 89-112.

102 Bruce, A. J. (1972). The rediscovery of Notopoides latus Henderson in the western Indian ocean (Crustacea-Decapoda, Raninidae).

103 Cabezas, P., Marcpherson, E., Machordom, A. (2010). Allogalathea (Decapoda: Galatheidae): a monospecific genus of squat lobster?. Zoological Journal of Linnea Society, 201, 162.

104 Campodónico, I. (1979). Centollón, Paralomis granulosa (Jacquinot)(Crustacea Decapoda Anomura Lithodidae) En: Estado actual de las principales pesquerías nacionales. Bases para un desarrollo pesquero (Crustáceos). Corporación de Fomento de la Producción, Instituto de Fomento Pesquero. Santiago.

105 Çetin, C., Furman, A., Bilgin, R., & Kalkan, E. (2015, August). Cytonuclear discordance in the marbled crab, Pachygrapsus marmoratus (Fabricius, 1787) along the Mediterranean coasts of Turkey. In 2nd Ecology and Evolutionary Biology Symposium, Turkey (pp. 6-7).

106 Cely Moque, J. K. (2018). Colección de crustáceos de referencia presentes en el museo de ciencias de la Universidad El Bosque. Universidad El Bosque, Facultad de Ciencias.

107 Chace Jr, F. A. (1951). The oceanic crabs of the genera Planes and Pachygrapsus. Proceedings of the United States National Museum, 101: 65-103.

108 Chace Jr, F. A. (1966). Decapod Crustaceans from St. Helena lsland, South Atlantic. Proceedings of the United States National Museum, 118(3536): 623-661.

109 Chace, F. A., & Hobbs, H. H. (1969). The freshwater and terrestrial decapod crustaceans of the West Indies with special reference to Dominica. Unites States National Museum Bulletin, 292: 1-242.

110 Chang, S. C., & Chan, T. Y. (2019). On the clawed lobsters of the genus Nephropsis Wood-Mason, 1872 recently collected from deep-sea cruises off Taiwan and the South China Sea (Crustacea, Decapoda, Nephropidae). ZooKeys, 833, 41.

111 Chang, S. C., Wang, T. W., & Chan, T. Y. (2015). First record of the Nephropid genus Acanthacaris Bate, 1888 (Crustacea: Decapoda: Nephropidae) from Taiwan. Zootaxa, 4032(5), 595-599.

112 Chang, S. C., Chan, T. Y., & Kumar, A. B. (2020). Deep-sea clawed lobster Nephropsis stewarti Wood-Mason, 1872 species complex in the Indo-West Pacific (Crustacea, Decapoda, Nephropidae), with description of a new species. ZooKeys, 1008, 37.

113 Cházaro-Olvera, S., & Rocha-Ramírez, A. (2007). Morphology of the Pachygrapsus gracilis (De Saussure, 1858) megalopa (Brachyura, Grapsidae) reared in the laboratory. Crustaceana, 80(1): 19-30.

114 Cházaro-Olvera, S., Rocha-Ramírez, A., & Vázquez-López, H. (2006). Morphological differentiation of megalopae in the family Panopeidae Ortmann, 1893, from a lagoon system inlet in the southwestern Gulf of Mexico. Crustaceana, 865-878.

115 Cházaro-Olvera, S., Aguilar, I. W., Touzet, M. O., Cházaro-Martínez, E., Vázquez-López, H., & Horta-Puga, G. J. (2013). Morphology of megalopae from Diogenidae family (Decapoda, Anomura) in Veracruz, south-western Gulf of Mexico: Identification keys to genera and species. American Journal of Life Sciences, 1(6), 261-266.

116 Cházaro-Olvera, S., Robles, R., Montoya-Mendoza, J., & Herrera-López, J. A. (2018). Intraspecific variation in megalopae of Clibanarius antillensis (Anomura, Diogenidae) among western Atlantic populations. Nauplius, 26, e2018031.

117 Christiansen, M.E. und Anger, K. (1990). Complete Larval Development of Galathea intermedia Lilljeborg Reared in Laboratory Culture (Anomura: Galatheidae). Journal of Crustacean Biology, Vol. 10, No. 1 (Feb., 1990).

118 Clark, P. F., & AL-Aidaroos, A. M. (1996). The First Zoeas of Actaeodes hirsutissimus (Ruppell, 1830) and A. tomentosus (H. Milne Edwards, 1834)(Crustacea: Decapoda: Brachyura: Xanthidae: Actaeinae). Marine Scienes-Ceased lssuerg, 17(1), 1-2.

119 Clark, P. F., & Galil, B. S. (1998). The first stage zoea of Pseudoliomra speciosa (Dana, 1852)(Crustacea, Decapoda, Brachyura, Xanthidae). Zosystema-Paris, 20, 193-200.

120 Clark, P. F., & Ng, P. K. (1998). The larval development of the poisonous mosaic crab,

Lophozozymus pictor (Fabricius, 1798)(Crustacea, Decapoda, Brachyura, Xanthidae, Zosiminae), with comments on familial characters for first stage zoeas. Zoosystema-Paris, 20, 201-221.

121 Clark, P. F., & Paula, J. (2003). Descriptions of ten Xanthoidean (Crustacea: Decapoda: Brachyura) first stage zoeas from Inhaca Island. Mozambique. Raffles Bulletin of Zoology, 51(2), 323-378.

122 Costlow Jr, J. D., & Bookhout, C. G. (1959). The larval development of Callinectes sapidus Rathbun reared in the laboratory. The Biological Bulletin, 116(3), 373-396.

123 Costlow, J. D., & Bookhout, C. G. (1961). The larval stages of Panopeus herbstii Milne-Edwards reared in the laboratory. Journal of the Elisha Mitchell Scientific Society, 77(1), 33-42.

124 Crain, J. A., & McLaughlin, P. A. (2000). Larval and early juvenile development in the Lithodidae (Decapoda: Anomura: Paguroidea) reared under laboratory conditions: 1. Subfamily Lithodinae: Lopholithodes mandtii Brandt, 1848. Invertebrate Reproduction & Development, 37(1), 43-59.

125 Crocetta, F., Mifsud, S., Paolini, P., Piscopo, J., & Schembri, P. J. (2011). New records of the genus Pachygrapsus (Crustacea: Decapoda) from the central Mediterranean Sea with a review of its Mediterranean zoogeography. Mediterranean Marine Science, 12(1), 75-94.

126 Crosnier, A. 1962. XVI Crustacés Décapodes – Portunidae. In Faune de Madagascar. Publiée sous les auspices du Gouvernement de la République Malgache.

127 Crosnier, A. (1965). XVIII Crustacés Décapodes – Grapsidae et Ocypodidae. In Faune de Madagascar. Publiée sous les auspices du Gouvernement de la République Malgache, 18, pls-1.

128 Crosnier, A. 2002. Révision du genre Parathranites Miers, 1886 (Crustacea, Brachyura, Portunidae). Zoosystema, 24(4):799-824.

129 Cuesta, J. A., & Rodríguez, A. (2000). Zoeal stages of the intertidal crab Pachygrapsus marmoratus (Fabricius, 1787)(Brachyura, Grapsidae) reared in the laboratory. Hydrobiologia, 436(1-3), 119-130.

130 Cuesta, J. A., Guerao, G., Schubart, C. D., & Anger, K. (2011). Morphology and growth of the larval stages of Geograpsus lividus (Crustacea, Brachyura), with the descriptions of new larval characters for the Grapsidae and an undescribed setation pattern in extended developments. Acta Zoologica, 92(3), 225-240.

131 Cuesta, J. A., Almón, B., Pérez-Dieste, J., Trigo, J. E., & Bañón, R. (2016). Role of ships' hull fouling and tropicalization process on European carcinofauna: new records in Galician waters (NW Spain). Biological invasions, 18, 619-630.

132 Dan, S., Oshiro, M., Ashidate, M., & Hamasaki, K. (2016). Starvation of Artemia in larval rearing water affects post-larval survival and morphology of the swimming crab, Portunus trituberculatus (Brachyura, Portunidae). Aquaculture, 452, 407-415.

133 Davidson, E. (1966). A new Paleocene crab from Texas. Journal of Paleontology, 211-213.

134 Davie, P. J. F., & Short, J. W. (1989). Deepwater Brachyura (Crustacea: Decapoda) from southern Queensland, Australia with descriptions of four new species. Memoirs of the Queensland Museum, 27(2), 157-187.

135 Davie, P. J. F. (1993). Deepwater xanthid crabs from French Polynesia (Crustacea, Decapoda, Xanthoidea). Bulletin du Muséum national d'Histoire naturelle, 4(14), A2.

136 DeAngeli, A., & Garassino, A. (2003). Galatheids, chirostylids and porcellanids (Crustacea, Decapoda, Anomura) from the Eocene and Oligocene of Vicenza (northern Italy). Contributions to Zoology, 72(2-3), 97-100.

137 Dell, R. K. (1963). Pachygrapsus marinus (Rathbun), a new crab for New Zealand waters. Transcations of the Royal Society of New Zealand, 3(18): 179-180.

138 Diele, K., Koch, V., Abrunhosa, F. A., de Farias Lima, J., & de Jesus de Brito Simith, D. (2010). The brachyuran crab community of the Caeté Estuary, North Brazil: Species richness, zonation and abundance. Mangrove dynamics and management in North Brazil, 251-263.

139 Dineen, J. F., Clark, P. F., Hines, A. H., Reed, S. A., & Walton, H. P. (2001). Life history, larval description, and natural history of Charybdis hellerii (Decapoda, Brachyura, Portunidae), an invasive crab in the western Atlantic. Journal of Crustacean biology, 21(3), 774-805.

140 Duguid, W. D., & Page, L. R. (2009). Larval and early post-larval morphology, growth, and behaviour of laboratory reared Lopholithodes foraminatus (brown box crab). Journal of the Marine Biological Association of the United Kingdom, 89(8), 1607-1626.

141 Duruflé, M. 1889. Description d'une nouvelle espèce du genre Blepharipoda Bulletin de la Sociéte Philomathique de Paris, 8: 92-95.

142 Ebrahimi et al. 2016: ابراهیمی, تارا, اردلان اشجع, آریا, & مورکی, (2016). شناسایی خرچنگ های پهن حقیقی
(خانواده Grapsidae) در مناطق بین جزر و مدی جزیره هرمز. دانش زیستی ایران, 11(3), 31-38. (1393-94)

143 Edmondson, C. H. (1962). Xanthidae of Hawaii (pp. 215-309). Bernice P. Bishop Museum.

144 Effendy, I., Kumar, A. A., & El-Sherbiny, M. M. (2022). First Description of the First Crabs of Thalamita Chaptalii (Audouin, 1826)(Crustacea: Brachyura: Portunidae) from the Saudi Arabian Red Sea. Thalassas: An International Journal of Marine Sciences, 38(1), 311-320.

145 Efford, I.E. and Haig, J. 1968. Two new genera and three new species of crabs (Decapoda: Anomura: Albuneidae) from Australia. Australian Journal of Zoology, 16: 897-914.

146 Eteobong, N., Lawal-Are, A. O., Bernard, E., & Island, V. (2016). Diet composition of Purple Lagoon Crab Goniopsis pelii (Herklots, 1851) from South West Nigeria. Journal of Biology, Agriculture and Healthcare, 6(21), 104-112.

147 Evans, N. 2018. Molecular phylogenetics of swimming crabs (Portunoidea Rafinesque, 1815) supports a revised family-level classification and suggests a single derived origin of symbiotic taxa. PeerJ, 6:e4260. https://doi.org/10.7717/peerj.4260

148 Fagetti, E., & Campodonico, I. (1971). Larval development of the red crab Pleuroncodes monodon (Decapoda Anomura: Galatheidae) under laboratory conditions. Marine Biology, 8(1), 70-81.

149 Faulkes, Z. 2017. The phenology of sand crabs, Lepidopa benedicti (Decapoda: Albuneidae). Journal of Coastal Research, 33: 1095-1101.

150 Feldmann, R. M., & Schweitzer, C. E. (2007). Sexual dimorphism in extinct and extant Raninidae

(Decapoda: Brachyura). Annals of Carnegie Museum, 76(1), 39-52.

151 Feldmann, R. M., Franţescu, A. & Schweitzer, C. E. (2010): A new genus and species of dromiid crab (Decapoda, Brachyura) from the middle Eocene of South Carolina. Studies on Malacostraca: Lipke Bijdeley Holthuis Memorial volume. Crustaceana Monographs. 14: 255-26.

152 Feldmann, R.M., Schweitzer, C.E., and Goedert, J.L. (2018): Complex taphonomic and depositional history of a new species of Carcinidae (Decapoda: Brachyura: Portunoidea) from Washington state, USA. Journal of Crustacean Biology, 38(5):579-585. https://doi.org/10.1093/jcbiol/ruy066

153 Ferreira, L. A. A., & Tavares, M. (2017). A new species of Pachycheles (Crustacea: Anomura: Porcellanidae), with taxonomic remarks on two other porcelain crabs from the remote oceanic archipelago of Trindade and Martin Vaz, South Atlantic Ocean. Zootaxa, 4299(4), 546-560.

154 Fielder, D. R., Greenwood, J. G., & Campbell, G. (1984). The megalopa of Charybdis feriata (Linnaeus) with additions to the zoeal larvae descriptions (Decapoda, Portunidae). Crustaceana, 46(2), 160-165.

155 Fitch, B. M., & Lindgren, E. W. (1979). Larval development of Pagurus hirsutiusculus (Dana) reared in the laboratory. The Biological Bulletin, 156(1), 76-92.

156 Flores, A.A., and Paula, J. 2000. Larval and early juvenile stages of Pirimela denticulata (Montagu, 1808)(Crustacea, Brachyura, Pirimelidae) reared in the laboratory. Journal of Natural History, 34(11):2123-2143. https://doi.org/10.1080/002229300750022367

157 Flores, A. A., Negreiros-Fransozo, M. L., & Fransozo, A. (1998). The megalopa and juvenile development of Pachygrapsus transversus (Gibbes, 1850)(Decapoda, Brachyura) compared with other grapsid crabs. Crustaceana, 197-222.

158 Fonghoy, C. (2015): Ontogeny and Larval Development of Sand Crab, Emerita sp. (Decapoda: Anomura: Hippidae) reared in laboratory. [Masters Thesis, Department of Marine Science, Chulalongkorn University]

159 Fransen, C.H.J.M. 2014. True crabs, pp. 249-358. FAO, Rome.

160 Fransozo, A., Mantelatto, F. L. M., & Negreiros-Fransozo, M. L. (1991). Larval development of Hexapanopeus paulensis Rathbun, 1930 (Crustacea, Brachyura, Xanthidae) under laboratory conditions. Revista brasileira de Zoologia, 7, 31-45.

161 Fransozo, A., Negreiros-Fransozo, M. L., Martin, J. W., & Trautwein, S. E. (2001). Morphology of the first zoeal stage of Platypodiella spectabilis (Herbst, 1794)(Decapoda, Brachyura, Xanthidae) obtained in the laboratory. Gulf and Caribbean Research, 13(1), 71-77.

162 Franţescu, A. L., Feldmann, R. M., & Schweitzer, C. E. (2010). A new genus and species of dromiid crab (Decapoda, Brachyura) from the middle Eocene of South Carolina. In Studies on Malacostraca: Lipke Bijdeley Holthuis Memorial Volume (pp. 255-267). Brill.

163 Freire, A. S., Pinheiro, M. A. A., Karam-Silva, H., & Teschima, M. M. (2011). Biology of Grapsus grapsus (Linnaeus, 1758)(Brachyura, Grapsidae) in the Saint Peter and Saint Paul Archipelago, Equatorial Atlantic Ocean. Helgoland Marine Research, 65(3), 263-273.

164 Froglia, C., and Manning, R.B. 1982. Notes on Liocarcinus pusillus (Leach) and related species.

Quaderni del Laboratorio di Tecnologia della Pesca, 3(2-5):257-266.

165 Froglia, C., & Speranza, S. (1993). First record of Dyspanopeus sayi (Smith, 1869) in the Mediterranean Sea (Crustacea: Decapoda: Xanthidae). Quaderni Istituto Ricerca Pesca Marittima, 5, 163-166.

166 Fujita, Y. (2007). First zoeas of two shallow-water galatheids, Lauriea gardineri (Laurie, 1926) and Phylladiorhynchus integrirostris (Dana, 1853)(Crustacea: Decapoda: Anomura: Galatheidae). Proceedings of the Biological Society of Washington, 120(1), 74-85.

167 Fujita, Y. (2010). Larval stages of the crinoid-associated squat lobster, Allogalathea elegans (Adams & White, 1848)(Decapoda: Anomura: Galatheidae) described from laboratory-reared material. Crustacean Research, 39, 37-53.

168 Fujita, Y. (2016). Terrestrial and semi-terrestrial decapod crustaceans from Fude-iwa Island, Miyako Group, the Ryukyu Islands, Japan. 半陸棲十脚甲殻類. 宮古島市総合博物館紀要,(20), 37-52.

169 Fujita, Y. (2017). Fauna of decapod crustaceans in Minna-jima Island, Miyako Island Group, southern Ryukyu Islands, Japan. Bull Miyakojima City Mus, 21, 91-110.

170 Fujita, Y., & Shokita, S. (2005). The complete larval development of Sadayoshia edwardsii (Decapoda: Anomura: Galatheidae) described from laboratory‐reared material. Journal of Natural History, 39(12), 865-886.

171 Fujita, Y., Baba, K., & Shokita, S. (2001). Larval development of Galathea inflata Potts, 1915 (Decapoda: Anomura: Galatheidae) described from laboratory-reared material. Crustacean Research, 30, 111-132.

172 Fujita, Y., Shokita, S., & Osawa, M. (2002). Complete larval development of Petrolisthes unilobatus reared under laboratory conditions (Decapoda: Anomura: Porcellanidae). Journal of Crustacean Biology, 22(3), 567-580.

173 Fujita, Y., Baba, K., & Shokita, S. (2003). Larval development of Galathea amboinensis (Decapoda: Anomura: Galatheidae) under laboratory conditions. Crustacean Research, 32, 79-97.

174 Gamo, S., & Muraoka, Y. (1977). Preliminary observations on megalopa larvae of brachyuran and porcelain crab-shaped anomuran Crustacea collected from the drifting seaweeds in Suruga Bay. Science Reports of the Yokohama National University Sec II, 24, 1-7.

175 Garassino, A., De Angeli, A., & Pasini, G. (2008). New decapod assemblage from the Upper Cretaceous (Cenomanian-Turonian) of Gara Sbaa, southeastern Morocco. Atti della Società italiana di Scienze naturali e del Museo civico di Storia naturale in Milano, 149(1), 37-67.

176 Garcia, L. (1994). Pachygrapsus transversus (Crustacea: Decapoda: Grapsidae) a les Illes Balears. Bolleti de la Societat d'Historia Natural de les Balears, 59-63.

177 García-Guerrero, M. U., Cuesta, J. A., Hendrickx, M. E., & Rodríguez, A. (2005a). Larval development of the eastern Pacific anomuran crab Petrolisthes robsonae (Crustacea: Decapoda: Anomura: Porcellanidae) described from laboratory reared material. Journal of the Marine Biological Association of the United Kingdom, 85(2), 339-349.

178 García-Guerrero, M. U., Rodríguez, A., Cuesta, J. A., & Hendrickx, M. E. (2005b). The complete larval development of Eurypanopeus canalensis Abele and Kim, 1989 (Crustacea: Brachyura: Panopeidae) described from laboratory reared material.

179 Garcia-Guerrero, M. U., Rodríguez, A., & Hendrickx, M. E. (2006). Larval development of the eastern Pacific anomuran crab Porcellana cancrisocialis (Crustacea: Decapoda: Anomura: Porcellanidae) described from laboratory reared material. Journal of the Marine Biological Association of the United Kingdom, 86(5), 1123-1132.

180 Gherardi, F., & McLaughlin, P. A. (1995). Larval and early juvenile development of the tube-dwelling hermit crab Discorsopagurus schmitti (Stevens)(Decapoda: Anomura: Paguridae) reared in the laboratory. Journal of Crustacean Biology, 15(2), 258-279.

181 Ghory, F. S., & Siddiqui, F. A. (2007). Distributional patterns of brachyuran larvae in Manora Channel (Karachi, Pakistan) collected during 1995. Pakistan Journal of Marine Sciences, 16(1): 49-68.

182 Gillespie, G.E., Phillips, A.C., Paltzat, D.L., and Therriault, T.W. 2007. Status of the European Green Crab, Carcinus maenas, in British Columbia, 2006. Canadian Technical Reports of Fisheries and Aquatic Sciences, 2700:7.

183 Goldstein, B., & Bookhout, C. G. (1972). The larval development of Pagurus pride Auxi Leach, 1814, under laboratory conditions (Decapoda, Paguridea). Crustaceana, 263-281.

184 Gomalanon, P. 2016. Species and distribution of sand crabs (Crustacea: Hippoidea) in Chalathat Beach, Songkhla Province. Songkhla, Prince of Songkla University, 100p., Avilable at , Avilable at https://kb.psu.ac.th/psukb/bitstream/2016/11390/1/416972.pdf Accessed on 15 February 2020. [In Thai with English abstract]. https://kb.psu.ac.th/psukb/bitstream/2016/11390/1/416972.pdf

185 Gonor, S. L., & Gonor, J. J. (1973). Descriptions of the larvae of four North Pacific Porcellanidae. Fishery Bulletin, 71(1), 189-224.

186 Gore, R. H. (1971a). Petrolisthes tridentatus: The development of larvae from a Pacific specimen in laboratory culture with a discussion of larval characters in the genus (Crustacea: Decapoda; Porcellanidae). The Biological Bulletin, 141(3), 485-501.

187 Gore, R. H. (1971b). Megalobrachium poeyi (Crustacea, Decapoda, Porcellanidae): comparison between larval development in Atlantic and Pacific specimens reared in the laboratory. Pacific Science, 25: 404-425.

188 Gore, R. H. (1972). Petrolisthes platymerus: The development of larvae in laboratory culture (Crustacea: Decapoda; Porcellanidae). Bulletin of Marine Science, 22(2), 336-354.

189 Gore, R. H. (1973). Studies on decapod Crustacea from the Indian River Region of Florida. II. Megalobrachium soriatum (SAY, 1818): The larval development under laboratory culture (Crustacea: Decapoda; Porcellanidae). Bulletin of Marine Science, 23(4), 837-856.

190 Gore, R. H. (1975). Petrolisthes zacae Haig, 1968 (Crustacea, Decapoda, Porcellanidae): the development of larvae in the laboratory. Pacific Science, 29(2): 181-196.

191 Gore, R. H. (1977). Neopisosoma angustifrons (Benedict, 1901): the complete larval development under laboratory conditions, with notes on larvae of the related genus Pachycheles (Decapoda

Anomura, Porcellanidae). Crustaceana, 33(3): 284-300.

192 Gore, R. H. (1978). Larval development in the Galatheidae (Crustacea: Anomura). Fishery Bulletin, 76(4), 781.

193 Gore, R. H., & Abele, L. G. (1973). Three new species of porcellanid crabs (Crustacea, Decapoda, Porcellanidae) from the Bay of Panama and adjacent Caribbean waters. Bulletin of Marine Science, 23(3), 559-573.

194 Gore, R. H., & Scotto, L. E. (1982). Cyclograpsus integer H. Milne Edwards, 1837 (Brachyura, Grapsidae): the complete larval development in the laboratory, with notes on larvae of the genus Cyclograpsus. Fishery Bulletin, 80(3), 501-521.

195 Gordon, I. 1952. On a new crab from Cadaqués on the north east coast of Spain (Sirpus zariquieyi ng and. sp.). Eos, Rev. Esp. Ento., 28(4):303-314.

196 Greenwood, J. G. (1965). The Larval Development of Petrolisthes elongatus (H. Milne Edwards) and Petrolisthes nov aezelandiae Filhol (Anomura, Porcellanidae) with Notes on Breeding. Crustaceana, 8(3): 285-307.

197 Greenwood, J. G., & Fielder, D. R. (1979). The zoeal stages and megalopa of Portunus rubromarginatus (Lanchester)(Decapoda: Portnnidae), reared in the laboratory. Journal of Plankton Research, 1(2), 191-205.

198 Greenwood, J. G., & Fielder, D. R. (1983). Description of the later zoeal stages and megalopa of Charybdis truncata (Fabricius, 1798)(Crustacea: Portunidae). Journal of Natural History, 17(1), 15-30.

199 Griffin, D. J. G. (1973). A revision of the two southern temperate shore crabs Leptograpsus variegatus (Fabricius) and Plagusia chabrus (Linnaeus)(Crustacea, Decapoda, Grapsidae). Journal of the Royal Society of New Zealand, 3(3), 415-440.

200 Guerao, G., Abello, P., & Cuesta, J. A. (1997). Morphology of the megalopa and first crab stage of the mediolittoral crab Pachygrapsus marmoratus (Brachyura, Grapsidae, Grapsinae). ZOOSYSTEMA-PARIS-, 19, 437-448.

201 Guerao, G., Schubart, C. D., & Cuesta, J. A. (2001). The first zoeal stages of Grapsus grapsus (Linnaeus) and Geograpsus lividus (H. Milne Edwards)(Decapoda, Brachyura, Grapsidae) from the western Atlantic. Nauplius, 9(2), 111-121.

202 Guerao, G., Abelló, P., & Díaz, D. (2005). The complete larval development of the crab Pilumnus spinifer (Brachyura: Xanthoidea: Pilumnidae) reared in the laboratory. Journal of Natural History, 39(35), 3187-3216.

203 Guerao, G., Abelló, P., and Dos Santos, A. 2006. Morphological variability of the megalopa of Liocarcinus depurator(Brachyura: Portunidae) in Mediterranean and Atlantic populations. Journal of Natural History, 40(32-34):1851-1866. https://doi.org/10.1080/00222930601046584

204 Guerao, G., and Abelló, P. 2011. Early juvenile development of Mediterranean Liocarcinus depurator (Crustacea: Decapoda: Brachyura: Portunidae). Journal of Natural History, 45(35-36):2171-2189. https://doi.org/10.1080/00222933.2011.590948

205 Haig, J., Murugan, T., and Nair, N.B. 1986. Hippa indica, a new species of mole crab (Decapoda, Anomura, Hippidae) from the south west coast of India. Crustaceana, 51: 286-292.

206 Hall, W. R. (1972). The larval morphology of three species of Pagurus (Decapoda, Anomura) from California. University of the Pacific, Thesis. https://scholarlycommons.pacific.edu/uop_etds/1779

207 Harvey, A., Boyko, C. B., McLaughlin, P. & Martin, J. W. (2014): Anomura. In: Martin JW, Olesen J, Høeg JT, (eds): Atlas of Crustacean Larvae. 185-89, Baltimore: The Johns Hopkins University Press.

208 Harshith, U. P., Apoorva, M. D., Precilla, D. S., & Anita, D. D. (2016). Crabs diversity in mangrove and coastal ecosystem. In Conference on Conservation and Sustainable Management of Ecologically Sensitive Regions in Western Ghats (pp. 360-366).

209 Hart, J. F. (1935). The larval development of British Columbia brachyura: I. Xanthidae, Pinnotheridae (in part) and Grapsidae. Canadian Journal of Research, 12(4), 411-432.

210 Hart, J. F. (1965). Life history and larval development of Cryptolithodes typicus Brandt (Decapoda, Anomura) from British Columbia. Crustaceana, 255-276.

211 Haug, J. T., & Haug, C. (2014). Eoprosopon klugi (Brachyura)–the oldest unequivocal and most "primitive" crab reconsidered. Palaeodiversity, 7, 149-158.

212 Haug, J. T., Martin, J. W., & Haug, C. (2015). A 150-million-year-old crab larva and its implications for the early rise of brachyuran crabs. Nature Communications, 6(1), 6417.

213 Haynes, E. B. (1993). Stage-I zoeae of laboratory-hatched Lopholithodes mandtii (Decapoda, Anomura, Lithodidae). Fishery Bulletin, 91, 379-381.

214 Heasman, K. G., & Jeffs, A. G. (2019). Fecundity and potential juvenile production for aquaculture of the New Zealand scampi, Metanephrops challengeri (Balss, 1914)(Decapoda: Nephropidae). Aquaculture, 511, 634184.

215 Hebling, N. J., & Negreiros-Fransozo, M. L. (1983). Desenvolvimento pós-embrionário de Paguristes tortugae Schmitt, 1933 (Decapoda, Diogenidae), em laboratório. Boletim de Zoología, 6(6), 157-176.

216 Herrick, F. H. (1895). The American lobster: a study of its habits and development (Vol. 15). US Government Printing Office.

217 Hernández, G., Graterol, K., Álvarez, A., & Bolaños, J. (1998). Larval development of Porcellana sayana (Leach, 1820)(Crustacea: Decapoda: Porcellanidae) under laboratory conditions. Nauplius, 6, 101-118.

218 Hernández, G., Graterol, K., Bolaños, J. A., & Gaviria, J. I. (2002). Larval development of Megalobrachium roseum (Decapoda: Anomura: Porcellanidae) under laboratory conditions. Journal of Crustacean Biology, 22(1), 113-125.

219 Hiller, A., Viviani, C. A., & Werding, B. (2010). Hypercarcinisation: an evolutionary novelty in the commensal porcellanid Allopetrolisthes spinifrons (Crustacea: Decapoda: Porcellanidae). Nauplius, 18(1), 95-102.

220 Hogarth, P.J. 1978. Variation in the carapace pattern of juvenile Carcinus maenas. Marine Biology, 44(4):337-343. https://doi.org/10.1007/BF00390898

221 Holthuis, L. B. (1991). Marine lobsters of the world. FAO fisheries synopsis, 13(125), I.

222 Hong, Y.S. (1969). The Larval Development of Pagurus lanuginosus do Haan (Crustacea, Anomura) Reared in the Laboratory. Korean Journal of Fisheries and Aquatic Sciences, 2(1), 1-15.

223 Hong, S. Y., & Williamson, D. I. (1986). The larval development of Petalomera japonica (Henderson)(Decapoda, Dromiidae) reared in the laboratory. Journal of natural history, 20(5), 1259-1278.

224 Hsueh, P. W. (2015). A new species of Emerita (Decapoda, Anomura, Hippidae) from Taiwan, with a key to species of the genus. Crustaceana, 88(3), 247-258.

225 Huang, M. C., & Kawai, T. (2020). Observations on Metanephrops neptunus (Bruce, 1965)(Crustacea: Astacidea: Nephropidae) from the Pratas Islands, South China Sea. Crustacean Research, 49, 187-196.

226 Innocenti, G., Schubart, C. D., & Fratini, S. (2020). Description of Metopograpsus cannicci, new species, a pseudocryptic crab species from East Africa and the Western Indian Ocean (Decapoda: Brachyura: Grapsidae). The Raffles Bulletin of Zoology, 68, 619-628.

227 Islam, M. S., Shokita, S., & Higa, T. (2000). Larval development of the swimming crab Charybdis natator (Crustacea: Brachyura: Portunidae) reared in the laboratory. Species Diversity, 5(4), 329-349.

228 Islam, M. S., Machiko, K., & Shokita, S. (2005). Larval development of the swimming crab Thalamita pelsarti Montgomery, 1931 (Crustacea: Brachyura: Portunidae) reared in the laboratory. Russian Journal of Marine Biology, 31, 78-90.

229 Itoh, T. (2020). 駿河湾初記録となるハシリイワガニモドキ Metopograpsus thukuhar (Owen, 1839)(甲殻類: 十脚目: イワガニ科). 神奈川自然誌資料, 2020(41), 17-20.

230 Johnson, M.W. and Lewis, W.M. 1942. Pelagic larval stages of the sand crabs Emerita analoga (Stimpson), Blepharipoda occidentalis Randall, and Lepidopa myops Stimpson. Biological Bulletin, 83: 67-87.

231 Josileen, J., & Menon, N. G. (2004). Larval stages of the blue swimmer crab, Portunus pelagicus (Linnaeus, 1758)(Decapoda, Brachyura). Crustaceana, 785-803.

232 Jung, J., & Kim, W. (2019). DNA Barcoding of Six Diogenid Species (Crustacea: Decapoda: Paguroidea) in Korea. Animal Systematics, Evolution and Diversity, 35(4), 182-185.

233 Jung, J., & Park, J. K. (2022). The first report of tropical hermit crab Calcinus vachoni (Malacostraca, Decapoda, Calcinidae) in Korea and the preliminary revision of its cryptic diversity. ARPHA Preprints, 3, e94749.

234 Juwana, S., Aswandy, I., & Pangabean, M. L. (1987). Larval development of the Indonesian blue swimming crab, Portunus pelagicus (L)(Crustacea: Decapoda: Portunidae) reared in the laboratory. Marine Research in Indonesia, 26, 29-49.

235 Kakati, V. S. (1982). Larval development of the Indian grapsid crab, Metopograpsus lattifrons H. Milne-Edwards in vitro. Indian Journal of Marine Sciences, 11: 311-316.

236 Karasawa, H., and Fudouji, Y. 2000. Palaeogene decapod Crustacea from the Kishima and Okinoshima Groups, Kyushu, Japan. Paleontological Research, 4(4):239-253. https://doi.org/10.2517/prpsj.4.239

237 Karasawa, H., & Hayakawa, H. (2000). Additions to Cretaceous decapod crustaceans from Hokkaido, Japan: Part 1. Nephropidae, Micheleidae and Galatheidae. Paleontological Research, 4(2), 139-145.

238 Kasinathan, C., Sukumaran, S., Gandhi, A., Boominathan, N., & Rajamani, M. (2007). Rare species of Spanner crab Ranina ranina (Crustacea: Brachyura: Raninidae) from Gulf of Mannar, India. Journal of the Marine Biological Association of India, 49(1), 89-90.

239 Kato, T. and Suzuki, H. 1992. Some biological observations of the mole crabs (Hippidae, Anomura, Decapoda, Crustacea) in Sagami Bay and the complete larval development of Hippa truncatifrons (Miers). Reports of the Manazuru Marine Laboratory for Science Education, Faculty of Education, Yokohama University, 8: 77-97. (In Japanese with English abstract)

240 Kato, H., and Karasawa, H. 1994. Minohellenus macrocheilus sp. nov. (Decapoda: Crustacea) from the Oligocene Ashiya Group, Kyushu, Japan. Bulletin of Kitakyushu Museum of Natural Hsitory, 13:51-58.

241 Kensley, B. F. (1970). A small collection of decapod Crustacea from Mozambique. Annals of the South African Museum, 57(5): 103-123.

242 Kim, K.B., and Hong, S.Y. 1999. Larval development of the wrinkled swimming crab Liocarcinus corrugatus (Decapoda: Brachyura: Portunidae) reared in the laboratory. Journal of Crustacean Biology, 19(4):792-808. https://doi.org/10.1163/193724099X00510

243 Kim, M. H., & Hong, S. Y. (2000). Larval development of Cryptolithodes expansus Miers (Decapoda: Anomura: Lithodidae) reared in the laboratory. Proceedings-Biological Society of Washington, 113(1), 54-65.

244 Kim, M. H., & Hong, S. Y. (2005). Larval development of Pagurus pectinatus (Stimpson)(Decapoda: Anomura: Paguridae) reared in the laboratory. Invertebrate reproduction & development, 47(2), 91-100.

245 Kim, M. H., & Hong, S. Y. (2010). Larval development of Rhinolithodes wosnessenskii Brandt (Decapoda: Anomura: Lithodidae) reared in the laboratory. Animal cells and systems, 14(2), 115-123.

246 Kim, M. H., Hong, S. Y., Son, M. H., & Huh, S. H. (2007). Larval Development of Pagurus simulans (Decapoda, Anomura, Pagueridae) Reared in the Laboratory. Crustaceana, 327-343.

247 Knight, M. D. (1966). The larval development of Polyonyx quadriungulatus Glassell and Pachycheles rudis Stimpson (Decapoda, Porcellanidae) cultured in the laboratory. Crustaceana, 75-97.

248 Knight, M. D. (1967): The Larval Development of the Sand Crab Emerita rathbunae Schmitt

(Decapoda, Hippidae). – Pacific Science, 21: 58–76.

249 Knight, M. D. (1968): The larval development of Raninoides benedicti Rathbun (Brachyura, Raninidae), with notes on the Pacific records of Raninoides laevis (Latreille). Crustaceana, suppl. 2: 145-169.

250 Knight, C. R. (2014). Taxonomy, diversity, and distribution patterns of portunid crab megalopae in the northern Gulf of Mexico during fall of 2003.

251 Ko, H. S. (2006). Complete larval development of Novactaea pulchella (Crustacea: Decapoda: Xanthidae). Integrative Biosciences, 10(1), 7-14.

252 Ko, S. H., An, S. H., & Sulkin, S. (2004). Zoeal development of Palapedia integra (Decapoda: Brachyura: Xanthidae) reared in the laboratory. Journal of Crustacean Biology, 24(4), 637-651.

253 Kocataş, A. 1982. On the occurrence of Sirpus zariquieyi Gordon (Decapoda brachyura) in the Black Sea and Sea of Marmara. Crustaceana, 43(2):177-180. https://doi.org/10.1163/156854082X00506

254 Koch, M., and Duris, Z. 2016. Notes on distribution of some portunid crabs in the Mediterranean Sea (Decapoda: Brachyura: Portunidae). Acta Musei Silesiae. Scientiae Naturales, 65(2):117. https://doi.org/10.1515/cszma-2016-0015

255 Komai, T. (2009). A new species of the hermit crab genus Catapaguroides (Crustacea: Decapoda: Anomura: Paguridae) from shallow water in the Ryukyu Islands, Japan. Species Diversity, 14(3), 197-205.

256 Kondylatos, G., Crocetta, F., Corsini-Foka, M., and Froglia, C. 2020. Crustacea Decapoda from the Rhodes Island area (eastern Mediterranean): new records and an updated checklist. Diversity, 12(6):246. https://doi.org/10.3390/d12060246

257 Konishi, K. 1987a. Larval development of the spiny sand crab Lophomastix japonica (Durufle, 1889) (Crustacea, Anomura, Albuneidae) under laboratory conditions. Publications of the Seto Marine Biological Laboratory, 32: 123-139.

258 Konishi, K. (1987b). The larval development of Pachycheles stevensii Stimpson, 1858 (Crustacea: Anomura: Porcellanidae) under laboratory conditions. Journal of Crustacean Biology, 7(3), 481-492.

259 Konishi, K., & Imafuku, M. (2000). Hatchling of the symmetrical hermit crab Pomatocheles jeffreysii Miers, 1879: the first information on pylochelid larva (Anomura: Pylochelidae). Crustacean Research, 29, 65-69.

260 Konishi, K., & Saito, T. (2000). Larvae of the deep-sea squat lobsters, Agononida incerta (Henderson, 1888) and Munida striola Macpherson and Baba, 1993 with notes on larval morphology of the family (Crustacea: Anomura: Galatheidae). Zoological science, 17(7), 1021-1029.

261 Korn, O. M., Kornienko, E. S., & Komai, T. (2008). A reexamination of adults and larval stages of Diogenes nitidimanus (Crustacea: Decapoda: Anomura: Diogenidae). Zootaxa, 1693(1), 1-26.

262 Kornienko, E. S., & Korn, O. M. (2006). The larval development of Pagurus proximus (Decapoda:

Anomura: Paguridae) reared in the laboratory. Journal of the Marine Biological Association of the United Kingdom, 86(2), 369-381.

263 Kornienko, E. S., & Korn, O. M. (2007). Larval development of the hermit crab Pagurus gracilipes (Stimpson, 1858)(Decapoda: Anomura: Paguridae) reared in the laboratory. Invertebrate Reproduction & Development, 50(1), 31-46.

264 Kornienko, E. S., Korn, O. M., & Kashenko, S. D. (2008). Comparative morphology of larvae of coastal crabs (Crustacea: Decapoda: Varunidae). Russian Journal of Marine Biology, 34, 77-93.

265 Krishnan, T., & Kannupandi, T. (1990). Laboratory cultured zoeae, megalopa and first crab of the estuarine crab Thalamita crenata (Latr.) A. Milne Edwards 1861 (Brachyura: Portunidae). Mahasagar, 23(2), 139-152.

266 Kristensen, T., Nielsen, A.I., Jørgensen, A.I., Mouritsen, K.N., Glenner, H., Christensen, J.T., Lützen, J., and Høeg, J. T. 2012. The selective advantage of host feminization: a case study of the green crab Carcinus maenas and the parasitic barnacle Sacculina carcini. Marine biology, 159(9):2015-2023. https://doi.org/10.1007/s00227-012-1988-4

267 Krobicki, M., & Zatoń, M. (2016). A new homolodromioid crab (Brachyura: Dromiacea: Tanidromitidae) from the Bajocian of central Poland and a review of the stratigraphical distribution and paleoenvironments of the known Middle Jurassic homolodromioids. Journal of Crustacean Biology, 36(5), 695-715.

268 Kurian, A. M., & Apreshgi, K. P. (2020). Checklist of brachyuran mangrove crabs of Kerala, India. Journal of Threatened Taxa, 12(15), 17153-17160.

269 Lago, R. P. (1987). Larval development of Sesarma catenata Ortmann (Brachyura, Grapsidae, Sesarminae) reared in the laboratory. African Zoology, 22(3), 200-212.

270 Lai, J. C., Mendoza, J. C. E., Guinot, D., Clark, P. F., & Ng, P. K. (2011). Xanthidae MacLeay, 1838 (Decapoda: Brachyura: Xanthoidea) systematics: a multi-gene approach with support from adult and zoeal morphology. Zoologischer Anzeiger-A Journal of Comparative Zoology, 250(4), 407-448.

271 Langeneck, J., and Di Franco, D. 2013. Further records of two uncommon Crustaceans in Italian seas: Maja goltzianaD'Oliveira, 1888 (Decapoda Brachyura Majidae) and Xaiva biguttata (Risso, 1816)(Decapoda Brachyura Portunidae). Biodiversity Journal, 4(2):281-284.

272 Laughlin R. A., Rodríguez, P. J., Marval, J. A. (1982): The Complete Larval Development of the Sponge Crab Dromia Erythropus (George Edwards, 1771) (Brachyura: Dromiidae) from the Archipielago De Los Roques, Venezuela, Journal of Crustacean Biology, Volume 2, Issue 3. S. 342–359.

273 Lawal-Are, A. O., Moruf, R. O., Akubueze, K. L., & Adewole, O. (2019). Morphometric characteristics of two walking crabs, Cardiosoma armatum (Gecarcinidae) and Goniopsis pelii (Graspidae) from a tropical mangrove swamp in Lagos, Nigeria. Nigerian Journal of Agriculture, Food and Environment, 15(1), 83-88.

274 Lebour, M. V. (1930). The larvae of the Plymouth Galatheidae. I. Munida banffica, Galathea strigosa and Galathea dispersa. Journal of the Marine Biological Association of the United Kingdom, 17(1), 175-181.

275 Lebour, M. V. (1931). The larvae of the Plymouth Galatheidae. II. Galathea squamifera and Galathea intermedia. Journal of the Marine Biological Association of the United Kingdom, 17(2), 385-390.

276 Lebour, M.V. 1944. The larval stages of Portumnus (Crustacea Brachyura) with notes on some other genera. Journal of the Marine Biological Association of the United Kingdom, 26(1):7-15. https://doi.org/10.1017/S0025315400014429

277 Lee, S. H., & Ko, H. S. (2012). Larval stages of Areopaguristes japonicus (Miyake, 1961)(Decapoda: Anomura: Diogenidae) described from laboratory reared material. Zootaxa, 3368(1), 146-160.

278 Lemaitre, R. (1989). Revision of the genus Parapagurus (Anomura, Paguroidea, Parapaguridae), including redescriptions of the western Atlantic species. Zoologische Verhandelingen (Leiden), 253: 1-106.

279 Lemaitre, R. (1990). A review of eastern Atlantic species of the family Parapaguridae (Decapoda, Anomura, Paguroidea). Journal of Natural History, 24(1), 219-240.

280 Lemaitre, R. (1996). Hermit crabs of the family Parapaguridae (Crustacea: Decapoda: Amomura) from Australia: species of Strobopagurus Lemaitre, 1989, Sympagurus Smith, 1883 and two new genera. Records of the Australian Museum, 48: 163-221.

281 Lemaitre, R. (1997). Crustacea Decapoda: Parapaguridae from the KARUBAR Cruise in Indonesia, with descriptions of two new species. Mémoires du Muséum national d'histoire naturelle, 16: 573-596.

282 Lemaitre, R. (1998). Revisiting Tylaspis anomala Henderson, 1885 (Parapaguridae) with comments on its relationships and evolution. Zoosystema, 20(2): 289-305. Résultats des Campagnes MUSORSTROM, 20: 303-378.

283 Lemaitre, R. (1999). Crustacea Decapoda: Révision des espèces du genre Parapagurus Smith, 1879 (parapaguridae) des océans Indien et Pacifique (en anglais).

284 Lemaitre, R., & Campos, N. H. (1993). Two new hermit crabs (Crustacea: Decapoda: Paguridae) from the Caribbean sea. Proceedings of the biological Society of Washington.

285 Lemaitre, R., & McLaughlin, P. A. (1992). Descriptions of megalopa and juveniles of Sympagurus dimorphus (Studer, 1883), with an account of the Parapaguridae (Crustacea: Anomura: Paguroidea) from Antarctic and Subantarctic waters. Journal of Natural History, 26(4), 745-768.

286 Lemaitre, R., & Tavares, M. (2015). New taxonomic and distributional information on hermit crabs (Crustacea: Anomura: Paguroidea) from the Gulf of Mexico, Caribbean Sea, and Atlantic coast of South America. Zootaxa.

287 Lewinsohn, Ch. (1977): Die Dromiidae des Roten Meeres (Crustacea Decapoda, Brachyura). Zoologische Verhandelingen, 151(1), 1–41.

288 Fürst von Lieven, A., Humar, M., & Scholtz, G. (2021). Aristotle’s lobster: the image in the text. Theory in Biosciences, 140, 1-15.

289 Lörenthey, E., and Beurlen, K. 1929. Die Fossilen Dekapoden der Länder der ungarischen Krone. Geologica Hungarica Series Palaeontologica, 3:1-420.

290 Lyskin, S. A., & Britayev, T. A. (2001). Description of the megalopa of Lissocarcinus orbicularis Dana, 1852 (Decapoda: Portunidae: Caphyrinae), a crab associated with tropical holothurians Îïèñàíèå ìåãàëîïû Lissocarcinus orbicularis Dana, 1852 (Decapoda: Portunidae: Caphyrinae).

291 MacDonald, J. D., Pike, R. B., & Williamson, D. I. (1957, April). Larvae of the British species of Diogenes, Pagurus, Anapagurus and Lithodes (Crustacea, Decapoda). In Proceedings of the Zoological Society of London (Vol. 128, No. 2, pp. 209-258). Oxford, UK: Blackwell Publishing Ltd.

292 Maciel, D. C., & Alves, Â. G. C. (2009). Conhecimentos e práticas locais relacionados ao aratu Goniopsis cruentata (Latreille, 1803) em Barra de Sirinhaém, litoral sul de Pernambuco, Brasil. Biota Neotropica, 9, 29-36.

293 MacMillan, F. E. (1972). The larval development of northern California Porcellanidae (Decapoda, Anomura). I. Pachycheles pubescens Holmes in comparison to Pachycheles rudis Stimpson. The Biological Bulletin, 142(1), 57-70.

294 Macpherson, E. 1989. The identity of Xaiva pulchella MacLeay, 1838 (Decapoda, Portunidae). Crustaceana, 57(1):107-110. https://doi.org/10.1163/156854089X00428

295 Macpherson, E. (1990). Crustacea Decapoda: On a collection of Nephropidae from the Indian Ocean and western Pacific. In. A. CROSNIER (ed.), Résultats des Campagnes MUSORSTOM, Volume 6. Mém. Mus. natn. Hist. nat, (A), 145 : 289-328.

296 Macpherson, E., & Robainas-Barcia, A. (2015). Species of the genus Galathea Fabricius, 1793 (Crustacea, Decapoda, Galatheidae) from the Indian and Pacific Oceans, with descriptions of 92 new species. Zootaxa, 3913(1), 1-335.

297 Malay, M. C. M. D., Rahayu, D. L., & Chan, T. Y. (2018). Hermit crabs of the genera Calcinus Dana, Clibanarius Dana, and Dardanus Paul'son from the PANGLAO 2004 Expedition, with description of a new species and a checklist of the hermit crabs of the Philippines (Crustacea: Anomura: Paguroidea). Raffles Bulletin of Zoology, 66.

298 De Man, J. G. (1909). XXIV. The Fauna of Brackish Ponds at Port Canning, Lower Bengal. Jounral of Indian Zoology, 11: 211-232.

299 Manning, R. B. (1990). Decapod and stomatopod crustacea from ascension Island, South Atlantic Ocean. Smith. Contr. Zool., 503, iv+-l.

300 Marco-Herrero, E. 2015. Aplicación de técnicas morfológicas y moleculares en la identificación de la megalopa de decápodos braquiuros de la península ibérica. Thesis, Facultat de Ciències Biològiques, Universidad de Valencia.

301 Marco-Herrero, E., González-Gordillo, J. I., & Cuesta, J. A. (2014). Morphology of the megalopa of the mud crab, Rhithropanopeus harrisii (Gould, 1841)(Decapoda, Brachyura, Panopeidae), identified by DNA barcode. Helgoland Marine Research, 68(2), 201-208.

302 Marco-Herrero, E., Cuesta, J. A., & González-Gordillo, J. I. (2021). DNA barcoding allows identification of undescribed crab megalopas from the open sea. Scientific reports, 11(1), 20573.

303 Marin, I. (2016). Notes on holotypes of hermit crabs (Decapoda: Anomura: Paguroidea) deposited in the collection of Zoological Museum of RAS (Saint-Petersburg, Russia) with remarks on hermit crab diversity along the Russian coast of the Sea of Japan. Zootaxa, 4105(2), 171-180.

304 Martin, J. W. (1988). Phylogenetic significance of the brachyuran megalopa: evidence from the Xanthidae. In Symposium of the Zoological Society of London (Vol. 59, pp. 69-102).

305 Mashar, A.; Wardiatno, Y.; Boer, M.; Butet, N.A.; Farajallah, A. and Ardika, P. U. 2015. First record of Albunea symmysta (Crustacea: Decapoda: Albuneidae) from Sumatra and Java, Indonesia. Aquaculture, Aquarium, Conservation & Legislation, 8: 611-615.

306 Matondo, D. A. P., & Demayo, C. G. (2015). Morphological description of the red frog crab Ranina ranina Linnaeus, 1758 (Brachyura: Raninidae) from South Western Mindanao, Philippines. Journal of Entomology and Zoology Studies, 3(2), 251-256.

307 McLaughlin, P. A. (1997). Crustacea Decapoda: Pagures de la famille Paguridae récoltés lors de la campagne Karubar en Indonésie (en anglais). Résultats des Campagnes MUSORSTROM, 16: 433-572.

308 McLaughlin, P. A. (2005). The “Troglopagurus Group” of Diogenes (Decapoda: Anomura: Paguroidea: Diogenidae) revisited. Journal of Crustacean Biology, 25(4), 598-619.

309 McLaughlin, P. A., & Gore, R. H. (1988). Studies on the Provenzanoi and other pagurid groups: I. The larval stages of Pagurus maclaughlinae García-Gómez, 1982 (Decapoda: Anomura: Paguridae), reared under laboratory conditions. Journal of Crustacean Biology, 8(2), 262-282.

310 McLaughlin, P. A., & Gore, R. H. (1992). Studies on the provenzanoi and other pagurid groups: VII. The zoeal and megalopal stages of Pagurus armatus (Dana)(Decapoda: Anomura: Paguridae), reared in the laboratory. Journal of Crustacean Biology, 12(3), 448-463.

311 McLaughlin, P. A., & Lemaitre, R. (2001). A new family for a new genus and new species of hermit crab of the superfamily Paguroidea (Decapoda: Anomura) and its phylogenetic implications. Journal of Crustacean Biology, 21(4), 1062-1076.

312 McLaughlin, P. A., & Lemaitre, R. (2008). Larvae of two species of Trizocheles (Decapoda: Anomura: Paguroidea: Pylochelidae: Trizochelinae), description of the adult of one, and preliminary implications of development on pylochelid phylogeny. Zootaxa, 1911: 52-68.

313 McLaughlin, P. A., & Lemaitre, R. (2009). A new classification for the Pylochelidae (Decapoda: Anomura: Paguroidea) and descriptions of new taxa. Raffles Bulletin of Zoology, 20: 159-231.

314 McLaughlin, P. A., Gore, R. H., & Buce, W. R. (1989). Studies on the provenzanoi and other pagurid groups: III. The larval and early juvenile stages of Pagurus kennerlyi (Stimpson)(Decapoda: Anomura: Paguridae) reared in the laboratory. Journal of Crustacean Biology, 9(4), 626-644.

315 McLaughlin, Patsy A., Robert H. Gore, and Alan W. Harvey. (1991a). "Studies on the provenzanoi and other pagurid groups: V. The larval stages of Pagurus arenisaxatilis Harvey and McLaughlin, 1991 (Decapoda: Anomura: Paguridae), reared in the laboratory." Journal of Crustacean Biology 11.3: 416-431.

316 McLaughlin, P. A., Gore, R. H., & Harvey, A. W. (1991b). Studies on the provenzanoi and other

pagurid groups: V. The larval stages of Pagurus arenisaxatilis Harvey and McLaughlin, 1991 (Decapoda: Anomura: Paguridae), reared in the laboratory. Journal of Crustacean Biology, 11(3), 416-431.

317 McLaughlin, P. A., Crain, J. A., & Gore, R. H. (1992). Studies on the provenzanoi and other pagurid groups: VI. Larval and early juvenile stages of Pagurus ochotensis Brandt (Decapoda; Anomura; Paguridae) from a northeastern Pacific population, reared under laboratory conditions. Journal of Natural History, 26(3), 507-531.

318 McLaughlin, P. A., Siddiqui, F. A., & Crain, J. A. (1993). Larval and early juvenile development in Pagurus stevensae Hart, 1971 (Decapoda: Anomura: Paguridae), reared in the laboratory. Journal of Crustacean Biology, 13(2), 322-342.

319 McLaughlin, P. A., Anger, K., Kaffenberger, A., & Lovrich, G. A. (2001). Megalopal and early juvenile development in Lithodes santolla (Molina, 1782)(Decapoda: Anomura: Paguroidea: Lithodidae), with notes on zoeal variations. Invertebrate reproduction & development, 40(1), 53-67.

320 McLaughlin, P. A., Anger, K., Kaffenberger, A., & Lovrich, G. A. (2003). Larval and early juvenile development in Paralomis granulosa (Jacquinot)(Decapoda: Anomura: Paguroidea: Lithodidae), with emphasis on abdominal changes in megalopal and crab stages. Journal of Natural History, 37(12), 1433-1452.

321 McLay, C. L. (1993). Crustacea Decapoda: The sponge crabs (Dromiidae) of New Caledonia and the Philippines with a review of the genera. Résultats des campagnes MUSORSTOM, 10(5), 111-251.

322 McLay, C. L., & Ryan, P. A. (1990). The terrestrial crabs Sesarma (Sesarmops) impressum and Geograpsus crinipes (Brachyura, Grapsidae, Sesarminae) recorded from the Fiji Is. Journal of the Royal Society of New Zealand, 20(1), 107-118.

323 McLay, C. L., Jeng, M.-S., Chan, T. (2001). New Records of Dromiidae, Aethridae, and Parthenopidae from Taiwan (Decapoda, Brachyura). Crustaceana14 (9): 963-976.

324 McLay, C. L., & Ng, P. K. (2005). On a collection of Dromiidae and Dynomenidae from the Philippines, with description of a new species of Hirsutodynomene McLay, 1999 (Crustacea: Decapoda: Brachyura). Zootaxa, 1029(1), 1-30.

325 Miers, E.J. 1878. Revision of the Hippidea. Zoological Journal of the Linnean Society, 14(76): 312-336.

326 Milne-Edwards, H. and Lucas, H. 1841. Description des Crustacés nouveaux ou peu connus conservés dans la collection du muséum d'histoire naturelle. Archives du Muséum national d'Histoire naturelle Paris, 2: 461-483.

327 Micu, D., Niţă, V., & Todorova, V. (2010). First record of Say's mud crab Dyspanopeus sayi (Brachyura: Xanthoidea: Panopeidae) from the Black Sea. Marine Biodiversity Records, 3, e36.

328 Minagawa M 1990. Complete larval development of the red frog crab Ranina ranina (Crustacea, Decapoda, Raninidae) reared in the laboratory. Nippon Suisan Gakkaishi, 56(4): 577-589. DOI: 10.2331/suisan.56.577

329 Montú, M., Anger, K., de Bakker, C., Anger, V., & Loureiro Fernandes, L. (1988). Larval

development of the Brazilian mud crab Panopeus austrobesus Williams, 1983 (Decapoda: Xanthidae) reared in the laboratory. Journal of Crustacean Biology, 8(4), 594-613.

330 Moraes, C. M. (2019). Goniopsis cruentata: caracterização cinética e bioquímica da (Na+, K+)-ATPase do tecido branquial e análise da sequência de RNAm (Doctoral dissertation, Universidade de São Paulo).

331 Moraes, I. R., Davanso, T. M., Silva, A. R. D., Cobo, V. J., Alves, D. F., Santana, W., Mantelatto, F. L. & Castilho, A. L. (2022). A first report of decapod crustaceans (Anomura and Brachyura) from Laje de Santos: a no-take marine reserve in the southeast coast of Brazil. Revista mexicana de biodiversidad, 93: e933658.

332 Moraes, J. C. B., & Neigreiros-Fransozo, M. L. (2008). Desenvolvimento juvenil do caranguejo Bathyrhombila sp.(Crustacea, Decapoda, Pseudorhombilidae), a partir de megalopas obtidas no nêuston. Departamento de Zoologia, Instituto de Biociências, Universidade Estadual Paulista, Campus de Botucatu, Distrito de Rubião Júnior, s/no, Estado de São Paulo.

333 Mujica, A., González-Cornejo, F., Meerhoff, E., & Yannicelli, B. (2019). Larval development of Phylladiorhynchus pusillus (Henderson, 1885)(Decapoda, Anomura, Galatheidae). Latin american journal of aquatic research, 47(5), 774-783.

334 Müller, H. G., and Werding, B. (1990). Larval development of Petrolisthes magdalenensis Werding, 1978 (Decapoda: Anomura: Porcellanidae un der Laboratory conditions). Cah. Biol. Mar, 31, 257-270.

335 Nakano, T., & Minato, R. (2020). 西之島の潮間帯海洋生物相. 小笠原研究, 46, 109-121.

336 Nayak, V. N., & Kakati, V. S. (1983). Larval development of the hermit crab Troglopagurus manaarensis Henderson (Decapoda anomura diogenidae) observed in the laboratory.

337 Nayak, V. N. (1981). Larval development of the hermit crab Diogenes planimanus Henderson (Decapoda, Anomura, Diogenidae) in the laboratory.

338 Negri, M., Pileggi, L. G., & Mantelatto, F. L. (2012). Molecular barcode and morphological analyses reveal the taxonomic and biogeographical status of the striped-legged hermit crab species Clibanarius sclopetarius (Herbst, 1796) and Clibanarius vittatus (Bosc, 1802)(Decapoda: Diogenidae). Invertebrate Systematics, 26(6), 561-571.

339 Negri, M., Lemaitre, R., & Mantelatto, F. L. (2014). Molecular and morphological resurrection of Clibanarius symmetricus, a cryptic species hiding under the name for the “Thinstripe” Hermit Crab C. vittatus (Decapoda: Anomura: Diogenidae). Journal of Crustacean Biology, 34(6), 848-861.

340 Negreiros-Fransozo, M. L., Meyers, N., Fransozo, V., & Thorton-De Victor, S. (2007). The megalopa stage of Portunus spinimanus Latreille, 1819 and Portunus gibbesii (Stimpson, 1859)(Decapoda, Brachyura, Portunidae) from the southeastern Atlantic coast of the United States. Zootaxa, 1638(1), 21-37.

341 Ng, P. 2002. On the unusual swimming crab, Coelocarcinus foliatus Edmondson, 1930, with description of a new species from the Indian Ocean (Decapoda, Brachyura, Portunidae). Crustaceana, 75(1):51-60. http://www.jstor.org/stable/20105385

342 Ng, P. K. L., & Yang, S. L. (1998). Description of a new genus for the xanthid crab Pilodius

etisoides Takeda and Miyake, 1968 (Crustacea: Decapoda: Brachyura: Xanthoidea). Journal of Natural History, 32(10-11), 1685-1696.

343 Ng, P. K., Wang, C. H., Ho, P. H., & Shih, H. T. (2001). An annotated checklist of brachyuran crabs from Taiwan (Crustacea: Decapoda) (Vol. 11). National Taiwan Museum.

344 Ng, P. K., Guinot, D., & Davie, P. J. (2008). Systema Brachyurorum: Part I. An annotated checklist of extant brachyuran crabs of the world. The raffles bulletin of zoology, 17(1), 1-286.

345 Ng, P. K., Safaie, M., & Naser, M. D. (2012). A new species of Raphidopus Stimpson, 1858, from the Persian Gulf (Crustacea: Decapoda: Anomura: Porcellanidae). Zootaxa, 3402(1), 54-60.

346 Nichols, J. H., & Lawton, P. (1978). The occurrence of the larval stages of the lobster Homarus gammarus,(Linnaeus, 1758) off the northeast coast of England in 1976. ICES Journal of Marine Science, 38(2), 234-243.

347 Nishikawa, K. S., Negri, M., & Mantelatto, F. L. (2021). Unexpected absence of population structure and high genetic diversity of the western Atlantic hermit crab Clibanarius antillensis Stimpson, 1859 (Decapoda: Diogenidae) based on mitochondrial markers and morphological data. Diversity, 13(2), 56.

348 Nunes, D.M., Ferreira, R.C.P., Hazin, F.H., Travassos, P.E., and Souza-Filho, J.F. 2017. Deep sea decapod crustaceans of São Pedro and São Paulo Archipelago, Equatorial Atlantic, Brazil. Zootaxa, 4324(2):331-347. https://doi.org/10.11646/zootaxa.4324.2.6

349 Ogburn, M. B., Stuck, K. C., Heard, R. W., Wang, S. Y., & Forward Jr, R. B. (2011). Seasonal variability in morphology of blue crab, Callinectes sapidus, megalopae and early juvenile stage crabs, and distinguishing characteristics among co-occurring Portunidae. Journal of Crustacean Biology, 31(1), 106-113.

350 Onadeko, A. B., Lawal-Are, A. O., & Igborgbor, O. S. (2015). Habitat diversity and species richness of brachyuran crabs off University of Lagos lagoon coast, Akoka, Nigeria. The Bioscientist Journal, 3(1), 14-30.

351 Osawa, M., & McLaughlin, P. A. (2010). Annotated checklist of anomuran decapod crustaceans of the world (exclusive of the Kiwaoidea and families Chirostylidae and Galatheidae of the Galatheoidea) Part II–Porcellanidae. The Raffles Bulletin of Zoology, 23(sSuppl).

352 Ossó, A., and Stalennuy, O. 2011. Description of the first fossil species of Bathynectes (Brachyura, Polybiidae) in the Badenian (middle Miocene) of the Medobory Hills (Ukraine, Central Parathetys), with remarks on its habitat ecology. Treballs del Museu de Geologia de Barcelona, 18:37-46. https://doi.org/10.32800/tmgb.2011.18.0037

353 Özcan, T., and Ateş, A.S. 2018. Presence of Knobby Swim Crab, Macropipus tuberculatus (Roux, 1830) (Decapoda: Brachyura: Polybiidae) on the Levantine Sea Coast of Turkey. Proceeding Book, International Marine & Freshwater Sciences Symposium, Kemer-Antalya, Turkey, p. 402.

354 Özcan, T., Irmak, E., Ateş, A.S., and Katağan, T. 2009. The occurrence of Bathynectes maravigna (Decapoda: Brachyura: Portunidae) in the Turkish part of the Levantine Sea. Marine Biodiversity Records, 2:e101. https://doi.org/10.1017/S1755267209000955

355 Pancucci-Papadopoulou, M.A., and Naletaki, M. 2007. A new alien species in the Mediterranean?

On the presence of Sirpus monodi Gordon, 1953 (Brachyura, Pirimelidae) in Greece. Mediterranean Marine Science, 8(2):91-96. https://doi.org/10.12681/mms.157

356 Pastore, M.A. 1977. Presenza di Thia scutellata (Fabricius) e Xaiva biguttata (Risso) nel golfo di Taranto (mar Jonio). Thalassia Salentina, 7:83-90. https://doi.org/10.1285/i15910725v7p83

357 Pasupathi, K., & Kannupandi, T. (1986). Laboratory reared larval stages of the mangrove grapsid crab, Metopograpsus maculatus H. Milne Edwards. Mahasagar, 19(4), 233-244.

358 Paul, R., Sankolli, K. N., & Shenoy, S. (1993). Juvenile morphology and appearance of sexual appendages in two porcellanid crabs, Petrolisthes rufescens (Heller, 1861) and Pisidia gordoni (Johnson, 1970)(Decapoda, Anomura, Porcellanidae). Crustaceana, 65(3), 346-357.

359 Paula, J. 1987. Planktonic stages of brachyuran crabs from the southwestern Iberian coast (Crustacea, Decapoda, Brachyura). Journal of Natural History, 21(3):717-756. https://doi.org/10.1080/00222938700770411

360 Paula, J. 1988. The larval and post-larval development of Pennant's swimming crab, Portumnus latipes (Pennant)(Brachyura, Portunidae), reared in the laboratory. Crustaceana, 55(2):202-216. https://doi.org/10.1163/156854088X00537

361 Paulay, G. (2007). Metopograpsus oceanicus (Crustacea: Brachyura) in Hawai ‘i and Guam: Another Recent Invasive? 1. Pacific Science, 61(2), 295-300.

362 Pawar, P. R. (2017). Biodiversity of brachyuran crabs (Crustacea: Decapoda) from Uran, Navi Mumbai, west coast of India. Advances in Environmental Biology, 11(2), 103-112.

363 Pellegrini, N. C., & Gamba, A. L. (1985). Larval development of Petrolisthes tonsorius Haig, 1960, under laboratory conditions (Decapoda, Porcellanidae). Crustaceana, 49(1-3): 251-267.

364 Pinheiro, M. A. A., João, M. C. A., Leme, M. H. A., Matsunaga, A. M. F., Rio, J. P. P., & Hernáez, P. (2017). Insights of the life history in the porcellanid crab Petrolisthes armatus (Gibbes, 1850)(Crustacea: Anomura: Porcellanidae) from the Southwestern Atlantic coast. Invertebrate reproductIon & development, 61(2), 78-89.

365 Poore, G.C.B., Ahyong, S.T., Taylor, J. (2011). The Biology of Squat Lobsters. CSIRO Publishing

366 Poupin, J., Zubia, M., Gravier-Bonnet, N., Chabanet, P., & Malay, M. (2012). Illustrated checklist of the Decapoda at Europa Island. Western Indian Ocean Journal of Marine Science, 11(1), 1-25.

367 Priyadarshani, S. H. R., Jayamanne, S. C., & Hirimuthugoda, Y. N. (2010). Diversity of mangrove crabs in Kadolkele, Negombo eatuary, Sri Lanka. Sri Lanka journal of aquatic sciences, 13: 109-121.

368 Provenzano Jr, A. J., & Rice, A. L. (1964). The larval stages of Pagurus marshi Benedict (Decapoda, Anomura) reared in the laboratory. Crustaceana, 217-235.

369 Puls, A. L. (2001): Arthropoda: Decapoda. In: Shanks, A. (ed.) An identification guide to the larval marine invertebrates of the Pacific Northwest. Oregon State University Press.

370 Quintana, R., & Takeda, M. (1988): On the megalopa and first crab stages of a species of the family Xanthidae (Crustacea, Brachyura) from Japanese Waters. Bulletin of the National Science Museum

Tokyo Series A, 14(1): 27-33.

371 Raghunathan, C. (2015). Littoral faunal diversity of great Nicobar Island. Records of the Zoological Survey of India, 369, 1-172.

372 Rahayu, D. L., Shih, H. T., & Ng, P. K. (2016). A new species of land hermit crab in the genus Coenobita Latreille, 1829 from Singapore, Malaysia and Indonesia, previously confused with C. cavipes Stimpson, 1858 (Crustacea: Decapoda: Anomura: Coenobitidae). Raffles Bull. Zool. Suppl, 34, 470-488.

373 Ramírez, R., & Haroun, R. (2014). Variability in the abundance of the rock crab Grapsus adscensionis (decapoda: grapsidae) in the canary islands (Eastern Atlantic). Journal of Shellfish Research, 33(3), 787-793.

374 Raso, J.E.G., and Manjón-Cabeza, E. 1996. New record of Liocarcinus mcleayi (Barnard, 1947), new combination (Decapoda, Brachyura, Portunidae) from south Europe. Crustaceana, 69(1):84-93. https://doi.org/10.1163/156854096X00114

375 Rathbun, M.J. 1926. The fossil stalk-eyed Crustacea of the Pacific slope of North America. Bulletin of the United States National Museum, 138:155 pp.

376 Reese, E. S., & Kinzie III, R. A. (1968). The larval development of the coconut or robber crab Birgus latro (L.) in the laboratory (Anomura, Paguridea). Crustaceana. Supplement, 117-144.

377 Retamal, M. A., & Santa-Cruz, F. (2018). The larval development from prezoea to megalopa and juvenile stages of Allopetrolisthes punctatus (Guérin, 1835)(Decapoda, Anomura, Porcellanidae). Latin american journal of aquatic research, 46(4), 820-824.

378 Reyes, C.C. 2015. Biology, ecology and dynamics of Pennant's swimming crab (Portumnus latipes) in the south of Portugal (Doctoral dissertation). Thesis, Universidade do Algarve.

379 Rice, A.L. and Ingle, R.W. (1977). “Ship-Wrecked” Raninid and portunid larvae from the south-western Indian Ocean (Decapoda, Brachyura). Crustaceana, 32(1): 94-97.

380 Rice, A. L., & Provenzano, A. J. (1966). The larval development of the West Indian sponge crab Dromidia antillensis (Decapoda: Dromiidae). Journal of Zoology, 149(3), 297-316.

381 Rice, A. L., Ingle, R. W., & Allen, E. (1970). The larval development of the sponge crab, Dromia personata (L.)(Crustacea, Decapoda, Dromiidea), reared in the laboratory. Vie et Milieu, 223-240.

382 Rice, A. L., & Kristensen, I. (1982). Surface swarms of swimming crab megalopae at Curaçao (Decapoda, Brachyura). Crustaceana, 233-240.

383 Roberts Jr, M. H. (1970). Larval development of Pagurus longicarpus Say reared in the laboratory, I. Description of larval instars. The Biological Bulletin, 139(1), 188-202.

384 Rodriguez, A., & Paula, J. (1993). Larval and postlarval development of the mud crab Panopeus africanus A. Milne Edwards (Decapoda: Xanthidae) reared in the laboratory. Journal of Crustacean Biology, 13(2), 296-308.

385 Rodriguez, A., & Martin, J. W. (1997). Larval development of the crab Xantho poressa (Decapoda: Xanthidae) reared in the laboratory. Journal of Crustacean Biology, 17(1), 98-110.

386 Rodríguez, A., & Spivak, E. D. (2001). The larval development of Panopeus margentus (Decapoda: Brachyura: Panopeidae) reared in the laboratory. Journal of Crustacean Biology, 21(3), 806-820.

387 Rodríguez, I. T., Hernández, G., Magán, I., Bolaños, J. A., & Felder, D. L. (2004). Larval development of Pachycheles serratus (Decapoda: Anomura: Porcellanidae) under laboratory conditions, with notes on the larvae of the genus. Journal of Crustacean Biology, 24(2), 291-308.

388 Rodríguez, I. T., Hernández, G., & Felder, D. L. (2005). Review of the Western Atlantic Porcellanidae (Crustacea: Decapoda: Anomura) with new records, systematic observations, and comments on biogeography. Caribbean journal of Science, 41(3): 544-582.

389 Rudolf, N.R.; Haug, C. and Haug, J.T. 2016. Functional morphology of giant mole crab larvae: a possible case of defensive enrollment. Zoological Letters, 2: 17.

390 Ryan, E. P. (1956). Observations on the life histories and the distribution of the Xanthidae (mud crabs) of Chesapeake Bay. American Midland Naturalist, 56(1): 138-162.

391 Saba, M. (1976). Studies on the larvae of crabs of the family Xanthidae: I. on the larval development of Leptodius exaratus H. Milne-Edwards. Researches on Crustasea, 7, 57-67.

392 Saelzer, H. E., Quintana, R., & Quiñones, R. (1986). Larval development of Petrolisthes granulosus (Guérin, 1835)(Decapoda: Anomura: Porcellanidae) under laboratory conditions. Journal of Crustacean Biology, 6(4), 804-819.

393 Salas, C., Tirado, C., and Manjón-Cabeza, M.E. 2001. Sublethal foot-predation on Donacidae (Mollusca: Bivalvia). Journal of Sea Research, 46(1):43-56. https://doi.org/10.1016/S1385-1101(01)00064-8

394 Salgado-Barragán, J., & Ruiz-Guerrero, M. (2005). Larval development of the eastern Pacific mud crab Acantholobulus mirafloresensis (Abele and Kim, 1989)(Decapoda: Brachyura: Panopeidae) described from laboratory-reared material. Invertebrate reproduction & development, 47(2), 133-145.

395 Samuelsen, T. J. (1972). Larvae of Pagurus variabilis Milne—Edwards & Bouvier (Decapoda, Anomura) reared in the laboratory. Sarsia, 48(1), 1-12.

396 Sankolli, K. N. (1965). On a New Species of Emerita (Decapoda, Anomura) from India, with a Note on Emerita emeritus (L.). Crustaceana, 48-54.

397 Scholtz, G. (2014). Evolution of crabs–history and deconstruction of a prime example of convergence. Contributions to Zoology, 83(2), 87-105.

398 Scholtz, G. (2020). Eocarcinus praecursor Withers, 1932 (Malacostraca, Decapoda, Meiura) is a stem group brachyuran. Arthropod structure & development, 59, 100991.

399 Schubart, C. D., Cuesta, J. A., & Felder, D. L. (2005). Phylogeography of Pachygrapsus transversus (Gibbes, 1850): The effect of the American continent and the Atlantic Ocean as gene flow barriers and recognition of Pachygrapsus socius Stimpson 1871 as a valid species. Nauplius, 13(2), 99-113.

400 Schweigert, G. (2019). An additional representative of Lecythocaridae (Decapoda: Brachyura: Dromiacea) from the Upper Jurassic of southern Germany. Journal of Crustacean Biology, 39(1),

36-39.

401 Schweigert, G., & Koppka, J. (2011). Decapods (Crustacea: Brachyura) from the Jurassic of Germany and Lithuania, with descriptions of new species of Planoprosopon and Tanidromites. Neues Jahrbuch fur Geologie und Palaontologie-Abhandlungen, 260(2), 221.

402 Schweigert, G., & Robins, C. M. (2016). Earliest representatives of Lecythocaridae (Crustacea: Brachyura) from the Upper Jurassic of southern Germany. Neues Jahrbuch für Geologie und Paläontologie-Abhandlungen, 325-330.

403 Schweitzer, C.E., and Feldmann, R.M. 2000. New fossil portunids from Washington, USA, and Argentina, and a re-evaluation of generic and family relationships within the Portunoidea Rafinesque, 1815 (Decapoda: Brachyura). Journal of Paleontology, 74(4):636-653. https://doi.org/10.1666/0022-3360(2000)074<0636:NFPFWU>2.0.CO;2

404 Schweitzer, C. E., & Feldmann, R. M. (2007). A new classification for some Jurassic Brachyura (Crustacea: Decapoda: Brachyura: Homolodromioidea): families Goniodromitidae BEURLEN, 1932 and Tanidromitidae new family. Senckenbergiana lethaea, 87, 119-155.

405 Scelzo, M. A. (2004). Cangrejos Anomura: Ermitaños y chinches de arena. La vida entre mareas: vegetales y animales de las costas de Mar del Plata, Argentina. Instituto Nacional de Investigación y Desarrollo Pesquero, 213-218.

406 Schmitt, W. L. (1935). Crustacea Macrura and Anomura of Porto Rico and the Virgin Islands (Vol. 15). Academy [ie New York Academy of Sciences].

407 Schmitt, W.L. 1942. A new species of sand bug, Blepharipodida doelloi, from Argentina. Smithsonian Miscellaneous Collections, 101(18): 1-10.

408 Schweitzer, C.E., and Feldmann, R.M. 2002. New Eocene decapods (Thalassinidea and Brachyura) from Southern California. Journal of Crustacean Biology, 22(4):938-967. https://doi.org/10.1163/20021975-99990304

409 Schweitzer, C.E., and Feldmann, R.M. 2010a. New fossil decapod crustaceans from the Remy Collection, Muséum national d'Histoire naturelle, Paris. Geodiversitas, 32(3):399-415. https://doi.org/10.5252/g2010n3a3

410 Schweitzer, C. E., & Feldmann, R. M. (2010b). Sphaerodromiidae (Brachyura: Dromiacea: Dromioidea) in the fossil record. Journal of Crustacean Biology, 30(3), 417-429.

411 Schweitzer, C.E., Feldmann, R.M., and Karasawa, H. 2021. Part R, Revised, Volume 1, Chapter 8T15: Systematic descriptions: Superfamily Portunoidea. Treatise Online, 151:1-40. https://doi.org/10.17161/to.vi.15392

412 Seridji, R. 1988. Some planktonic larval stages of Albunea carabus (L., 1758) (Crustacea, Decapoda, Anomura). Journal of Natural History, 22: 1293-1300.

413 Shen, C.J. 1949. Notes on the genera Blepharipoda and Lophomastix of the family Albuneidae (Crustacea Anomura) with description of a new species, B. liberata, from China. Contributions of the Institute of Zoology of the National Academy of Peiping, 5: 153-170.

414 Siddiqi, F. A. & Ghory, F. S. (1999): Studies on the complete larval development of Actaea

jaquelinae Guinot, 1976 (Decapoda: Brachyura, Xanthidae) reared in the laboratory. Proceedings of the Seminar on Aquatic Biodiversity in Pakistan: 215-226.

415 Siddiqi, F. A. & Ghory, F. S. (2006): Complete Larval Development of Emerita holthuisi Sankolli, 1965 (Crustacea: Decapoda: Hippidae) Reared in the Laboratory. – Turk J Zool, 30: 121–135.

416 Souza, L. P. D. (2008). Maturidade sexual e relações morfométricas do caranguejo Goniopsis cruentata (Latreille, 1803) Crustacea: Brachyura: Grapsidae) do estuário do Rio Jaguaribe (Aracati-Ceará.

417 Spitzner, F., Meth, R., Krüger, C., Nischik, E., Eiler, S., Sombke, A., Torres, G., and Harzsch, S. 2018. An atlas of larval organogenesis in the European shore crab Carcinus maenas L. (Decapoda, Brachyura, Portunidae). Frontiers in Zoology, 15(1):1-39. https://doi.org/10.1186/s12983-018-0271-z

418 Squires, H. J. (1996). Larvae of the hermit crab, Pagurus arcuatus, from the plankton (Crustacea, Decapoda). Journal of Northwest Atlantic Fishery Science, 18: 43-56.

419 Stimpson, W. (1907). Report on the Crustacea (Brachyura and Anomura) Collected by the North Pacific Exploring Expedition, 1853-1856: By William Stimpson (Vol. 49, No. 2). Smithsonian Institution.

420 Stuck, K.C. and Truesdale, F.M. 1986. Larval and early development of Lepidopa benedicti Schmitt, 1935 (Anomura: Albuneidae) reared in laboratory. Journal of Crustacean Biology, 6: 89-110.

421 Stuck, K. C., & Truesdale, F. M. (1988). Larval development of the speckled swimming crab, Arenaeus cribrarius (Decapoda: Brachyura: Portunidae) reared in the laboratory. Bulletin of Marine Science, 42(1), 101-132.

422 Tan, L. W., Lim, S. S., & Ng, P. K. (1986). Larval development of the dromiid crab Cryptodromia pileifera Alcock, 1899 (Decapoda: Dromiidae) in the laboratory. Journal of Crustacean Biology, 6(1), 111-118.

423 Tanaka, H., & Konishi, K. (2001). Larval development of the poisonous crab Atergatis floridus (Linnaeus, 1767)(Crustacea, Decapoda, Xanthidae) described from laboratory-reared material. Crustacean Research, 30, 21-42.

424 Tanaka, H., Saruwatari, T., & Minami, T. (2010). Larval development of two Atergatis species (Decapoda, Xanthidae) described from laboratory-reared material. Crustacean Research, 39, 11-35.

425 Takeda, M., & Sugiyama, H. (2018). Some Freshwater, Semi-terrestrial and Land Crabs (Crustacea, Decapoda, Brachyura) from Ghana, West Africa. Bulletin of the National Museum of Nature and Science. Series A, Zoology= 国立科学博物館研究報告. A 類, 動物学/National Museum of Nature and Science, 44(2), 41-50.

426 Takeda, M., & Kurata, Y. (1977). Crabs of the Ogasawara islands IV. A collection made at the new volcanic island, Nishino-Shima-Shinto, in 1975. Bulletin of National Scientific Museum Series A, 3: 91-111.

427 Teschima, M. M. (2012). Conectividade do caranguejo Grapsus grapsus (Linnaeus, 1758) em ilhas oceânicas brasileiras. Universidade Federeal de Santa Catarina, Centro de Ciencias Biologicas.

428 Todd, P.A., Briers, R.A., Ladle, R.J., and Middleton, F. 2006. Phenotype-environment matching in the shore crab (Carcinus maenas). Marine Biology, 148(6):1357-1367. https://doi.org/10.1007/s00227-005-0159-2

429 Tshudy, D., Chan, T. Y., & Sorhannus, U. (2007). Morphology based cladistic analysis of Metanephrops: the most diverse extant genus of clawed lobster (Nephropidae). Journal of Crustacean Biology, 27(3), 463-476.

430 Tweedie, M. W. F. (1936). On the crabs of the family Grapsidae in the collection of the Raffles Museum. Bulletin of the Raffles museum, 7: 44-70.

431 Uribe A. R., Rubio R. J., Carbajal E. P., & Berrú P. P. (2013). Invertebrados marinos bentónicos del litoral de la Región Áncash, Perú. – Boletín Instituto del Mar del Perú, 28(1-2): 136–293.

432 Vieira, R. R. R., & Rieger, P. J. (2004). Larval development of Hexapanopeus caribbaeus (Stimpson, 1871)(Crustacea, Decapoda, Xanthoidea, Panopeidae) reared under laboratory conditions. Journal of Plankton Research, 26(10), 1175-1182.

433 Watts, J., Thatje, S., Clarke, S., & Belchier, M. (2006). A description of larval and early juvenile development in Paralomis spinosissima (Decapoda: Anomura: Paguroidea: Lithodidae) from South Georgia waters (Southern Ocean). Polar Biology, 29, 1028-1038.

434 Wear, R. G. (1968). Life - history studies on New Zealand Brachyura: 2. Family Xanthidae. Larvae of heterozius rotundifrons a. milne edwards, 1867, ozius Truncatus H. Milne Edwards, 1834, and Heteropanope (Pilumnopeus) serratifrons (Kinahan, 1856). New Zealand Journal of Marine and Freshwater Research, 2(2), 293-332.

435 Wear, R. G. (1970). Some larval stages of Petalomera wilsoni (Fulton & Grant, 1902)(Decapoda, Dromiidae). Crustaceana, 1-12.

436 Wear, R. G. (1976). Studies on the larval development of Metanephrops challengeri (Balss, 1914)(Decapoda, Nephropidae). Crustaceana, 113-122.

437 Werding, B. (2001). Description of two new species of Polyonyx Stimpson, 1858 from the Indo-West Pacific, with a key to the species of the Polyonyx sinensis group (Crustacea: Decapoda: Porcellanidae). Proceedings-Biological Society of Washington, 114(1), 109-119.

438 Wehrtmann, I. S., Albornoz, L., Pardo, L. M., & Véliz, D. (1997). The larval development of Petrolisthes violaceus (Guérin, 1831)(Decapoda, Anomura, Porcellanidae) from Chilean waters, cultivated under laboratory conditions. Crustaceana, 562-583.

439 Werding, B., & Müller, H. G. (1990). Larval development of Neopisosoma neglectum Werding, 1986 (Decapoda: Anomura: Porcellanidae) under laboratory conditions. Helgoländer Meeresuntersuchungen, 44, 363-374.

440 Wicksten, M. K. (2011). Decapod Crustacea of the Californian and Oregonian zoogeographic provinces. UC San Diego: Scripps Institution of Oceanography. Retrieved from https://escholarship.org/uc/item/7sk9t2dz

441 Widyastuti, E. (2016). Crab diversity at mangrove ecosystem in Lingga waters and adjacent area, Riau Islands. Zoo Indonesia, 25(1), 22-32.

442 Williamson, D. I., & Von Levetzow, K. G. (1967). Larvae of Parapagurus diogenes (Whitelegge) and some related species (Decapoda, Anomura). Crustaceana, 12(2): 179-192.

443 Ximenez, A. C. D. S. R. (2020). Período de incubação de ovos de Pachygrapsus transversus (Gibbes, 1850)(Crustacea, Brachyura, Grapsidae) em condições de laboratório.

444 Yaghmour, F., & Al Naqbi, H. (2020). First record of Columbus crab Planes minutus (Crustacea: Decapoda: Brachyura: Grapsidae) Linnaeus, 1758 for the northwestern Indian Ocean. Marine Biodiversity Records, 13(1), 7.

445 Yaqoob, M. (1977). The development of larvae of Petrolisthes ornatus Paulson, 1875 (Decapoda, Porcellanidae) under laboratory conditions. Crustaceana, 32(3): 241-255.

446 Yaqoob, M. (1979a). Larval development of Pisidia dehaanii (Krauss, 1843) under laboratory conditions (Decapoda, Porcellanidae). Crustaceana. Supplement, (5), 69-76.

447 Yaqoob, M. (1979b). Rearing of Petrolisthes lamarckii (Leach, 1820) under laboratory conditions (Decapoda, Porcellanidae). Crustaceana, 37(3), 253-264.

448 Zaouali, J., Souissi, J. B., Galil, B. S., d'Acoz, C. D. U., & Abdallah, A. B. (2008). Grapsoid crabs (Crustacea: Decapoda: Brachyura) new to the Sirte Basin, southern Mediterranean Sea—the roles of vessel traffic and climate change. Marine Biodiversity Records, 1, e73.

449 Gore, R. H. (1970). *Petrolisthes Armatus*: a Redescription of Larval Development Under Laboratory Conditions (Decapoda, Porcellanidae) 1. Crustaceana, 18(1), 75-89.

450 Guerao, G., & Abelló, P. (1999). Morphology of the early zoeal stages of *Macropipus tuberculatus* (Roux, 1830)(Crustacea, Brachyura, Portunidae). Journal of plankton research, 21(10).

451 Ko, H. S., Yang, H. J., & Ban, K. H. (2002). Zoeal Stages of Actaea semblatae (Cruistacea, Decapoda, Xanthidae), with a Key to the Known Xanthid Zoeas of Korea. Animal Systematics, Evolution and Diversity, 18(1), 1-12.

452 McLaughlin, P. A., Anger, K., Kaffenberger, A., & Lovrich, G. A. (2003). Larval and early juvenile development in Paralomis granulosa (Jacquinot)(Decapoda: Anomura: Paguroidea: Lithodidae), with emphasis on abdominal changes in megalopal and crab stages. Journal of Natural History, 37(12), 1433-1452.

453 McLaughlin, P. A., Anger, K., Kaffenberger, A., & Lovrich, G. A. (2001). Megalopal and early juvenile development in Lithodes santolla (Molina, 1782)(Decapoda: Anomura: Paguroidea: Lithodidae), with notes on zoeal variations. Invertebrate reproduction & development, 40(1), 53-67.

454 McLay, C. L. (2001). The Dromiidae of French Polynesia and a new collection of crabs (Crustacea, Decapoda, Brachyura) from the Marquesas Islands. Zoosystema-Paris-, 23(1), 77-100.

455 Rahayu, D. L. (2003). Hermit crab species of the genus Clibanarius (Crustacea: Decapoda: Diogenidae) from mangrove habitats in Papua, Indonesia, with description of a new species. Memoirs of Museum Victoria, 60(1), 99-104.

456 Rice, A., & Iingle, R. (1975). A comparative study of the larval morphology of the british portuis crabs *Macropipus puber* (L.) and M. holsatus (Fabricius), with a discussion of generic and sub-

familial larval characters within the Portunidae. Bulletin of the British Museum, 28(4): 121-151.

457 Spiridonov, V. A. (2020). An update of phylogenetic reconstructions, classification and morphological characters of extant Portunoidea Rafinesque, 1815 (Decapoda, Brachyura, Heterotremata), with a discussion of their relevance to fossil material. Geologija, 63(1), 133-166.

458 Ximenez, A. C. D. S. R. (2020). Período de incubação de ovos de Pachygrapsus transversus (Gibbes, 1850)(Crustacea, Brachyura, Grapsidae) em condições de laboratório. University of Taubaté, Brazil.

459 Ng, P. K. L., & Yang, S. L. (1998). Description of a new genus for the xanthid crab Pilodius etisoides Takeda and Miyake, 1968 (Crustacea: Decapoda: Brachyura: Xanthoidea). Journal of Natural History, 32(10-11), 1685-1696.
