# Supplementary material for: Morphological diversity in true and false crabs reveals the plesiomorphy of the megalopa phase
Source: Sci Rep. 2024 Apr 15;14:8682. doi: 10.1038/s41598-024-58780-7 (PMC11018780; doi:10.1038/s41598-024-58780-7)

Supplementary Figure S4: Morphospace of meiuran crabs. Principal components 1 and 2 of principal component analysis on the shield outline plotted against each other. A total of 1567 shields of true and false crabs were reconstructed, including zoea, megalopa, juvenile and adult developmental phases. Color-coded are major ingroups of Anomala and Brachyura.

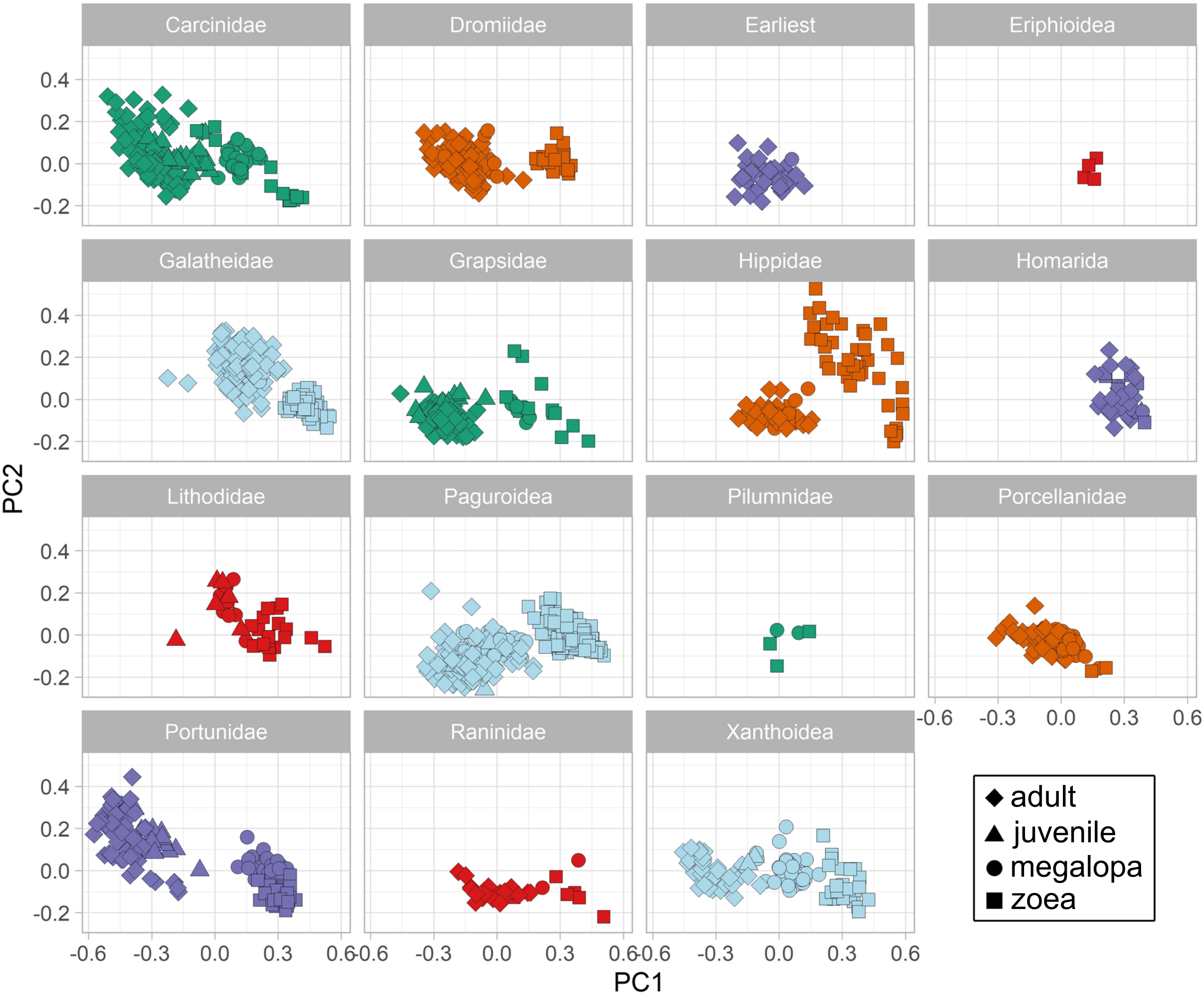

Supplement: Supplementary file 4 — Supplementary Information 4. [file 41598_2024_58780_MOESM4_ESM.pdf]
